# Cut-insert-stitch editing reaction (CIStER) sequence for surgical chemical glycan editing

**DOI:** 10.1038/s42004-024-01152-z

**Published:** 2024-04-02

**Authors:** Sumit Sen, Suman Kundu, Sandip Pasari, Srinivas Hotha

**Affiliations:** Department of Chemistry, Indian Institute of Science Education and Research Pune, Pune, 411 008 India

**Keywords:** Carbohydrate chemistry, Synthetic chemistry methodology, Synthetic chemistry methodology

## Abstract

Post-synthetic surgical editing enables synthesizing diverse molecules from a common scaffold. Editing carbohydrates by inserting a foreign glycan is still a far-reaching goal for synthetic chemists. In this study, a one-pot-three-step chemical approach was employed to edit glycoconjugates. It is comprised of three steps: the first is a ‘cut’ step, cleaving one of the interglycosidic bonds and producing an intermediate that could be intercepted with 4-mercaptotoluene; second step activates the thiotolyl glycoside in the presence of an aglycon containing an orthogonally activatable ethynylcycloxyl carbonate moiety; and the third step involves ‘stitching’ by activating the carbonate donor. The cut-insert stitch-editing reaction (CIStER) is demonstrated by inserting branched and linear arabinans reminiscent of M. tuberculosis cell wall from the same designer trimannoside. Glycosylating an activated hydroxyacid (serinyl, steroidal, and lipid) after cutting the interglycosidic bond and stitching in the presence of base extendes the CIStER approach to the synthesis of glycohybrids.

## Abstract (web summary)

Molecular editing has been used for the late-stage functionalization of chemical scaffolds at the atomic level, however, chemically editing carbohydrates by inserting a foreign glycan remains underexplored. Here, the authors develop a cut-insert-stitch editing reaction sequence to insert various carbohydrates and activated hydroxyacids into oligosaccharides.

## Introduction

Nucleic acids, proteins, carbohydrates, and lipids are among the numerous biomolecules produced by living organisms that occur in myriad sizes and structures^1^. These biomolecules perform several biological functions and have been extensively investigated^2^. Additionally, nucleic acid chemistry has advanced owing to the development of excellent molecular biology tools, such as recombinant DNA technology, polymerase chain reaction, DNA amplification, CRISPR-editing, and prime editing^3–7^. These tools have had an oblique impact on the synthesis of proteins as a direct consequence of the central dogma^8^, which states that a detailed residue-by-residue transfer of sequential information is possible in a unidirectional way from DNA to RNA, to Protein. Genomic editing techniques play a pivotal role in many fields such as agriculture, the discovery of pharmaceuticals and diagnostic tools, the production of biofuels, and to development of a better understanding of disease biology^9^. In this study, we report a surgical editing strategy (CIStER) for glycans that involves cutting an interglycosidic bond in a regiospecific manner, inserting a foreign glycan, and stitching the glycosidic bond to obtain a hybrid glycan by fine-tuning the subtle reactivity differences and inherent susceptibility to hydrolysis.

Carbohydrates are ubiquitous molecules that perform several biological functions, including signal transduction, cell-cell communication, and cellular development^10^. Carbohydrates exist as conjugates of glycans and aglycons, wherein the aglycon can be a protein, as in glycoproteins; a lipid, as in glycolipids; or a steroid, as in saponins^11^. The isolation of carbohydrates from natural systems is complicated because natural systems are innately microheterogeneous, and post-synthetic correction, amplification, and modification of synthetic or natural glycans are still in their nascent stages.

**Fig. 1:**
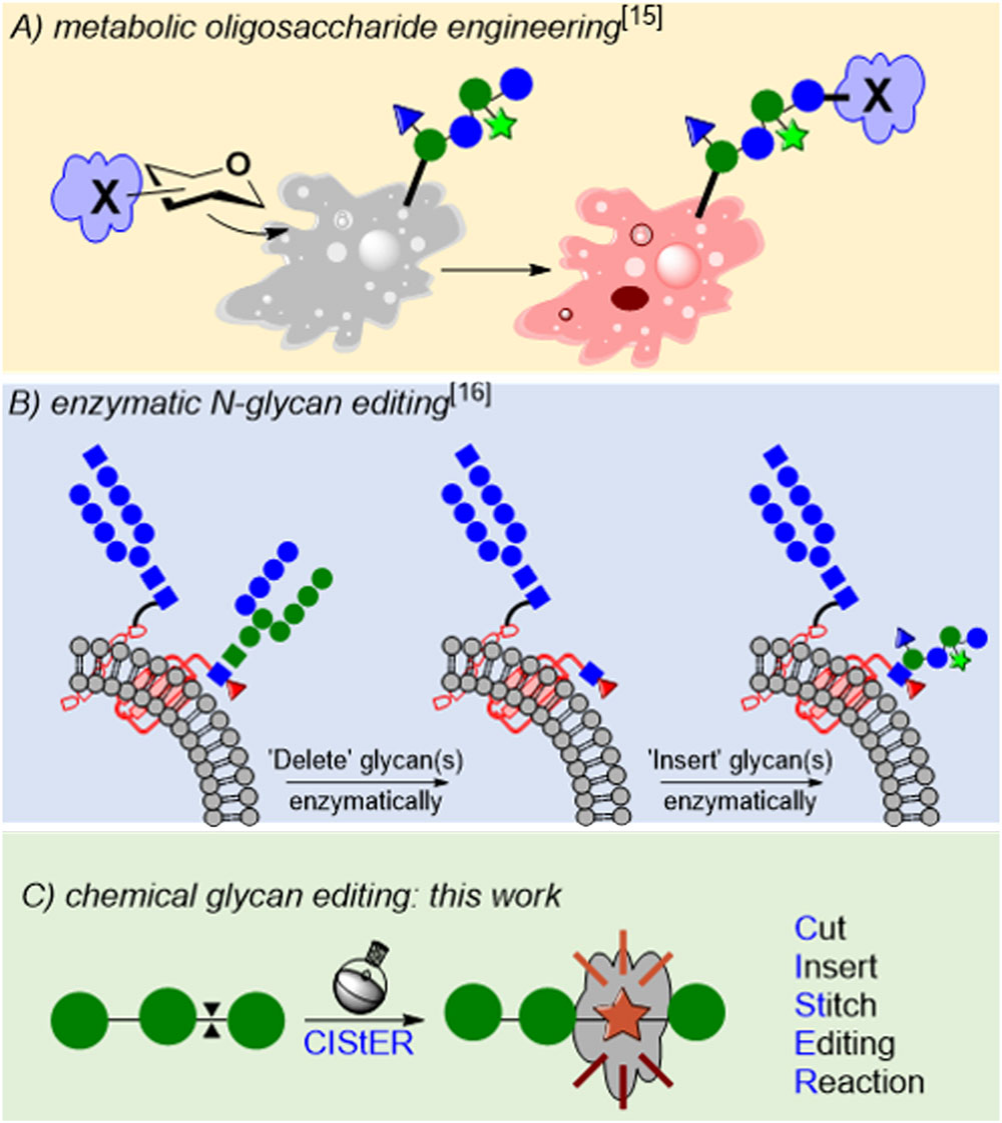
Techniques for Glycan editing. Glycan editing using metabolic oligosaccharide engineering (**A**), Enzymatic *N*-glycan editing (**B**), and Hypothesized chemical glycan editing (**C**).

Some of the most powerful approaches for saccharide editing in glycotechnology include skeletal editing *via* the acid-catalyzed transformation of furanosides to pyranosides^12^, Ferrier rearrangement for converting carbohydrates to cyclitols^13^, sugar-to-sugar transformations via oxidation followed by reduction^14^, uncontrolled editing of cell surface glycans by metabolic oligosaccharide engineering (MOE)^15^, and enzymatic addition of glycans (EAG)^16^. In MOE, cells are cultured with a monosaccharide containing a reporter moiety (e.g., alkyne/azide); hypothesizing that the conjugate of the reporter moiety and monosaccharide are incorporated into the cellular processes, the incorporated moiety can guide the investigation of biological pathways including cell imaging using bioorthogonal chemistry (Fig. 1A)^15^. This technique has been utilized for cell imaging and probing biosynthetic machinery. In contrast, EAG exploits the site-specific cleavage of glycosidic bonds using designer glycosidases, and the resulting hydrolyzed glycans are subjected to post-synthetic modifications using glycosyl transferases to attach new glycans/probes (Fig. 1B)^16^. To the best of our knowledge, no editing technique enables the insertion of a foreign glycan into the already synthesized glycans via chemical or enzymatic routes (Fig. 1C).

## Results and discussion

Despite the challenges associated with the synthesis of glycoconjugates, several innovative developments have occurred over the past century using chemical or enzymatic reactions while accomplishing good quality control^11^. The key reaction in the assembly of oligosaccharides is glycosidation, in which a glycosyl donor and aglycon condense to form a glycosidic bond. Glycosyl donors contain an appendage at the anomeric carbon (C1) that can be activated using promoters to obtain a highly reactive oxocarbenium ion intermediate; subsequently, the oxocarbenium ion intermediate can be attacked by an aglycon to afford a glycoside^11^. Several pioneering efforts have resulted in the development of glycosyl donor chemistry. Moreover, previously reported glycosyl donors have been revived via modern reagents using either stoichiometric or catalytic quantities of the reagent(s)^17^. In addition to these developments, linear and convergent strategies have been formulated for glycan assembly using latent, iterative, catalytic, or orthogonal activation protocols.

### Thioglycosyl donors and alkynyl glycosyl donors are orthogonal

Hybrid glycans can be synthesized via the surgical insertion of a foreign glycan into the parent sequence of the glycan by cutting the latter sequence to facilitate insertion of the former glycan, followed by final stitching using glycosylation. The CIStER technique requires the identification of a pair of orthogonally activatable stable glycosyl donors that can produce the desired glycosidic linkages in a chemo-specific manner with faster kinetics. Considering the suite of glycosylation protocols with stable glycosyl donors, the activation of alkynyl glycosyl carbonates using [Au]/[Ag] catalysts and thioglycosides by thiophilic reagents is promising because activation affords glycosides rapidly and in high yields. Furthermore, the reactivity and selectivity can be fine-tuned by placing the substituents at the C2-position that display the −*I* or +*I* inductive effect.

**Fig. 2:**
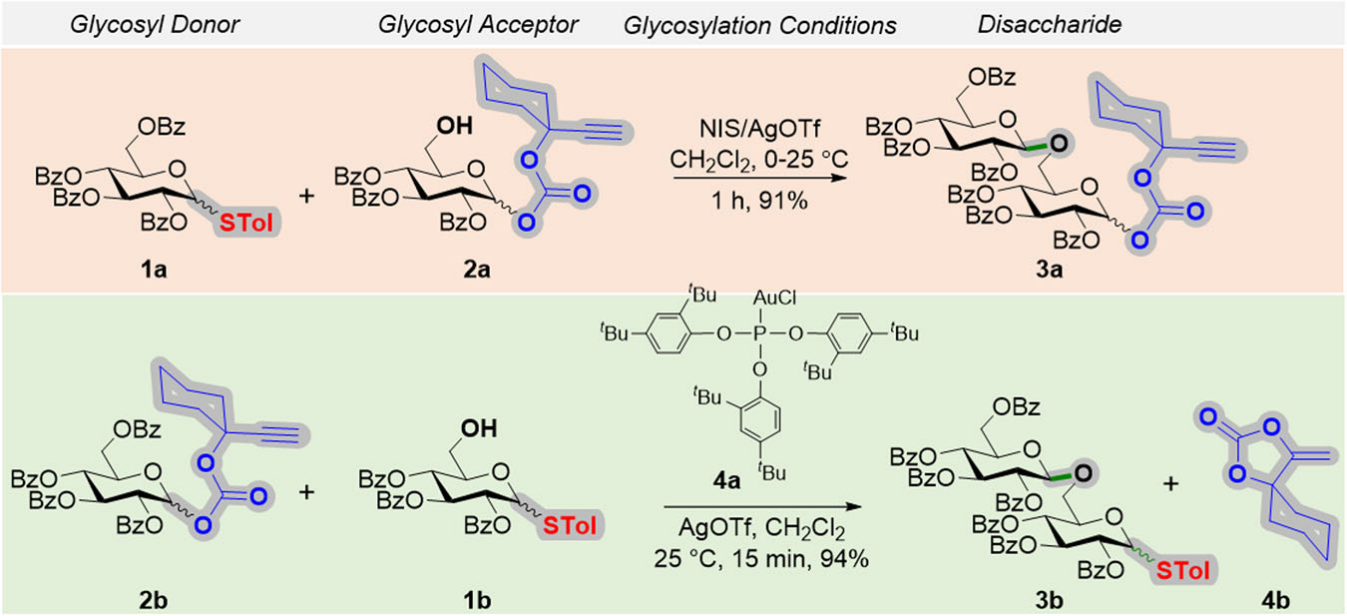
Thiotolyl and ethynylcyclohexyl carbonate donors are orthogonal. Abbreviations: STol thiotolyl, NIS N-Iodosuccinimide, Tf Trifluoromethylsulfonyl, tBu tertiary butyl, Bz benzoyl.

We started our investigation by studying the orthogonality relationship between thioglycosyl and ethynylcyclohexyl glycosyl carbonate donors. The thiotolyl, per-*O*-benzoyl-D-glucopyranoside **1a**, and aglycon **2a** reacted in the presence of *N*-iodosuccinimide/silver triflate (NIS/AgOTf) to afford gentiobioside **3a** in 91% yield; thereby confirming that the alkynyl glycosyl carbonate moiety was stable during the activation of the thiotolyl group. Similarly, ethynylcyclohexyl glucosyl donor **2b** was activated using 10 mol% each of the Au-phosphite catalyst (**4a**) and AgOTf in the presence of aglycon **1b**. Gentiobioside **3b** was produced in 94% yield along with the extrusion product **4b**, indicating that the thiotolyl group was stable. These experiments revealed that the thiotolyl glycosyl and ethynylcyclohexyl glycosyl carbonate donors were orthogonal to each other (Fig. 2)^18^.

### Cut insert stitch editing reaction (CIStER)

**Fig. 3:**
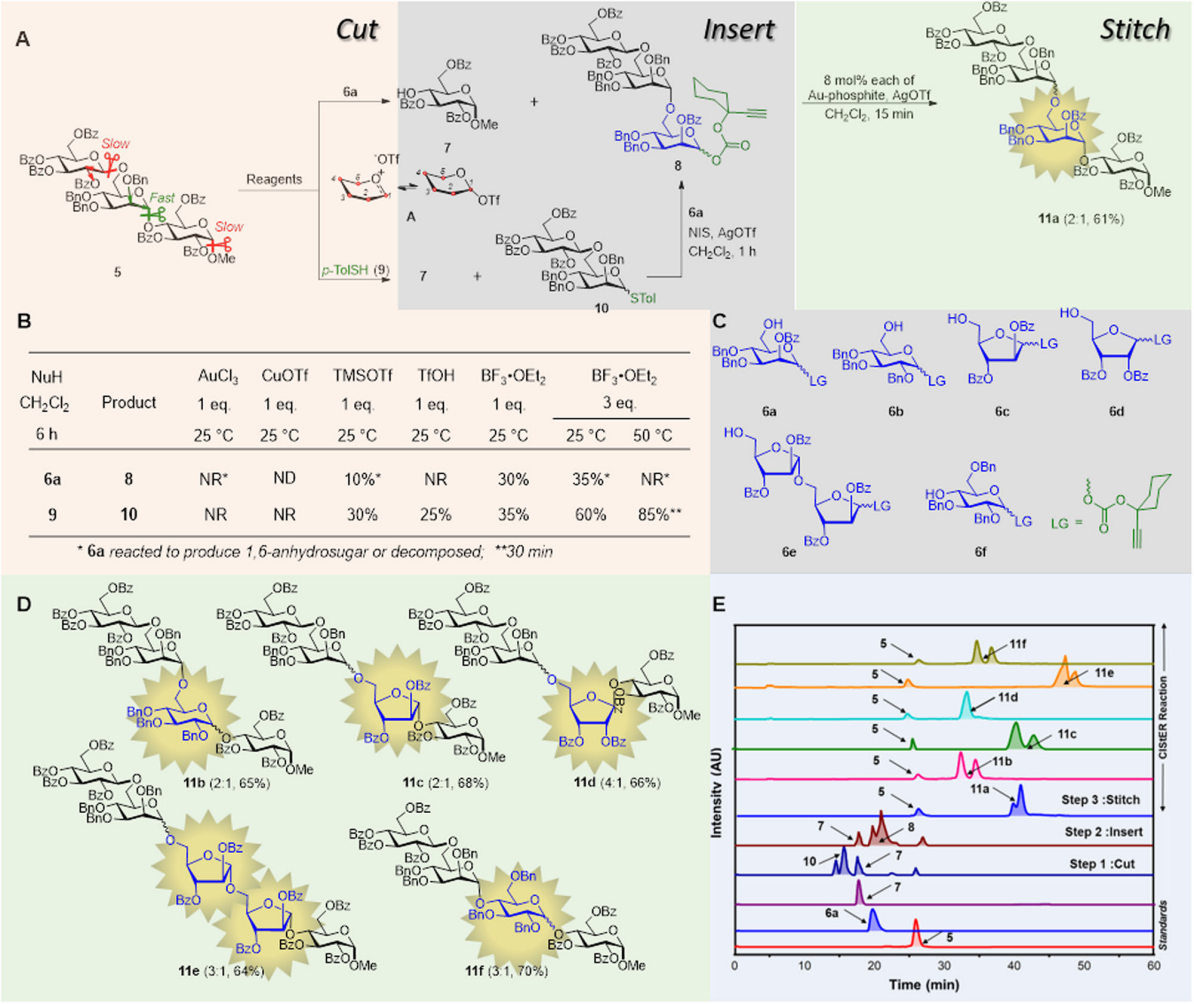
Cut insert stitch editing reaction (CIStER). **A** CIStER sequence; (**B**) Optimization of reaction conditions; (**C**) Chart ofInserting Glycans; (**D**) Products of CIStER sequence; (**E**) LC-chromatograms ofCIStER reaction. Abbreviations: Bz benzoyl, Me methyl, p-TolSH 4-methylbenzenethiol, NIS *N*-iodosuccinimide, Tf trifluoromethyl sulfonyl, NuH nucleophile, LG leaving group, AU arbitrary units, numbers in parenthesis indicate the α:β ratio and the overall yield.

The chemical fine-tuning of reactivity for site-specific cleavage is another criterion that must be satisfied for the successful implementation of CIStER. In a glycosylation reaction, the leaving group at the anomeric position (C1) extrudes upon activation to afford an active intermediate (**A**), and its stability can be fine-tuned by modifying the substituents at the C2-position. The electron-donating moieties (+*I* effect) at the C2 position stabilize the oxocarbenium ion intermediate, whereas the electron withdrawing moieties (−*I* effect) destabilize the intermediate **A**. For CIStER, glycosidic bonds are more susceptible to cleavage if the substituents on the ring impart the +*I* effect, compared to those of the saccharides that comprise −*I* imparting groups. Previous studies have suggested that the glycosyl donors equipped with C2-*O*-acyl groups require harsher conditions for glycosidation compared to those required for sugars comprising the C2-*O*-alkyl groups^19^. In other words, glycosyl donors with C2-*O*-acyl groups require harsher conditions to hydrolyze compared to those with C2-*O*-alkyl groups. Therefore, the interglycosidic bond can be cleaved regiospecifically via judicial exploitation of the benefits of the stereoelectronic effects, imparted by the protecting groups present in the glycan or using specific glycosyl hydrolases. Accordingly, trisaccharide **5** was intuitively designed to ensure three glycosidic linkages, out of which two are interglycosidic in nature, and one of the interglycosidic bonds is more susceptible to cleavage than the other because of the presence of the +*I*− directing benzyl ether at the C2-position. Previous reports indicated that the cleavage of the interglycosidic bond resulted in the formation of 1,6-anhydrosugars in the presence of 5 mol% AuCl_3_^20^. However, in this study, the treatment of compound **5** with one molar equivalent of AuCl_3_, in the presence of aglycon **6a**, produced several compounds, including the 1,6-anhydroglucose derivative^20^. An improvement was noted upon changing the reagent from AuCl_3_ to TMSOTf, and 10% of product **8** was produced along with monosaccharide **7**. By contrast, when TfOH is used, trisaccharide **8** is not obtained (Fig. 3A). The yield increases to 30% when the Lewis acid is replaced with BF_3_•Et_2_O; three equivalents of the Lewis acid marginally improve the yield of **8** to 35% at 25 °C; however, decomposition occurs when the temperature is increased to 50 °C (Fig. 3B). The synthesis of trisaccharide **8** from trisaccharide **5** was noteworthy because the former could be activated in the presence of 8 mol% each of Au-phosphite (**4a**) and AgOTf to afford tetrasaccharide **11a** (See Supplementary Figs. S38a–c, S49).

We hypothesized that a two-step process would increase the efficiency of this process; it was proposed that the oxocarbenium ion intermediate **A** from the cleavage of trisaccharide **5** could be trapped by a more nucleophilic species, and the resulting glycoside could subsequently be activated to obtain trisaccharide **8**. Accordingly, trisaccharide **5** and *p*TolSH (**9**) are treated with 1 equivalent of BF_3_•Et_2_O to afford thiotolyl gentiobiose **10** in 35% yield at 25 °C (Fig. 3B, Supplementary Data 1–2).

A 60% yield is obtained when trisaccharide **5** and *p*TolSH (**9**) are treated with 3 equivalents of BF_3_•Et_2_O and an 85% yield is obtained when the temperature is increased to 50 °C for 30 min. Gentiobiose **10** is converted to trisaccharide **8** within 1 h in a facile manner and subsequently transformed into the tetrasaccharide **11a** employing [Au]/[Ag]- catalysis (Fig. 3A, Supplementary methods S1–S3). This *cut-insert-stitch* sequence clearly demonstrated the potential of CIStER, wherein the interglycosidic bond of trisaccharide **5** was cut to insert a monosaccharide and stitched to obtain tetrasaccharide **11a**. All three independent steps (Cut-Insert-Stitch) were performed in a single pot to avoid chromatographic purification after each step (See Supplementary Fig. S1). Intermediate disaccharide **10** was glycosylated with glucosyl substrate **6b** to afford the edited tetrasaccharide **11b**. The CIStER process facilitated the insertion of furanosyl substrates such as **6c,** **6d**, and **6e** (Fig. 3C) to yield tetrasaccharides and pentasaccharide **11c,** **11d**, and **11e** respectively (Fig. 3D, E and Supplementary Fig. S1). The CIStER reaction is found to be suitable for the insertion of secondary C4-hydroxy glucoside substrates (**6** **f**) to afford **11** **f** in 70% yield.

**Fig. 4:**
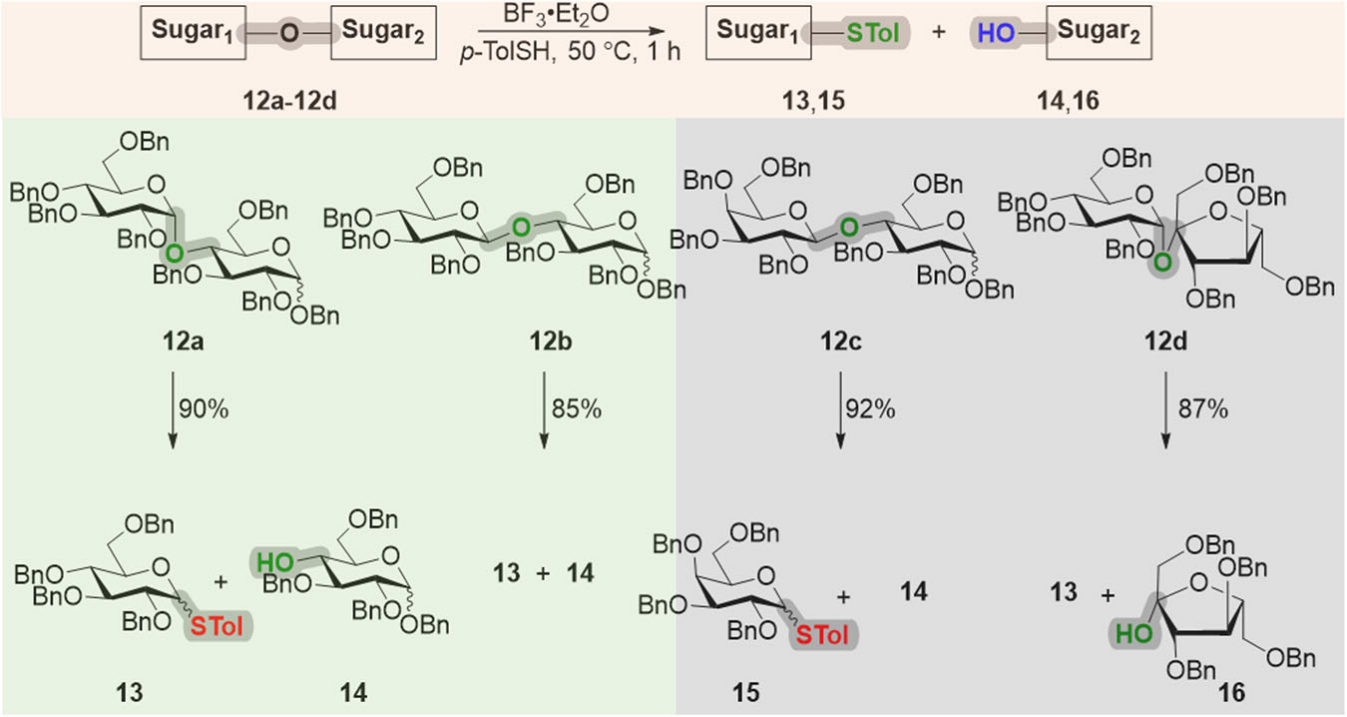
Cutting of the interglycosidic bonds in naturally occurring disaccharides. Abbreviations: Bn benzyl, p-TolSH 4-methylbenzenethiol.

In continuation, the cutting reaction was executed on four per-*O*-benzyl disaccharides (**12a**–**12d**) by treating them under the aforementioned conditions. D-Maltose derivative **12a** underwent the cleavage of the interglycosidic bond to afford the glycosyl donor **13** and the C4-OH glucoside **14** in 90% overall yield. Similarly, per-*O*-benzyl cellobiose **12b** afforded the donor **13** and the alcohol **14**. The interglycosidic bond of the lactose derivative **12c** was cleaved off in the presence of BF_3_•Et_2_O at 50 °C to give the thiotolyl galactopyranoside **15** and the alcohol **14**. Furthermore, the non-reducing sugar disaccharide **12d** had also undergone the cleavage to produce the thiotolyl glucoside **13** and the tertiary hydroxy containing fructose derivative **16**. It is important to note that the thiolyl derivatives **13** and **15** can also be activated to obtain the inserted CIStER products (Fig. 4 and Supplementary Fig. S2).

### CIStER for the synthesis of glycohybrids

In addition, we envisioned that the CIStER procedure is ideal for synthesizing unnatural glycoconjugates by choosing suitable function groups. The preceding studies proved that the interglycosidic bond can be cleaved off conveniently to install the thiotolyl moiety at the anomeric center of non-reducing sugar resulting in the formation of a thiotolyl glycosyl donor.

**Fig. 5:**
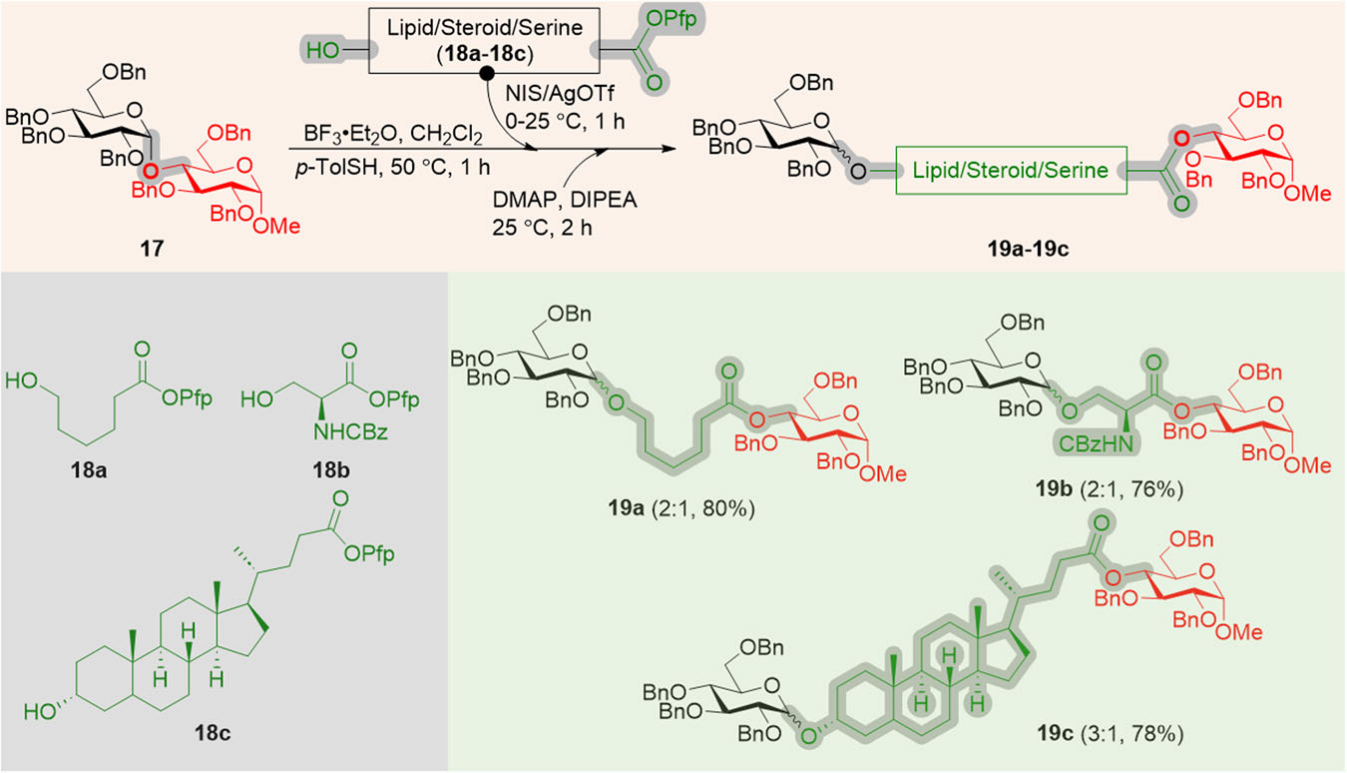
Synthesis of glycohybrids by CIStER technique. Abbreviations: Bn benzyl, p-TolSH 4-methylbenzenethiol, DMAP 4-*N,N*’-dimethylaminopyridine, Pfp pentafluorophenyl, numbers in parenthesis indicate the α:β ratio and overall yield.

Thus formed glycosyl donors can be activated in the presence of bifunctional substrates (**18a**–**18c**) to afford glycosides which can be further subjected to esterification in order to isolate the glycoconjugates. A three-step molecular surgery was performed on methyl per-*O*-benzyl maltopyranoside **17** in a single pot using inserts **18a**–**18c** to afford CIStER products **19a**–**19c**. The interglycosidic bond of the maltose derivative **17** was cut and glycosylated with the 6-hydroxycaproate (**18a**) which was subjected to the esterification under DMAP/DIPEA at 25 °C to afford an ester at the C4-position of the glucoside for stitching to produce the glycoconjugate **19a** (Supplementary methods S1–S3). Similarly, pentafluorophenyl esters of serine-derived (**19b**) and lithocholic acid (**19c**) also got inserted into the disaccharide by the CIStER technique (Fig. 5 and See Supplementary Figs. S24a–S27c).

### Gram scale synthesis by CIStER

We further examined the utility of the CIStER technique for the synthesis of human milk oligosaccharides starting from the per-*O*-acetyl lactose **20** on a gram scale. Disaccharide **20** has an acetyl moiety at the C2-position; thence, invoked the TfOH mediated cutting. Accordingly, disaccharide **20** was treated with TfOH and *p*-TolSH (**9**) in CH_2_Cl_2_ at 50 °C for 1 h, and the resulting thiotolyl galactosyl derivative was subjected to the glycosylation with the aglycone **21** in the presence of NIS/AgOTf to obtain a disaccharide with the carbonate moiety at the reducing end (Supplementary Data 1–2).

**Fig. 6:**
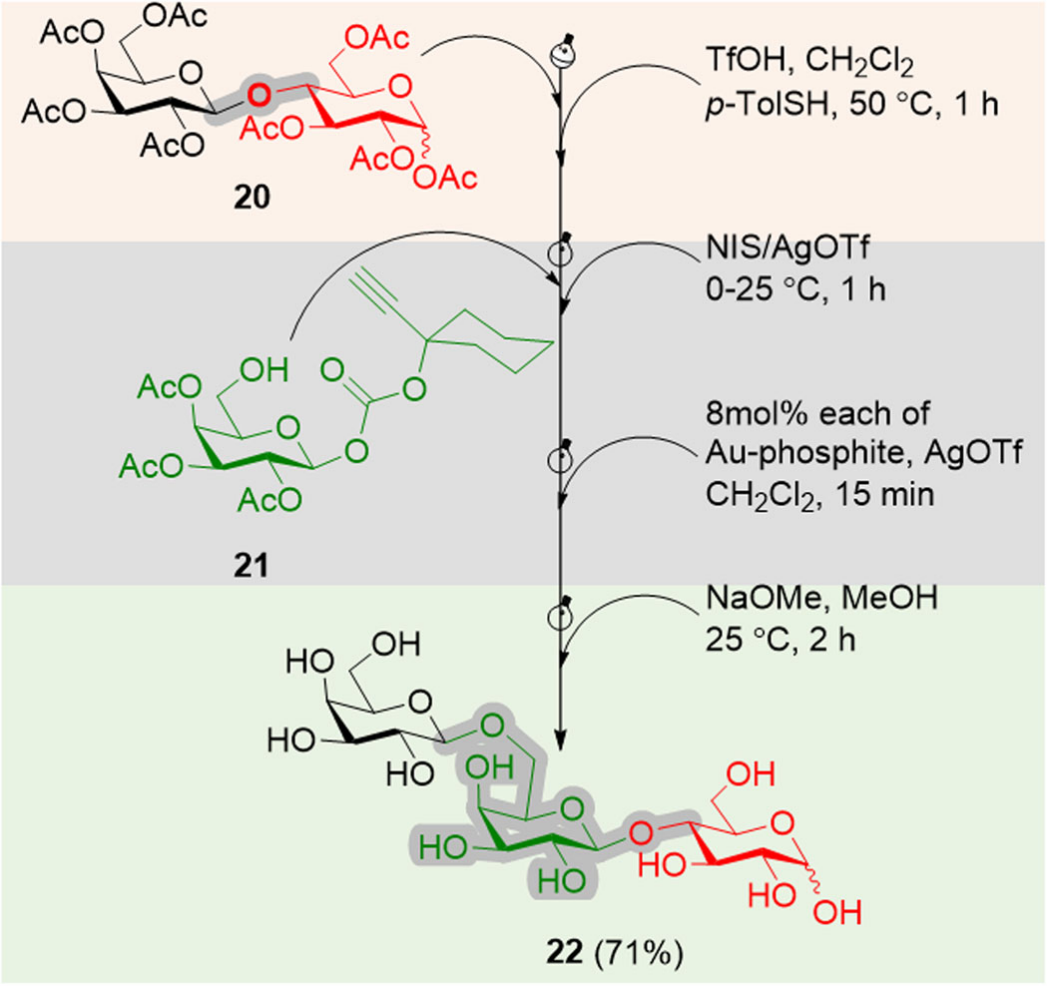
Gram scale synthesis of 6-galactosyl lactose from lactose by CIStER technique. Abbreviations: Ac acetyl, Tf trifluoromethyl sulfonyl, NIS *N*-iodosuccinimidate, Me methyl.

The carbonate donor was activated using 8 mol% each of [Au]/[Ag]-catalysts to afford a trisaccharide that upon deprotection under Zemplén conditions afforded the trisaccharide **22** in 71% overall yield. These experiments clearly illustrate the power of CIStER technique for the gram-scale synthesis of important human milk oligosaccharides (Fig. 6 and See Supplementary Figs. S46a–S46e).

### CIStER for linear and branched oligosaccharides

The CIStER technique is sufficiently robust to afford oligosaccharides, which otherwise require long reaction sequences. Furthermore, CIStER was suitable for the synthesis of lipoarabinomannan (LAM) analogs present in the *Mycobacterium tuberculosis* (Mtb). LAM is one of the most antigenic epitopes present in the cell wall of Mtb, and is currently being investigated as a target for the development of vaccines and diagnostic tools^21^. LAM comprises of an arabinan chain capped with mannopyranosyl residues in a regioselective manner. Thus, mannotrisaccharide **23** was identified as a suitable candidate for CIStER.

**Fig. 7:**
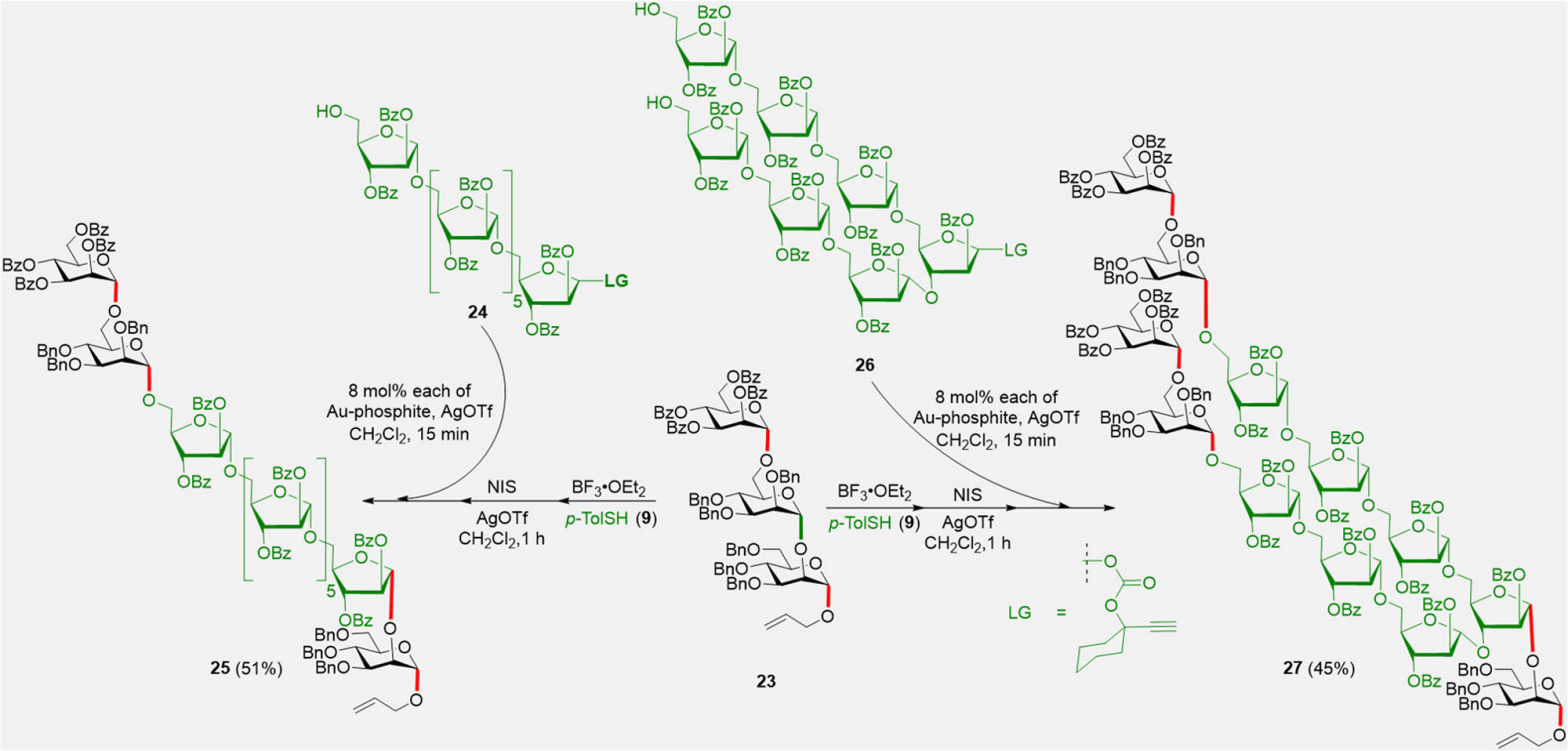
Synthesis of linear and branched oligosaccharides using CIStER. Abbreviations: Bn benzyl, Bz benzoyl, p-TolSH 4-methylbenzenethiol, LG leaving group.

The intuitively incorporated stereoelectronic groups in the trisaccharide **23** facilitated the cutting of the trisaccharide **23** to obtain a disaccharide and a monosaccharide. As depicted in Fig. 7, activation of the thiotolyldisaccharide moiety using aglycon leads to in-pot trapping of the oxocarbenium ion by heptasaccharide **24** and the subsequent stitching using [Au]/[Ag]-catalysis results in the formation of the desired arabinomannan **25**. The same disaccharide-thiotolyl intermediate was trapped with two molar equivalents of the aglycon **26** to obtain the LAM **27**, reminiscent of the cell wall of Mtb (Fig. 7 and See Supplementary Figs. S3, S43a–S43c, S44a–S44c, S56, S57).

### Conclusions

In summary, these results successfully demonstrated that the glycans can be surgically edited using CIStER. Such CIStER processes require thorough planning of the guide glycan and subtle stereoelectronic factors, leading to alterations in their interglycosidic bonds that undergo hydrolysis. CIStER is based on the activation of glycosyl donors by trapping the oxocarbenium ion intermediate with a nucleophile (*p*TolSH) that can be orthogonally activated in the presence of an aglycon equipped with another leaving group (ethynylcyclohexyl carbonate) to afford a new glycoside. Subsequently, ethynylcyclohexyl carbonate was activated to stitch the glycan and obtain a library of higher oligosaccharides in a surgical manner. Using the CIStER methodology a library of new glycohybrids could be produced that are powerful probes to unravel new biological pathways, and enhancing our current understanding thereof. Extension of CIStER methodology for small molecules is currently underway.

## Methods

### General methods

Unless otherwise noted, materials were obtained from commercial suppliers and were used without further purification. Gold-phosphite catalyst was purchased from Proactive Molecular Research, Florida (USA) and AgOTf was purchased from Sigma-Aldrich. All the moisture sensitive reactions were performed in flame dried glassware under nitrogen or argon atmosphere unless stated otherwise. 4 Å molecular sieves were activated by heating at 150–200 °C under high vacuum for 4 h before storing in a dry desiccator. Freshly distilled CH_2_Cl_2_ was stored over activated 4 Å molecular sieves (preheated to 200–250 °C). The reactions were monitored by analytical thin layer chromatography (TLC) performed on 0.25 mm Merck silica gel plates (60F_254_) under 254 nm UV lamp and stained by anisaldehyde stain. Removal of solvent in vacuo refers to distillation using a rotary evaporator attached to an efficient vacuum pump. Column chromatography of the crude compounds was performed on silica gel of 100–200 mesh (75–150 µm). Products obtained as solids or syrups were dried under high vacuum. Optical rotations were measured on digital polarimeter at 25 °C in CHCl_3_ solution. IR spectra were recorded on a FT-IR spectrometer. NMR spectra were recorded either on a 400 or 600 MHz with CDCl_3_ as the solvent and TMS as the internal standard. High resolution mass spectroscopy (HRMS) was performed using an ESI-TOF mass analyzer or MALDI-TOF mass analyzer. Normal Phase HPLC purifications were performed using Gilson’s PLC 2050 series. For NMR analysis and high-resolution mass spectrometry of the compounds in this article, see Supplementary Figs. S4–S58. Detailed experimental methods including all intermediate structures and figures (see Supplementary sections S1–S4 and Figs. S1–S3) are provided in the Supplementary Information. Synthetic procedures and Characterization Data See Supplementary Methods.

### Supplementary information

Supplementary Methods

Description of Additional Supplementary Files

Supplementary Data 1

Supplementary Data 2

## Data Availability

The authors declare that some of the data supporting the findings of this study are available in its Supplementary Information files. All data are available from the authors upon reasonable request. Supplementary Figs., Supplementary Methods and Supplementary References. NMR Spectra of all new compounds: Supplementary Data 1 MALDI-ToF spectra of selected compounds: Supplementary Data 2.
