# Supplementary material for: Cut-insert-stitch editing reaction (CIStER) sequence for surgical chemical glycan editing
Source: Commun Chem. 2024 Apr 2;7:73. doi: 10.1038/s42004-024-01152-z (PMC10987650; doi:10.1038/s42004-024-01152-z)
Supplement: Supplementary file 1 — Supplementary Methods [file 42004_2024_1152_MOESM1_ESM.pdf]

Supplementary Materials for

Cut-Insert-Stitch Editing Reaction (CIStER) Sequence for Surgical Chemical Glycan Editing

Sumit Sen, Suman Kundu, Sandip Pasari and Srinivas Hotha*

Department of Chemistry, Indian Institute of Science Education and Research Pune

Pune – 411 008, India

s.hotha@iiserpune.ac.in

Supplementary Methods

S1. Supplementary Experimental Procedures

Ⓐ**Procedure A: Deprotection of Allyl Glycosides:** To a solution of the allyl glycoside (1.0 mmol) in CH_2Cl_2/MeOH (1:4 in 10 mL), a solution of $PdCl_2$ (0.3 mmol) in MeOH (6 mL) was added and the reaction mixture was stirred at 25 °C for 5 h. After complete conversion, the reaction mixture was neutralized by the addition of excess Et_3N (~2 mL) and the solid residue was filtered off through a pad of Celite®. The volatile organics were evaporated *in vacuo* and the crude residue was purified by silica gel column chromatography to obtain desired hemiacetals.

Ⓑ**Procedure B: Deprotection of Pent-4-ene-1-ol Glycosides:** The pent-4-enyl glycoside (1.0 mmol) was dissolved in a mixture of solvents CH_2Cl_2/CH_3CN/H_2O (5.0:3.0:0.5) and the reaction mixture was cooled to -10 °C. After 15 min, NIS (2.2 mmol) and TfOH (0.2 mmol) were added to the reaction mixture simultaneously and the reaction was stirred for 1 h at 0 ° C. After completion, the reaction mixture was quenched with $NaHCO_3$ solution and diluted with CH_2Cl_2. The organic layer was successively washed with water (2x25 mL), brine solution (1x25 mL), dried over anhydrous Na_2SO_4, filtered and the filtrate was concentrated in *vacuo* to obtain a crude residue that was purified by silica gel column chromatography using ethyl acetate and hexane to furnish corresponding hemiacetals.

Ⓒ**Procedure C: Synthesis of Ethynyl Cyclohexyl Glycosyl Carbonate Donors:** To a solution of hemiacetal (1.0 mmol) in anhydrous CH_2Cl_2 (5 mL), ethynyl cyclohexyl (4-nitrophenyl) carbonate **S2** (1.2 mmol) and DMAP (1.1 mmol) were added and the reaction mixture was stirred at 25 °C for 5 h. the reaction mixture was concentrated and purified by silica gel column chromatography (EtOAc/Hexane as mobile phase) to obtain ethynylcyclohexyl glycosyl carbonate donors.

Ⓓ**Procedure D: Deprotection of the TBDPS-ethers:** To a solution of the silylated ether (1.0 mmol) in anhydrous pyridine and THF (4 mL, 3:1), HF•Py (3 mmol per TBDPS) was added at 0 °C and the reaction mixture was stirred at 25 °C for 3 h. After completion, the reaction mixture was quenched by the drop-wise addition of 2*N* HCl at 0 °C and diluted with 25 mL of ethyl acetate. The organic layer was successively washed with 1*N* HCl (25 mL), saturated aqueous solution of $NaHCO_3$ (25 mL) and brine solution (50 mL). The organic layer was dried over anhydrous Na_2SO_4 and concentrated *in vacuo*. The

crude residue was purified by silica gel column chromatography using ethyl acetate and hexane to furnish corresponding alcohol.

Ⓔ**Procedure E: Glycosidation using Ethynyl Cyclohexyl Glycosyl Carbonate Donors:**

To a solution of acceptor (1.0 mmol) and donor (1.1 mmol) in anhydrous CH_2Cl_2 (5 mL) was added freshly activated 4Å MS powder (0.200 g) at 25 °C under argon atmosphere. After 15 min of vigorous stirring at 25 °C, chloro[tris(2,4-di-*tert*butylphenyl)phosphite]gold(I) (8 mol%) and AgOTf (8 mol%) were added simultaneously to the reaction mixture and stirred for 30 min to 1 h. After consumption of the glycosyl acceptor, the reaction mixture was filtered through a bed of Celite® and the filtrate was concentrated *in vacuo*, the residue was purified by silica gel column chromatography using EtOAc and Hexane as mobile phase.

S2. Supplementary Synthesis of Substrates

S2.1.1-*O*-(((1-ethynylcyclohexyl)oxy)carbonyl)-2,3,4-tri-*O*-benzoyl-α/β-D-glucopyranoside (**2a**):

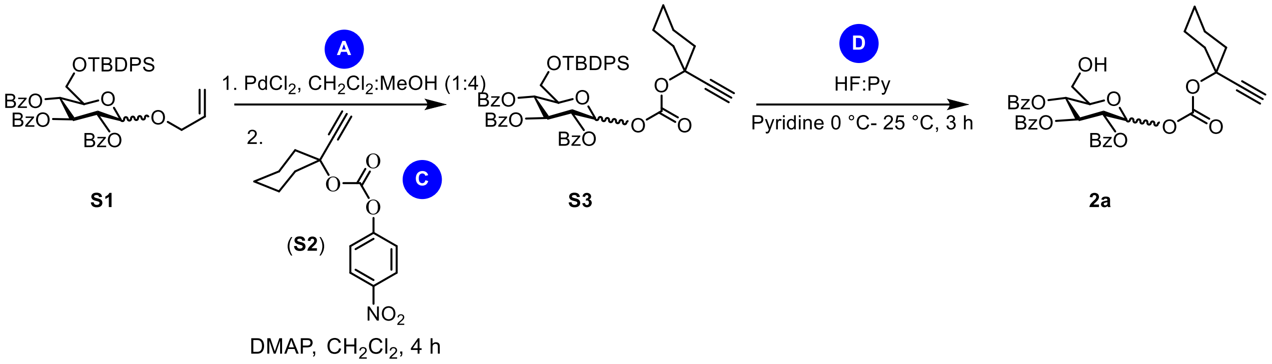

The compound **S1**[23] was converted to the compound **S3** according to general experimental procedure A,C mentioned above. After that compound **S3** was subjected to primary TBDPS deprotection according to general experimental procedure D to obtain the compound **2a** (Yield 87 %, white solid) (α :β = 1:1.3). mp**:** 101 °C; $[\alpha]_D^{25}$ = +42.048 (*c* 0.16 $CHCl_3$); ^1H NMR (400 MHz, $CDCl_3$): δ 8.06 – 7.77 (m, 6H), 7.65 – 7.09 (m, 9H), 6.55 (d, *J* = 3.7 Hz, 1H), 6.28 (t, *J* = 10.0 Hz, 1H), 6.09 – 5.90 (m, 1H), 5.71 – 5.47 (m, 2H), 4.03 –3.69 (m, 2H), 2.81 (s, 1H), 2.49 (s, 1H), 2.27 – 1.94 (m, 2H), 1.93 – 1.18 (m, 8H); ^{13}C NMR (101 MHz, $CDCl_3$): δ 166.1, 165.9, 165.8, 165.7, 165.3, 164.9, 151.0, 150.7, 133.8, 133.7, 133.4, 133.3, 130.0 (2C), 129.9 (2C), 129.9 (2C), 129.8 (2C), 129.7 (2C), 128.9 (4C), 128.7 (4C), 128.5 (2C), 128.4 (4C), 128.3 (4C), 128.3 (4C), 95.2, 92.7, 82.3, 82.2, 78.9, 78.8, 75.6, 75.4, 75.3, 72.8, 72.7, 70.9, 70.4, 70.0, 68.9, 68.7, 60.9, 60.7, 36.8, 36.7, 36.5, 36.4, 24.9, 24.7, 22.7, 22.5, 22.3, 22.3; IR ($CHCl_3$): 3444,

2919, 1453, 1027, 747, 696, 664 cm^{-1}; **HRMS** (*m/z*): $[M+Na]^+$ calcd. for $C_{36}H_{34}NaO_{11}$, 665.1999; found 665.1997.

S2.2 1-*O*-(((1-ethynylcyclohexyl)oxy)carbonyl)-2,3,4-tri-*O*-benzoyl-6-*O*-(2,3,4,6- tetra-*O*-benzoyl β-D-glucopyranosyl) - α/β D-glucopyranoside (**3a**) :

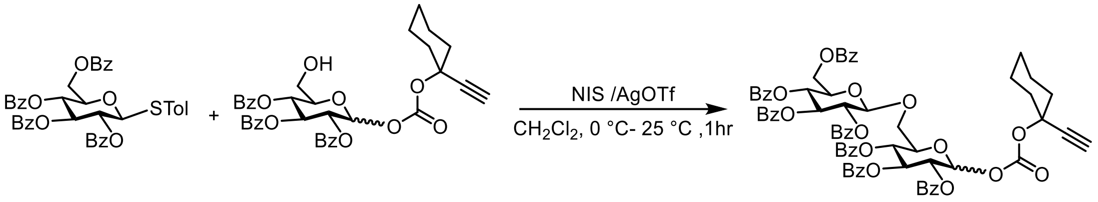

1a **2a** **3a**

The compound **1a**[24] (100 mg, 0.142 mmol) and compound **2a** (91.45 mg, 0.142 mmol) were dissolved in dry CH_2Cl_2 (3 mL), the reaction mixture was stirred at 0 °C for 10 min under nitrogen atmosphere. Freshly activated 4Å MS powder (30 mg) was added and stirred for 30 min. Following that, NIS (48 mg, 0.213 mmol) and AgOTf (9 mg, 0.035 mmol) were added and the reaction mixture was kept at 0 °C for 30 min. The reaction mixture was quenched by addition of Et_3N and filtered through Celite®. The filtrate was concentrated *in vacuo*. The residue was purified by silica gel column chromatography (30% ethyl acetate / hexane) to give the compound **3a**. (Yield 91%, white solid). (α:β = 1:1.2).

mp: 102.7 °C; $[\alpha]_D^{25}$ = +57° (*c* 0.16 $CHCl_3$); ^1H NMR (400 MHz, $CDCl_3$): δ 8.09 – 7.75 (m, 14H), 7.54 – 7.23 (m, 21H), 6.40 (d, *J* = 3.4 Hz, 1H), 6.11 (t, *J* = 9.9 Hz, 1H), 5.90 (d, *J* = 9.5 Hz, 1H), 5.85 – 5.78 (m, 1H), 5.63 (d, *J* = 9.7 Hz, 1H), 5.61 – 5.46 (m, 2H), 5.40 – 5.30 (m, 1H), 4.95 (d, *J* = 7.8 Hz, 1H), 4.63 – 4.41 (m, 2H), 4.16 – 3.99 (m, 2H), 3.87 – 3.76 (m, 1H), 2.39 (s, 1H), 2.21 – 1.98 (m, 2H), 1.94 – 1.52 (m, 8H); ^{13}C NMR (101 MHz, $CDCl_3$) δ 166.3, 166.2, 165.9, 165.9, 165.8, 165.7, 165.4, 165.3, 165.3, 165.2, 165.2, 165.1, 165.0, 164.9, 151.0, 150.7, 133.6 -133.1 (10C), 130.2-129.8 (30C), 129.7-128.9 (14C), 128.6 -128.4 (30C), 101.0, 100.5, 95.0, 92.6, 82.5, 82.4, 79.0, 78.8, 76.0, 75.7, 75.5, 73.1, 73.0, 72.8, 72.4, 72.3, 71.9, 71.8, 71.7, 70.9, 70.5, 70.2, 69.9, 69.8, 69.1, 68.8, 67.2, 66.7, 63.2 (2C), 37.1, 36.8, 36.7, 36.6, 25.0, 24.8, 22.6, 22.6, 22.5, 22.5; IR ($CHCl_3$): 3290, 2940, 1729, 1601, 1452, 1267, 1172, 1099, 1028, 906, 708 cm^{-1}; HRMS (*m/z*): $[M+Na]^+$ calcd. for $C_{70}H_{60}NaO_{20}$, 1243.3576; found, 1243.3574.

S2.3 *p*-Tolyl-2,3,4-tri-*O*-benzoyl-6-*O*-(2,3,4,6-tetra-*O*-benzoyl β-D-glucopyranosyl)-1-thio-β-D-glucopyranoside (**3b**):

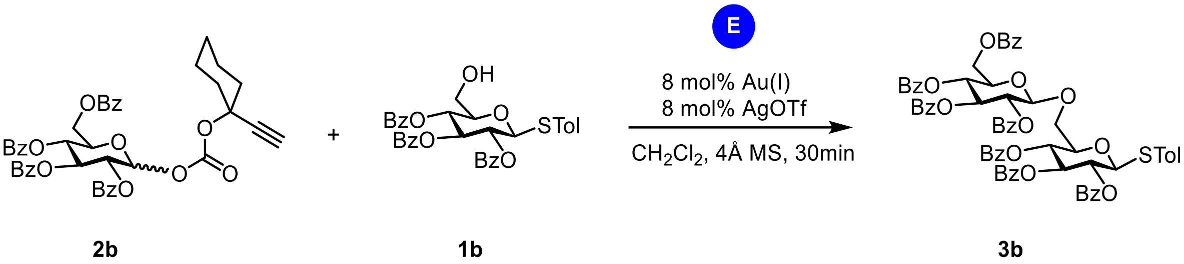

The glycosyl donor **2b**[25] and the glycosyl acceptor **1b**[26] was glycosylated according to the general reaction procedure E as detailed above to give the disaccharide **3b**. (Yield 94%, white solid).

mp: 96 °C; $[\alpha]_D^{25}$ = +78°(*c* 0.16 $CHCl_3$); ^{1}H NMR (400 MHz, $CDCl_3$): δ 8.03 – 7.78 (m, 14H), 7.55 – 7.33 (m, 23H), 7.17 (d, *J* = 7.8 Hz, 2H), 5.86 (t, *J* = 9.6 Hz, 1H), 5.77 (t, *J* = 9.5 Hz, 1H), 5.61 (t, *J* = 9.7 Hz, 1H), 5.50 (dd, *J* = 9.8, 7.8 Hz, 1H), 5.33 (t, *J* = 9.7 Hz, 1H), 5.25 (t, *J* = 9.7 Hz, 1H), 5.02 (d, *J* = 7.8 Hz, 1H), 4.81 (d, *J* = 10.0 Hz, 1H), 4.61 (dd, *J* = 12.1, 3.1 Hz, 1H), 4.41 (dd, *J* = 12.1, 5.2 Hz, 1H), 4.08– 3.92 (m, 4H), 2.36 (s, 3H); ^{13}C NMR (101 MHz, $CDCl_3$): δ 166.2, 165.9, 165.8, 165.4, 165.3, 165.3, 165.1, 139.0, 134.0, 133.6, 133.6, 133.4, 133.3, 133.3, 133.3, 130.0-29.9 (20C), 129.8, 129.7, 129.4, 129.4, , 129.0, 128.9, 128.8, 128.6-128.4 (12C), 127.8, 101.2, 86.1, 78.6, 74.3, 73.1, 72.4, 72.0, 70.7, 69.8, 69.7, 68.4, 63.1, 21.4., 21.4; IR ($CHCl_3$): 3444, 2919, 1453, 1026, 747, 696, 665 cm^{-1}; HRMS (*m/z*): $[M+Na]^+$ calcd. for $C_{68}H_{56}NaO_{17}S$, 1199.3136; found, 1199.3172.

S2.4 Allyl 2,3,4 tri-*O*-benzyl α-D-mannopyranoside (**S7**):

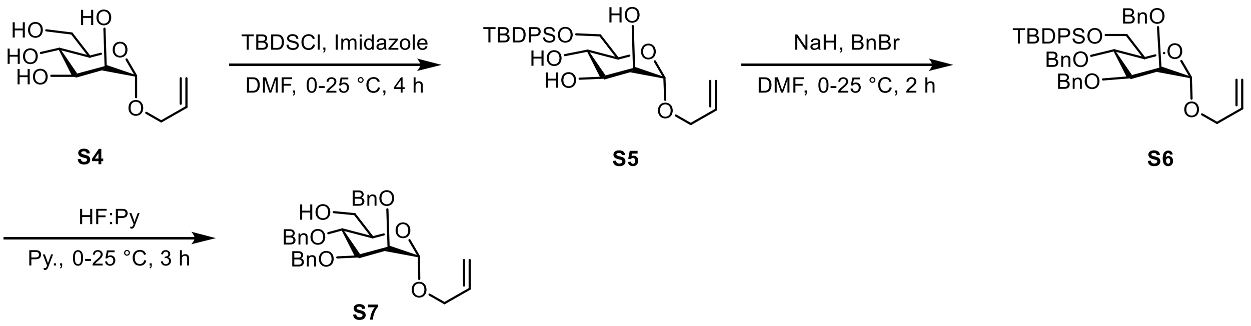

To a solution of DMF (25 mL) of the compound **S4**[27] (5.00 g, 22.91 mmol) at 0 °C imidazole (3.82 g, 45.82 mmol), TBDPSCl (7mL, 24.97 mmol) were added and resulting solution was kept 15 min at 0 °C and then heated to 25 °C. After 3 h, the reaction was arrested by the addition of ice-cold water, extracted with ethyl acetate (3x400 mL). Combined organic layers were washed with brine solution, dried over anhydrous Na_2SO_4 and concentrated *in vacuo*, the crude was the purified by silica gel column chromatography (40% ethyl acetate in hexane) to provide compound **S5** as a thick syrup (92%). Further,

compound **S5** (9.82 g, 21.08 mmol) in 150 mL of DMF was cooled to 0 °C, NaH (3.03 g, 75.84 mmol) was added portion wise over a period of 10 min, and stirred. After 15 min, benzyl bromide (11.26 mL, 94.20 mmol) was slowly added at 0 °C and stirred at 25 °C. After 2 h, the reaction mixture was poured into ice cold water, extracted with ethyl acetate (2x50 mL), the organic layer was washed with brine solution, combined organic layers were dried over anhydrous Na_2SO_4 and concentrated *in vacuo*. Resulting crude residue was purified by silica gel column chromatography (15% ethyl acetate/hexane) to give the compound **S6** as a yellow syrup (88%).

In continuation, the compound **S6** (5.0 g, 6.86 mmol) was dissolved in anhydrous pyridine (30 mL), HF•py (1.84 mL, 20.58 mmol) was added at 0 °C and the reaction mixture was stirred at 25 °C for another 3 h. Upon completion of the reaction, the reaction mixture was quenched by the addition of 1*N* HCl at 0 °C and diluted with ethyl acetate. The organic layer was successively washed with saturated $NaHCO_3$ solution and brine solution. The organic layer was dried over Na_2SO_4 and concentrated *in vacuo*. The crude product was purified with silica gel column chromatography (25% ethyl acetate/hexane) to furnish the compound **S7** as a white solid (85%).

mp: 129 °C; $[\alpha]_D^{25}$ = +62° (*c* 0.16 $CHCl_3$); ^1H NMR (400 MHz, $CDCl_3$): δ 7.38 – 7.27 (m, 15H), 5.88 – 5.77 (m, 1H), 5.24 – 5.13 (m, 2H), 4.94 (dd, *J* = 10.9, 1.7 Hz, 1H), 4.85 (d, *J* = 1.8 Hz, 1H), 4.82 – 4.59 (m, 6H), 4.17 – 4.07 (m, 1H), 4.01 – 3.94 (m, 2H), 3.94 – 3.87 (m, 1H), 3.84 – 3.76 (m, 3H), 3.66 (td, *J* = 7.0, 3.8 Hz, 1H); ^{13}C NMR (101 MHz, $CDCl_3$): δ 138.6, 138.5, 138.3, 133.7, 128.6(2C), 128.5(4C), 128.2(2C), 128.0(2C), 127.9, 127.9, 127.7, 127.7(2C), 117.5, 97.5, 80.3, 75.4, 75.0, 74.9, 73.1, 72.4, 72.3, 68.0, 62.5; IR ($CHCl_3$): 3445, 2919, 1454, 1027, 747, 696, 665 cm^{-1}; HRMS (*m/z*): $[M+Na]^+$ calcd. for $C_{30}H_{34}NaO_6$, 513.2253; found, 513.2250.

S2.5 Methyl 2,3,6 tri-*O*-benzoyl α-D-glucopyranoside (**7**):

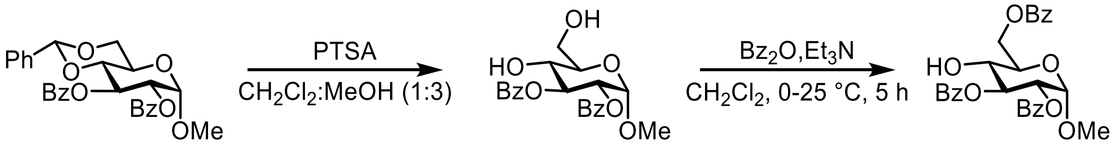

S8 **S9** **7**

To a solution of compound **S8**[28] (5.0 g, 10.19 mmol) in 80 mL of CH_2Cl_2:MeOH (1:3), *p*-toluenesulphonic acid (PTSA) (877 mg, 5.10 mmol) was added and stirred at 25 °C. After 4 h, the reaction mixture was neutralized by addition of Et_3N, extracted with CH_2Cl_2 (2x25 mL), the organic layer was washed with sodium bicarbonate and brine solution. Combined organic layers were dried over anhydrous Na_2SO_4 and concentrated *in vacuo*, the resulting crude residue was purified by silica gel

column chromatography (45% ethyl acetate/hexane) to obtain the compound **S9** as a thick syrup (85%). Further, compound **S9** (3.49 g, 8.66 mmol) was taken in 40 mL of anhydrous CH_2Cl_2 and Et_3N (6.93 mL, 49.70 mmol) and benzoic anhydride (Bz_2O) (2.70 gm, 11.93 mmol) were added simultaneously at 0 °C. The reaction mixture was brought to 25 °C and stirred for 2 h, extracted with CH_2Cl_2, the organic layer was washed with sodium bicarbonate and brine solution. Collected organic layer was dried over anhydrous Na_2SO_4, filtered, and the filtrate was concentrated to obtain a crude residue that was purified by silica gel column chromatography (20% ethyl acetate/hexane) to afford the compound **7** as a white solid (76%).

mp: 74 °C; $[\alpha]_D^{25}$ = +149° (*c* 0.18 $CHCl_3$); ^1H NMR (400 MHz, $CDCl_3$): δ 8.13 – 8.07 (m, 2H), 8.06 (dd, *J* = 8.4, 1.3 Hz, 2H), 7.98 (dd, *J* = 8.4, 2.5, 2H), 7.60 – 7.33 (m, 9H), 5.83 (dd, *J* = 10.1, 9.2 Hz, 1H), 5.30 – 5.25 (m, 1H), 5.16 (d, *J* = 3.7 Hz, 1H), 4.81 – 4.75 (m, 1H), 4.66 (dd, *J* = 12.1, 2.3 Hz, 1H), 4.13 (s, 1H), 3.92 (d, *J* = 4.6 Hz, 1H), 3.59 (d, *J* = 4.9 Hz, 1H), 3.45 (s, 3H); ^{13}C NMR (101 MHz, $CDCl_3$): δ 167.3, 167.0, 166.1, 133.5, 133.4, 133.4, 130.0(2C), 129.9(2C), 129.9(2C), 129.8, 129.3, 129.2, 128.6(2C), 128.5(2C), 128.5(2C), 97.2, 73.9, 71.6, 70.1, 69.7, 63.6, 55.5; IR ($CHCl_3$): 3455, 3024, 1719, 1452, 1270, 1094, 1068, 1026, 748, 707, 666 cm^{-1}; HRMS (*m/z*): $[M+H]^+$ calcd. $C_{28}H_{27}O_9$, 507.1655 found, 507.1658.

S2.6 Allyl-2,3,4 tri-*O*-benzyl-6-*O*-(2,3,4,6-tetra-*O*-benzoyl β-D-glucopyranosyl)-α-D-mannopyranoside (**S10**):

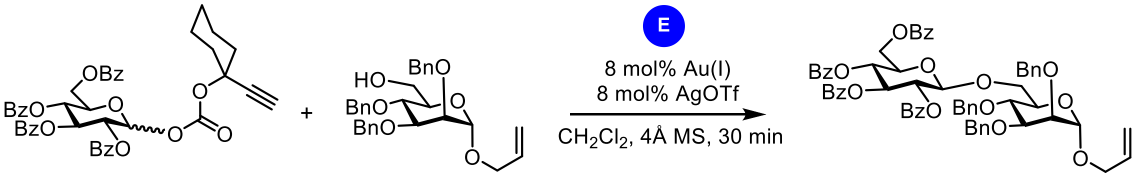

2b **S7** **S10**

The glycosyl donor **2b** and acceptor **S7** were coupled according to the above delineated general procedure E to afford compound **S10**. (94% yield, thick syrup).

$[\alpha]_D^{25}$ = +20° (*c* 0.33, $CHCl_3$); ^1H NMR (400 MHz, $CDCl_3$): δ 8.08 – 7.80 (m, 8H), 7.51 – 7.12 (m, 27H), 5.96 (t, *J* = 9.5 Hz, 1H), 5.74 (t, *J* = 9.6 Hz, 1H), 5.70 – 5.55 (m, 2H), 5.15 – 5.03 (m, 2H), 4.98 (d, *J* = 7.8 Hz, 1H), 4.74 (d, *J* = 11.0 Hz, 2H), 4.71 – 4.37 (m, 7H), 4.31 – 4.10 (m, 2H), 4.00 – 3.52 (m, 7H); ^{13}C NMR (101 MHz, $CDCl_3$): δ 166.3, 166.1, 165.4, 165.3, 138.6, 138.6, 138.5, 133.7, 133.6, 133.5, 133.4, 133.3, 130.1(2C), 130.0(4C), 130.0(4C), 129.9(4C), 129.8, 129.6, 129.0, 129.0, 128.6(2C),

128.6(4C), 128.5(2C),128.1(2C), 128.0(2C), 127.9, 127.8(4C), 117.2, 101.9, 97.0, 80.4, 77.7, 75.2, 75.2, 74.7, 73.3, 72.8, 72.3, 72.2, 71.6, 70.1, 69.8, 67.5, 63.4; IR ($CHCl_3$): 3005, 1725, 1648, 1452, 1263, 1091, 1068, 1026, 746, 707, 666 cm^{-1}; HRMS (*m/z*): $[M+H]^+$ calcd. for $C_{64}H_{61}O_{15}$, 1069.4010; found, 1069.4015.

S2.7 1-*O*-(((1-ethynylcyclohexyl)oxy)carbonyl)-2,3,4-tri-*O*-benzyl-6-*O*-(2,3,4,6-tetra-*O*-benzoyl β-D-glucopyranosyl)-α/β-D-mannopyranoside (**S11**) :

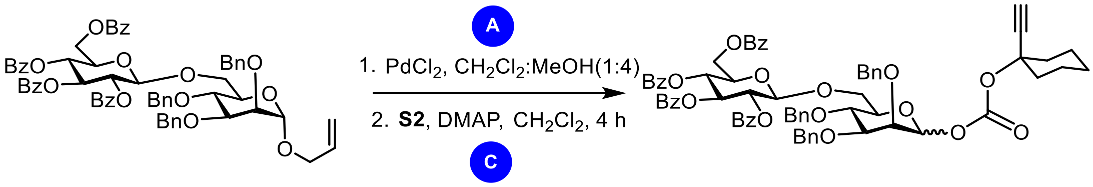

S10 **S11**

The compound **S10** was converted to hemiacetal according to general experimental procedure A, thus resulting hemiacetals were transformed to the compound **S11** according to the general procedure C. (84% yield over two steps, viscous syrup) (α: β =1 :1.05).

$[\alpha]_D^{25}$ = +9° (*c* 0.16, $CHCl_3$); ^{1}H NMR (400 MHz, $CDCl_3$): δ 8.05 – 7.82 (m, 8H), 7.54 – 7.23 (m, 27H), 6.04 (d, *J* = 2.1 Hz, 1H), 5.93 – 5.78 (m, 1H), 5.73 – 5.52 (m, 2H), 4.95 (d, *J* = 7.9 Hz, 1H), 4.79 – 4.36 (m, 8H), 4.20 – 4.08 (m, 2H), 3.97 – 3.70 (m, 5H), 2.80 (s, 1H), 2.32 – 2.08 (m, 2H), 2.00 – 1.53 (m, 8H); ^{13}C NMR (101 MHz, $CDCl_3$) : δ 166.3, 166.2, 165.9, 165.9, 165.3, 165.2, 165.2, 165.1, 151.3, 150.8, 138.3, 138.3, 138.2, 137.9, 137.9, 137.9, 133.4- 133.0 (10C), 130.2 - 129.8 (20C) , 129.7, 129.7, 129.5 (2C), 129.4, 129.0, 128.9 (2C), 128.5- 128.3 (20C), 128.0-127.6 (20C), 101.1, 100.9, 95.6, 94.9, 82.8, 82.7 (2C), 81.4, 79.1(2C), 78.4, 78.3, 77.3, 75.7, 75.5, 75.0, 74.8, 74.1, 74.1, 73.9, 73.4, 73.3 (2C), 73.2, 72.5 (2C), 72.0, 72.0, 71.9, 71.8, 69.9, 69.9, 68.2, 67.8, 63.3 (2C), 36.9, 36.8, 36.7, 36.6, 25.0, 25.0, 25.0, 22.8, 22.7, 22.6, 22.6; IR ($CHCl_3$): 3026, 2956, 1731, 1452, 1265, 1091, 1026.53, 902, 746, 707, 666 cm^{-1}; HRMS (*m/z*): $[M+H]^+$ calcd. for $C_{70}H_{67}O_{17}$, 1179.4378; found, 1179.4369.

S2.8 Methyl-2,3,6-tri-*O*-benzoyl-4-*O*-(2,3,4-tri-*O*-benzyl-6-*O*-(2,3,4,6-tetra-*O*-benzoyl β D-glucopyranosyl) α-D-mannopyranosyl) α-D-glucopyranoside (**5**):

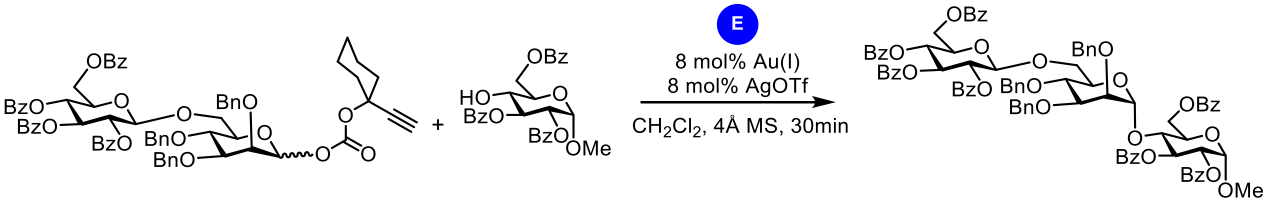

S11 **7** **5**

The compounds **S11** and **7** were coupled according to the general glycosylation procedure E to obtain the compound **5** (Yield 92%, white solid).

mp: 104 °C; $[\alpha]_D^{25}$ = +45° (*c* 0.16 $CHCl_3$); ^{1}H NMR (400 MHz, $CDCl_3$): δ 8.11 (d, *J* = 7.8 Hz, 2H), 8.01 – 7.71 (m, 12H) ,7.52 – 7.14 (m, 36H), 5.99 (t, *J* = 9.5 Hz, 1H), 5.88 (t, *J* = 9.6 Hz, 1H), 5.63 (q, *J* = 9.8 Hz, 2H), 5.12 (d, *J* = 3.4 Hz, 1H), 5.09 (d, *J* = 3.2 Hz, 1H), 5.07 (d, *J* = 9.6 Hz, 1H), 4.80 (d, *J* = 7.7 Hz, 1H), 4.70 (d, *J* = 11.1 Hz, 1H), 4.59 – 4.34 (m, 6H), 4.23 (dd, *J* = 16.3, 10.8 Hz, 2H), 4.12 – 3.97 (m, 3H), 3.97 – 3.85 (m, 3H), 3.75 (q, *J* = 10.0, 9.5 Hz, 3H), 3.56 (s, 1H), 3.40 (s, 3H); ^{13}C NMR (101 MHz, $CDCl_3$): δ 166.3(2C), 166.0(2C), 165.6, 165.3, 138.7, 138.5, 138.3, 133.7(2C), 133.4(4C), 133.3(2C), 133.2(2C), 133.1, 133.1, 130.0, 130.0, 129.9, 129.9, 129.9, 129.7, 129.6, 129.3, 129.2, 129.0, 128.8(3C), 128.5, 128.4,(4C) 128.4(6C), 128.3(4C), 128.1(4C), 127.8(4C), 127.6, 127.4(4C), 127.2(4C), 101.8, 100.1, 96.7, 79.6, 76.1, 75.9, 74.7, 74.1, 73.2, 73.1, 72.6, 72.4, 72.2, 72.1, 71.9, 71.7, 70.0, 69.3, 68.5, 63.9, 63.5, 55.5; IR ($CHCl_3$): 2946, 1722, 1452, 1264, 1092, 1068, 1026, 749, 707 cm^{-1}; HRMS (*m/z*): $[M+H]^+$ calcd. for $C_{89}H_{81}O_{23}$, 1517.5169; found, 1517.5168.

S2.9 1-*O*-(((1-ethynylcyclohexyl)oxy)carbonyl) 2,3-di-*O*-benzoyl α/β-D-arabinofuranoside (**6c**):

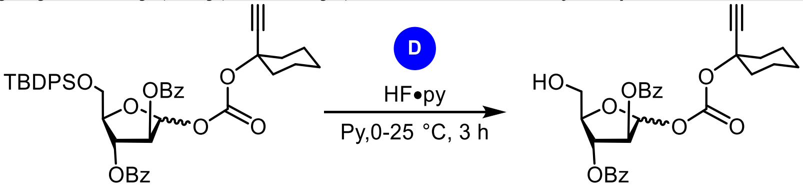

S12 **6c**

Compound **S12**[29] was converted to compound **6c** according to the general experimental procedure D (Yield 92%, white solid) (α:β =3.7:1).

mp: 59 °C; $[\alpha]_D^{25}$ = -41° (*c* 0.16, $CHCl_3$); ^{1}H NMR (400 MHz, $CDCl_3$): δ 8.12 – 8.01 (m, 4H), 7.63 – 7.41 (m, 6H), 6.33 (s, 1H), 5.89 – 5.77 (m, 1H), 5.72 (d, *J* = 1.6 Hz, 1H), 5.54 (dd, *J* = 4.6, 1.6 Hz, 1H), 4.52 (q, *J* = 4.1 Hz, 1H), 4.06 – 3.95 (m, 2H), 2.69 (s, 1H), 2.20 (dq, *J* = 13.1, 6.6, 5.5 Hz, 2H), 1.93 (m, 8H); ^{13}C NMR (101 MHz, $CDCl_3$): δ 166.3, 165.9, 165.4, 165.1, 151.0, 150.8, 133.8(2C), 133.7(2C), 133.7(2C), 130.0(2C), 130.0(2C), 129.9(2C), 129.0, 128.8, 128.7, 128.7, 128.6, 128.6, 128.5(4C), 128.4(2C), 102.1, 96.7, 85.9, 83.3, 82.5, 81.0, 78.5, 78.4, 77.4, 77.0, 76.2, 75.5, 75.2, 75.1, 63.6, 61.9, 36.8, 36.8, 36.7, 36.5, 24.9, 24.8, 22.7, 22.5, 22.4, 22.3; IR($CHCl_3$): 3440, 2939, 1725, 1240, 1109, 1026, 902, 746, 708, 666 cm^{-1}; HRMS (*m/z*): $[M+Na]^+$ calcd. for $C_{28}H_{28}NaO_9$, 531.1631; found 531.1637.

S2.10 1-*O*-(((1-ethynylcyclohexyl)oxy)carbonyl) 2-*O*-benzoyl, 3,4-di-*O*-benzyl α-D-mannopyranoside (**6a**):

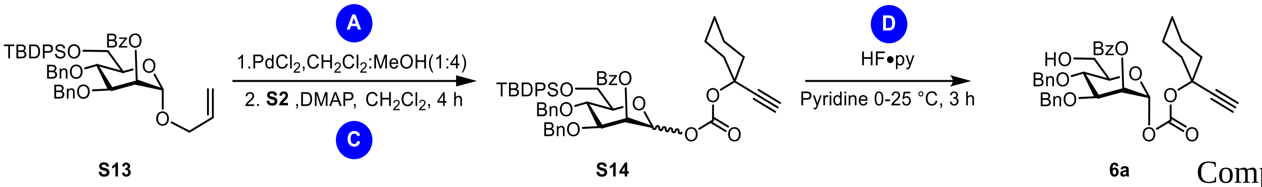

Compound **S13**[30] was converted to carbonate donor **S14** according to general experimental procedure A, C. Next, the compound **S14** was subjected to the experimental procedure D to obtain the compound **6a** (yield 88% ,white solid).

mp: 60 °C; $[\alpha]_D^{25}$ = + 3° (*c* 0.16, $CHCl_3$); ^1H NMR (400 MHz, $CDCl_3$): δ 8.13 – 8.04 (m, 2H), 7.64 – 7.45 (m, 3H), 7.36 – 7.24 (m, 10H), 6.08 (d, *J* = 2.1 Hz, 1H), 5.70 (dd, *J* = 3.2, 2.1 Hz, 1H), 4.95 – 4.57 (m, 4H), 4.20 – 3.76 (m, 6H), 2.65 (s, 1H), 2.18 (d, *J* = 8.1 Hz, 2H), 2.07 – 1.58 (m, 8H); ^{13}C NMR (101 MHz, $CDCl_3$): δ 165.5, 150.5, 138.1, 137.8, 133.6, 130.1(2C), 129.6, 128.7(2C), 128.5(2C), 128.5(2C), 128.2(2C), 128.1(2C), 128.0, 127.8, 94.3, 82.5, 78.8, 77.9, 75.5, 75.5, 74.2, 73.4, 71.9, 67.9, 61.8, 37.0, 36.7, 25.0, 22.8, 22.6; IR ($CHCl_3$): 3440, 2941, 1760, 1724, 1453, 1264, 1239, 1095, 1011, 902, 745, 709, 666 cm^{-1}; HRMS (*m/z*): $[M+Na]^+$ calcd. for $C_{36}H_{38}NaO_9$, 637.2414; found, 637.2413.

S2.11 1-*O*-(((1-ethynylcyclohexyl)oxy)carbonyl)-2,3,4-tri-*O*-benzyl-α/β-D-glucopyranoside (**6b**):

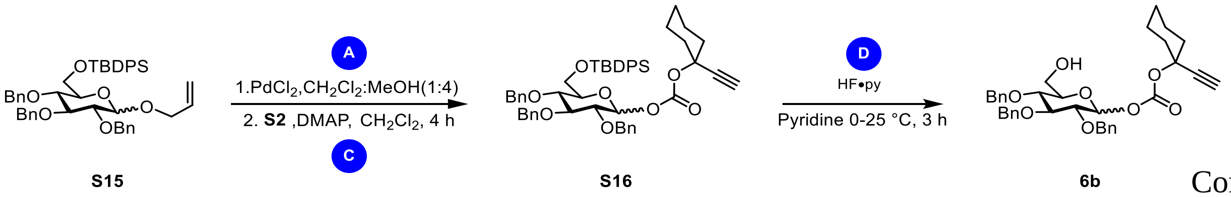

Compound **S15**[31] was converted to carbonate donor **S16** according to general experimental procedure A,C. Next, the compound **S16** was subjected to the experimental procedure D to obtain the compound **6b** (yield 92%, thick syrup)(α:β=2.5:1).

$[\alpha]_D^{25}$ = + 44° (*c* 0.23, $CHCl_3$); ^1H NMR (400 MHz, $CDCl_3$): δ 7.37 – 7.29 (m, 15H), 6.14 (d, *J* = 3.5 Hz, 1H), 4.99 – 4.68 (m, 6H), 4.02 (t, *J* = 9.3 Hz, 1H), 3.90 – 3.84 (m, 1H), 3.84 – 3.62 (m, 4H), 3.59 – 3.45 (m, 1H), 2.62 (d, *J* = 9.1 Hz, 1H), 2.23 – 2.09 (m, 2H), 1.95 – 1.58 (m, 8H); ^{13}C NMR (101 MHz, $CDCl_3$): δ 151.5, 151.2, 138.6, 138.3, 138.0, 137.9, 137.9, 137.7, 128.6, 128.5, 128.5(4C), 128.4(4C), 128.1(4C), 128.1, 128.1(4C), 128.0(4C), 127.9(4C), 127.9, 127.8, 127.7, 97.4, 93.4, 84.4, 82.8, 82.5, 81.4, 81.0, 79.0, 78.6, 78.1, 76.9, 76.6, 76.1, 76.1, 75.8, 75.8, 75.4, 75.2, 75.2, 75.1, 73.5, 73.3, 61.5, 61.4, 36.8, 36.8, 36.8, 36.7, 25.0, 24.9, 22.6, 22.6, 22.5, 22.4; IR ($CHCl_3$): 3447, 2938, 1755, 1454,

1268, 1239, 1070, 1006, 895, 848, 749, 695 cm^{-1}; HRMS (*m/z*): $[M+Na]^+$ calcd. for $C_{36}H_{40}NaO_8$, 600.2621; found, 600.2610.

S2.12 1-*O*-(((1-ethynylcyclohexyl)oxy)carbonyl)-2,3-di-*O*-benzoyl β-D-ribofuranoside (**6d**):

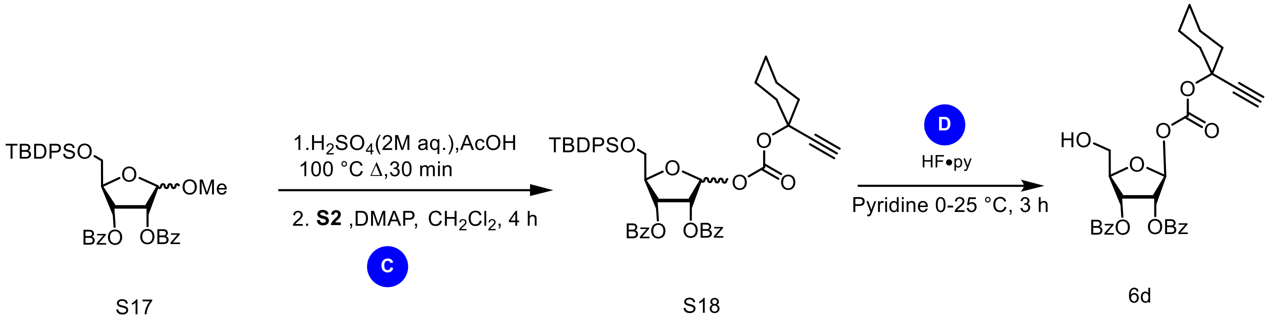

To a solution of compound **S17** (4gm, 6.55 mmol) in glacial acetic acid (80 mL) and aqueous H_2SO_4 (2M, 40 mL) was heated to 100 °C for 30 min. After completion of starting material the reaction mixture was diluted with cold water (70 ml) and ethyl acetate (70ml). The organic layer was washed several times with cold water to remove AcOH and then with ice cold saturated $NaHCO_3$. The combined organic layer was concentrated under vacuum to get a syrup. This syrup was purified using silica gel column chromatography (20% ethyl acetate/ hexane) to obtain the hemiacetal., which was transformed to the glycosyl donor **S18** according to general experimental C. (82% yield over two steps, thick syrup). Finally, the TBDPS group was deprotected using general experimental procedure D to obtain compound **6c** (78%, viscous syrup).

$[\alpha]_D^{25}$ = -11° (*c* 0.16, $CHCl_3$); ^1H NMR (400 MHz, $CDCl_3$): δ 8.06 – 7.99 (m, 2H), 7.91 – 7.83 (m, 2H), 7.61 – 7.28 (m, 6H), 6.31 (d, *J* = 8.4 Hz, 1H), 5.81 – 5.76 (m, 2H), 4.54 (q, *J* = 5.8, 4.7, 3.7 Hz, 1H), 4.02 – 3.80 (m, 2H), 2.68 (s, 1H), 2.36 (s, 1H), 2.27 – 2.10 (m, 2H), 1.97 – 1.53 (m, 8H).^{13}C NMR (101 MHz, $CDCl_3$): δ 165.6, 165.0, 150.8, 133.7, 133.5, 129.9, 129.8, 128.9(2C), 128.8(2C), 128.6(2C), 128.4(2C), 101.3, 83.7, 82.4, 78.8, 75.5, 75.3, 71.0, 62.6, 36.8, 36.7, 24.9,22.7, 22.6; IR ($CHCl_3$): 3410, 2940, 2860, 1729, 1602, 1452, 1264, 1238, 1178, 1107, 1010, 902, 748, 706, 660 cm^{-1}; HRMS (*m/z*): $[M+Na]^+$ calcd. for $C_{28}H_{28}NaO_9$, 531.1631; found, 531.1636.

S2.13 1-*O*-(((1-ethynylcyclohexyl)oxy)carbonyl)-2,3 di -*O*-benzoyl -5- *O*-(2,3,-di- *O*-benzoyl – α-D-arabinofuranosyl)- α/β-D-arabinofuranoside (**6e**) :

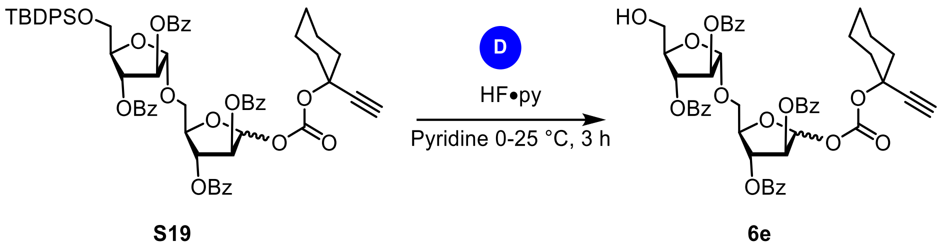

The compound **S19**[29] was converted to compound 6e according to general reaction procedure D. (Yield 88 %, white foam). (α:β=4.5:1).

$[\alpha]_D^{25}$ = -15° (*c* 0.16 $CHCl_3$); ^{1}H NMR (400 MHz, $CDCl_3$): δ 8.13 – 7.89 (m, 8H), 7.61 – 7.28 (m, 12H), 6.36 (d, *J* = 4.5 Hz, 1H), 5.74 (d, *J* = 1.4 Hz, 1H), 5.72 (d, *J* = 4.6 Hz, 1H), 5.59 (d, *J* = 4.9 Hz, 1H), 5.48 – 5.43 (m, 1H), 5.40 (s, 1H), 4.66 (d, *J* = 4.0 Hz, 1H), 4.46 (m, 1H), 4.21 (dd, *J* = 11.3, 4.3 Hz, 1H), 3.98 (m, 3H), 2.68 (s, 1H), 2.22 (s, 1H), 2.19 – 1.92 (m, 2H), 1.91 – 1.22 (m, 8H); ^{13}C NMR (101 MHz, $CDCl_3$): δ 166.2, 166.0, 165.7, 165.6, 165.5, 165.2, 165.2, 165.1, 151.1, 151.0, 133.7(2C), 133.7(2C), 133.6(2C), 133.4(2C), 130.1, 130.1, 130.0, 129.9(4C), 129.9(4C), 129.8, 129.8(4C), 129.2, 129.1, 129.1, 129.1, 129.0, 128.9, 128.8, 128.8, 128.7(4C), 128.6(4C), 128.5(4C), 128.4(4C), 106.0, 105.6, 102.3, 96.6, 84.3, 84.0, 83.9, 82.6, 82.0, 81.6, 81.0, 80.7, 78.4, 78.4, 77.7, 77.4, 77.6, 76.8, 76.1, 75.5, 75.2, 74.5, 67.3, 65.9, 62.3, 62.2, 36.8, 36.7, 36.6, 36.6, 24.9, 24.8, 22.7, 22.6, 22.4, 22.4; IR ($CHCl_3$): 3316, 2934, 1722, 1452, 1264, 1109, 1070, 1026, 709 cm^{-1}; HRMS (*m/z*): $[M+Na]^+$ calcd. for $C_{47}H_{44}O_{11}Na$, 872.2611; found, 872.2618.

S2.14 1-*O*-(((1-ethynylcyclohexyl)oxy)carbonyl)- 2,3,6 -tri-*O*-benzyl-α-D-glucopyranoside (**6f**)

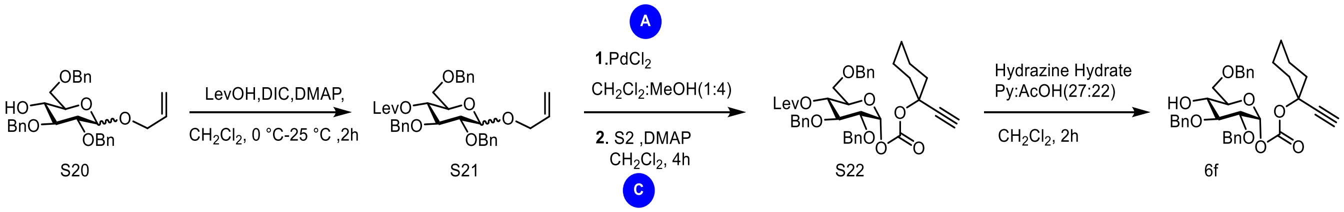

To a solution of compound S20[32] (**1**mmol, 1gm) in anhydrous CH_2Cl_2 (10ml), DMAP (1mmol, 250mg), Levulinic acid (1.5 mmol, 356mg), N, N'-Diisopropylcarbodiimide (1.5 mmol, 474μL) were added at 0 °C under nitrogen atmosphere and the reaction was allowed to stir for another 2 hr. After complete conversion of starting alcohol, the reaction mixture was concentrated in *vaccu* and the crude reaction mixture was purified by silica gel column chromatography with ethyl acetate and hexane as eluent (25-30 % e.a/ hexane) to afford the compound S21. After this the compound was converted to the compound S22 according the general reaction procedure A, C. Finally, the compound S22 (1mmol, 1.5 gm) was

dissolved in 10 ml of anhydrous CH_2Cl_2. To this solution a buffer solution of py/AcOH (27:22 mol) and 80% hydrazine hydrate (6mmol, 651 µL) were added and the reaction mixture was stirred for 2hr.After completion, the reaction mixture was quenched by addition of acetone (2ml) and the reaction mixture was extracted with ethyl acetate. The organic layer was further washed by 1(N) HCl, $NaHCO_3$ (2X 50 ml), brine (3x25 ml), dried over Na_2SO_4 and concentrated in *vaccu*. The crude reaction mixture was purified in silica gel column chromatography (15-20 e.a/hexane) to afford the compound the 6f. (88 % yield, thick syrup)

$[\alpha]_D^{25}$ = + 70º (*c* 0.25, $CHCl_3$); ^{1}H NMR (400 MHz, $CDCl_3$): δ 7.34 – 7.27 (m, 15H), 5.52 (d, *J* = 7.8 Hz, 1H), 4.93 – 4.68 (m, 4H), 4.62 – 4.43 (m, 2H), 3.78 – 3.66 (m, 3H), 3.59 – 3.49 (m, 3H), 2.76 (s, 1H), 2.60 (s, 1H), 2.24 – 2.09 (m, 2H), 1.98 – 1.56 (m, 8H); ^{13}C NMR (101 MHz, $CDCl_3$): δ 151.3, 138.5, 138.0, 137.8, 128.5-127.8 (15C), 97.5, 83.9, 82.6, 80.5, 78.4, 75.4, 75.3, 75.0, 75.0, 73.6, 71.2, 69.5, 36.8, 36.7, 24.9, 24.7, 22.5; IR ($CHCl_3$): 3446, 2937, 1755, 1453, 1268, 1239, 1069, 1005, 895, 847, 749, 695 cm^{-1}; HRMS (*m/z*): $[M+Na]^+$ calcd. for $C_{36}H_{40}NaO_8$, 623.2621; found, 623.2610.

S2.15 Allyl-2,3,4-tri-*O*-benzyl-6-*O*-(2,3,4,6-tetra-*O*-benzoyl- α- D-mannopyranosyl)- α- D-mannopyranoside (**S24**):

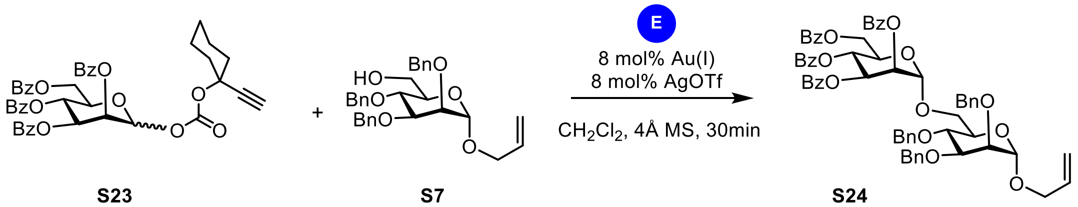

The mannosyl donor **S23**[25] and acceptor **S7** was coupled according to general glycosylation procedure E to obtain compound **S24** (viscous Syrup, 88% yield)

$[\alpha]_D^{25}$ = + 3º (*c* 0.26 $CHCl_3$); ^{1}H NMR (400 MHz, $CDCl_3$): δ 8.17 – 8.02 (m, 4H), 7.95 – 7.79 (m, 4H), 7.60 – 7.22 (m, 27H), 6.14 (t, *J* = 10.0, 3.0 Hz, 1H), 5.99 (d, *J* = 3.3 Hz, 1H), 5.95 – 5.86 (m, 1H), 5.79 (q, *J* = 2.6 Hz, 1H), 5.32 (d, *J* = 17.3 Hz, 1H), 5.24 – 5.22 (m, 1H), 5.21 (d, *J* = 10.7 Hz, 1H), 5.04 (dd, *J* = 11.3, 2.5 Hz, 1H), 4.92 (d, *J* = 2.4 Hz, 1H), 4.81 – 4.60 (m, 6H), 4.56 – 4.50 (m, 1H), 4.46 (dd, *J* = 12.1, 3.6 Hz, 1H), 4.27 (dd, *J* = 13.1, 4.8 Hz, 1H), 4.08 – 3.95 (m, 4H), 3.94 – 3.84 (m, 3H); ^{13}C NMR (101 MHz, $CDCl_3$): δ 166.3, 165.6, 165.4, 165.3, 138.5, 138.5, 138.4, 133.9, 133.5, 133.2, 133.1, 130.1, 130.0(2C), 129.9(2C), 129.9(2C), 129.8(2C), 129.6, 129.3, 129.2, 128.7, 128.6(2C), 128.5(2C), 128.5, 128.4, 128.0(4C), 128.0(4C), 127.8(4C), 127.8(4C), 117.6, 97.8, 97.2, 80.5, 75.2, 74.9, 72.9, 72.2, 71.6,

70.6, 70.6, 70.2, 68.9, 68.1, 67.3, 67.1, 62.9; IR ($CHCl_3$): 2980, 1727, 1452, 1263, 1094, 1027, 747, 709, 667 cm^{-1}; HRMS (*m/z*): $[M]^+$ calcd. for $C_{64}H_{60}O_{15}$, 1068.3932; found, 1068.3940.

S2.16 1-*O*-(((1-ethynylcyclohexyl)oxy)carbonyl)-2,3,4-tri-*O*-benzyl-6-*O*-(2,3,4,6- tetra-*O*-benzoyl α - D-mannopyranosyl) – α- D-mannopyranoside (**S26**) :

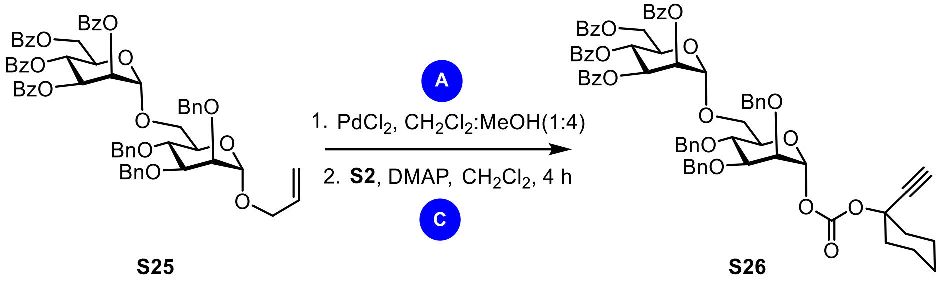

The compound **S25** was converted into glycosyl carbonate donor **S26** according to general experimental procedure A, C. (Yield 90% over two steps, viscous syrup).

$[\alpha]_D^{25}$ = + 4° (*c* 0.26, $CHCl_3$); ^{1}H NMR (400 MHz, $CDCl_3$) : δ 8.15 – 8.09 (m, 2H), 8.06 – 7.99 (m, 4H), 7.86 – 7.78 (m, 2H), 7.51 – 7.24 (m, 27H), 6.14 (t, *J* = 10.0 Hz, 1H), 6.07 (d, *J* = 2.0 Hz, 1H), 5.88 – 5.75 (m, 2H), 5.14 (d, *J* = 1.3 Hz, 1H), 5.04 (d, *J* = 11.2 Hz, 1H), 4.85 – 4.52 (m, 7H), 4.50 – 4.42 (m, 1H), 4.06 – 3.89 (m, 6H), 2.70 (s, 1H), 2.21 (m, 2H), 1.94 – 1.49 (m, 8H); ^{13}C NMR (101 MHz, $CDCl_3$) δ 166.2, 165.6, 165.2, 165.2, 150.8, 138.2, 138.2, 137.8, 133.3, 133.3, 133.1, 132.9, 130.1, 130.0(2C), 129.9(2C), 129.9(2C), 129.8(2C), 129.7, 129.5, 129.2, 129.2, 128.6(2C), 128.5(2C), 128.5-128.3(12C), 128.3, 128.0, 127.9, 127.8, 127.7, 127.7, 97.9, 94.4, 82.7, 79.5, 78.4, 75.4, 75.2, 74.1, 73.6, 73.2, 72.6, 72.2, 70.4, 70.3, 68.6, 67.3, 66.7, 62.7, 37.2, 36.3, 24.9, 22.7, 22.6; IR ($CHCl_3$): 2990, 1728, 1265, 1216, 1109, 1027, 903, 743, 709, 667 cm^{-1}; HRMS (*m/z*): $[M]^+$ calcd. for $C_{70}H_{67}O_{17}$, 1179.4378; found, 1179.4367.

S2.17 Allyl-3,4,6-tri-*O*-benzyl-2-*O*-(2,3,4-tri-*O*-benzyl-6-*O*-(2,3,4,6-tetra-*O*-benzoyl-β-D-mannopyranosyl) α-D-mannopyranosyl) α-D-mannopyranoside (23):

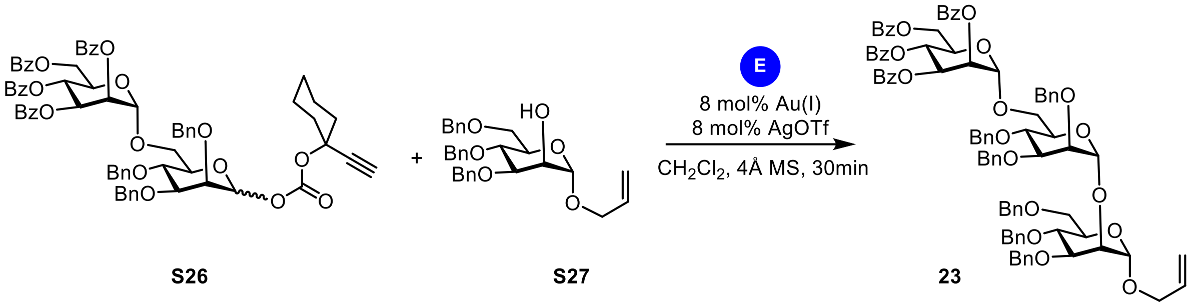

The glycosyl donor **S26** and the acceptor **S27**[30] was coupled according to general experimental procedure E to give compound **23** (Yield 88%, yellow syrup).

$[\alpha]_D^{25}$ = +12º (*c* 0.20 $CHCl_3$); ^1H NMR (400 MHz, $CDCl_3$): δ 8.16 – 8.00 (m, 4H), 7.95 – 7.77 (m, 4H), 7.61 – 7.16 (m, 42H), 6.10 (t, *J* = 10.0 Hz, 1H), 5.94 (d, *J* = 7.0 Hz, 1H), 5.88 – 5.76 (m, 2H), 5.27 (d, *J* = 3.2 Hz, 1H), 5.24 (d, *J* = 3.2 Hz, 1H), 5.21 – 5.15 (m, 1H), 5.08 – 5.00 (m, 2H), 4.95 (d, *J* = 3.2 Hz, 1H), 4.86 – 4.37 (m, 14H), 4.17 (d, *J* = 9.0 Hz, 2H),), 4.02 -3.97 (m, , 6H), 3.92 – 3.85 (m, 1H), 3.83 – 3.76 (m, 3H), 3.76 – 3.65 (m, 2H); ^{13}C NMR (101 MHz, $CDCl_3$): δ 166.3, 165.6, 165.3, 165.2, 138.7, 138.6, 138.6, 138.6, 138.4, 138.3, 134.0, 133.5, 133.1, 130.1, 130.0(3C), 129.9(3C), 129.9(3C), 129.9(3C), 129.6, 129.4, 129.2, 128.7-127.5(36C), 117.3, 99.0, 98.4, 98.0, 80.4, 79.9, 75.3, 75.2, 74.9, 74.7, 74.5, 73.8, 73.5, 72.6, 72.1, 72.1, 72.0, 72.0, 70.5, 70.2, 69.3, 68.9, 68.2, 67.4, 67.1, 62.8; IR ($CHCl_3$): 3005, 1728, 1453, 1264, 1093, 1027, 745, 709, 665 cm^{-1}; HRMS (*m/z*): $[M+H]^+$ calcd. for $C_{91}H_{89}O_{20}$, 1501.5947; found, 1501.5944.

S2.18 Synthesis of linear heptasaccharide Glycan unit (**24**):

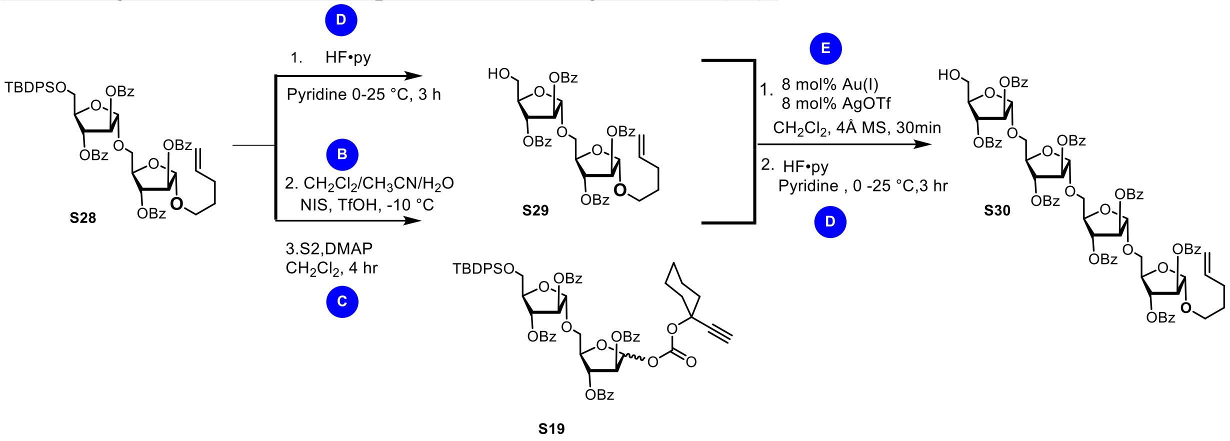

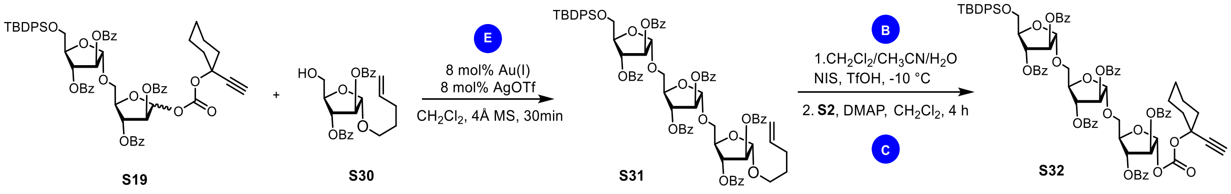

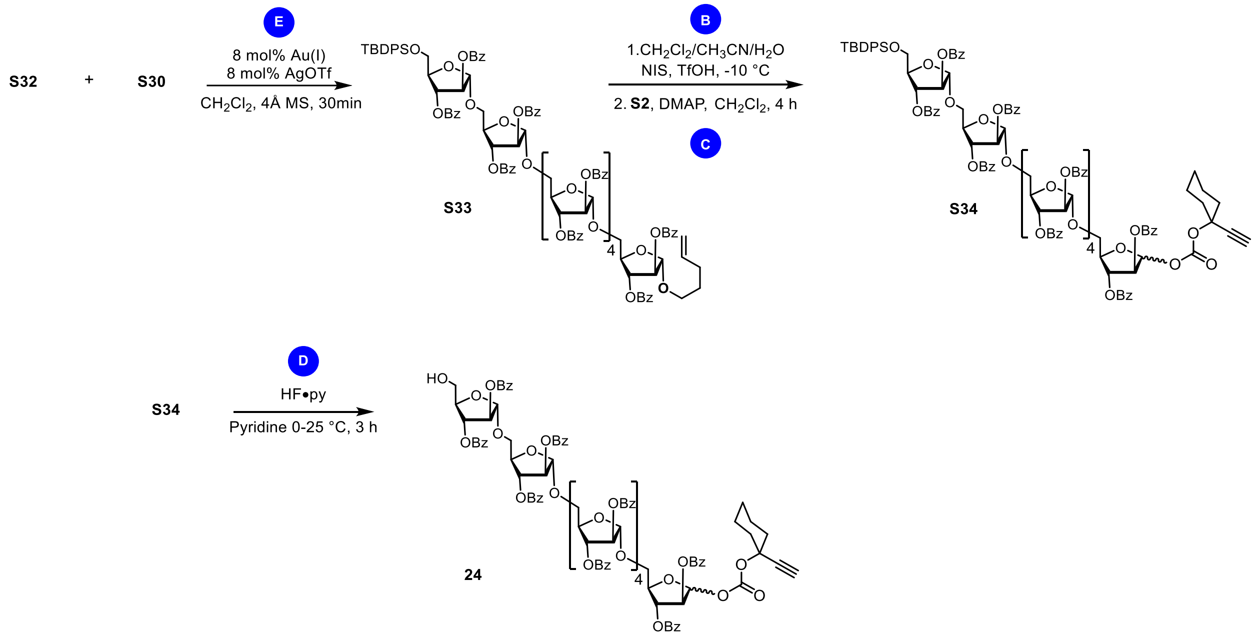

Pent-4-enyl-2,3 di-*O*-benzoyl 5-*O*-(2, 3-di-*O*-benzoyl-5-*O*-(2,3-di-*O*-benzoyl-5-*O*-(2,3-di-*O*-benzoyl-α-D-arabinofuranosyl)-α-D-arabinofuranosyl)-α-D- arabinofuranosyl)- α-D-arabinofuranoside (**S26**):

Then the donor **S19**[29] and the acceptor **S29**[29] were coupled according to the general glycosylation method E to obtain compound the tetrasaccharide. After that primary TBDPS group was deprotected following the general experimental method D to obtain compound **S30** (84 % overall yield, thick syrup). $[\alpha]_D^{25}$ = - 9º (*c* 0.23, $CHCl_3$); ^{1}H NMR (400 MHz, $CDCl_3$): δ 8.09 – 7.84 (m, 16H), 7.63 – 7.16 (m, 24H), 5.87 – 5.76 (m, 1H), 5.69 – 5.61 (m, 6H), 5.52 (d, *J* = 1.3 Hz, 1H), 5.43 (s, 1H), 5.42 (s, 1H), 5.41 (s, 2H), 5.22 (s, 1H), 5.05 – 4.93 (m, 2H), 4.62 (dt, *J* = 4.5, 2.2 Hz, 2H), 4.50 – 4.41 (m, 2H), 4.24 – 4.14 (m, 3H), 3.95 – 3.85 (m, 5H), 3.82 – 3.72 (m, 1H), 3.57 – 3.47 (m, 1H), 2.43 (s, 1H), 2.24 – 2.13 (m, 2H), 1.81 – 1.66 (m, 2H); ^{13}C NMR (101 MHz, $CDCl_3$): δ 166.2, 165.8 (2C), 165.5, 165.3(2C), 165.2(2C), 138.2, 133.6, 133.5(4C), 133.4, 133.3, 133.3, 130.0(4C), 129.9(8C), 129.9(4C), 129.4, 129.3, 129.29(4C), 129.1, 129.1 128.6(8C), 128.6(2C), 128.4(2C), 128.4(4C), 115.1, 105.9, 105.9, 105.7, 105.7, 83.8, 82.2, 82.1, 82.0, 81.9, 81.8, 81.7, 81.6, 77.8, 77.4, 76.9, 66.8, 66.2, 66.1, 65.9, 62.4, 30.4, 28.8; IR ($CHCl_3$): 3453, 3022, 1720, 1264, 1216, 1109, 1027, 745, 702, 667 cm^{-1}; HRMS (*m/z*): $[M+Na]^+$ calcd. for $C_{81}H_{74}O_{25}$, 1469.4417; found, 1469.4412.

1-*O*-(((1-ethynylcyclohexyl)oxy) carbonyl) 2,3 di-*O*-benzoyl 5-*O*-(2,3-di-*O*-benzoyl-5-*O*-(2,3-di-*O*-benzoyl)-5-*O*-*tert*-butyldiphenylsilyl-α-D-arabinofuranosyl)-α-D-arabinofuranosyl)-α-D-arabinofuranoside **(S32)**:

The glycosyl donor **S19** and acceptor **S30**[29] was coupled according to general glycosylation method E to obtain compound **S31** .The obtained compound **S31** was converted to compound **S32** according to general experimental procedure A,C (88% overall yield, viscous syrup).

$[\alpha]_D^{25}$ = -2º (*c* 0.16 , $CHCl_3$); ^{1}H NMR (400 MHz, $CDCl_3$): δ 8.11 – 7.92 (m, 12H), 7.75 – 7.66 (m, 4H), 7.61 – 7.30 (m, 24H), 6.35 (s, 1H), 5.73 (dt, *J* = 5.1, 0.9 Hz, 2H), 5.68 – 5.62 (m, 2H), 5.61 (t, *J* = 1.0 Hz, 1H), 5.58 (d, *J* = 1.3 Hz, 1H), 5.41 – 5.37 (m, 2H), 4.68 – 4.60 (m, 2H), 4.51 (q, *J* = 4.6 Hz, 1H), 4.26 – 4.15 (m, 2H), 4.02 – 3.90 (m, 4H), 2.67 – 2.64 (m, 1H), 2.30 – 2.09 (m, 2H), 2.06 – 1.53 (m, 8H), 1.02 (s, 9H); ^{13}C NMR (101 MHz, $CDCl_3$): δ 165.7, 165.79(2C), 165.4, 165.3, 165.2, 151.1, 135.8(2C), 133.8, 133.7, 133.5, 133.4, 133.3, 133.3, 133.2, 130.1, 130.1, 130.0, 129.9, 129.9, 129.8, 129.3(2C), 129.2(2C), 129.1(2C), 128.7, 128.6, 128.5, 128.3, 127.8, 126.2, 115.7, 106.1, 106.0, 102.4, 84.3, 83.3, 82.6, 82.3, 82.2, 81.6, 81.0, 78.5, 75.5, 65.9, 65.8, 63.5, 36.9, 36.8, 26.9(3C), 25.0, 22.8, 22.7, 19.4; IR ($CHCl_3$): 3297, 2864, 2939, 1727, 1599, 1453, 1363, 1261, 1107, 1020, 966, 852, 756, 708 cm^{-1}; HRMS (*m/z*): $[M+Na]^+$ calcd. for $C_{82}H_{78}O_{21}SiNa$, 1449.4703; found, 1449.4725.

1-*O*-(((1-ethynylcyclohexyl) oxy) carbonyl) 2, 3 di-*O*-benzoyl 5-*O*-(2, 3-di-*O*-benzoyl-5-*O*-(2,3-di-*O*-benzoyl-5-*O*-(2,3-di-*O*-benzoyl-5-*O*-(2,3-di-*O*-benzoyl-5-*O*-(2,3-di-*O*-benzoyl-5-*O*-(2,3-di-*O*-benzoyl-5-*O*-α-D-(2,3-di-*O*-benzoyl-5-*O*-*tert*-butyldiphenylsilyl-α-D-arabinofuranosyl)-α-D-arabinofuranosyl)-α-D-arabinofuranosyl)-α-D-arabinofuranosyl)-α-D-arabinofuranosyl)-α-D-arabinofuranosyl)-α-D-arabinofuranoside (**S34**):

The donor **S30** and acceptor **S30**were coupled according to general glycosylation procedure E to obtain the product **S33**, which was converted to carbonate donor **S34** according to general experimental procedure A, C (80 % overall yield, viscous syrup).

$[\alpha]_D^{25}$ = -10º (*c* 0.16 $CHCl_3$); ^{1}H NMR (400 MHz, $CDCl_3$): δ 8.09 – 7.85 (m, 28H), 7.74 – 7.24 (m, 52H), 6.33 (d, *J* = 4.1 Hz, 1H), 5.72 (d, *J* = 1.7 Hz, 1H), 5.65 (d, *J* = 3.9 Hz, 3H), 5.63 (s, 3H), 5.62 (s, 3H), 5.61 (d, *J* = 3.7 Hz, 1H), 5.59 (d, *J* = 1.3 Hz, 1H), 5.55 (d, *J* = 1.4 Hz, 1H), 5.37 (d, *J* = 6.2 Hz, 6H), 4.66 – 4.47 (m, 7H), 4.25 – 4.10 (m, 7H), 4.00 – 3.86 (m, 8H), 2.64 (s, 1H), 2.29 – 1.99 (m, 2H), 1.95 – 1.63 (m, 8H), 1.00 (s, 9H); ^{13}C NMR (101 MHz, $CDCl_3$): δ 165.6(4C), 165.6, 165.5, 165.2(2C), 165.1(4C), 165.1(2C), 150.9, 135.7(2C), 135.7(2C), 133.7-133.1(16C), 130.0-129.7(24C), 129.3(2C), 129.3(4C), 129.1(4C), 129.1(4C), 128.7(2C), 128.6-128.2(24C), 127.7(12C), 106.0(3C), 105.9(3C), 102.3, 84.3, 83.2(2C), 82.6, 82.2(2C), 82.1(2C), 82.1(2C), 82.1(2C), 81.5(4C), 81.0, 78.4, 77.3(2C), 77.3(2C), 77.2, 75.4, 65.8(6C), 63.4, 36.8, 36.7, 26.8(3C), 25.0, 22.7, 22.6, 19.3; IR ($CHCl_3$): 3016, 2941, 1718, 1453, 1264, 1109, 1071, 1027, 747, 707, 667 cm^{-1}; HRMS (*m/z*): $[M+Na]^+$ calcd. for $C_{158}H_{142}O_{45}SiNa$, 2810.8524; found, 2810.8521.

1-*O*-(((1-ethynylcyclohexyl) oxy) carbonyl) 2, 3 di-*O*-benzoyl 5-*O*-(2, 3-di-*O*-benzoyl-5-*O*-(2,3-di-*O*-benzoyl-5-*O*-(2,3-di-*O*-benzoyl-5-*O*-(2,3-di-*O*-benzoyl-5-*O*-(2,3-di-*O*-benzoyl-5-*O*-(2,3-di-*O*-benzoyl-α-D-arabinofuranosyl)-α-D-arabinofuranosyl)-α-D-arabinofuranosyl)-α-D-arabinofuranosyl)-α-D-arabinofuranosyl)-α-D-arabinofuranosyl)-α-D-arabinofuranoside (**24**):

The compound **24** was synthesized from compound **S34** according to general experimental method D (78% yield, white solid).

mp: 96.5 °C; $[\alpha]_D^{25}$ = + 5° (*c* 0.17, $CHCl_3$); ^{1}H NMR (400 MHz, $CDCl_3$): δ 8.14 – 7.84 (m, 28H), 7.60 – 7.24 (m, 42H), 6.33 (s, 1H), 5.76 – 5.57 (m, 13H), 5.43 – 5.35 (m, 6H), 4.67 – 4.41 (m, 8H), 4.25 – 4.10 (m, 6H), 4.02 – 3.85 (m, 8H), 2.65 (s, 1H), 2.34 (s, 1H), 2.26 – 2.11 (m, 2H), 2.07 – 1.63 (m, 8H); ^{13}C NMR (101 MHz, $CDCl_3$): δ 166.1, 165.7, 165.6(4C), 165.2(4C), 165.2(4C), 151.0, 133.7-133.2(10C), 130.1-129.9 (30C),129.3 (2C), 129.2 (2C), 129.1(4C), 129.1(4C), 129.0(2C), 128.6 - 128.3(30C), 106.1, 106.0(5C), 102.4, 84.3, 83.7(2C), 82.7, 82.3, 82.2(4C), 82.1, 81.8, 81.7, 81.6(4C), 81.0, 78.5, 77.8, 77.4(2C), 77.3, 75.5, 66.2, 65.9(5C), 62.4, 36.9, 36.8, 25.0, 22.7, 22.7; IR ($CHCl_3$): 3016, 2940, 1720, 1452, 1264, 1105, 1070, 1026, 747, 707, 666 cm^{-1}; HRMS (*m/z*): $[M+Na]^+$ calcd. for $C_{142}H_{124}O_{45}SiNa$, 2572.7346; found, 2572.7385.

S2.19 1-*O*-(((1-ethynylcyclohexyl) oxy) carbonyl) 2-*O*-benzoyl-3,5-di-*O*-(2,3-di-*O*-benzoyl-5-*O*-(2,3-di-*O*-benzoyl-5-*O*-(2,3-di-*O*-benzoyl-α-D-arabinofuranosyl)-α-D-arabinofuranosyl)-α-D-arabinofuranosyl)-α/ β -D-arabinofuranoside (**26**):

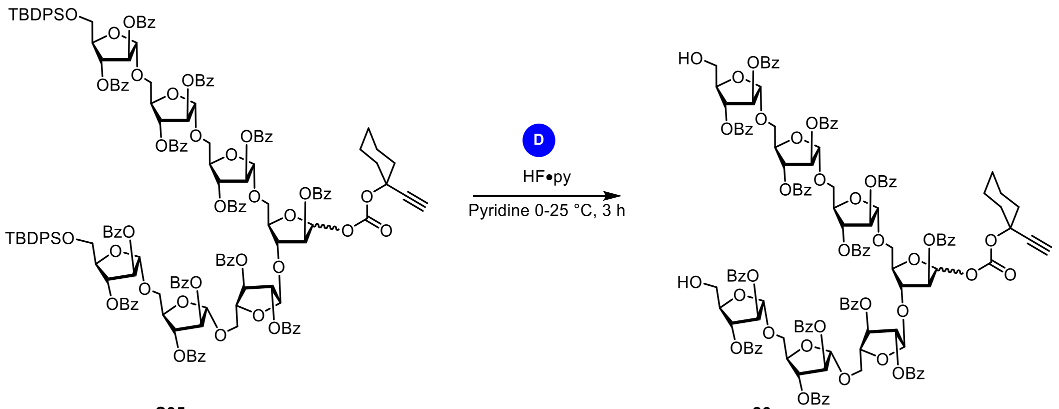

S35 **26**

The compound **S35**[29] was subjected to di-TBDPS deprotection according to general reaction procedure D to afford the compound **26**. (Yield 81%, white solid) (α :β = 2:1).

mp: 106 °C; $[\alpha]_D^{25}$ = -5° (*c* 0.16 $CHCl_3$); ^{1}H NMR (400 MHz, $CDCl_3$): δ 8.11 – 7.78 (m, 26H), 7.58 – 7.22 (m, 39H), 6.31 (s, 1H), 5.68 (d, *J* = 4.8 Hz, 1H), 5.59 (t, *J* = 4.9 Hz, 7H), 5.55 (d, *J* = 10.6 Hz, 1H), 5.52 (d, *J* = 2.8 Hz, 2H), 5.45 – 5.31 (m, 6H), 5.28 (s, 2H), 4.57 (d, *J* = 15.2 Hz, 4H), 4.46 – 4.34 (m,

4H), 4.22 – 4.06 (m, 4H), 4.03 – 3.94 (m, 3H), 3.94 – 3.76 (m, 7H), 2.63 (s, 1H), 2.53 – 2.31 (m, 2H), 2.25 – 1.99 (m, 2H), 1.95 – 1.50 (m, 8H); ^{13}C NMR (101 MHz, $CDCl_3$): δ 166.0 (2C), 166.0 (2C), 165.6 (4C), 165.6 (4C), 165.4, 165.3 (2C), 165.2 (5C), 165.2, 165.1 (5C), 151.2, 151.0, 133.5-133.2 (30C), 130.1 (2C), 130.0-129.7 (50C), 129.2 (4C), 129.2 (4C), 129.1 (4C), 129.0 (4C), 129.0 (6C), 128.8 (2C), 128.5-128.3 (50C), 106.0 (2C), 105.9 (4C), 105.8 (4C), 105.3 (2C), 102.8, 102.7, 83.7 (8C), 82.9 (2C), 82.6 (2C), 82.5 (2C), 82.4, 82.3, 82.1 (2C), 81.9, 81.7 (8C), 81.6 (2C), 81.4, 81.4 (2C), 80.0 (2C), 78.5 (2C), 77.7 (4C), 77.3 (6C), 77.0 (2C), 75.4 (2C), 66.0 (2C), 65.6 (2C), 65.6 (2C), 65.5 (2C), 65.3 (2C), 62.3 (4C), 36.9, 36.7, 36.6, 36.5, 25.0, 24.8, 22.7, 22.6, 22.6, 22.5; IR ($CHCl_3$): 3390, 2935, 1719, 1602, 1452, 1249, 1178, 1107, 1069, 1027, 754, 708 cm^{-1}; HRMS (*m/z*): $[M+Na]^+$ calcd. for $C_{135}H_{120}O_{44}$, 2468.7084; found, 2468.7088.

S3. One-pot glycan Editing by **CIStER**:

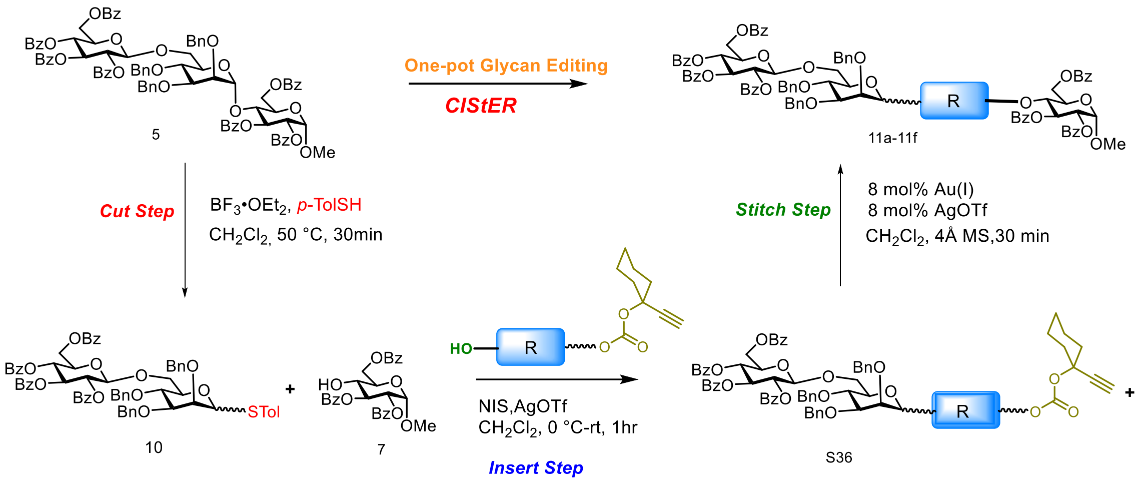

Inserted Glycans

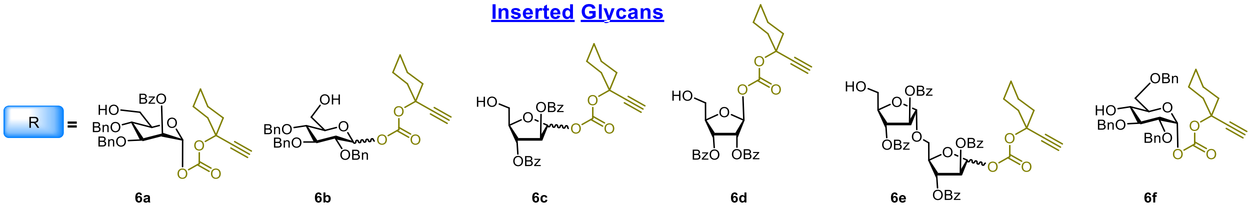

Stitched Products

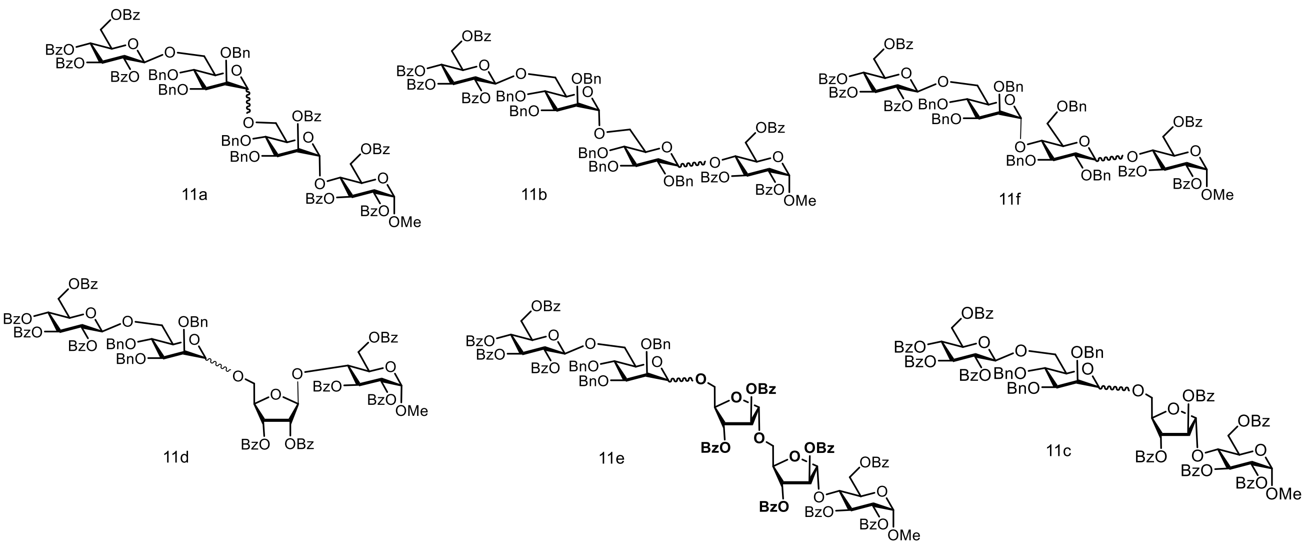

Procedure for one-pot Glycan Editing methodology: To a solution of trisaccharide **5** (1eq., 300 mg) in 3 mL of anhydrous CH_2Cl_2, BF_3•OEt_2 (3 eq, 74 µL) was added at 0 °C. After 10 min, reaction mixture was refluxed at 50 °C for 30 min. The reaction mixture was brought to 25 °C, 1 equivalent of glycosyl acceptor 6**a-6e** [**6a** (121mg), **6b** (118 mg), **6c** (100 mg), **6d** (100 mg,),or **6e** (167 mg)] were added to the reaction mixture and cooled to 0 °C. Freshly activated 4Å MS powder (80mg) was added at 0 °C under nitrogen atmosphere and kept for vigorous stirring for another 15 min. Then NIS (1.5 eq, 66 mg), AgOTf (1.5 eq, 10 mg) were added simultaneously. The reaction mixture was stirred for 15 min at 0 °C and gradually warmed to 25 °C and stirred for 45 min. After this, chloro[tris(2,4- di^{t}butylphenyl)phosphite] gold(I) (0.08 eq, 13 mg), AgOTf (0.1 eq, 5 mg) were added simultaneously to the reaction mixture and stirred for another 30 min. Et_3N was added to quench the reaction mixture and the solvent was removed under reduced pressure. The crude residue was purified by silica gel column chromatography (30% - 40% ethyl acetate/hexane) to afford the compounds **11a-11e**.

Supplementary Figure S1: LC Profile of CIStER:

HPLC system: Gilson's PLC 2050 series
Column: Chromasol ONYX DIOL column (5μ, 4.6 mm ×250 mm)
Mobile Phase A: Ethyl Acetate
Mobile Phase B: n-hexane
Column temp: 25 °C
Sample temp: 25 °C
Flow Rate: 1ml/min
Run time: 60 min
Uv-detection: 270nm
Mobile Phase: 20% ethyl acetae/ n-hexane (Isocratic 20% 60 min)

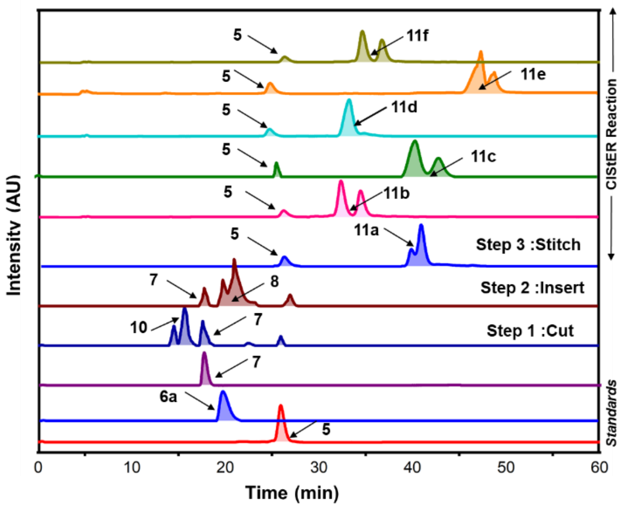

Progress of the one-pot CIStERs was monitored by using semi-preparative HPLC system equipped with a normal phase silica gel diol column. Initially, mobile phase conditions were optimized by injecting the three standards samples **5**, **6a**, and **7** so that the peaks are well resolved for identification purpose. Subsequently, aliquots of CIStER reaction (**11a-11e**) were injected under above optimized mobile phase conditions. Products **11a-11e** and intermediates during the CIStER were collected and characterized.

p-Tolyl-2,3,4-tri-*O*-benzyl-6-*O*-(2,3,4,6-tetra-*O*-benzoyl-α-D-glucopyranosyl)-1-thio-α/β-D-glucopyranoside (**10**):

This compound was synthesized from the trisaccharide **5** (Cut step of the CIStER) according to the one pot glycan editing methodology mentioned above (85% yield, white solid) (α :β = 5:1).

mp: 98 °C; $[\alpha]_D^{25}$ = +8º (*c* 0.26, $CHCl_3$); ^{1}H NMR (400 MHz, $CDCl_3$): δ 8.02 – 7.81 (m, 8H), 7.53 – 7.21 (m, 29H), 7.07 (d, *J* = 8.5 Hz, 2H), 5.85 (t, *J* = 9.6, 2.7 Hz, 1H), 5.64 (t, *J* = 11.2, 8.5 Hz, 1H), 5.58 (d, *J* = 4.1 Hz, 1H), 4.91 (dd, *J* = 11.8, 3.1 Hz, 1H), 4.86 – 4.73 (m, 1H), 4.78 – 4.28 (m, 9H), 4.15 – 4.02 (m, 2H), 3.89 (dd, *J* = 10.9, 3.0 Hz, 1H), 3.76 (dt, *J* = 5.9, 3.0 Hz, 2H), 3.50 (d, *J* = 8.9 Hz, 1H), 2.31 (s, 3H); ^{13}C NMR (101 MHz, $CDCl_3$): δ 166.3, 166.2, 165.9, 165.9, 165.3, 165.3, 165.1, 165.1, 138.9, 138.8, 138.4, 138.2, 138.0, 137.8, 137.6, 137.2, 133.5, 133.5, 133.2, 133.1, 132.4(2C), 130.5, 130.5, 130.2-129.9(32C), 129.7, 129.6, 129.4, 129.4, 129.2, 129.2, 128.7 -127.8(40C), 101.1, 101.1, 87.8, 87.4, 82.4, 80.7, 79.8, 77.4, 75.7, 75.5 (2C), 75.0, 74.8(2C), 73.2(2C), 73.0(2C), 72.6(2C), 72.3(2C), 72.0, 71.9(2C), 70.7(2C), 69.9, 68.1, 68.0, 63.4, 63.2, 21.3, 21.2; IR ($CHCl_3$): 2990, 1728,

1265, 1215, 1108, 1027, 903, 742, 708, 666 cm^{-1}; HRMS (*m/z*): $[M+Na]^+$ calcd. for $C_{68}H_{62}O_{14}S$, 1158.3791; found 1158.3785.

Methyl-2,3,6-tri-*O*-benzoyl-4-*O*-(2,3,-di-*O*-benzoyl-5-*O*-(2,3,4-tri-*O*-benzyl-6-O-(2,3,4,6-tetra-*O*-benzoyl-β-D-glucopyranosyl)-α/β-D-mannopyranosyl)-α-D-arabinofuranosyl)-α-D-glucopyranoside (**11c**):

This compound was synthesized according the one pot glycan editing procedure mentioned above. (68% overall yield, white solid). (α :β = 2:1).

mp: 111 °C; $[\alpha]_D^{25}$ = + 34º (*c* 0.23 $CHCl_3$); ^1H NMR (400 MHz, $CDCl_3$): δ 8.13 – 7.74 (m, 18H), 7.58 – 7.15 (m, 42H), 6.27 – 6.06 (m, 1H), 5.90 (t, *J* = 9.7 Hz, 1H), 5.76 – 5.59 (m, 2H), 5.54 (d, *J* = 7.5 Hz, 1H), 5.49 (d, *J* = 9.5 Hz, 1H), 5.40 (d, *J* = 8.2 Hz, 2H), 5.21 (d, *J* = 5.2 Hz, 1H), 5.17 (s, 1H), 4.88 (d, *J* = 7.6 Hz, 1H), 4.86 – 4.73 (m, 1H), 4.71 – 4.43 (m, 8H), 4.39 (d, *J* = 4.9 Hz, 1H), 4.33 (d, *J* = 11.6 Hz, 1H), 4.28 (d, *J* = 11.2 Hz, 1H), 4.25 – 4.19 (m, 3H), 4.15 (d, *J* = 13.9 Hz, 1H), 3.90 (s, 1H), 3.74 (dd, *J* = 19.0, 9.8 Hz, 4H), 3.61 (t, *J* = 9.7 Hz, 1H), 3.44 (s, 3H); ^{13}C NMR (101 MHz, $CDCl_3$): δ 166.3, 166.2, 166.1, 166.1, 166.1, 165.9, 165.7, 165.4, 165.4, 165.3, 165.3, 165.2, 165.2, 165.1, 164.9, 164.8, 164.7, 164.7, 138.7, 138.6, 138.6, 138.5, 138.1, 138.0, 133.5-132.8(20C), 130.1- 129.5(20C), 129.4(2C), 129.3(2C), 129.2(2C), 129.1(2C), 128.9 (2C), 128.9(2C), 128.8(2C), 128.8(2C), 128.7(2C), 128.6-128.1(60C), 127.9-127.3(20C), 107.8, 107.4, 101.7(2C), 101.3, 98.2, 97.0(2C), 83.3, 82.2(2C), 82.1, 82.0, 80.2, 77.7, 77.3, 75.2, 74.8, 74.7, 74.5, 74.5, 74.2, 74.0, 73.0, 72.8, 72.4(4C), 72.4, 72.3, 72.1, 71.9, 71.9, 71.6, 71.2, 71.1, 69.9(2C), 69.6, 69.3, 68.9, 68.8, 68.8, 68.6, 66.1, 63.4 (2C), 63.3 (2C), 55.5, 55.5; IR ($CHCl_3$): 3010 ,1727, 1268, 1215, 1095, 745, 708, 667 cm^{-1}; HRMS (*m/z*): $[M+Na]^+$ calcd. for $C_{108}H_{96}O_{29}Na$, 1880.5962; found 1882.5971.

Methyl-2,3,6-tri-*O*-benzoyl-4-*O*-(2,3,4-tri-*O*-benzyl-6-*O*-(2,3,4-tri-*O*-benzyl-6-O-(2,3,4,6-tetra-*O*-benzoyl-β-D-glucopyranosyl)-α-D-mannopyranosyl)-α/β-D-glucopyranosyl)-α-D-glucopyranoside **(11b)**:

This compound was synthesized according the one pot glycan editing procedure mentioned above (65% overall yield, thick syrup) (α :β = 2:1).

$[\alpha]_D^{25}$ = + 47º (*c* 0.16 $CHCl_3$); ^1H NMR (600 MHz, $CDCl_3$): δ 8.18 – 7.86 (m, 24H), 7.56 – 7.08 (m, 106H), 6.28 (t, *J* = 10.2 Hz, 1H), 6.00 (t, *J* = 9.6 Hz, 1H), 5.74 (t, *J* = 9.8 Hz, 1H), 5.61 (t, *J* = 8.9 Hz, 1H), 5.37 (dd, *J* = 10.3, 3.7 Hz, 1H), 5.22 (d, *J* = 3.7 Hz, 1H), 5.14 (dd, *J* = 11.9, 4.0 Hz, 1H), 5.08 (d, *J* = 8.2 Hz, 1H), 4.97 (d, *J* = 11.3 Hz, 1H), 4.92 (d, *J* = 4.0 Hz, 2H), 4.89 (d, *J* = 6.3 Hz, 1H), 4.86 (d, *J* = 10.4 Hz, 2H), 4.80 (d, *J* = 8.7 Hz, 5H), 4.76 – 4.39 (m, 24H), 4.39 – 4.23 (m, 7H), 4.23 – 4.17 (m,

2H), 4.14 (t, J = 11.0 Hz, 2H), 4.10 (d, J = 11.4 Hz, 2H), 3.96 (dd, J = 11.1, 7.8 Hz, 4H), 3.82 – 3.65 (m, 11H), 3.63 – 3.56 (m, 3H), 3.54 – 3.47 (m, 3H), 3.45 (s, 1H), 3.44 (s, 3H), 3.32 (t, J = 9.5 Hz, 2H), 3.24 – 3.14 (m, 3H); ^{13}C NMR (151 MHz, $CDCl_3$): δ 166.3, 166.2, 166.1, 166.1, 166.1, 166.0, 166.0, 165.9, 165.9, 165.7, 165.3, 165.2, 165.2, 165.1, 139.2, 138.8, 138.6, 138.6, 138.5, 138.5, 138.4, 138.4, 138.3, 138.2, 138.2, 137.9, 133.4 - 133.0 (20C), 130.2-129.6 (30C), 129.2 (2C), 129.1 (2C), 129.1 (2C), 129.0 (2C), 128.9 (2C), 128.9 (2C), 128.7 (2C), 128.6, 128.5 - 128.1 (50C), 127.8 - 127.4 (30C), 101.5, 101.3, 101.0, 100.9, 99.2, 97.9, 96.9, 96.8, 82.7, 81.5, 81.4, 79.6, 79.4, 79.2, 79.1, 76.4, 75.6, 75.3, 75.2, 74.8, 74.7, 74.6, 74.2, 74.1, 73.5, 73.1, 73.0, 73.0, 72.7, 72.7, 72.5, 72.3, 72.3, 72.1 , 72.1, 72.1, 72.0, 71.9, 71.8, 71.7, 71.7, 71.6, 71.6, 71.5, 71.2, 69.9, 69.8, 69.3, 69.0, 68.8, 68.6, 68.4, 68.1, 65.6, 65.1, 63.8, 63.6, 63.4, 63.3, 55.4, 55.3; IR ($CHCl_3$): 2922, 1723, 1452, 1263, 1068, 1026, 749, 707 cm^{-1}; HRMS (*m/z*): $[M+Na]^+$ calcd. for $C_{116}H_{108}O_{28}Na$, 1972.6963; found 1972.6972.

Methyl-2, 3, 6-tri-*O*-benzoyl-4-*O*-(2-*O*-benzoyl 3,4-di-*O*-benzyl 6-*O*-(2,3,4-tri-*O*-benzyl-6-O-(2,3,4,6-tetra-*O*-benzoyl-β-D-glucopyranosyl)-α-D- mannopyranosyl)-α-D-mannopyranosyl)-α-D-glucopyranoside **(11a)** :

This compound was synthesized according the one pot glycan editing procedure mentioned above (61% overall yield, white solid) (α :β = 2:1).

mp: 99 °C; $[\alpha]_D^{25}$ = 43° (*c* 0.20, $CHCl_3$); ^{1}H NMR (400 MHz, $CDCl_3$): δ 8.11 – 7.69 (m, 16H), 7.50 – 7.13 (m, 49H), 6.13 (d, J = 9.9 Hz, 1H), 5.92 (t, J = 9.7 Hz, 1H), 5.67 (t, J = 9.7 Hz, 1H), 5.62 – 5.52 (m, 1H), 5.31 (s, 1H), 5.20 (s, 1H), 5.16 (s, 1H), 4.98 (d, J = 7.8 Hz, 1H), 4.92 (d, J = 13.0 Hz, 1H), 4.84 – 4.76 (m, 2H), 4.73 – 4.06 (m, 18H), 3.98 (d, J = 6.6 Hz, 2H), 3.90 – 3.74 (m, 4H), 3.68 (d, J = 8.3 Hz, 2H), 3.59 (t, J = 11.2 Hz, 1H), 3.37 (s, 3H); ^{13}C NMR (101 MHz, $CDCl_3$): δ 166.3, 166.3, 166.3, 166.2, 166.1, 166.0, 166.0, 165.9, 165.9, 165.8, 165.7, 165.7, 165.3, 165.2, 165.1, 164.9, 138.8, 138.7, 138.6, 138.5, 138.5, 138.4, 138.3, 138.3, 138.0, 137.9, 133.6 -133.0 (18C), 130.1- 129.7 (40C) , 129.5 (2C), 129.2 (2C), 129.1 (2C), 129.0 (2C), 129.0 (2C), 128.7-128.1 (40C), 128.0 (2C), 127.9 (2C), 127.8-127.4 (30C), 102.0, 101.5, 101.4, 100.5, 100.5, 98.1, 97.2, 96.9, 80.0 (2C), 78.0 (2C), 77.7 (2C) , 77.4 (2C), 75.5 (2C), 75.2 (2C), 74.9 (2C), 74.6 (2C), 73.9 (2C), 73.6 (2C), 73.2 (2C), 72.7 (2C), 72.3 (2C), 72.2 (2C), 72.1 (2C), 72.1 (2C), 71.6 (2C), 71.5 (2C), 71.4 (2C), 71.2 (2C), 70.5 (2C), 69.9 (2C), 69.1 (2C), 68.8 (2C), 65.6 (2C), 63.7 (2C), 63.4(2C), 55.9, 55.6; IR ($CHCl_3$): 2921, 1722, 1452, 1263, 1092, 1067, 1026, 750, 707 cm^{-1}; HRMS (*m/z*): $[M+Na]^+$ calcd. for $C_{116}H_{106}O_{29}Na$, 1986.6756; found, 1986.6765.

Methyl-2, 3, 6-tri-*O*-benzoyl-4-*O*-(2, 3-di-*O*-benzoyl 5-*O*-(2,3,4-tri-*O*-benzyl-6-O-(2,3,4,6-tetra-*O*-benzoyl-β-D-glucopyranosyl)-α-D- mannopyranosyl)-β-D-ribofuranosyl)-α-D-glucopyranoside (**11d)**:

This compound was synthesized according the one pot glycan editing procedure mentioned above. (66% overall yield, white solid). (α :β = 4:1)

mp: 104 °C; $[\alpha]_D^{25}$ = + 49° (*c* 0.17, $CHCl_3$); ^{1}H NMR (400 MHz, $CDCl_3$): δ 8.08 – 7.79 (m, 18H), 7.49 – 7.16 (m, 42H), 6.09 (t, *J* = 9.8 Hz, 1H), 5.91 (t, *J* = 9.7, 1H), 5.68 (dd, *J* = 11.7, 9.4 Hz, 1H), 5.61 – 5.52 (m, 2H), 5.45 (d, *J* = 4.9 Hz, 1H), 5.39 (d, *J* = 3.6 Hz, 1H), 5.20 (d, *J* = 3.6 Hz, 1H), 5.17 (d, *J* = 2.0 Hz, 1H), 4.95 (d, *J* = 4.4 Hz, 1H), 4.91 (d, *J* = 9.0 Hz, 1H), 4.86 (d, *J* = 6.3 Hz, 1H), 4.79 (d, *J* = 6.9 Hz, 1H), 4.75 (d, *J* = 3.4 Hz, 1H), 4.71 (s, 1H), 4.69 – 4.44 (m, 7H), 4.37 (dd, *J* = 11.6, 6.3, 1H), 4.27 – 4.08 (m, 5H), 4.05 – 3.98 (m, 1H), 3.94 – 3.84 (m, 1H), 3.82 – 3.69 (m, 2H), 3.68 – 3.60 (m, 1H), 3.36 (s, 3H); ^{13}C NMR (151 MHz, $CDCl_3$): δ :166.4, 166.3, 166.3, 166.2, 166.1, 166.0, 166.0, 165.7, 165.5, 165.5, 165.4, 165.4, 165.3, 165.2, 165.1, 165.1, 165.1, 165.1, 139.3, 139.3, 139.1, 138.9, 138.5, 138.4, 133.6 - 133.1 (20C), 130.1 -129.9 (40C) , 129.4 (2C), 129.3 (2C), 129.2 (2C), 129.2 (2C), 129.1 (2C), 129.0 (2C), 129.0 (2C), 128.7 - 128.4 (40C), 128.2 (2C), 128.1 (2C), 127.9 - 127.4 (20C), 107.7, 106.7, 101.9, 101.7, 101.6, 98.6, 97.2 (2C), 82.3, 81.9, 81.1, 80.4, 79.4, 78.6, 76.4, 76.0, 75.2, 75.2, 74.9, 74.9, 74.6, 74.6, 74.5, 73.3, 73.2, 73.2, 73.0, 72.5, 72.4, 72.3, 72.1, 72.0, 71.9, 71.8, 71.8, 71.5, 71.2, 70.3, 70.1, 70.0, 69.9, 69.3, 69.1, 68.7, 68.4, 66.7, 63.4, 63.4 , 63.0 (2C), 55.7 (2C); IR ($CHCl_3$): 2942, 1726, 1452, 1264, 1093, 1068, 1026, 747, 705, 666 cm^{-1}; HRMS (*m/z*): $[M+Na]^+$ calcd. for $C_{108}H_{96}O_{29}Na$, 1880.9596; found, 1879.9600.

Methyl-2, 3, 6-tri-*O*-benzoyl-4-*O*-(2, 3-di-*O*-benzoyl 5-*O*- (2, 3-di-*O*-benzoyl 5-*O* -(2,3,4-tri-*O*-benzyl-6-O-(2,3,4,6-tetra-*O*-benzoyl-β-D-glucopyranosyl)-α/β -D-mannopyranosyl)-α-D-arabinofuranosyl)-α-D-arabinofuranosyl)-α-D-glucopyranoside (**11e)**:

This compound was synthesized according the one pot glycan editing procedure mentioned above. (64% overall yield, white solid). (α :β = 3:1)

mp: 107 °C; $[\alpha]_D^{25}$ = + 33° (*c* 0.18, $CHCl_3$); ^{1}H NMR (400 MHz, $CDCl_3$): δ 8.10 – 7.74 (m, 22H), 7.50 – 7.15 (m, 48H), 6.17 (t, *J* = 9.5 Hz, 1H), 5.88 (t, *J* = 9.7 Hz, 1H), 5.67 – 5.58 (m, 3H), 5.50 (s, 1H), 5.48 (s, 1H), 5.41 (s, 1H), 5.36 (d, *J* = 4.9 Hz, 1H), 5.19 (s, 2H), 5.08 (s, 1H), 4.88 (d, *J* = 8.1 Hz, 1H), 4.78 (d, *J* = 9.4 Hz, 1H), 4.74 – 4.44 (m, 9H), 4.35 (d, *J* = 11.4 Hz, 1H), 4.31 – 4.18 (m, 6H), 4.13 – 4.00 (m, 2H), 3.90 (s, 1H), 3.84 – 3.69 (m, 5H), 3.64 (d, *J* = 8.8 Hz, 1H), 3.45 (s, 3H); ^{13}C NMR (101 MHz, $CDCl_3$): δ 166.4, 166.3, 166.3, 166.2, 166.1, 165.9, 165.8, 165.7, 165.5, 165.5, 165.5, 165.4, 165.4, 165.3, 165.2, 165.2, 165.2, 165.1, 165.1, 164.9, 164.6, 138.5(2C), 138.5(2C), 138.4, 137.9, 133.5 - 132.7(20C), 130.0 - 129.6(30C), 129.5(2C), 129.5(2C), 129.4(2C), 129.4(2C), 129.4(2C), 129.2(2C),

129.2(2C), 129.1(2C), 128.9(2C), 128.9(2C), 128.8(2C), 128.6 -128.1(50C), 127.8 -127.3(45C), 107.5, 107.4, 105.9, 105.9, 101.8(2C), 101.2, 98.2, 97.1(2C), 83.2, 83.2, 83.1, 82.0, 81.9, 81.8, 81.8, 81.7, 81.6, 80.2, 77.6, 76.9, 75.2, 74.7, 74.7, 74.4, 74.4, 74.2, 73.6, 73.5, 73.0, 72.7, 72.5, 72.4(4C), 72.3(4C), 72.1, 71.9, 71.9, 71.7, 71.2, 71.2, 69.9, 69.4, 69.4, 68.6, 68.5, 68.5, 66.2, 65.6, 65.6, 63.3, 63.0, 55.5, 55.5; IR ($CHCl_3$): 2922, 1724, 1452, 1268, 1096, 1027, 707 cm^{-1}; HRMS (*m/z*): $[M+Na]^+$ calcd. for $C_{127}H_{112}O_{35}Na$, 2219.6883; found, 2219.6889.

Methyl-2,3,6-tri-*O*-benzoyl-4-*O*-(2,3,6-tri-*O*-benzyl-4-*O*-(2,3,4-tri-*O*-benzyl-6-O-(2,3,4,6-tetra-*O*-benzoyl-β-D-glucopyranosyl)-α-D-mannopyranosyl)-α/β-D-glucopyranosyl)-α-D-glucopyranoside **(11f)**:

This compound was synthesized according the one pot glycan editing procedure mentioned above (70% overall yield, thick syrup) (α :β = 3:1).

$[\alpha]_D^{25}$ = + 46º (c 0.25, $CHCl_3$); ^{1}H NMR (600 MHz, $CDCl_3$) δ 8.12 – 7.78 (m, 14H), 7.42 – 7.18 (m, 51H), 5.98 – 5.77 (m, 1H), 5.74 – 5.65 (m, 1H), 5.61 – 5.49 (m, 1H), 5.33 – 5.27 (m, 1H), 5.17 (dd, *J* = 4.8, 3.0 Hz, 2H), 4.87 (dd, *J* = 11.6, 2.7 Hz, 2H), 4.84 – 4.81 (m, 2H), 4.60 – 4.36 (m, 13H), 4.26 – 4.11 (m, 6H), 4.09 – 4.01 (m, 2H), 3.83 – 3.71 (m, 6H), 3.66 – 3.58 (m, 3H), 3.45 (s, 3H).

^{13}C NMR (151 MHz, $CDCl_3$) δ 166.3, 166.2, 166.2, 166.2, 166.1, 166.0, 165.9, 165.8, 165.3, 165.3, 165.1, 165.0, 165.0, 165.0, 138.9, 138.8, 138.8, 138.7, 138.7, 138.6, 138.5, 138.4, 138.3, 138.0, 138.0, 137.8, 133.4 - 133.0 (20C), 130.2-129.6 (30C), 129.2 (2C), 129.1 (2C), 129.1 (2C), 129.0 (2C), 128.9 (2C), 128.9 (2C), 128.7 (2C), 128.6, 128.5 - 128.1 (50C), 127.8 - 127.4 (30C) 101.8, 101.7, 100.6, 99.7, 99.7, 98.6, 96.9, 96.9, 82.7, 81.5, 81.4, 79.6, 79.4, 79.2, 79.1, 76.4, 75.6, 75.3, 75.2, 74.8, 74.7, 74.6, 74.2, 74.1, 73.5, 73.1, 73.0, 73.0, 72.7, 72.7, 72.5, 72.3, 72.3, 72.1 , 72.1, 72.1, 72.0, 71.9, 71.8, 71.7, 71.7, 71.6, 71.6, 71.5, 71.2, 69.9, 69.8, 69.3, 69.0, 68.8, 68.6, 68.4, 68.1, 65.6, 65.1, 63.8, 63.6, 63.4, 63.3, 55.4, 55.3; IR ($CHCl_3$): 3446, 2937, 1755, 1453, 1268, 1239, 1069, 1005, 895, 847, 749, 695 cm^{-1}; HRMS (*m/z*): $[M+Na]^+$ calcd. for $C_{116}H_{108}O_{28}Na$, 1973.3201; found, 1973.3210.

Cutting of interglycosidic bond of naturally occurring disaccharides:

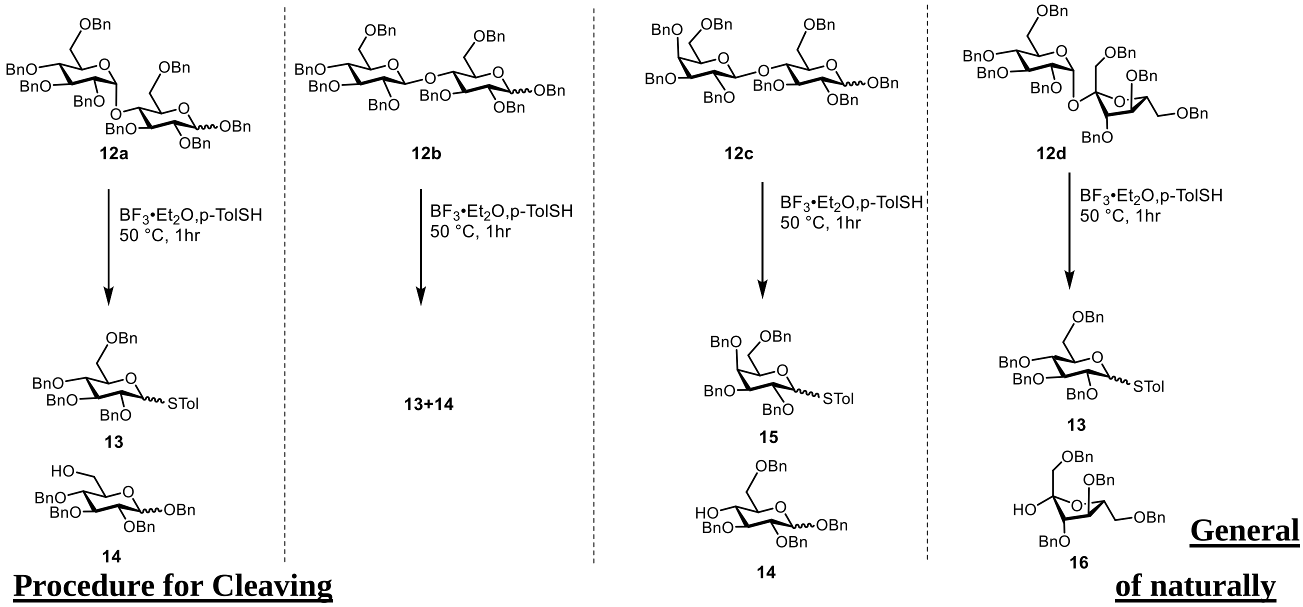

General Procedure for Cleaving of naturally occurring disaccharides:

To a solution of per-*O*-benzylated disaccharides **12a-12d**[33-36] (1eq) in 4 mL of anhydrous CH_2Cl_2, BF_3•OEt_2 (3 eq,) was added at 0 °C. After 10 min, reaction mixture was refluxed at 50 °C for 1hr. Et_3N was added to quench the reaction mixture and the solvent was removed under reduced pressure. The crude residue was purified by silica gel column chromatography (10% - 20% ethyl acetate/hexane) to afford the compounds **13,14,15,16.**

Supplementary Figure S2: LC Profile of CIStER:

HPLC system: Gilson's PLC 2050 series
Column: Chromasol ONYX DIOL column (5µ, 4.6 mm ×250 mm)
Mobile Phase A: Ethyl Acetaate
Mobile Phase B: n-hexane
Column temp: 25 °C
Sample temp: 25 °C
Flow Rate: 1ml/min
Run time: 30 min
Uv-detection: 270nm
Mobile Phase:
10% ethyl acetae/ n-hexane (Isocratic 10% ,30 min)

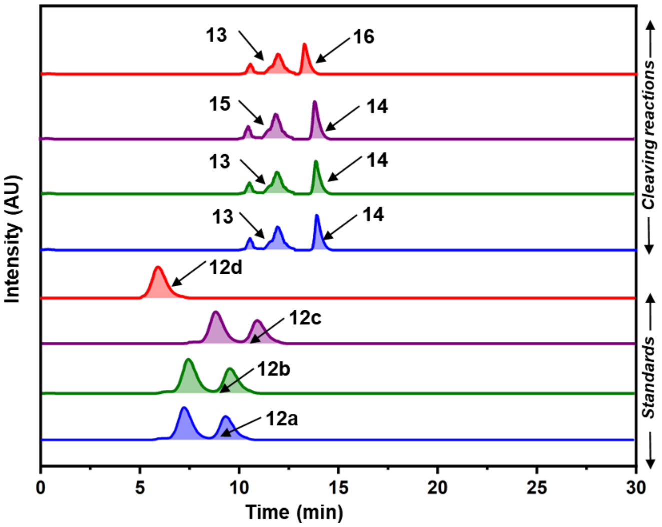

Progress of the cleaving of the per-*O*-benzylated disaccharides was monitored by using semi-preparative HPLC system equipped with a normal phase silica gel diol column. Initially, mobile phase conditions were optimized by injecting the four standards samples **12a-12d** so that the peaks are well resolved for identification purpose. Subsequently, aliquots of cleaving reactions were injected under above optimized mobile phase conditions. Products **13-16** were collected and characterized. The spectra of the compounds 13-16 were matched with the previous reports [36-38].

General Procedure for synthesis of OPfp ester:

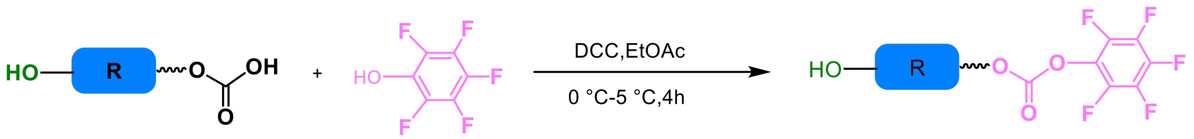

To a solution of compounds 6-hydroxycaproic acid, Lithocholic acid, N-CBz-L-Serine (1mmol) in anhydrous EtOAc (10ml) DCC was added (1.1 mmol) added at 0 °C under nitrogen atmosphere. After stirring for 10 min, pentaflurophenol (1mmol) was added and the reaction was allowed to stir for 4 hr at 25 °C. After completion of reaction, the reaction mixture was filtered filtered off through a pad of Celite®. The filtrate part containing the product was evaporated *in vacuo* and the crude residue was purified by silica gel column chromatography to obtain desired OPfp ester compounds **18a-18c**. (Purified in 10-20 ethyl acetate/hexane). (85-90% yield)

Perfluorophenyl *N*-(benzyloxy)carbonyl)-L-serinate :(**18b**)

This compound was synthesized according the general procedure for OPfp ester synyhesis mentioned above. (88% yield, white powder)

mp: 63 °C; $[\alpha]_D^{25}$ = +23 ° (*c* 0.25, $CHCl_3$); ^1H NMR (400 MHz, $CDCl_3$): δ 7.38 – 7.30 (m, 5H), 5.94 (d, *J* = 8.3 Hz, 1H), 5.15 (s, 2H), 4.82 (dd, *J* = 8.0, 3.9 Hz, 1H), 4.22 (dd, *J* = 11.4, 3.5 Hz, 1H), 4.04 (dd, *J* = 11.3, 3.4 Hz, 1H), 2.68 (s, 1H); IR ($CHCl_3$): 3446, 2937, 1755, 1453, 1268, 1239, 1069, 1005, 895, 847, 749, 695 cm^{-1}; HRMS (*m/z*): $[M+Na]^+$ calcd. for $C_{17}H_{12}F_5NO_3Na$, 428.0533, found 428.0530.

Perfluorophenyl 6-hydroxyhexanoate: (**18a**)

This compound was synthesized according the general procedure for OPfp ester synthesis mentioned above. (88% yield, syrup).

$[\alpha]_D^{25}$ = + 45 ° (*c* 0.25, $CHCl_3$); ^1H NMR (400 MHz, $CDCl_3$): δ 3.70 – 3.64 (m, 2H), 2.69 (t, *J* = 7.3 Hz, 2H), 1.98 (s, 1H), 1.86 – 1.78 (m, 2H), 1.68 – 1.60 (m, 2H), 1.56 – 1.48 (m, 2H); ^{13}C NMR (101 MHz, $CDCl_3$): δ 169.5, 142.3, 140.7, 139.8, 139.1, 138.2, 136.7, 62.4, 33.2, 32.1, 25.1, 24.5; IR (cm^{-1} $CHCl_3$):

3446, 2937, 1755, 1453, 1268, 1239, 969, 1023, 895, 847, 749; HRMS (*m/z*): $[M+Na]^+$ calcd. for $C_{12}H_{11}F_5O_3Na$, 321.0526; found, 321.0524.

Perfluorophenyl (*4R*)-4-((*3R,8R,9S,10S,13R,14S,17R*)-3-hydroxy-10,13-dimethylhexadecahydro-1H-cyclopenta[a]phenanthren-17-yl) pentanoate (**18c**):

This compound was synthesized according the general procedure for OPfp ester synyhesis mentioned above. (90% yield, White powder).

mp: 78 °C; $[\alpha]_D^{25}$ = + 8 ° (*c* 0.25, $CHCl_3$); ^1H NMR (400 MHz, $CDCl_3$): δ 3.63 (tt, *J* = 11.0, 4.6 Hz, 1H), 2.75 – 2.54 (m, 2H), 2.03 – 1.09 (m, 27H), 0.98 (d, *J* = 6.2 Hz, 3H), 0.93 (s, 3H), 0.67 (s, 3H); ^{13}C NMR (101 MHz, $CDCl_3$): δ 170.0, 142.3, 140.6, 139.8, 139.1, 138.1, 136.5, 71.8, 56.5, 55.9, 42.8, 42.1, 40.4, 40.2, 36.4, 35.9, 35.4, 35.3, 34.6, 30.9, 30.5, 30.4, 28.2, 27.2, 26.4, 24.2, 23.4, 20.8, 18.1, 12.0; IR ($CHCl_3$): 3446, 2937, 1755, 1453, 1268, 1239, 1069, 1005, 895, 847, 749, 695 cm^{-1}; HRMS (*m/z*): $[M+Na]^+$ calcd. for $C_{30}H_{39}F_5O_3Na$, 565.2713; found, 565.2710.

Methyl α-D-maltopyranoside (**17**):

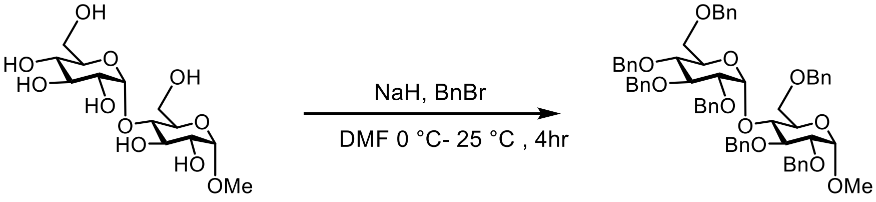

S37 **17**

To a solution of the compound **S37** (1gm, 2.81 mmol) was dissolved in 10 mL of DMF and cooled to 0 °C. To this, NaH (566 mg, 23.57 mmol) was added portion wise over a period of 5 min. The resulting solution was stirred for another 15 min. After that benzyl bromide (2.4 mL, 19.65 mmol) was slowly added. The reaction mixture was kept at 0 °C for 30 min and after that it was shifted to 25 °C. After completion, the reaction mixture was poured into ice cold water. Then it was extracted with ethyl acetate. The organic layer was further washed with brine solution. Next the organic layer was dried over Na_2SO_4 and concentrated in *vacuo*. The crude product was purified by silica gel column chromatography (10% ethyl acetate / hexane) to give the compound **17**. (96% yield, syrup);

$[\alpha]_D^{25}$ = + 40 ° (*c* 0.25, $CHCl_3$); ^1H NMR (400 MHz, $CDCl_3$): δ 7.41 – 7.14 (m, 35H), 5.03 – 4.65 (m, 9H), 4.63 (d, *J* = 3.6 Hz, 2H), 4.61 – 4.43 (m, 6H), 3.99 (t, *J* = 9.3 Hz, 2H), 3.78 – 3.53 (m, 9H), 3.36 (s, 3H); ^{13}C NMR (101 MHz, $CDCl_3$): δ 138.8 (2C), 138.3, 138.2 (2C), 137.9 (2C), 128.4-127.6 (42C), 98.2 (2C), 82.1 (2C), 79.9 (2C), 77.7, 75.7 (2C), 75.0 (2C), 73.5 (2C), 73.4 (2C), 70.1 (2C), 68.5 (2C),

55.2. (2C); IR ($CHCl_3$): 3446, 2937, 1453, 1268, 1239, 1167, 1005, 801, 847, 749 cm^{-1}; HRMS (*m/z*): $[M+Na]^+$ calcd. for $C_{62}H_{66}O_{11}Na$, 1010.4536; found, 1010.4533.

One-pot glycan Editing of Maltose:

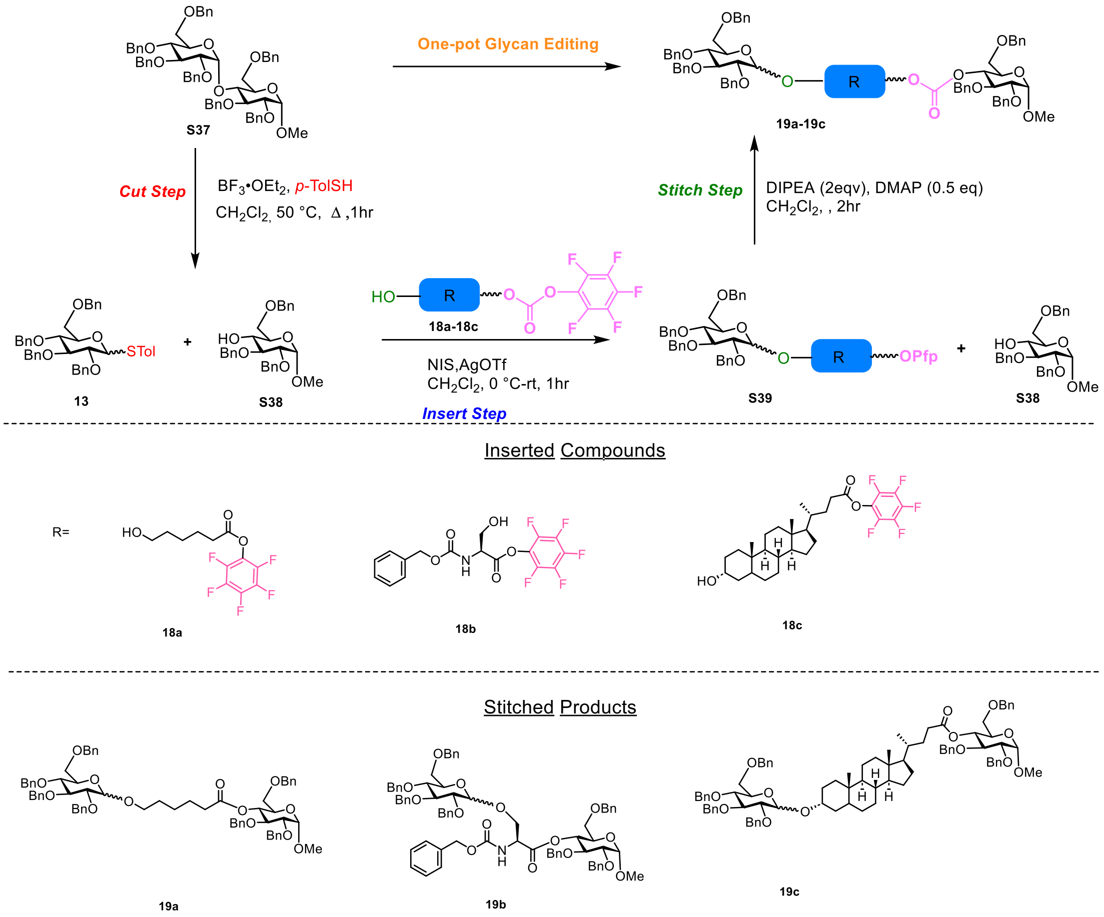

Procedure for one-pot Glycan Editing methodology:

To a solution of disaccharide **17** (1eq, 500 mg) in 4 mL of anhydrous CH_2Cl_2, BF_3•OEt_2 (3 eq, 188 μL) was added at 0 °C. After 10 min, reaction mixture was refluxed at 50 °C for 1hr. The reaction mixture was brought to 25 °C, 1 equivalent of compounds **18a-18c** [**18a** (170mg), **18b** (310mg), **18c** (232 mg)] were added to the reaction mixture and cooled to 0 °C. Freshly activated 4Å MS powder (80mg) was added at 0 °C under nitrogen atmosphere and kept for vigorous stirring for another 15 min. Then NIS (1.5 eq, 194 mg), AgOTf (0.25 eq, 36 mg) were added simultaneously. The reaction mixture was stirred for 15 min at 0 °C and gradually warmed to 25 °C and stirred for 45 min. After this, DIPEA (2eq, 164 μL), DMAP (0.5 eq, 29 mg) were added simultaneously to the reaction mixture and stirred for another 2 hr. After completion, the reaction mixture was concentrated under reduced pressure. The crude residue

was purified by silica gel column chromatography (15% - 20% ethyl acetate/hexane) to afford the compounds **19a-19c**

Methyl 2,3,6-tri-*O*-benzyl-4-*O*-(2,3,4,6-tetra-*O*-benzyl glucopyranosyloxyhexanoyl)-α-D-glucopyranoside (**19a**):

This compound was synthesized according the one pot glycan editing procedure mentioned above (80% overall yield, thick syrup) (α: β = 2:1)

$[\alpha]_D^{25}$ = +51º (*c* 0.25, $CHCl_3$); ^{1}H NMR (400 MHz, $CDCl_3$): δ 7.37 – 7.13 (m, 35H), 5.03 – 4.74 (m, 9H), 4.70 – 4.61 (m, 2H), 4.58 (d, *J* = 3.3 Hz, 2H), 4.57 – 4.44 (m, 3H), 4.36 (d, *J* = 7.8 Hz, 1H), 4.26 (d, *J* = 3.3 Hz, 2H), 4.05 – 3.90 (m, 2H), 3.81 (d, *J* = 9.9 Hz, 1H), 3.73 – 3.42 (m, 8H), 3.36 (s, 3H), 2.26 (td, *J* = 7.8, 3.0 Hz, 2H), 1.68 – 1.57 (m, 4H), 1.46 – 1.33 (m, 2H); ^{13}C NMR (101 MHz, $CDCl_3$) δ 173.3, 173.3, 138.9, 138.7, 138.6, 138.5, 138.4, 138.4, 138.3, 138.3, 138.2, 138.2, 138.1, 138.0, 137.9, 128.5, 128.5, 128.5, 128.4, 128.4, 128.2, 128.1, 128.1, 128.0, 128.0, 128.0, 128.0, 127.9, 127.9, 127.8, 127.8, 127.7, 127.7, 127.7, 127.7, 127.6, 127.6, 103.7, 98.0 (2C), 97.0, 84.8, 84.7, 82.3, 82.1, 82.1, 82.0, 80.1, 80.0, 80.0, 77.9, 77.8, 77.6, 77.6, 75.9, 75.8, 75.7, 75.2, 75.1, 75.0, 75.0, 74.9, 74.8, 73.6, 73.5, 73.4, 73.4, 73.2, 70.2, 69.8, 69.8, 69.0, 68.7, 68.7, 68.6, 67.9, 63.0, 62.9, 55.2, 55.2, 34.0, 29.5, 25.8, 24.7; IR ($CHCl_3$): 3446, 2937, 1755, 1453, 1268, 1239, 1069, 1005, 895, 847, 749, 695 cm^{-1}; HRMS (*m/z*): $[M+Na]^+$ calcd. for $C_{68}H_{76}O1_3Na$, 1123.5183; found, 1123.5184.

Methyl 2,3,6-tri-*O*-benzyl-4-*O*-(2*S*-((benzyloxy)carbonyl)amino-(3-(2,3,4,6-tetra-*O*-benzyl glucopyranosyloxy))propanoyl) α-D-glucopyranoside (**19b**):

This compound was synthesized according the one pot glycan editing procedure mentioned above (76% overall yield, thick syrup) (α: β = 2:1)

$[\alpha]_D^{25}$ = +15 º (*c* 0.25, $CHCl_3$); ^{1}H NMR (400 MHz, $CDCl_3$): δ 7.36 – 7.18 (m, 40H), 6.09 (dd, *J* = 28.6, 8.7 Hz, 1H), 5.12 – 4.64 (m, 11H), 4.59 – 4.50 (m, 5H), 4.46 – 4.28 (m, 5H), 4.17 – 4.08 (m, 1H), 3.97 (td, *J* = 9.2, 2.7 Hz, 1H), 3.87 – 3.78 (m, 2H), 3.71 – 3.44 (m, 6H), 3.37 (d, *J* = 9.3 Hz, 1H), 3.30 (s, 3H), 3.27 – 3.20 (m, 1H); ^{13}C NMR (101 MHz, $CDCl_3$): δ 170.0, 169.9, 156.3, 155.8, 139.0, 139.0, 138.8, 138.7, 138.6, 138.4, 138.4, 138.3, 138.3, 138.2, 138.1, 137.9, 137.9, 137.8, 136.3, 136.2-127.7 (96C), 104.1, 99.0, 98.1, 97.9, 84.5, 82.5, 82.2, 82.0, 81.8, 81.6, 80.3, 80.1, 79.8, 78.2, 78.0, 77.9, 76.0, 75.7, 75.5, 75.4, 75.1, 74.7, 73.8, 73.6, 73.4, 73.3, 72.9, 71.0, 70.5, 70.5, 70.3, 69.1, 68.9, 68.7, 68.3, 68.3, 67.3, 67.2, 64.5, 64.4., 55.3, 55.4; IR ($CHCl_3$): 3446, 2937, 1680, 1453, 1268, 1239, 1069, 1005, 895, 847, 749, 695 cm^{-1}; HRMS (*m/z*): $[M+Na]^+$ calcd. for $C_{73}H_{77}O_{15}NNa$, 1230.9519 found 1230.9510.

Methyl 2,3,6-tri-*O*-benzyl-4-*O*-((4*R*)-4-((3*R*,8*R*,9*S*,10*S*,13*R*,14*S*,17*R*)-3-(2,3,4,6-tetra-*O*-benzyl glucopyranosyloxy)-10,13-dimethylhexadecahydro-1H-cyclopenta[a]phenanthren-17-yl)pentanoyl) α-D-glucopyranoside (**19c**):

This compound was synthesized according the one pot glycan editing procedure mentioned above (78% overall yield, thick syrup) (α: β = 3:1)

$[\alpha]_D^{25}$ = +39° (*c* 0.25, $CHCl_3$); ^{1}H NMR (400 MHz, $CDCl_3$): δ 7.40 – 7.25 (m, 35H), 5.01 (d, *J* = 2.6 Hz, 1H), 4.99 – 4.62 (m, 11H), 4.60 (d, *J* = 1.2 Hz, 3H), 4.57 – 4.54 (m, 2H), 4.50 – 4.43 (m, 2H), 4.28 – 4.25 (m, 2H), 4.00 (td, *J* = 9.2, 3.5 Hz, 2H), 3.91 – 3.71 (m, 3H), 3.71 – 3.43 (m, 8H), 3.37 (s, 3H), 2.41 – 1.29 (m, 29H), 0.90 (s, 6H), 0.61 (d, *J* = 2.8 Hz, 3H); ^{13}C NMR (101 MHz, $CDCl_3$) δ 174.0, 174.0, 139.0, 138.7, 138.7, 138.6, 138.6, 138.3, 138.3, 138.3, 138.2, 138.1, 138.1, 138.0, 138.0, 137.9, 128.5-127.6 (84C), 102.4, 98.1, 94.7, 85.0, 84.9, 82.5, 82.3, 82.2, 82.1, 80.3, 80.2, 80.0, 79.9, 78.1, 78.0, 77.6, 76.5, 76.0, 75.9, 75.9, 75.7, 75.1, 75.0, 75.0, 74.9, 73.5, 73.5, 73.4, 70.1, 70.0, 69.3, 69.2, 68.7, 68.5, 68.4, 62.9, 62.8, 56.5, 56.4, 56.0, 55.3, 55.2, 42.9, 42.8, 42.2, 42.1, 40.2, 40.0, 35.9, 35.9, 35.6, 35.3, 34.8, 34.7, 32.3, 32.0, 31.1, 30.9, 29.7, 29.7, 28.2, 27.4, 27.4, 27.2, 26.4 , 24.2, 23.5, 23.3, 20.8, 20.8, 18.3, 18.3, 12.1, 12.1; IR ($CHCl_3$): 3446, 2937, 1755, 1453, 1268, 1239, 1069, 1005, 895, 847, 749, 695 cm^{-1}; HRMS (*m/z*): $[M+Na]^+$ calcd. for $C_{86}H_{104}O_{13}Na$, 1368.7406; found, 1368.7428.

One pot Editing of Mannose trisaccharide by CIStER :

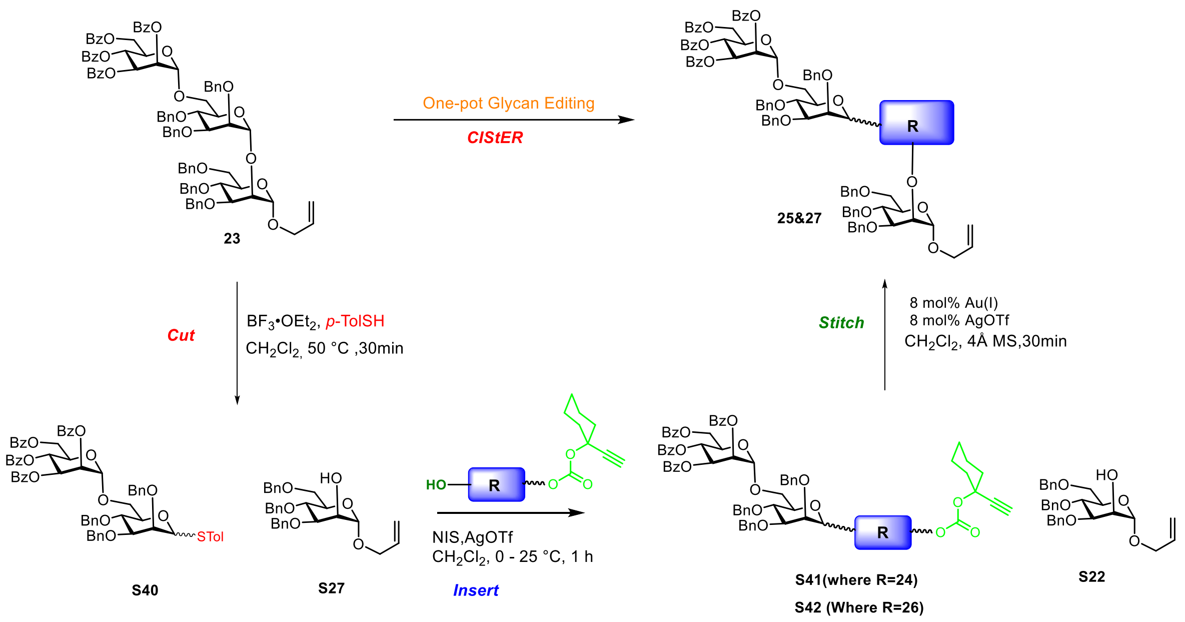

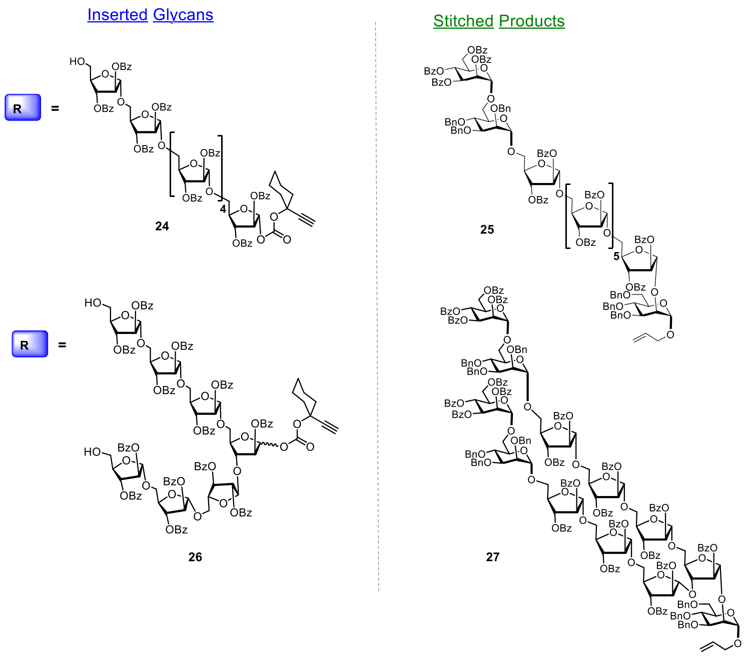

Procedure for one-pot Glycan Editing methodology:

The trisaccharide **23** (300 mg, 0.266 mmol) was dissolved in 3mL of anhydrous CH_2Cl_2. The reaction vessel was cooled to 0 °C and BF_3•OEt_2 (98.7 μL, 0.799 mmol) was added. The reaction mixture was refluxed at 50 °C for 30 min, brought to 25 °C and external glycan acceptor (1 eq. for **24** 0.5 eq. for **26**) [**24** (674 mg,) or **26** (323 mg)] were added to the reaction mixture and the reaction vessel was stirred at 0 °C. To this reaction mixture, freshly activated 4Å MS powder (80 mg) was added at 0 °C under nitrogen atmosphere and kept for vigorous stirring for another 15 min. Then NIS (1.5 eq., 89 mg), AgOTf (0.2 eq., 13 mg) were added simultaneously and stirred for 15 min at 0 °C and kept at 25 °C for another 45 min. After this to this reaction mixture, chloro[tris(2,4 di^{t}butylphenyl)phosphite] gold(I) (0.08 eq., 14 mg), AgOTf (0.1 eq., 5 mg) were added simultaneously to the reaction mixture and stirred for another 30 min. Et_3N was added to arrest the reaction and the solvent was removed under reduced pressure. The crude residue was purified by silica gel column chromatography (30% - 40% ethyl acetate/hexane) to afford the oligosaccharides **25** and **27**.

Supplementary Figure S3: LC Profile of CIStER:

HPLC system: Gilson's PLC 2050 series
Column: Chromasol ONYX DIOL column (5µ, 4.6 mm ×250 mm)
Mobile Phase A: Ethyl Acetaate
Mobile Phase B: n-hexane
Column temp: 25 °C
Sample temp: 25 °C
Flow Rate: 1ml/min
Run time: 60 min
Uv-detection: 270nm
Mobile Phase:

Time (min)	%A	%B
0	20	80
15	20	80
20	30	70
30	30	70
40	40	60
50	100	00
60	100	00

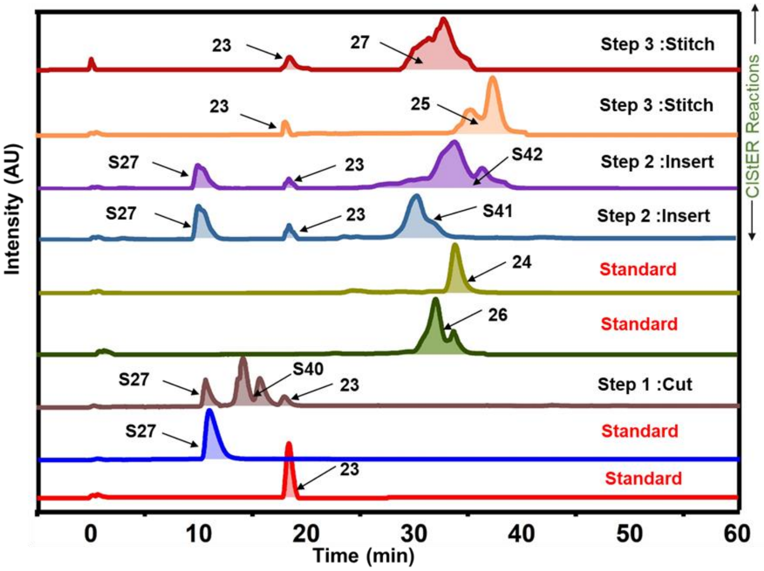

Progress of the one-pot CIStERs was monitored by using semi-preparative HPLC system equipped with a normal phase silica gel diol column. Initially, mobile phase conditions were optimized by injecting the four standards samples **23**, **S27**, **24**, and **26** so that the peaks are well resolved for identification purpose. Subsequently, aliquots of CIStER reaction (**14**,**16**) were injected under above optimized mobile phase conditions. Products **25&27** and intermediates during the CIStER were collected and characterized.

p-Tolyl-2,3,4-tri-*O*-benzyl-6-*O*-(2,3,4,6-tetra-*O*-benzoyl-α-D-mannopyranosyl)-1-thio-α/β-D-mannopyranoside (**S40**):

This compound was synthesized from the trisaccharide **23** (cut step of CIStER) according to the one pot glycan editing methodology mentioned above (85 %yield, white solid) (α: β = 6:1).

mp: 60 °C; $[\alpha]_D^{25}$ = +10° (*c* 0.26, $CHCl_3$); ^1H NMR (400 MHz, $CDCl_3$): δ 8.15 – 8.02 (m, 4H), 7.93 – 7.80 (m, 4H), 7.49 – 7.24 (m, 29H), 7.12 (d, *J* = 7.9 Hz, 2H), 6.16 – 6.06 (m, 1H), 5.91 (dd, *J* = 10.1, 3.3 Hz, 1H), 5.80 (dt, *J* = 5.0, 2.5 Hz, 1H), 5.50 (d, *J* = 1.7 Hz, 1H), 5.18 (d, *J* = 1.8 Hz, 1H), 5.11 – 5.02 (m, 1H), 4.77 – 4.52 (m, 7H), 4.43 – 4.37 (m, 2H), 4.10 – 4.00 (m, 3H), 3.97 – 3.83 (m, 2H), 2.15 (s, 3H); ^{13}C NMR (101 MHz, $CDCl_3$): δ 166.3, 166.3, 165.6, 165.5, 165.5, 165.4, 165.3, 165.2, 138.5, 138.5 , 138.2, 138.2, 138.1, 138.0, 138.0, 137.9, 133.5, 133.5, 133.2, 133.1, 132.4(2C), 130.5, 130.5,

130.2-129.9(32C), 129.7, 129.6, 129.4, 129.4, 129.2, 129.2, 128.7 -127.8(44C), 98.2, 98.1, 88.4, 86.3, 80.3, 78.9, 76.0, 75.4(2C), 75.3(2C), 74.9, 72.7 (2C), 72.4, 72.1(2C), 72.0, 70.5(2C), 70.3, 68.9(2C), 67.5(2C), 67.1, 62.9, 62.8, 21.2, 21.0; IR ($CHCl_3$): 2991, 1729, 1266, 1216, 1109, 1028, 904, 743, 709, 661 cm^{-1}; HRMS (*m/z*): $[M+Na]^+$ calcd. for $C_{68}H_{62}O_{14}S$, 1158.3179; found, 1158.3192.

Allyl-3,4,6-tri-*O*-benzyl-2-*O*-(2, 3 di-O-benzoyl -5-*O*-(2, 3-di-*O*-benzoyl-5-*O*-(2,3-di-*O*-benzoyl-5-*O*-(2,3-di-*O*-benzoyl-5-*O*-(2,3-di-*O*-benzoyl-5-*O*-(2,3-di-*O*-benzoyl-5-*O*-(2,3-di-*O*-benzoyl-5-*O*-(2,3,4-tri-*O*-benzyl-6-*O*-(2,3,4,6-tetra-*O*-benzoyl-α-D-mannopyranosyl)-α/β-D-mannopyranosyl)-α-D-arabinofuranosyl)-α-D-arabinofuranosyl)-α-D-arabinofuranosyl)-α-D-arabinofuranosyl)-α-D-arabinofuranosyl)-α-D-arabinofuranosyl)-α-D-arabinofuranosyl)-α-D- Mannopyranoside (**25**)):

This compound was synthesized according to above mentioned one pot glycan editing procedure (51% overall yield, white solid).

mp: 97 °C; $[\alpha]_D^{25}$ = + 3° (*c* 0.15, $CHCl_3$); ^{1}H NMR (600 MHz, $CDCl_3$): δ 8.15 – 7.75 (m, 36H), 7.57 – 7.14 (m, 84H), 6.11 (t, *J* = 10.1, 3.5 Hz, 1H), 5.97 (t, *J* = 10.2, 6.8, 3.3 Hz, 1H), 5.81 – 5.72 (m, 2H), 5.69 – 5.62 (m, 12H), 5.62 – 5.53 (m, 3H), 5.46 – 5.36 (m, 7H), 5.30 – 5.14 (m, 2H), 5.02 (t, *J* = 5.9 Hz, 2H), 4.86 (d, *J* = 10.8 Hz, 1H), 4.80 – 4.73 (m, 1H), 4.70 – 4.49 (m, 14H), 4.42 – 4.34 (m, 1H), 4.30 (d, *J* = 11.1 Hz, 2H), 4.25 – 4.13 (m, 10H), 4.12 – 4.02 (m, 2H), 4.02 – 3.81 (m, 16H), 3.75 – 3.64 (m, 2H), 3.63 – 3.55 (m, 1H); ^{13}C NMR (151 MHz, $CDCl_3$): δ 166.3-165.1(36C), 138.7, 138.7, 138.7, 138.5, 138.5, 138.5, 138.5, 138.5, 138.4, 138.4, 138.2, 138.1, 133.9(2C), 133.6 -133.0(40C), 130.1 - 129.8(80C), 129.7-129.2(36C), 128.6 - 128.1(80C), 128.0 - 127.5(40C), 117.3(2C), 108.5, 106.8, 106.2, 106.0-105.9(12C), 101.9, 98.5, 98.5, 98.0, 97.8, 82.7- 81.6(42C), 80.6, 80.1, 79.8, 78.0, 77.8, 75.3, 75.2(2C), 75.1, 75.0, 74.8(2C), 74.7(2C), 74.5, 74.2(2C), 73.9(2C), 73.6, 73.3, 72.7(2C), 72.2, 71.9, 71.9(2C), 71.7, 71.1, 70.6, 70.5, 70.2, 70.1, 69.6(2C), 68.9, 68.9, 68.0(2C), 67.0(2C), 66.9, 66.0(14C), 62.8(2C); IR ($CHCl_3$): 2918, 1722, 1452, 1264, 1109, 1026, 708 cm^{-1}; HRMS (*m/z*): $[M+Na]^+$ calcd. for $C_{224}H_{200}O_{62}Na$, 3905.8243; found, 3905.8248.

Allyl-3,4,6-tri-*O*-benzyl-2-*O*- (3, 5-di-*O*-(2, 3-di-*O*-benzoyl-5-*O*-(2,3-di-*O*-benzoyl-5-*O*-(2,3-di-*O*-benzoyl-5-*O*-(2,3,4,-tetra-*O*-benzyl-6-*O*-(2,3,4,6-tetra-*O*-benzoyl-α-D-mannopyranosyl)-α/β-D-mannopyranosyl)-α-D-arabinofuranosyl)-α-D-arabinofuranosyl)-α-D-arabinofuranosyl)-α-D-Mannopyranoside (**27**):

This compound was synthesized according to above mentioned one pot glycan editing procedure. (45% overall yield, white solid).

mp: 100 °C; $[\alpha]_D^{25}$ = -5° (*c* 0.11, $CHCl_3$); ^1H NMR (600 MHz, $CDCl_3$): δ 8.14 – 7.80 (m, 44H), 7.61 – 7.11 (m, 111H), 6.10 (t, *J* = 10.2 Hz, 2H), 5.97 (d, *J* = 9.4 Hz, 2H), 5.77 (d, *J* = 18.9 Hz, 2H), 5.71 (s, 1H), 5.69 – 5.62 (m, 6H), 5.61 – 5.53 (m, 5H), 5.49 (dd, *J* = 15.0, 6.1 Hz, 2H), 5.40 (q, *J* = 9.4, 8.6 Hz, 3H), 5.30 (d, *J* = 8.9 Hz, 1H), 5.27 (d, *J* = 10.5 Hz, 1H), 5.21 (d, *J* = 5.2 Hz, 1H), 5.14 (d, *J* = 9.3 Hz, 1H), 5.04 – 5.00 (m, 3H), 4.97 – 4.91 (m, 1H), 4.82 (d, *J* = 10.7 Hz, 1H), 4.74 (dd, *J* = 23.4, 10.9 Hz, 4H), 4.68 – 4.40 (m, 20H), 4.36 (dd, *J* = 11.8, 7.8 Hz, 3H), 4.31 – 4.13 (m, 11H), 4.11 – 4.02 (m, 5H), 3.98 (m, 5H), 3.93 – 3.82 (m, 13H), 3.76 (d, *J* = 4.0 Hz, 1H), 3.63 – 3.55 (m, 1H); ^{13}C NMR (151 MHz, $CDCl_3$): δ 166.3 (8C), 165.7 (8C), 165.6 (8C), 165.5,165.4, 165.3 (8C), 165.1 (8C), 138.7, 138.7, 138.6, 138.5, 138.5 (4C), 138.5 (4C), 138.4, 138.3, 138.1 (4C), 134.1, 134.0, 133.5, 133.5-133.0 (20C), 130.0-129.8 (120C), 129.7- 129.1 (44C), 128.6 -128.3 (120C), 128.0 -127.4 (50C), 117.3, 117.2, 106.2-106.0 (10C), 105.3, 105.1, 101.9 (2C), 98.8, 98.6, 98.5, 98.0, 97.8, 97.8, 82.7-81.4 (24C), 80.6-80.1 (12C), 78.1, 78.1, 78.0, 77.8, 75.3-75.0 (14C), 74.7-74.5 (16C), 74.2 (2C), 73.9, 73.5, 73.3 (4C), 73.3, 72.7 (4C), 72.3 - 71.7 (8C), 71.1, 70.6 ,70.5 , 70.0, 69.7 (2C), 69.3 (2C), 68.9, 68.9, 67.9 (4C), 67.1, 67.0, 66.9, 66.8, 66.7 (4C), 65.9-65.5 (12C), 62.8, 62.8; IR ($CHCl_3$): 2925, 1722, 1452, 1263, 1107, 1027, 709 cm^{-1}; HRMS (*m/z*): $[M+Na]^+$ calcd. for $C_{278}H_{250}O_{75}Na$, 4813.2147 found 4813.2145.

1-*O*-(((1-ethynylcyclohexyl)oxy)carbonyl)- 2,3,4 -tri-*O*-acetyl-α/β-D-glucopyranoside **(21):**

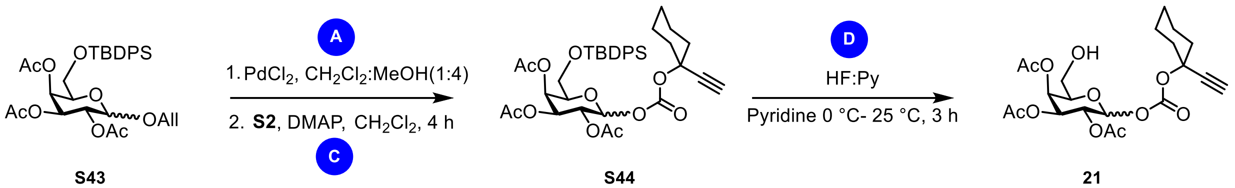

S43 **S44** **21**

Compound **S43** [39] was converted to carbonate donor **S44** according to general experimental procedure A, C. Next, the compound **S44** was subjected to the experimental procedure D to obtain the compound **21** (yield 92%, thick syrup)(α:β=2.5:1).

$[\alpha]_D^{25}$ = + 69° (*c* 0.25, $CHCl_3$); ^1H NMR (400 MHz, $CDCl_3$): δ 5.62 (d, *J* = 8.2 Hz, 1H), 5.12 (dd, *J* = 10.4, 3.4 Hz, 1H), 4.42 – 4.16 (m, 1H), 3.94 – 3.89 (m, 1H), 3.75 (dd, *J* = 11.7, 6.6 Hz, 1H), 3.55 (dd, *J* = 11.7, 6.4 Hz, 1H), 2.67 (s, 1H), 2.17 (s, 4H), 2.14 – 2.10 (m, 2H), 2.07 (s, 4H), 2.01 (s, 3H), 1.92 – 1.56 (m, 8H); ^{13}C NMR (101 MHz, $CDCl_3$) δ 171.0, 170.9, 170.0, 170.0, 169.5, 169.4, 151.0, 150.9, 95.3, 92.9, 82.3, 78.9, 78.7, 75.4, 75.3, 74.5, 73.1, 71.8, 70.9, 70.4, 68.1, 67.5, 66.7, 66.4, 60.7, 60.4, 36.8, 36.7, 36.6, 36.6, 24.9, 24.9, 24.8, 24.7, 22.5, 22.4, 20.8, 20.8, 20.7, 20.6, 20.6, 20.6; IR ($CHCl_3$):

3446, 2937, 1755, 1453, 1268, 1239, 1069, 1005, 895, 847, 749, 695 cm^{-1}; HRMS (*m/z*): $[M+Na]^+$ calcd. for $C_{21}H_{28}O_{11}Na$, 479.1529; found, 475.1524.

Gram scale One-pot synthesis of 6′-galactosyllactose:

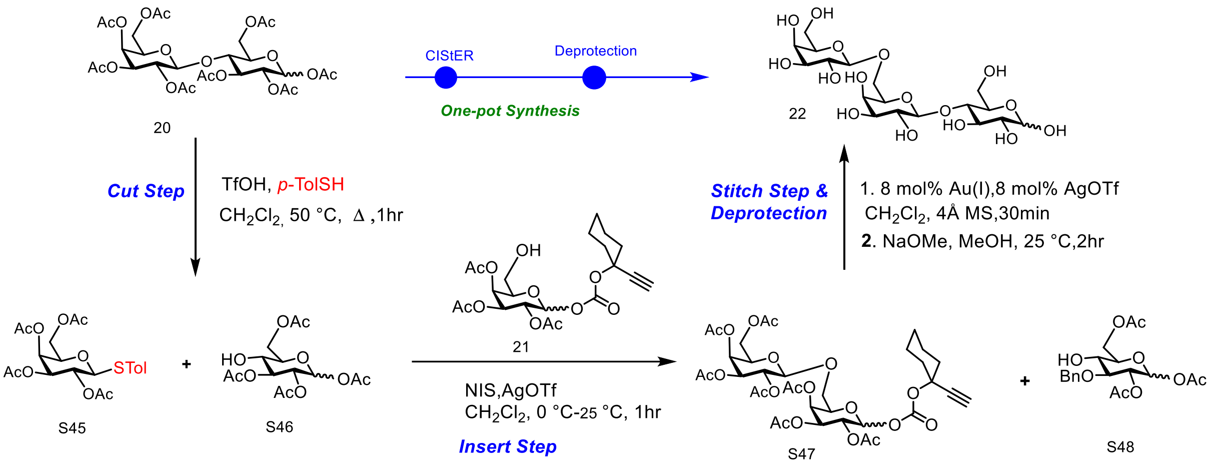

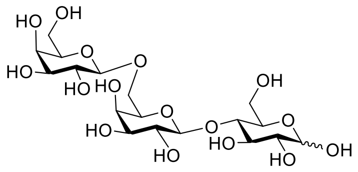

β- D -galactopyranosyl-(1 ⟶ 6)-β-D -galactopyranosyl-(1 ⟶ 4)- α /β D –glucopyranose **(22)**:

To a solution of compound **20** (1eq, 3gm) in 15 mL of anhydrous CH_2Cl_2, TfOH (3 eq, 390 μL) was added at 0 °C. After 10 min, reaction mixture was refluxed at 50 °C for 1hr. The reaction mixture was brought to 25 °C, 1 equivalent of compounds **21** (1.7gm) were added to the reaction mixture and cooled to 0 °C. Freshly activated 4Å MS powder (100mg) was added at 0 °C under nitrogen atmosphere and kept for vigorous stirring for another 15 min. Then NIS (1.5 eq, 1.22 gm), AgOTf (0.25 eq, 232 mg) were added simultaneously. The reaction mixture was stirred for 15 min at 0 °C and gradually warmed to 25 °C and stirred for 45 min. After this to this reaction mixture chloro[tris(2,4-di^{t}ertbutylphenyl)phosphite] gold(I) (0.08 eq, 223mg) AgOTf (0.08eqv 65mg,) were added simultaneously to the reaction mixture and stirred for another 30 min. After this to this solution 15 ml of anhydrous MeOH was added and then NaOMe (3.3 eq, 1.34gm) was added and kept for stirring at 25 °C for 2hr. After completion

(1 h) water (5 mL), Amberlite 120 H+ (4.5 g) were added and the reaction mixture was stirred for 30 min. Then it was concentrated in *vacuum*, co-evaporated with toluene, and lyophilized to yield 6′-galactosyllactose. (white foam, 1.4gm ,71% over 4 steps, α: β = 1:3)

mp: 185 °C; $[\alpha]_D^{25}$ = + 16 º (*c* 0.23, H_2O); ^{1}H NMR (600 MHz, D_2O): δ 5.16 (d, *J* = 3.7 Hz, 1H),4.59 (d, *J* = 7.9 Hz, 1H), 4.44 – 4.34 (m, 4H), 4.19 – 4.03 (m, 2H), 4.01 – 3.29 (m, 37H), 3.23 – 3.12 (m, 1H); ^{13}C NMR (151 MHz, D_2O): δ 103.3 (2C), 103.3, 103.2, 96.0, 92.1, 81.0, 75.6, 75.1, 74.9, 74.0, 73.9, 73.9, 72.7, 72.7, 72.5, 72.5, 71.4, 70.8, 70.7, 70.7, 70.4, 69.5, 69.5, 68.9, 68.9, 68.9, 68.7, 68.6, 67.3, 61.0; IR ($CHCl_3$): 3446, 2937, 1453, 1268, 1239, 1069, 1005, 895, 847, 749 cm^{-1}; HRMS (m/z): $[M+Na]^+$ calcd. for $C_{18}H_{32}NaO_{16}$, 527.1588; found, 527.1583.

4. Supplementary References:

[23] Panchadhayee, R.; Misra, A.K. *N*-Bromosuccinimide Mediated Conversion of Allyl Glycosides to Glycosyl Hemiacetals. *Journal of Carbohydrate Chemistry*, **29**, 76 -83 (2010).

[24] Liu, G., Zhang, X., Xing, G. A General Method for N-Glycosylation of Nucleobases Promoted by (p-Tol)$_2$SO/Tf$_2$O with Thioglycoside as Donor. *Chem. Commun*., **51**, 12803-12806 (2015).

[25] Mishra, B., Neralkar, M. and Hotha, S., Stable Alkynyl Glycosyl Carbonates: Catalytic Anomeric Activation and Synthesis of a Tridecasaccharide Reminiscent of *Mycobacterium tuberculosis* Cell Wall Lipoarabinomannan. *Angew. Chem., Int. Ed.*, **55,** 7786 -7791 (2016).

[26] Huang, X.; Huang, L.; Wang, H.; Ye, X.-S. Iterative One-Pot Synthesis of Oligosaccharides. *Angew. Chem., Int. Ed*, **43**, 5221-5224 (2004).

[27] Hartmann, M.; Betz, P.; Sun, Y.; Gorb, S. N.; Lindhorst, T. K.; Krueger, A. Saccharide-Modified Nanodiamond Conjugates for the Efficient Detection and Removal of Pathogenic Bacteria *Chem. Eur. J* ,**18**, 6485 – 6492 (2012).

[28] X. Chen, P. Xu, Y. Xu, L. Liu, Y. Liu, D. Zhu, P. Lei. Synthesis and antibacterial activity of novel modified 5-O-desosamine ketolides. *Bioorg. Med. Chem. Lett*, **22**, 7402–7405 (2012).

[29] Pasari, S., Manmode, S., Walke, G., and Hotha, S., A Versatile Synthesis of Pentacosafuranoside Subunit Reminiscent of Mycobacterial Arabinogalactan Employing One Strategic Glycosidation Protocol. *Chem. Eur. J.*, **24**, 1128 -1139 (2018).

[30] Islam,M., Shinde, G. P., and Hotha, S. Expedient synthesis of the heneicosasaccharyl mannose capped arabinomannan of the *Mycobacterium tuberculosis* cellular envelope by glycosyl carbonate donors. *Chem. Sci.*, **8**, 2033-2038 (2017).

[31] Xu, W., Springfield, S.A., Koh, J.T. Highly efficient synthesis of 1-thioglycosides in solution and solid phase using iminophosphorane bases. *Carbohydrate Research*, **325**, 169 – 176 (2000).

[32] Wennekes,T., Van den Berg, R J. B. H. N., Donker, W., Van der Marel, G.A., Strijland, A., Aerts, J. M. F. G. ,and Overkleeft, H. S. Development of Adamantan-1-yl-methoxy-Functionalized 1-Deoxynojirimycin Derivatives as Selective Inhibitors of Glucosylceramide Metabolism in Man. *J. Org. Chem.*, **72**, 1088-1097 (2007).

[33] Yu, Y., Gim, S., Kim, D., Arnon, Z. A., Gazit, E., Seeberger, P. H., and Delbianco, M., Oligosaccharides Self-Assemble and Show Intrinsic Optical Properties. *J. Am. Chem. Soc*., **141**, 4833-4838 (2019).

[34] Jalsa, N.K., Regioselective removal of the anomeric O-benzyl from differentially protected carbohydrates. *TetrahedronLetters*, **52**, 6587-6590 (2011).

[35] Cribiù, R., Borbas, K. E., and Cumpstey, I., On the synthesis of vinyl and phenyl C-furanosides by stereospecific debenzylative cycloetherification *Tetrahedron*, **65**, 2022-2031 (2009).

[36] Yamanoi, T., Misawa, N., Matsuda, S., and Watanabe, M., Preparation of partially benzylated mono-, di-, and trisaccharides by selective cleavage of the β-fructofuranosidic linkage in fully benzylated sucrose and sucrose-related oligosaccharides under acidic conditions. *Carbohydrate Research*, **343**, 1366-1372 (2008).

[37] Chang, C.W., Lin, M.H., Chan, C.K., Su, K .Y., Wu, C.H., Lo, W.C., Lam, S., Cheng, Y.T., Liao, P.H., Wong, C.H., and Wang, C.C., Automated Quantification of Hydroxyl Reactivities: Prediction of Glycosylation Reactions *Angew.Chem.Int.Ed*., **60,**12413-12423 (2021).

[38] Tambie, M. S., Jalsa, N. K., A Novel Selectfluor-Mediated Regioselective O-Benzyl Ether Acetolysis of Perbenzylated Monosaccharides. *Journal of Carbohydrate Chemistry*, **34**, 545-559 (2015).

[39] Miura, T., Goto, K., Waragai, H., Matsumoto, H., Hirose, Y., Ohmae, M., Ishida, H., Satoh, A. and Inazu, T., Rapid Oligosaccharide Synthesis Using a Fluorous Protective Group *J. Org. Chem.*, **69**, 5348-5353 (2004).
