# Supplementary material for: Cut-insert-stitch editing reaction (CIStER) sequence for surgical chemical glycan editing
Source: Commun Chem. 2024 Apr 2;7:73. doi: 10.1038/s42004-024-01152-z (PMC10987650; doi:10.1038/s42004-024-01152-z)
Supplement: Supplementary file 2 — Description of Additional Supplementary Files [file 42004_2024_1152_MOESM2_ESM.pdf]

Description of Additional Supplementary Files

File name: Supplementary Data 1

Description: NMR Spectra of all new compounds

File name: Supplementary Data 2

Description: MALDI-ToF Spectra of Representative Compounds
