# Supplementary material for: Cut-insert-stitch editing reaction (CIStER) sequence for surgical chemical glycan editing
Source: Commun Chem. 2024 Apr 2;7:73. doi: 10.1038/s42004-024-01152-z (PMC10987650; doi:10.1038/s42004-024-01152-z)

Supplementary Materials for

Cut-Insert-Stitch Editing Reaction (CIStER) Sequence for Surgical Chemical Glycan Editing

Sumit Sen, Suman Kundu, Sandip Pasari and Srinivas Hotha*

Department of Chemistry, Indian Institute of Science Education and Research Pune

Pune – 411 008, India

s.hotha@iiserpune.ac.in

NMR Spectra of compounds

Supplementary Figure S4a. ^{1}H NMR Spectrum (400 MHz, $CDCl_3$) of compound **2a**

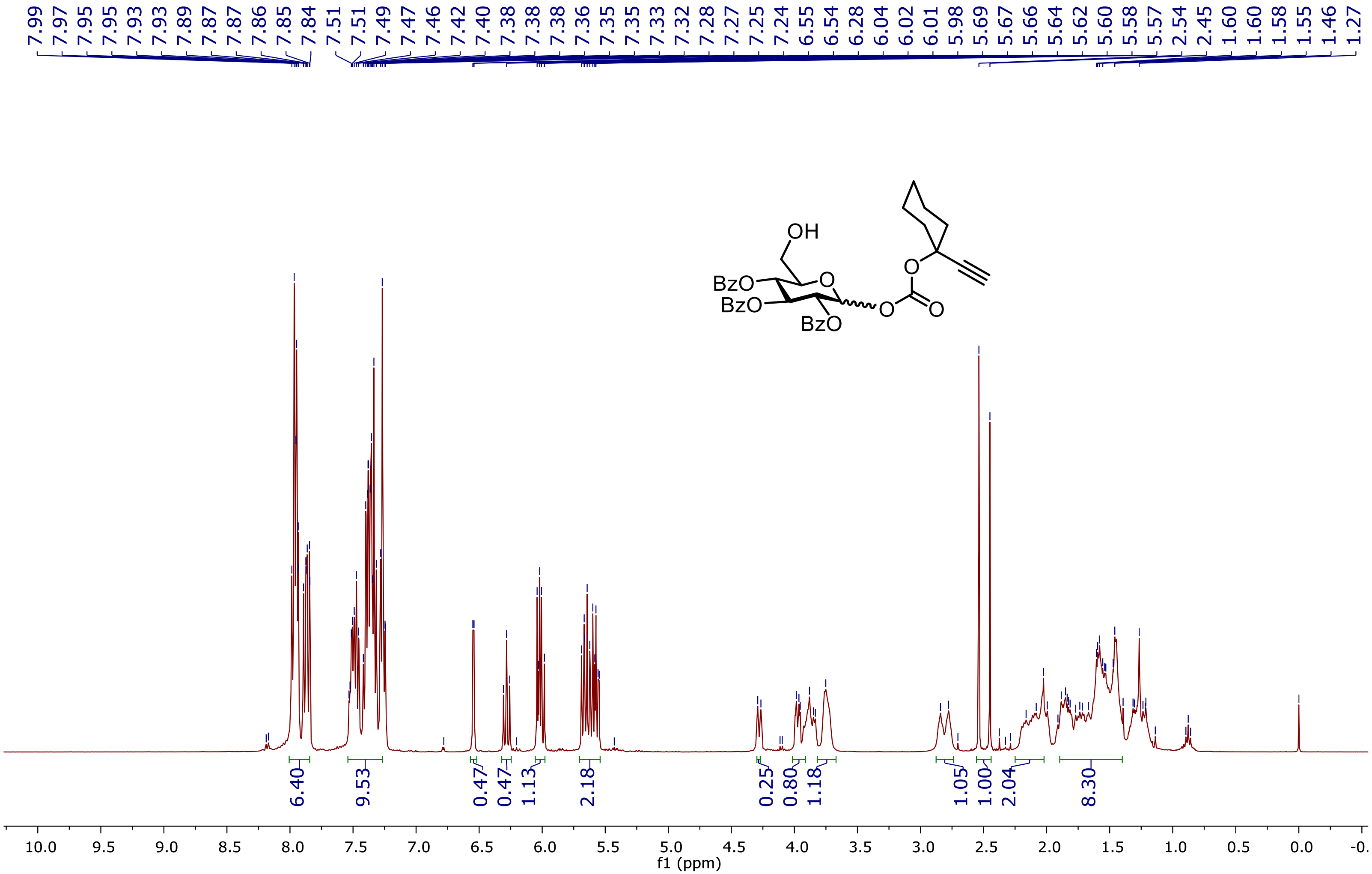

Supplementary Figure S4b. ^{13}C NMR Spectrum (101 MHz, $CDCl_3$) of compound **2a**

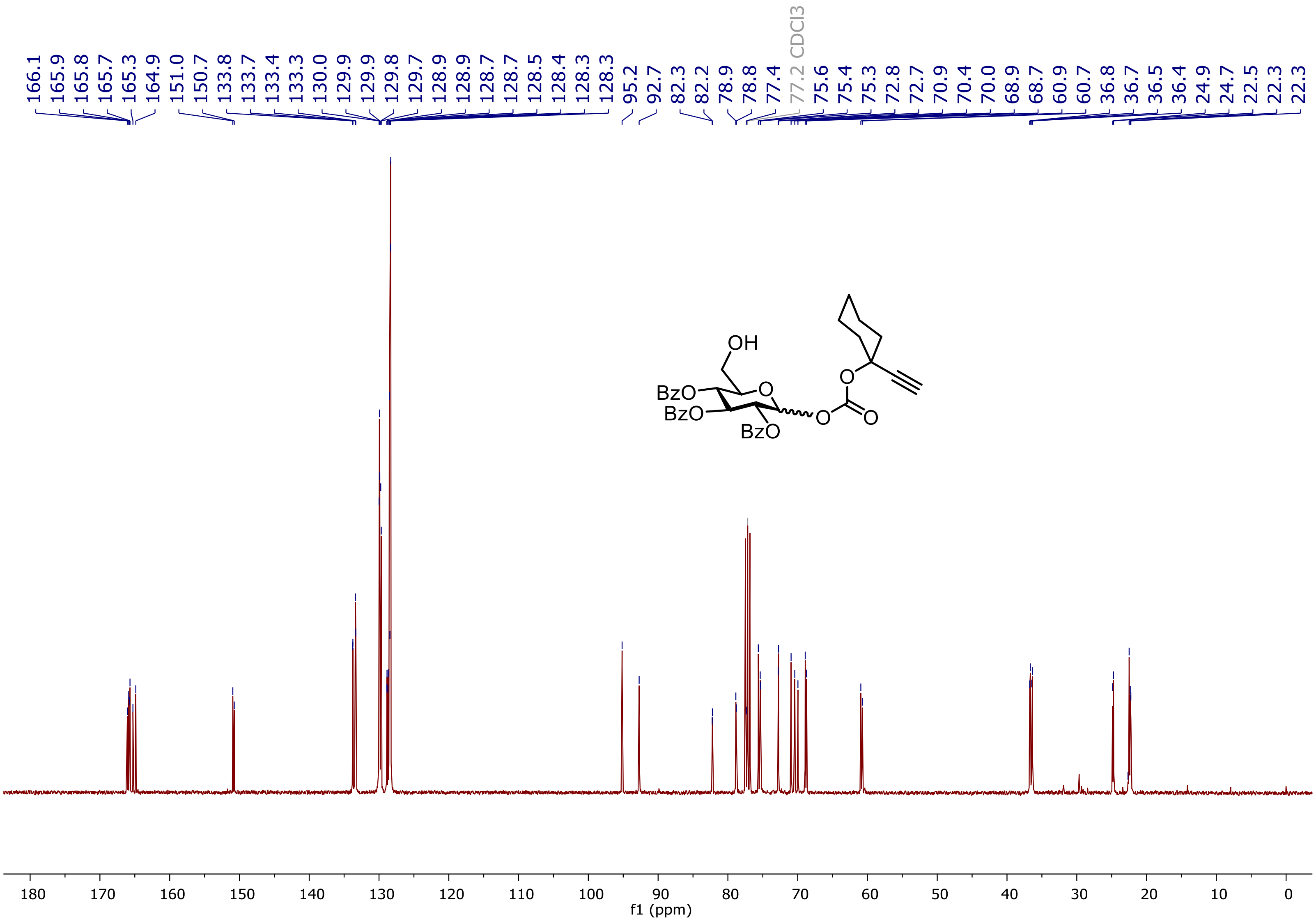

Supplementary Figure S4c. DEPT NMR Spectrum (101 MHz, $CDCl_3$) of compound **2a**

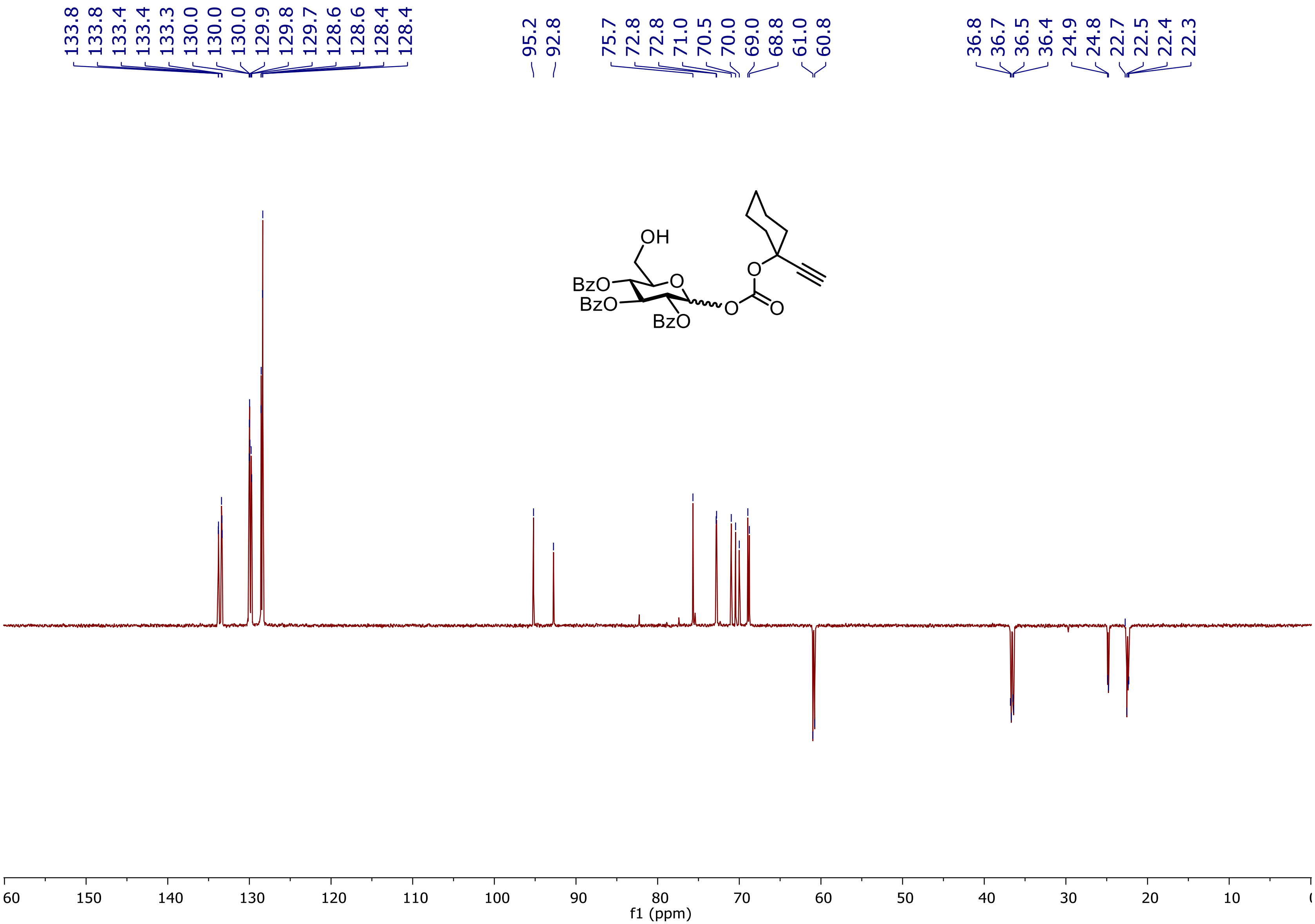

Supplementary Figure S5a. ^{1}H NMR Spectrum (400 MHz, $CDCl_3$) of compound **3a**

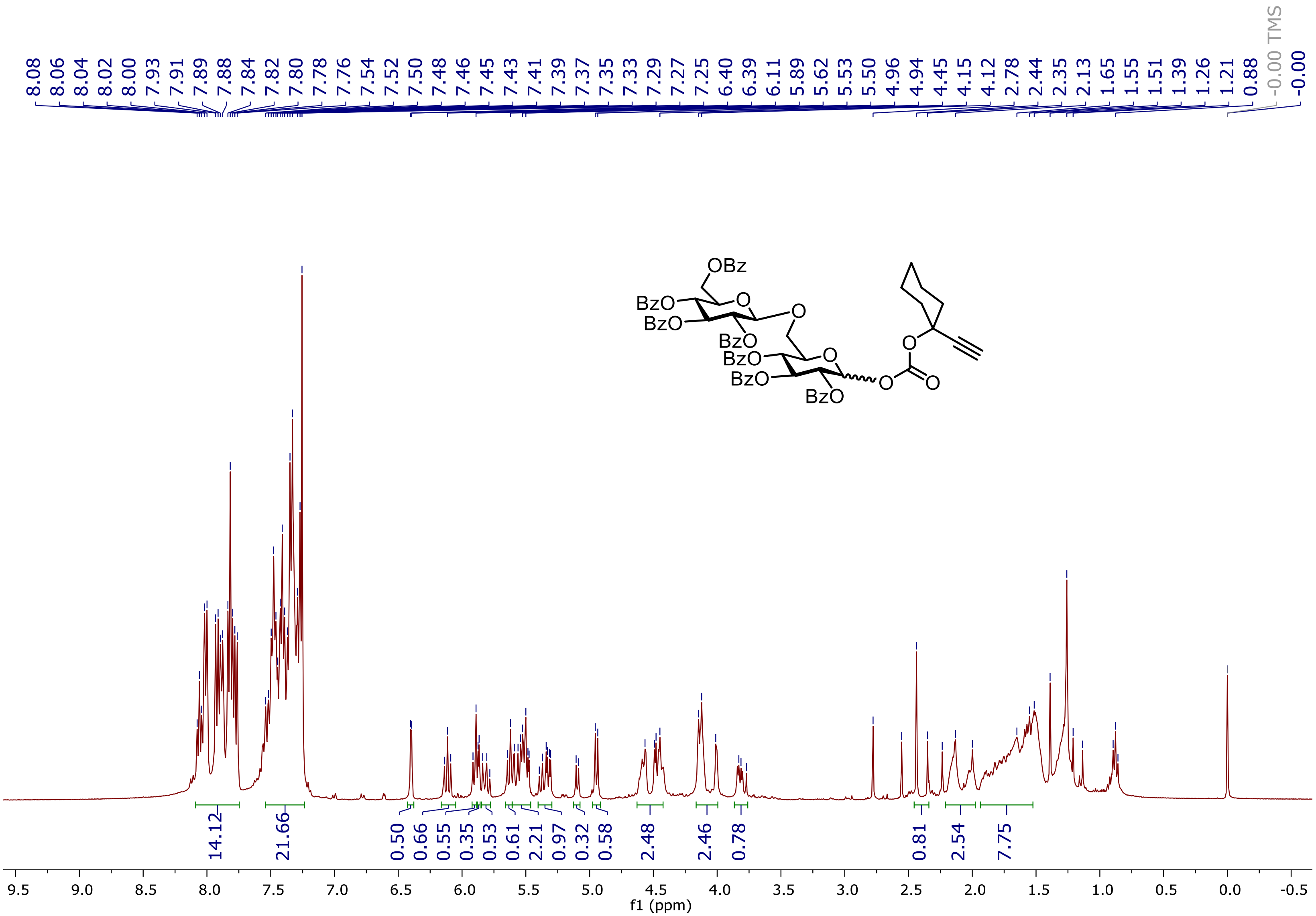

Supplementary Figure S5b. ^{13}C NMR Spectrum (101 MHz, $CDCl_3$) of compound **3a**

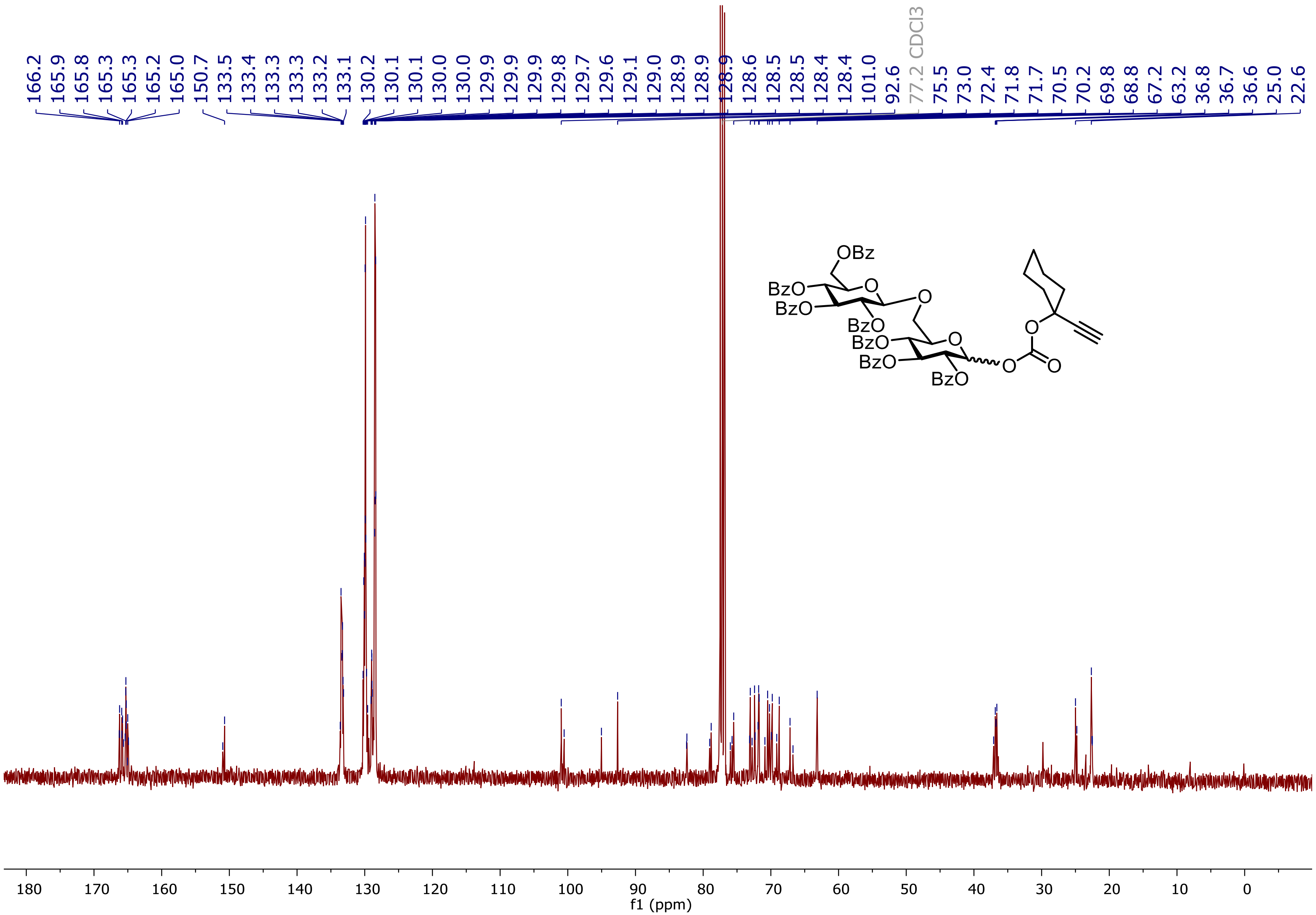

Supplementary Figure S5c. DEPT NMR Spectrum (101 MHz, $CDCl_3$) of compound **3a**

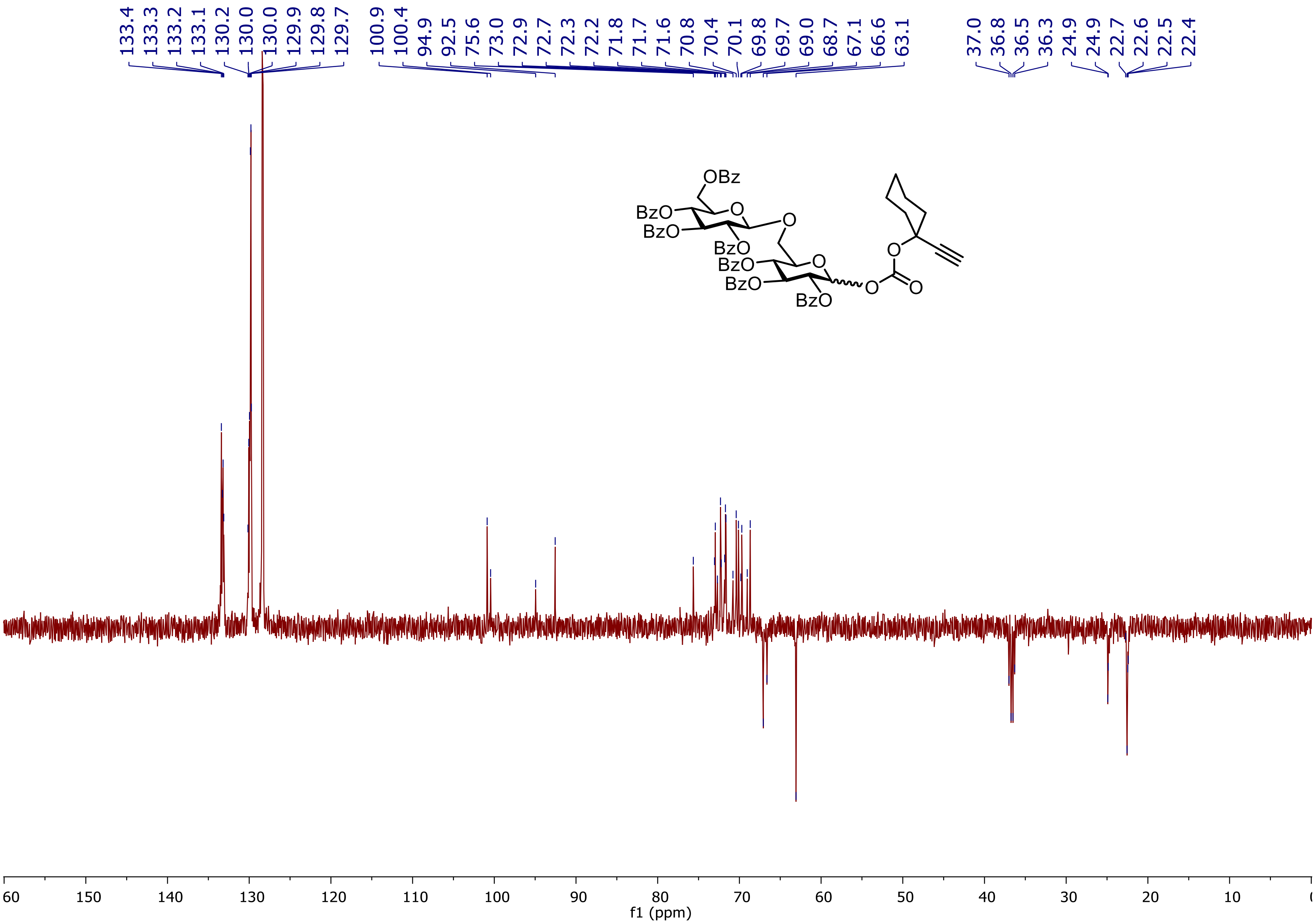

Supplementary Figure S6a. ^{1}H NMR Spectrum (400 MHz, $CDCl_3$) of compound **3b**

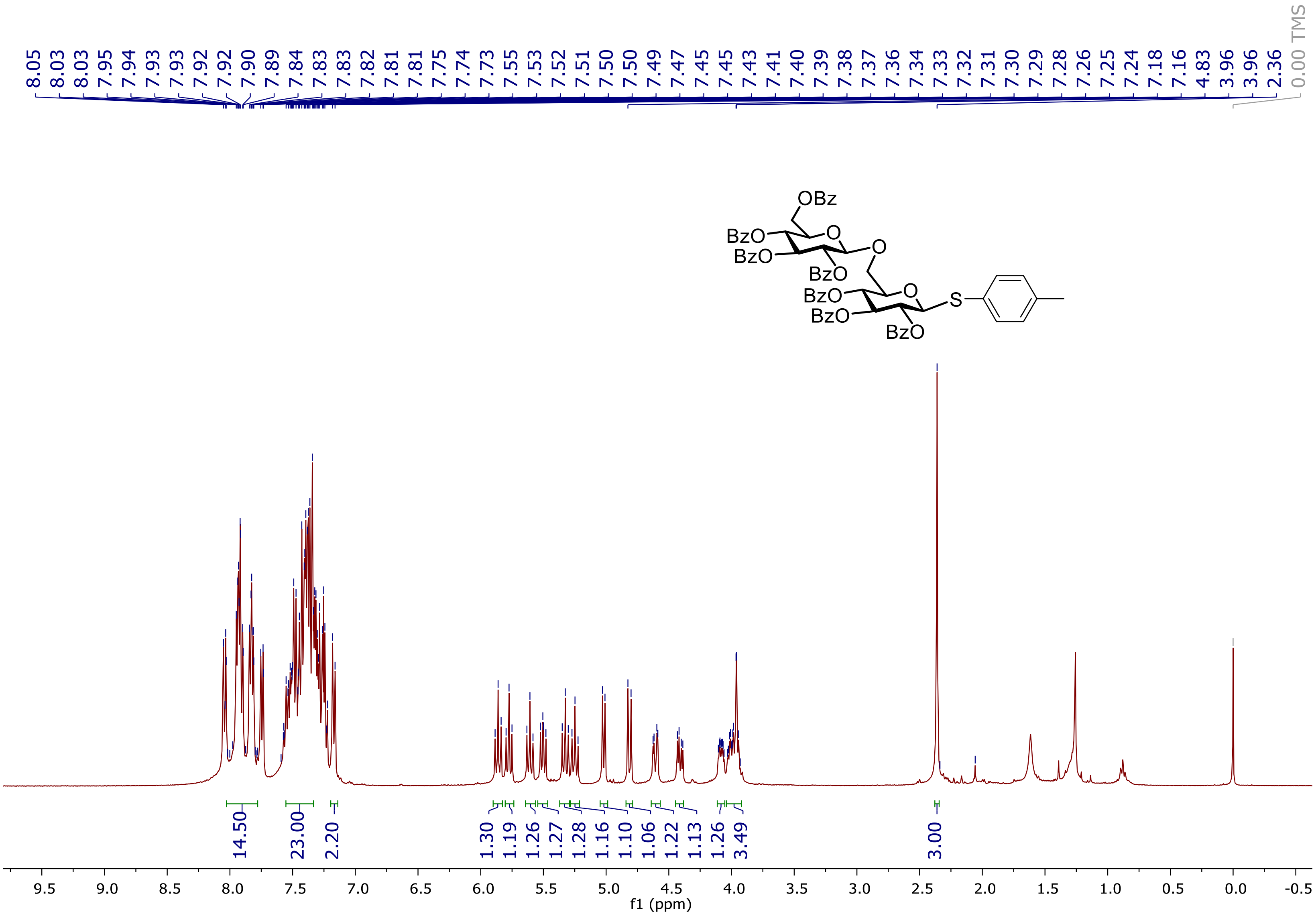

Supplementary Figure S6b. ^{13}C NMR Spectrum (101 MHz, $CDCl_3$) of compound **3b**

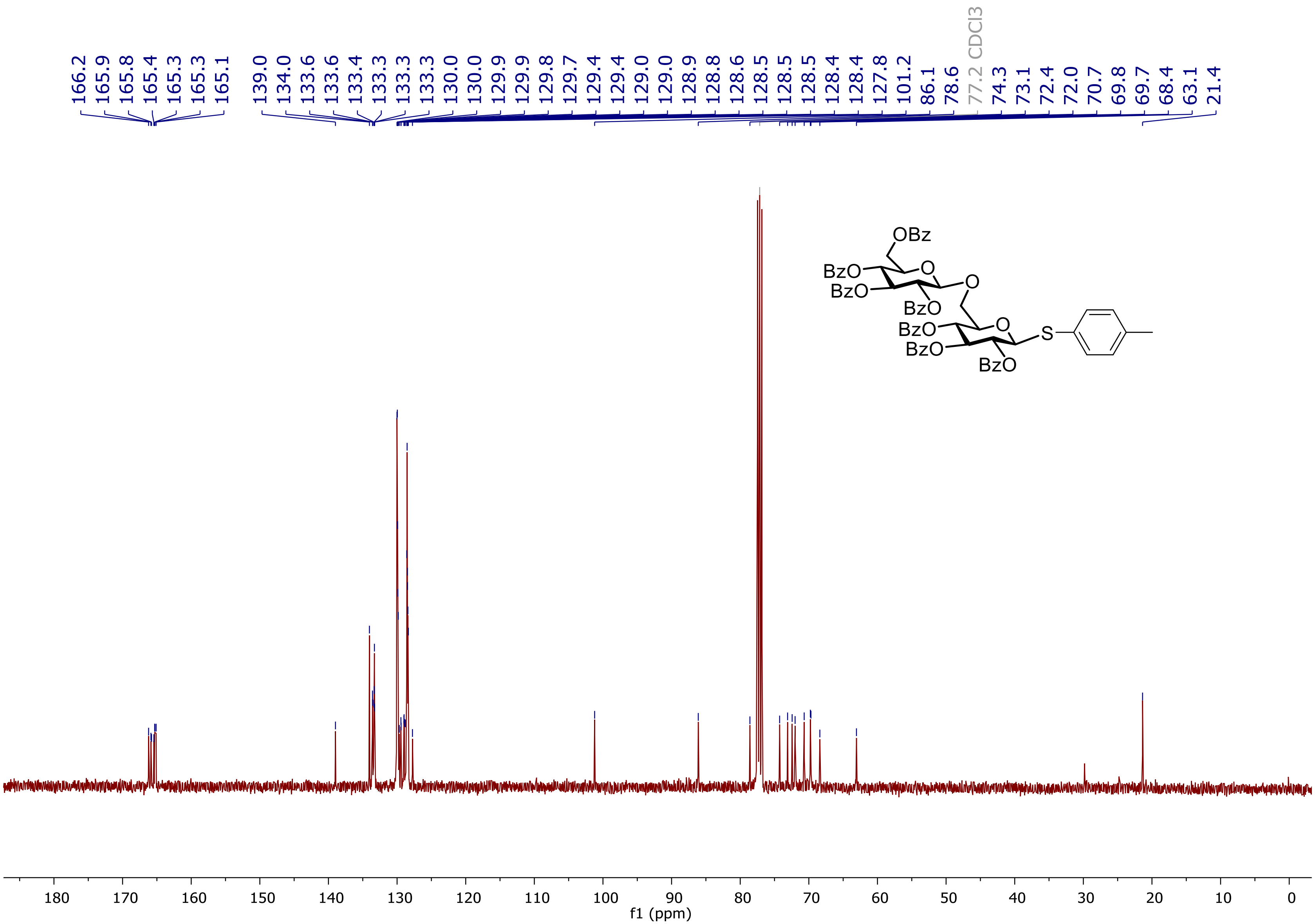

Supplementary Figure S6c. DEPT NMR Spectrum (101 MHz, $CDCl_3$) of compound **3b**

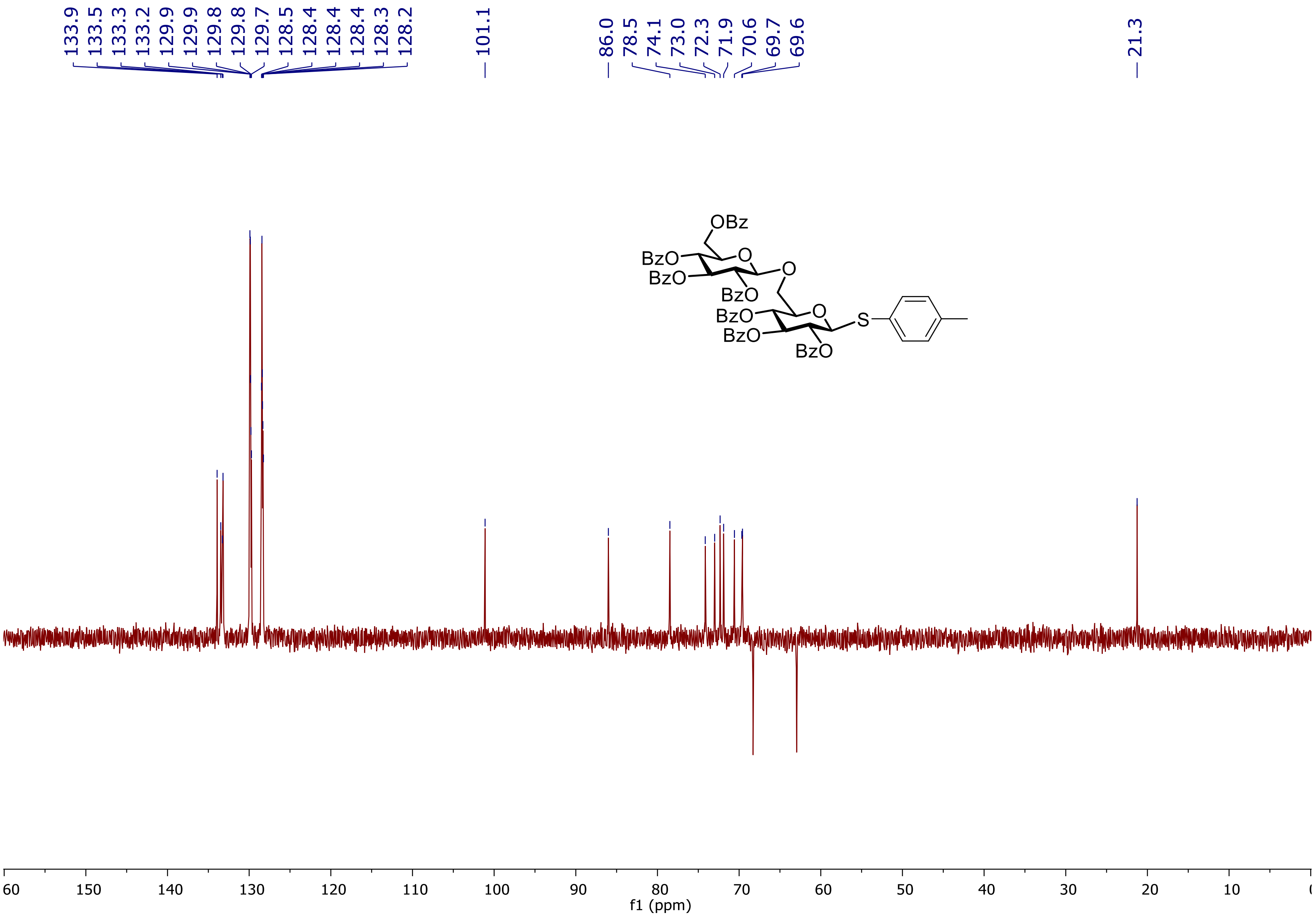

Supplementary Figure S7a. ^{1}H NMR Spectrum (400 MHz, $CDCl_3$) of compound **S7**

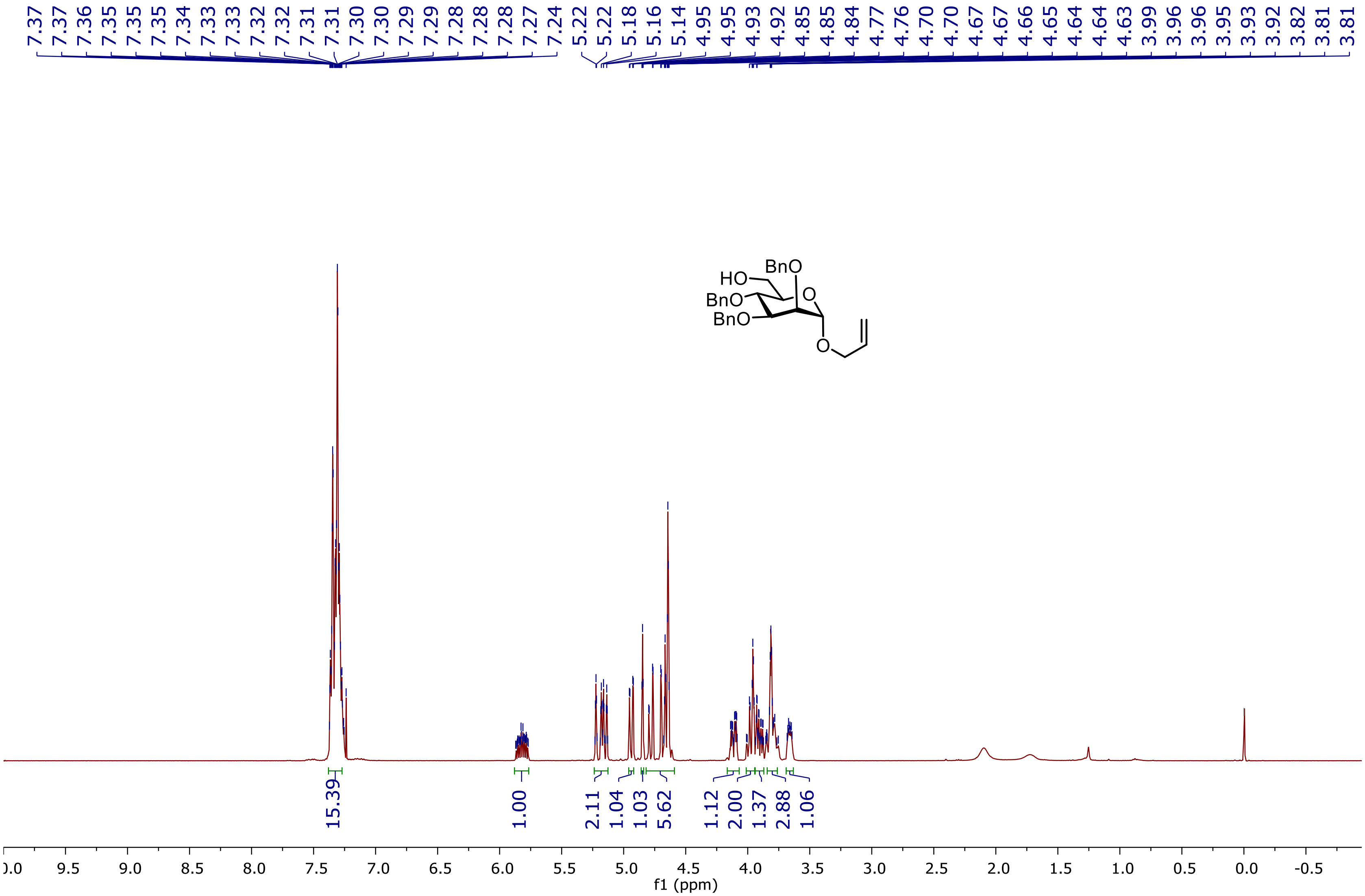

Supplementary Figure S7b. ^{13}C NMR Spectrum (101 MHz, $CDCl_3$) of compound **S7**

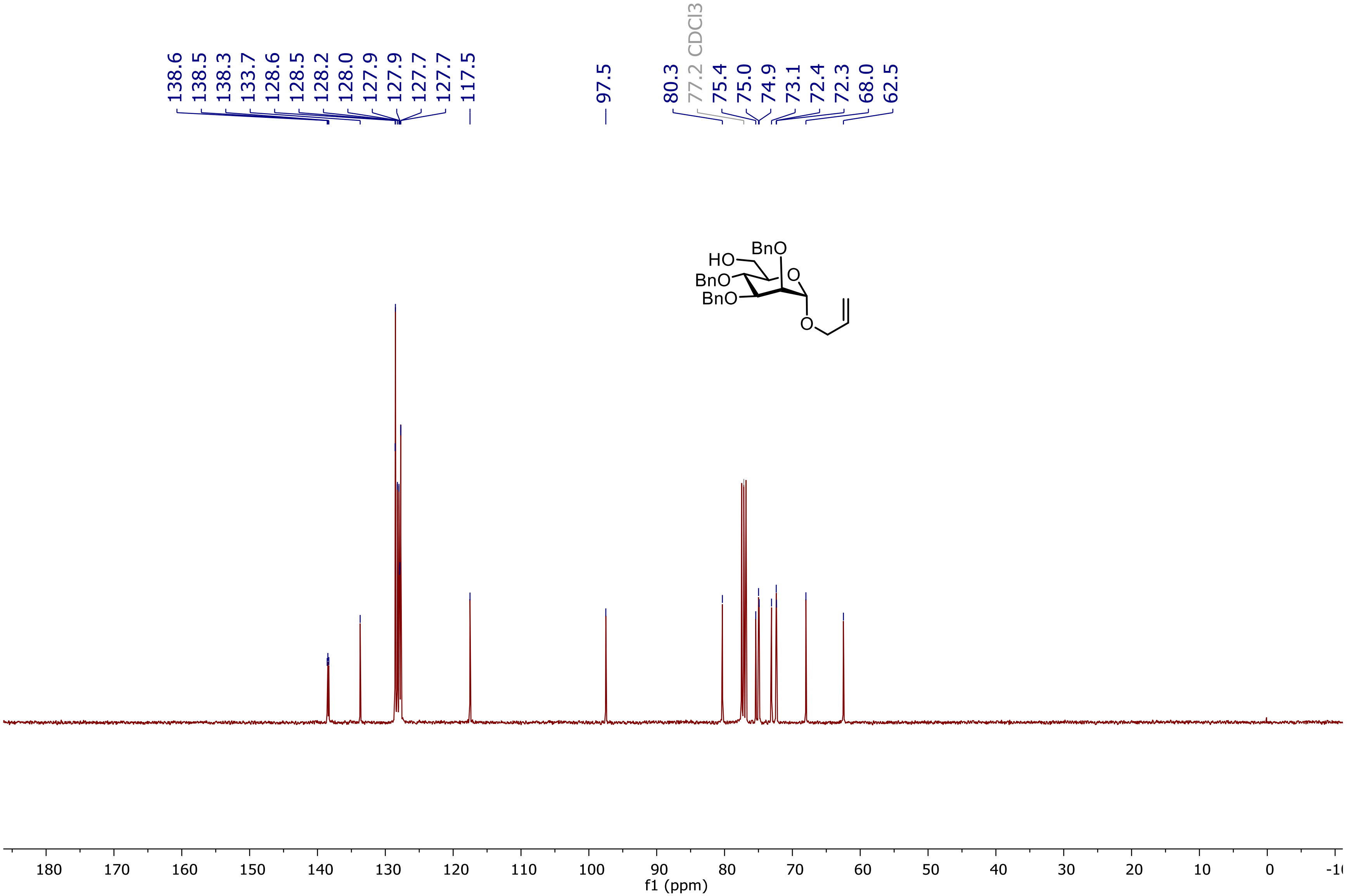

Supplementary Figure S7c. DEPT NMR Spectrum (101 MHz, $CDCl_3$) of compound **S7**

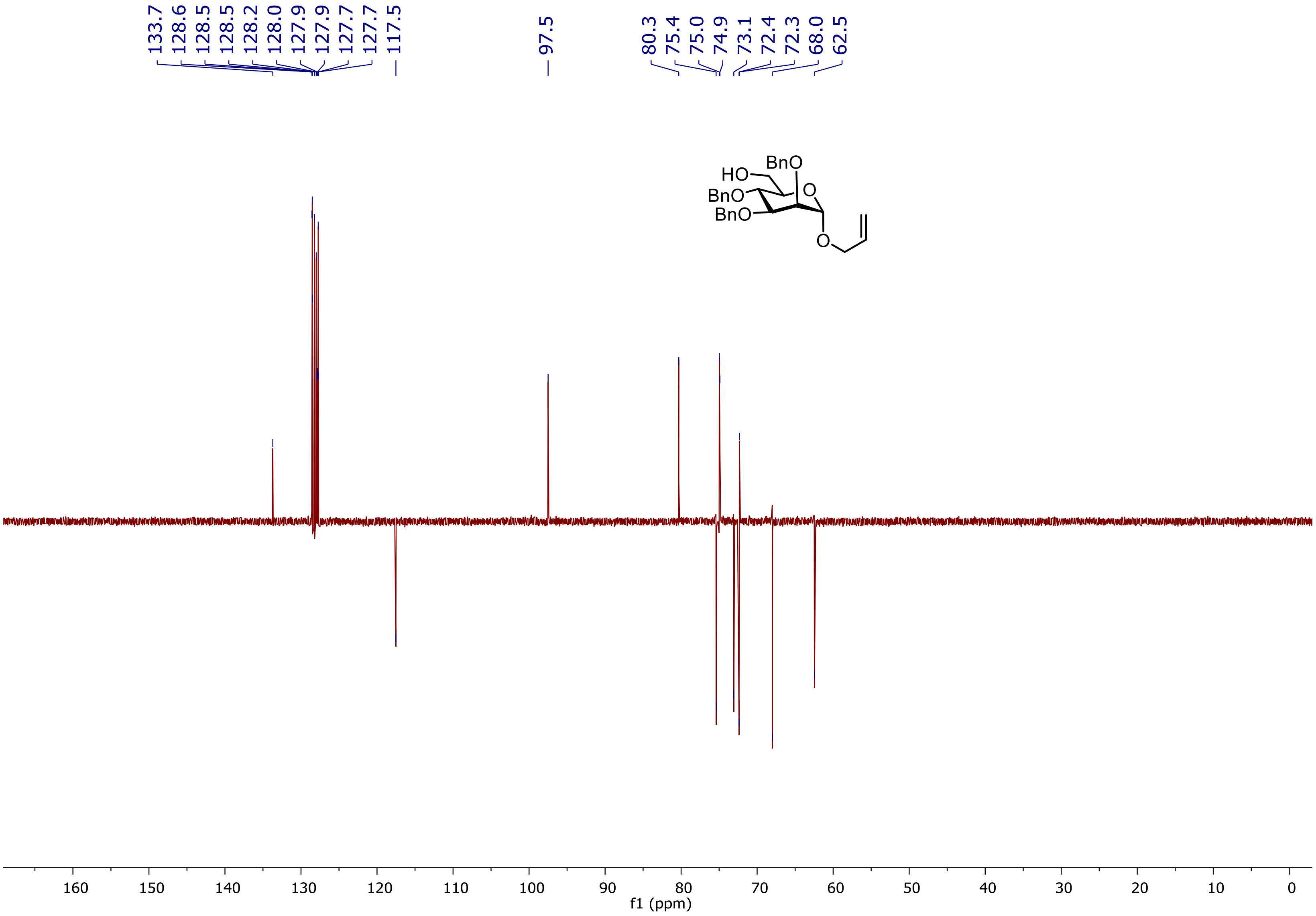

Supplementary Figure S8a. ^{1}H NMR Spectrum (400 MHz, $CDCl_3$) of compound **7**

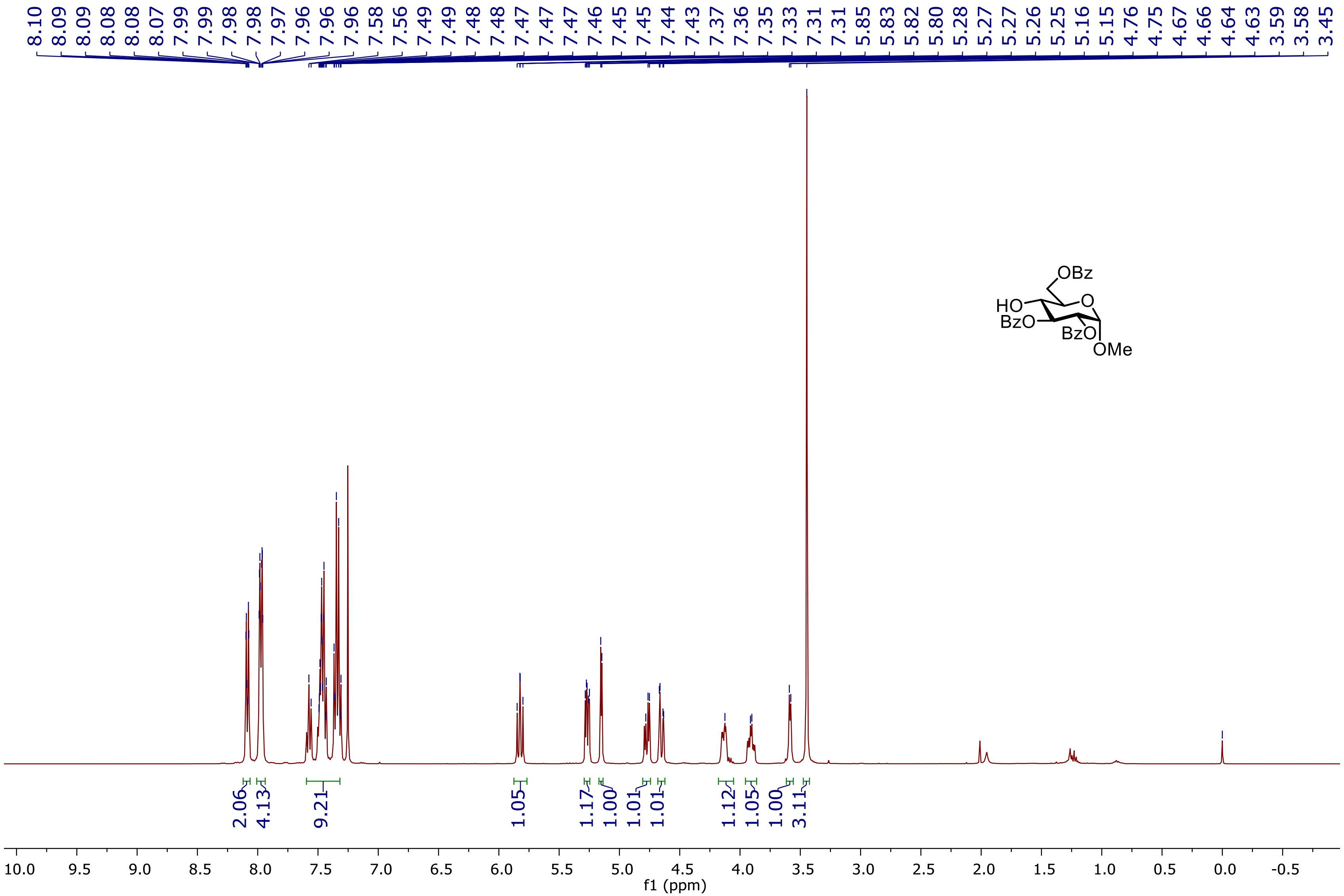

Supplementary Figure S8b. ^{13}C NMR Spectrum (101 MHz, $CDCl_3$) of compound **7**

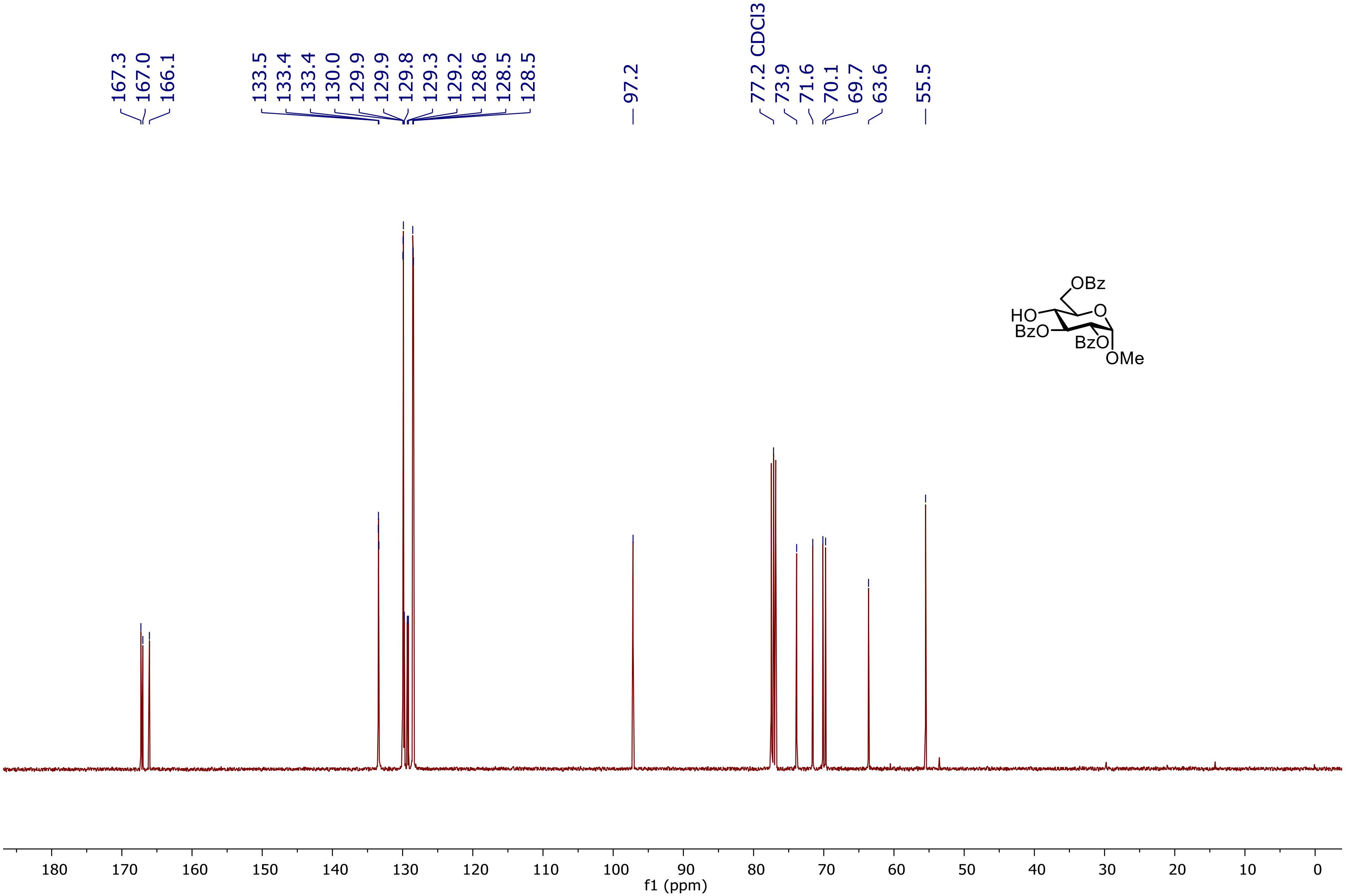

Supplementary Figure S8c. DEPT NMR Spectrum (101 MHz, $CDCl_3$) of compound **7**

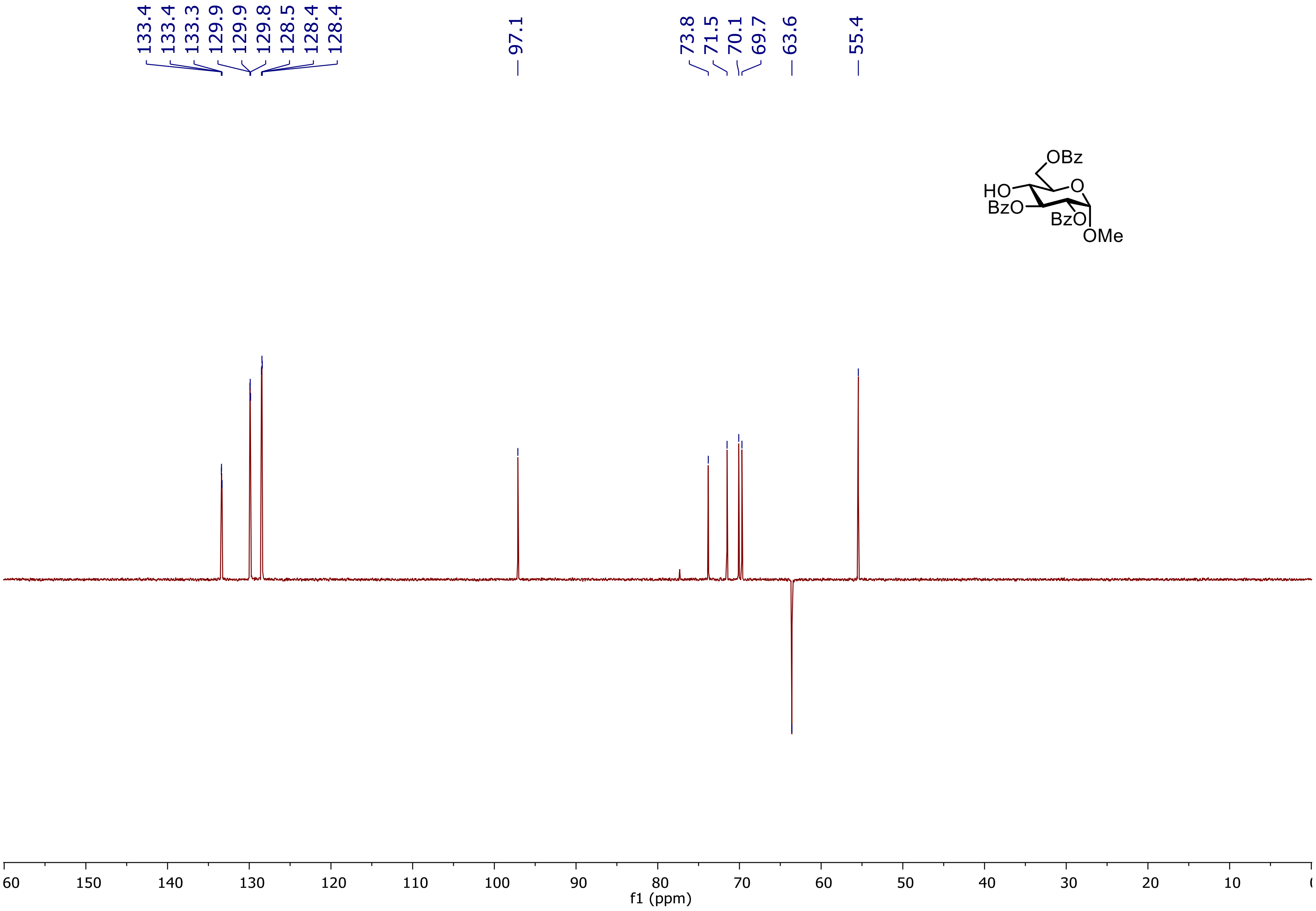

Supplementary Figure S9a. ^{1}H NMR Spectrum (400 MHz, $CDCl_3$) of compound **S10**

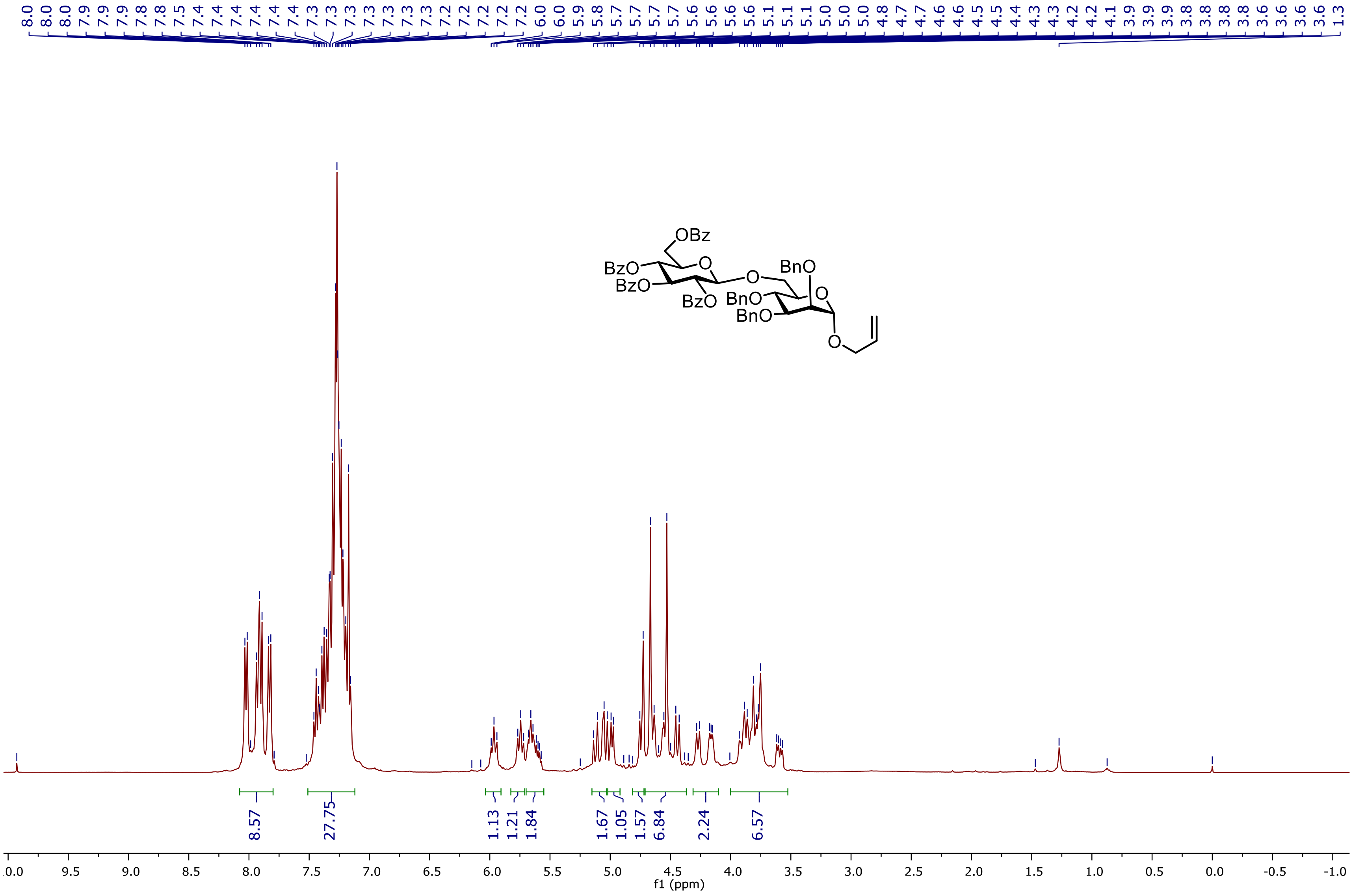

Supplementary Figure S9b. ^{13}C NMR Spectrum (101 MHz, $CDCl_3$) of compound **S10**

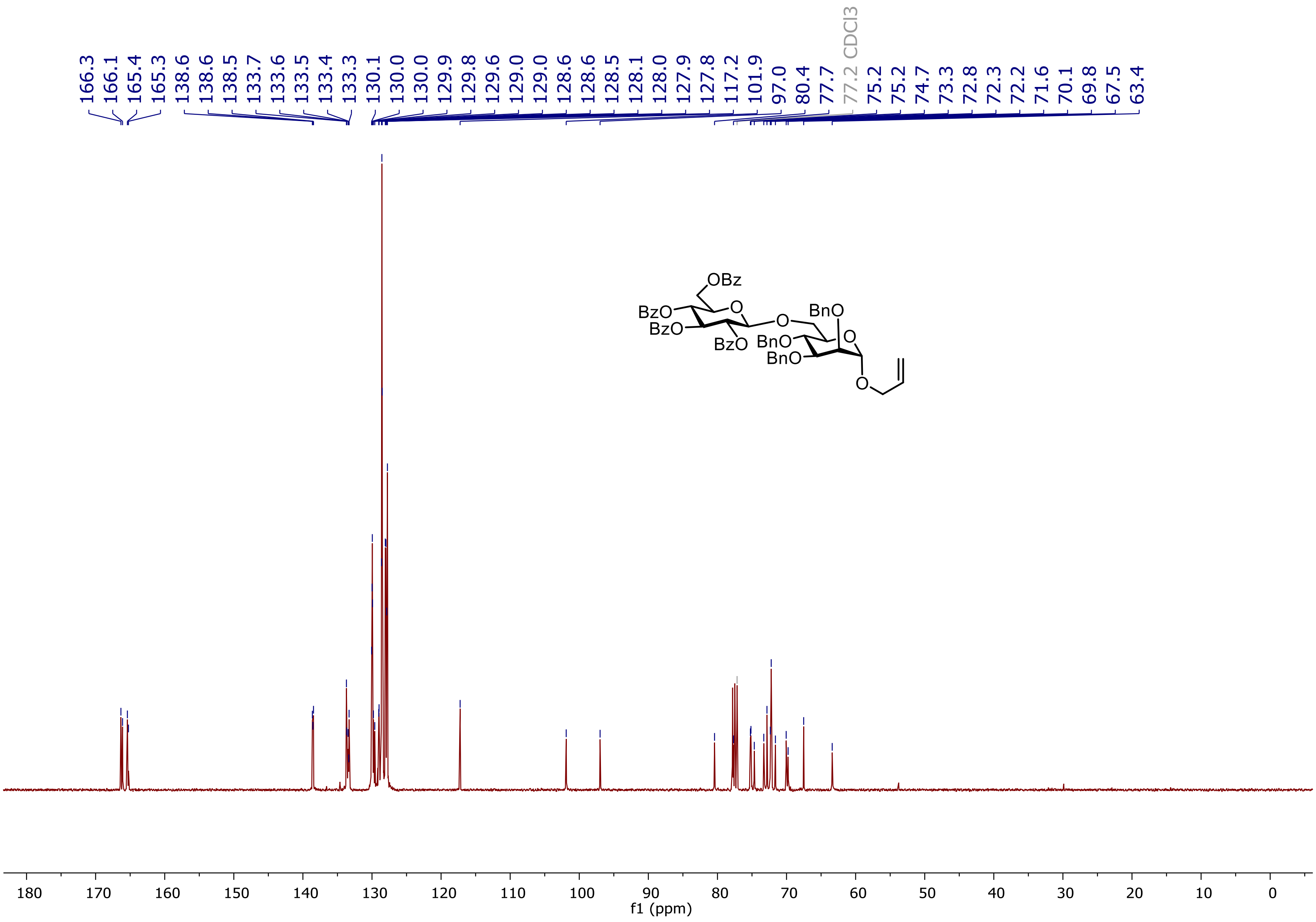

Supplementary Figure S9c. DEPT NMR Spectrum (101 MHz, $CDCl_3$) of compound **S10**

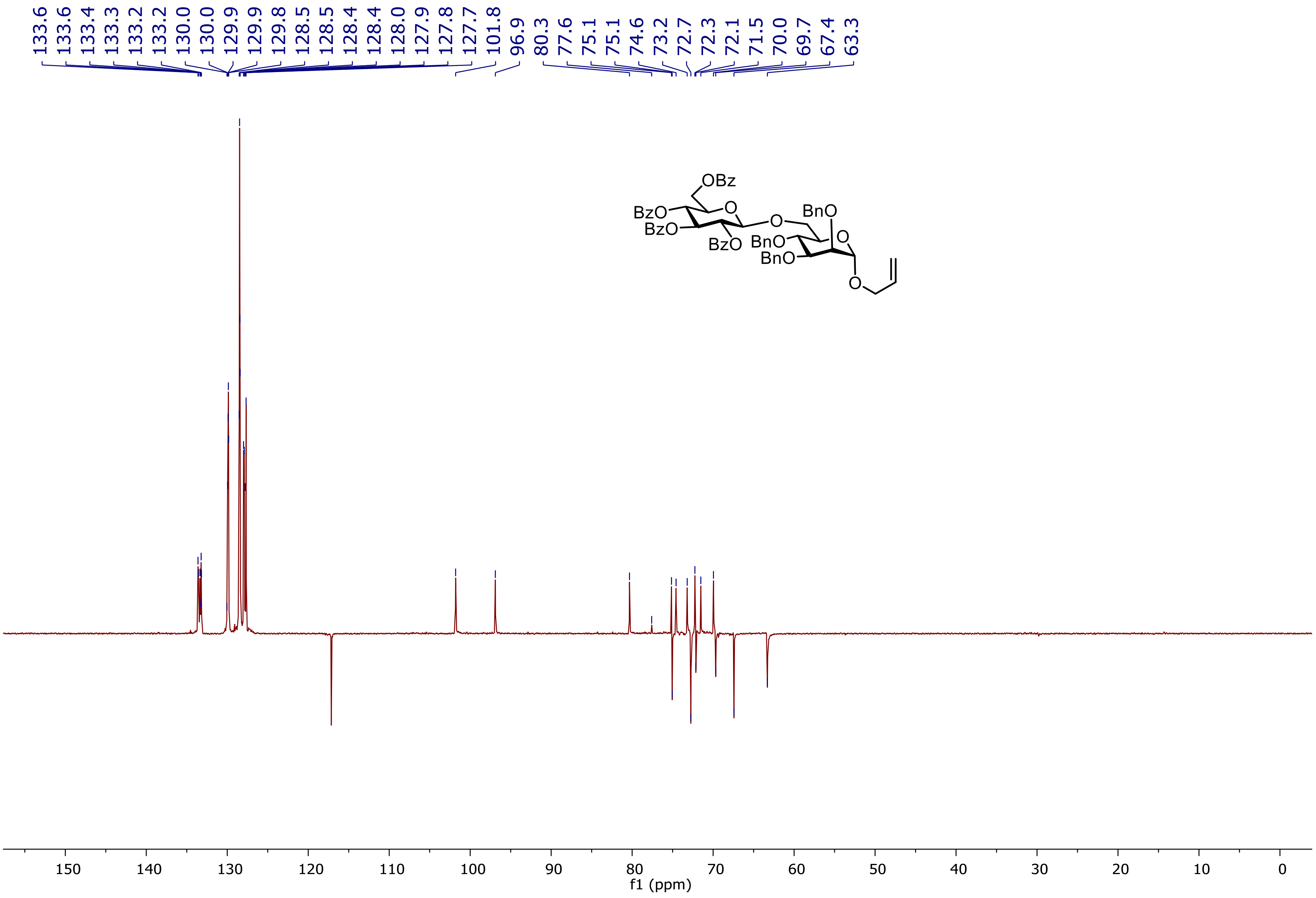

Supplementary Figure S10a. ^{1}H NMR Spectrum (400 MHz, $CDCl_3$) of compound **S11**

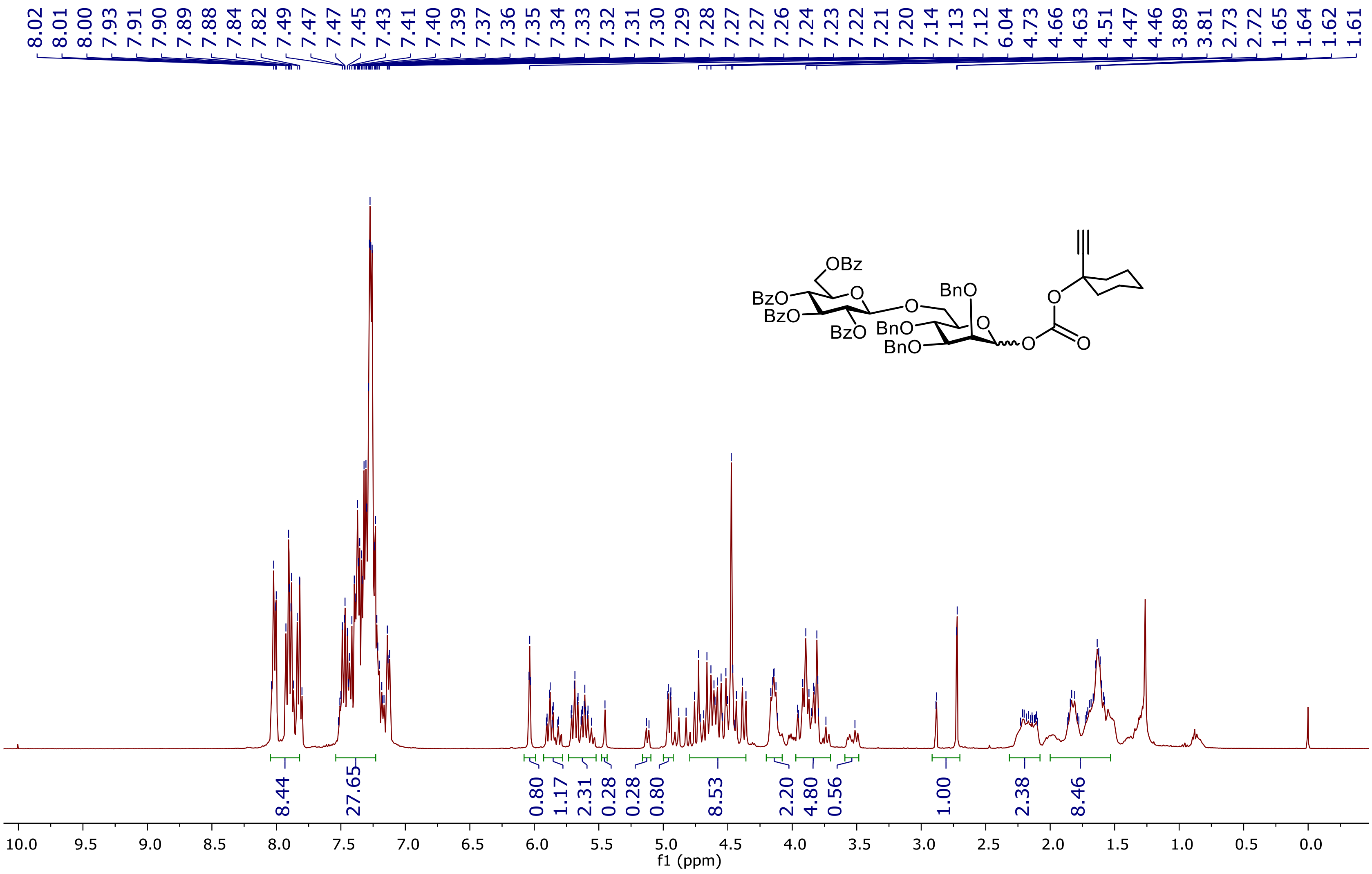

Supplementary Figure S10b. ^{13}C NMR Spectrum (101 MHz, $CDCl_3$) of compound **S11**

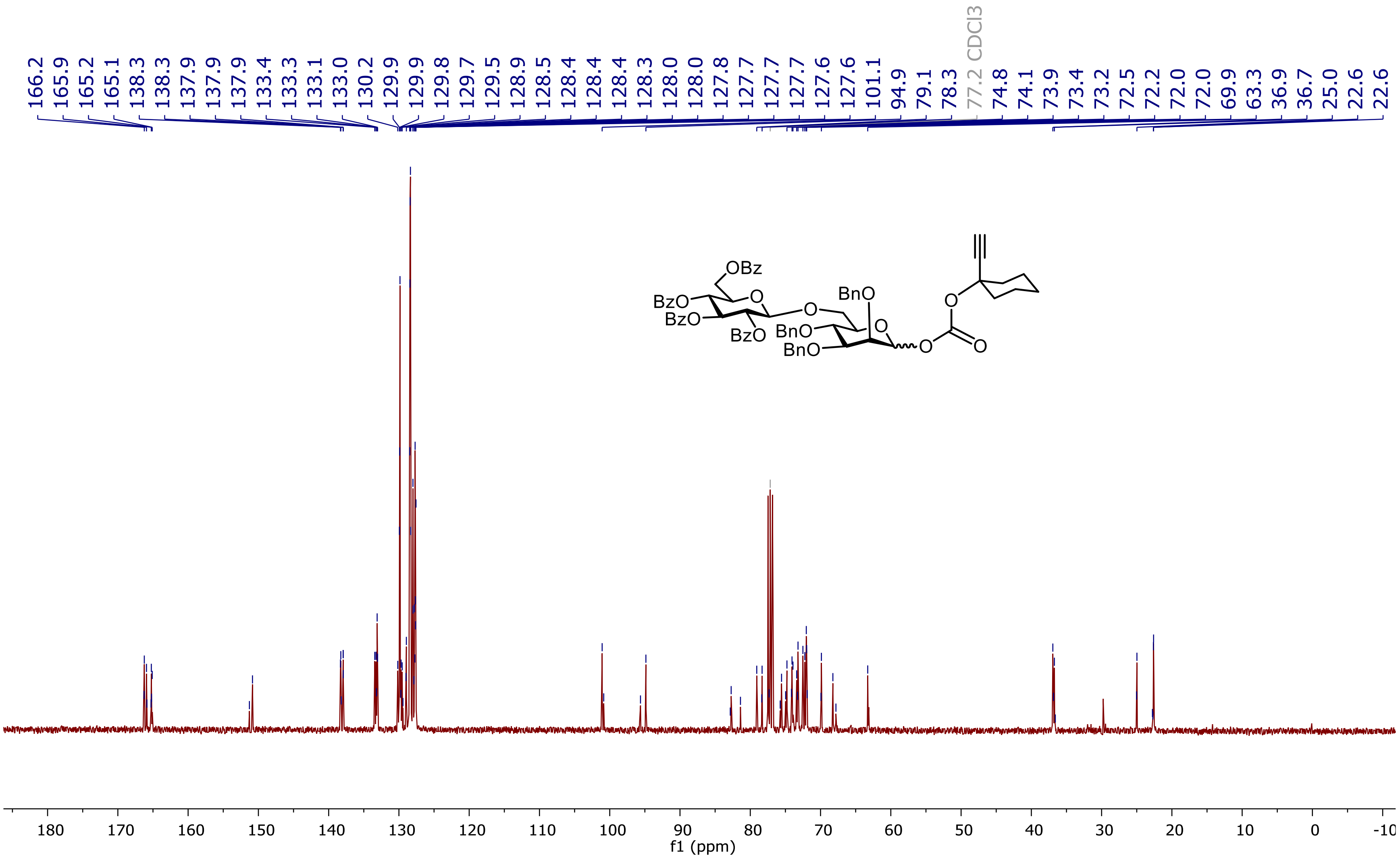

Supplementary Figure S10c. DEPT NMR Spectrum (101 MHz, $CDCl_3$) of compound **S11**

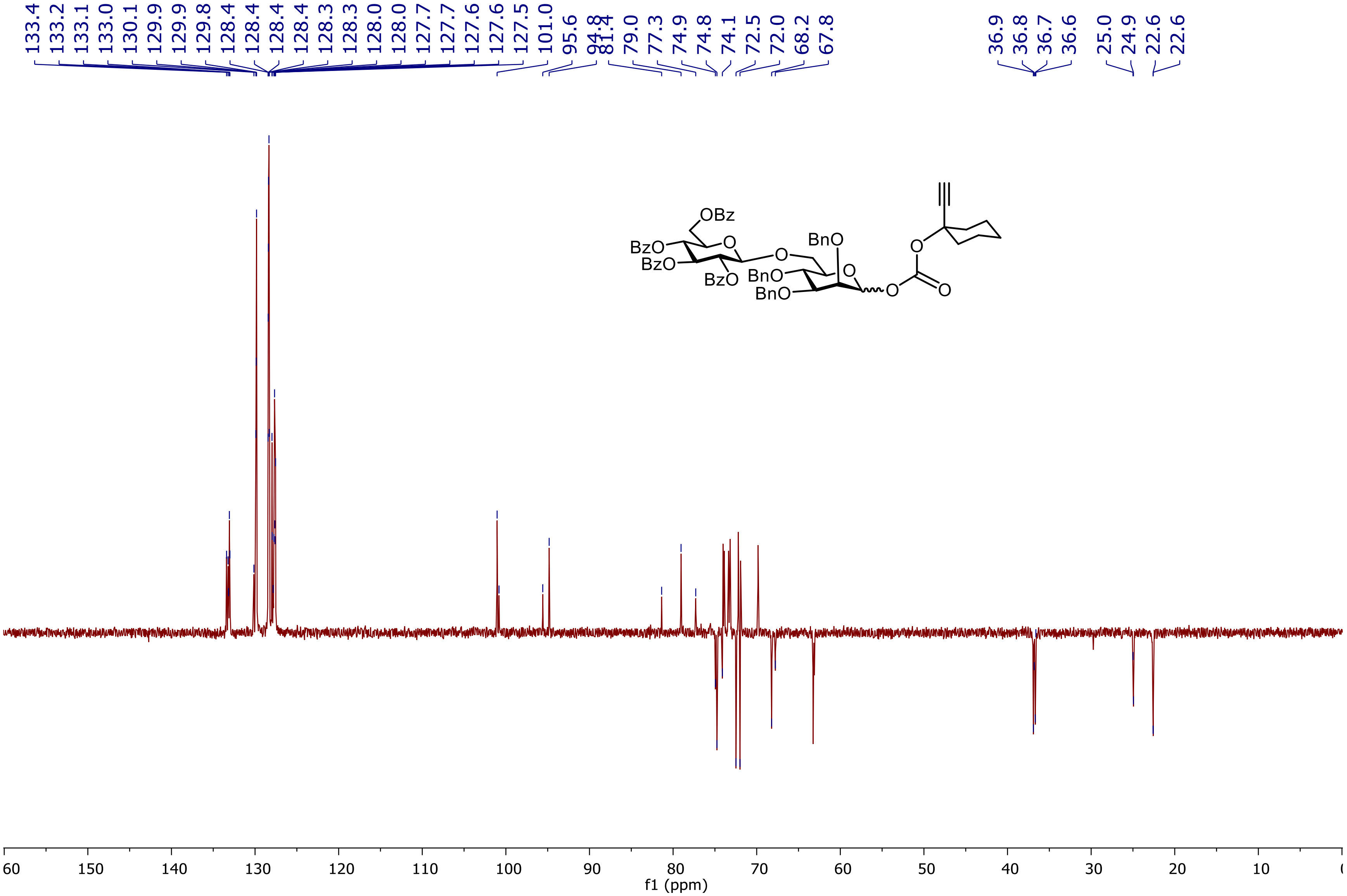

Supplementary Figure S11a. ^{1}H NMR Spectrum (400 MHz, $CDCl_3$) of compound **5**

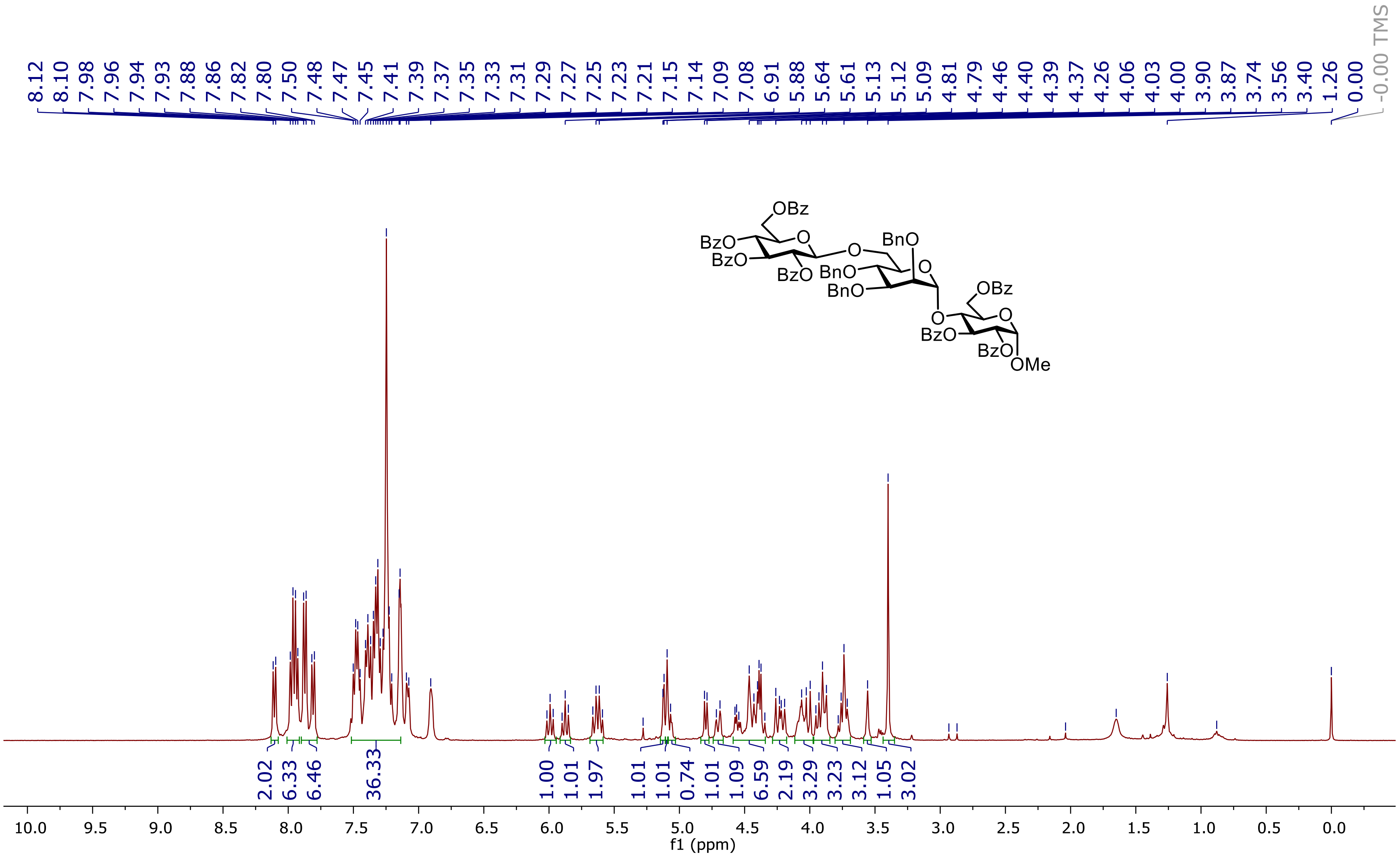

Supplementary Figure S11b. ^{13}C NMR Spectrum (101 MHz, $CDCl_3$) of compound **5**

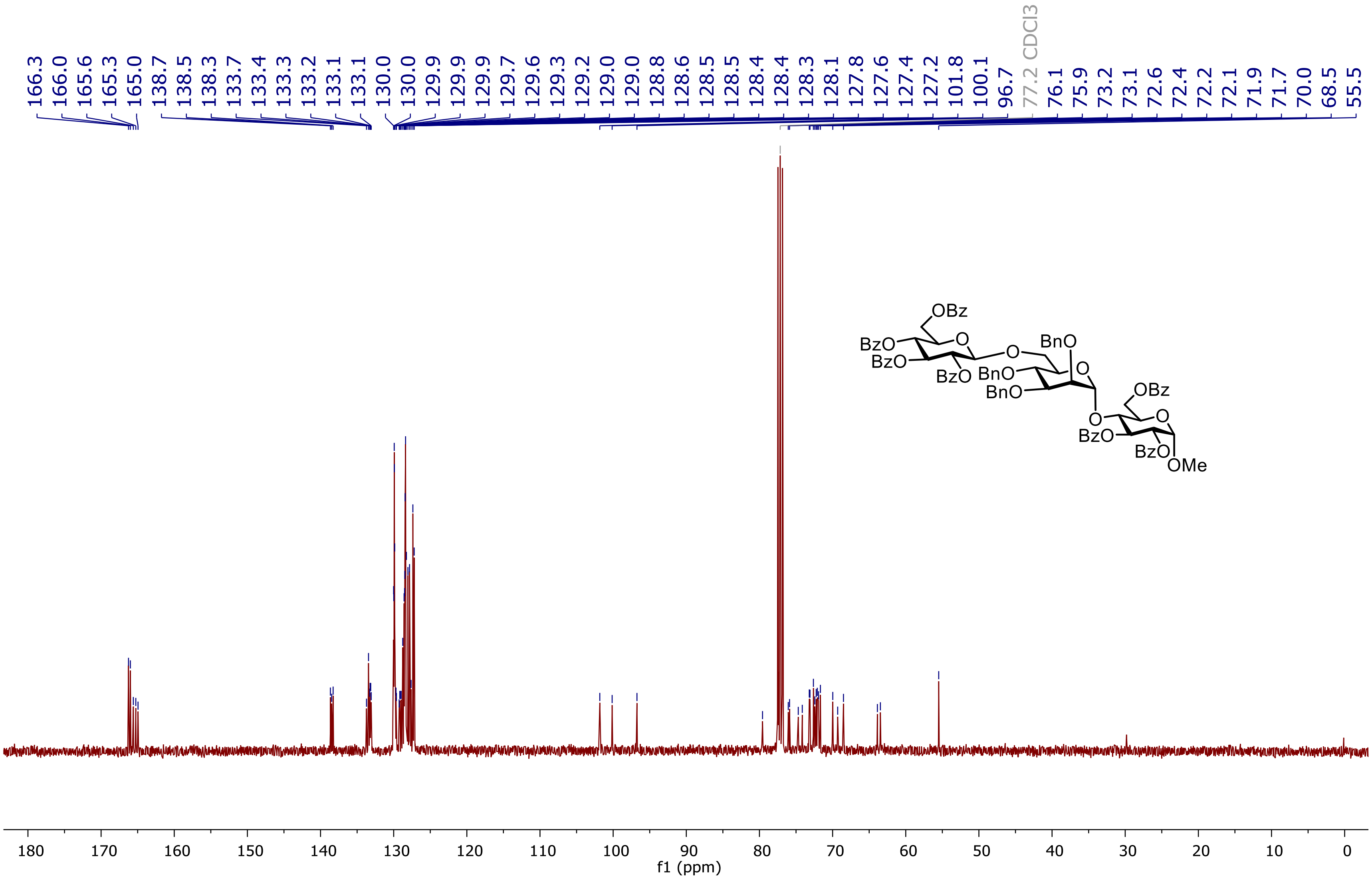

Supplementary Figure S11c. DEPT NMR Spectrum (101 MHz, $CDCl_3$) of compound **5**

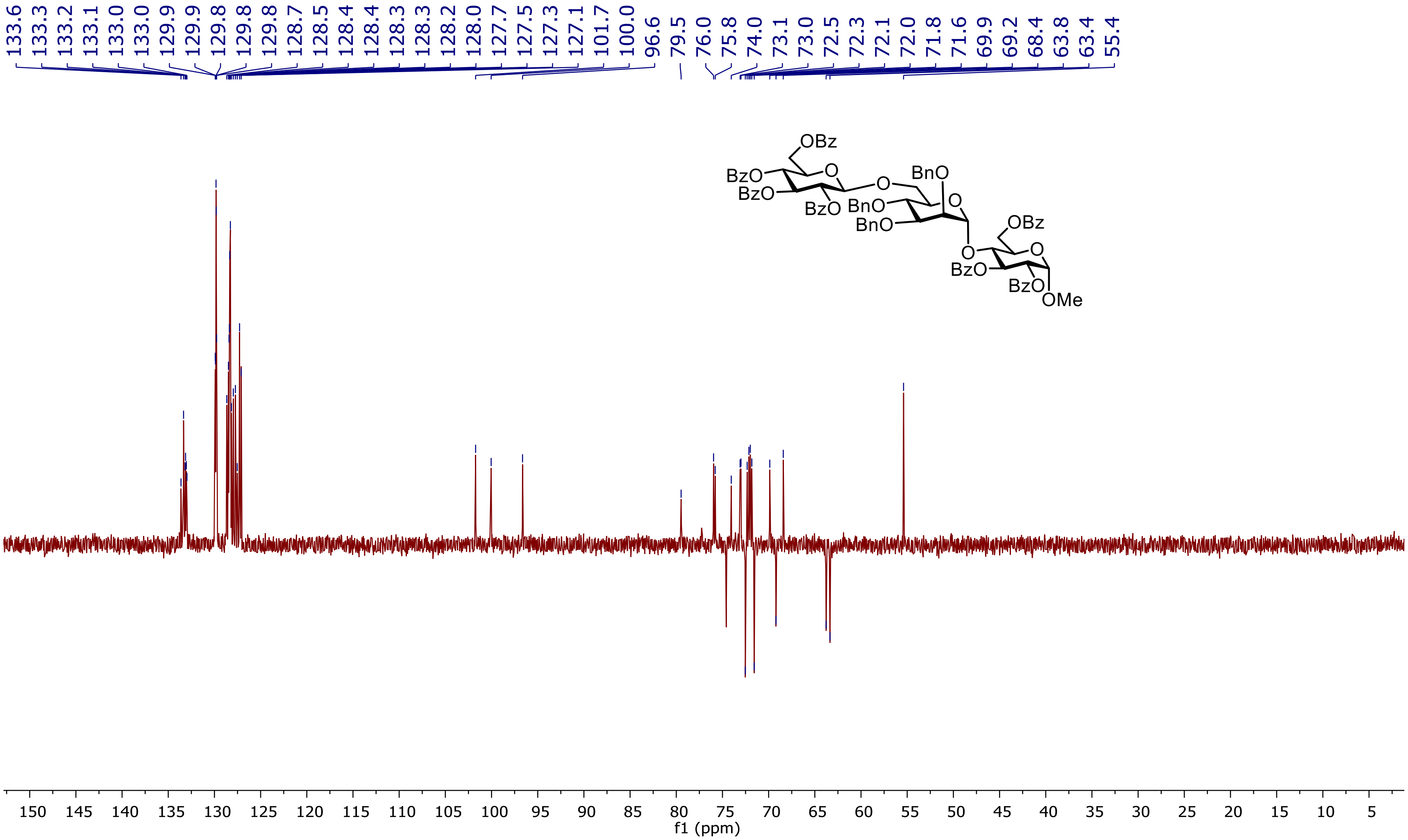

Supplementary Figure S12a. ^{1}H NMR Spectrum (400 MHz, $CDCl_3$) of compound **6c**

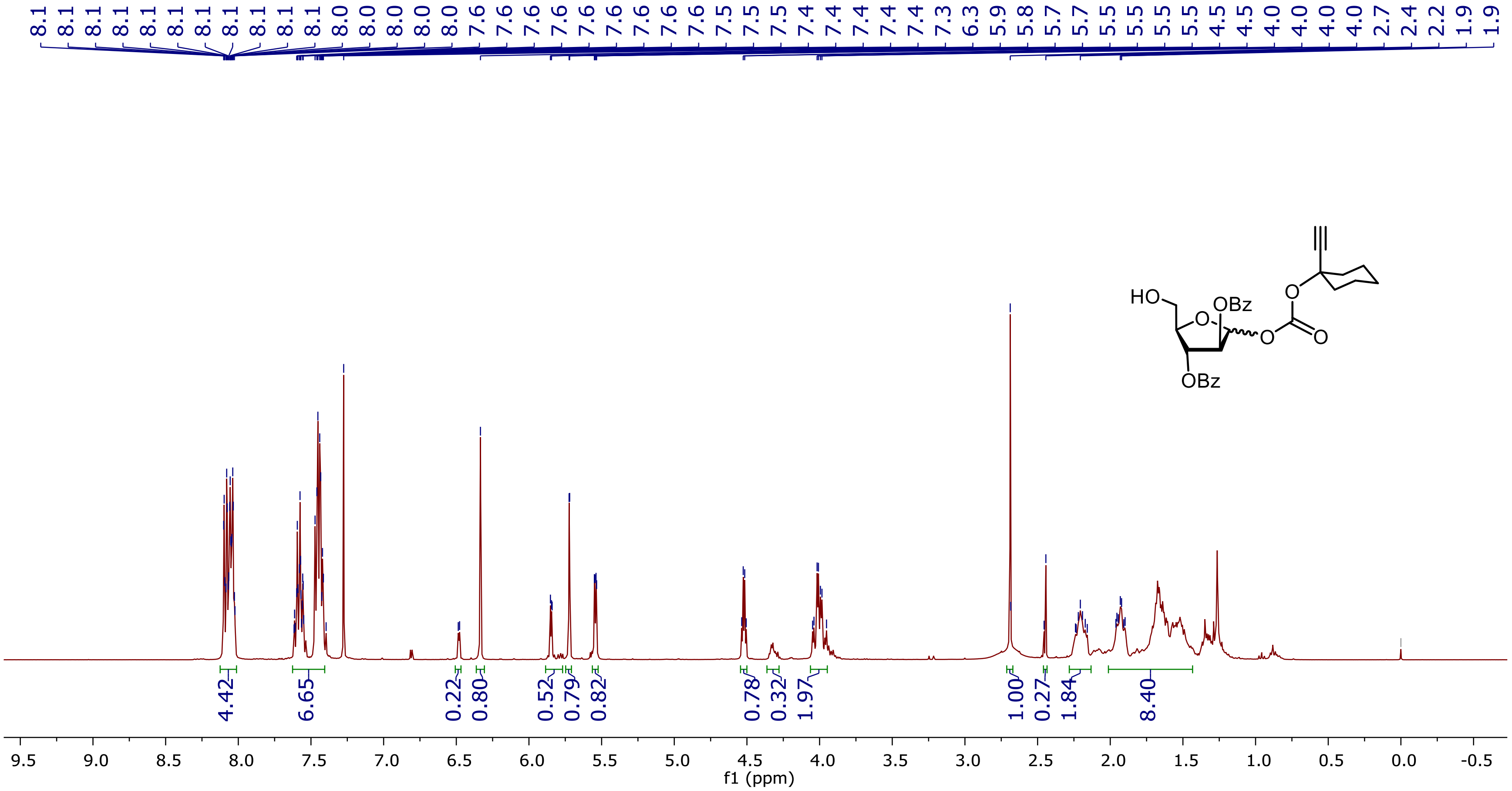

Supplementary Figure S12b. ^{13}C NMR Spectrum (101 MHz, $CDCl_3$) of compound **6c**

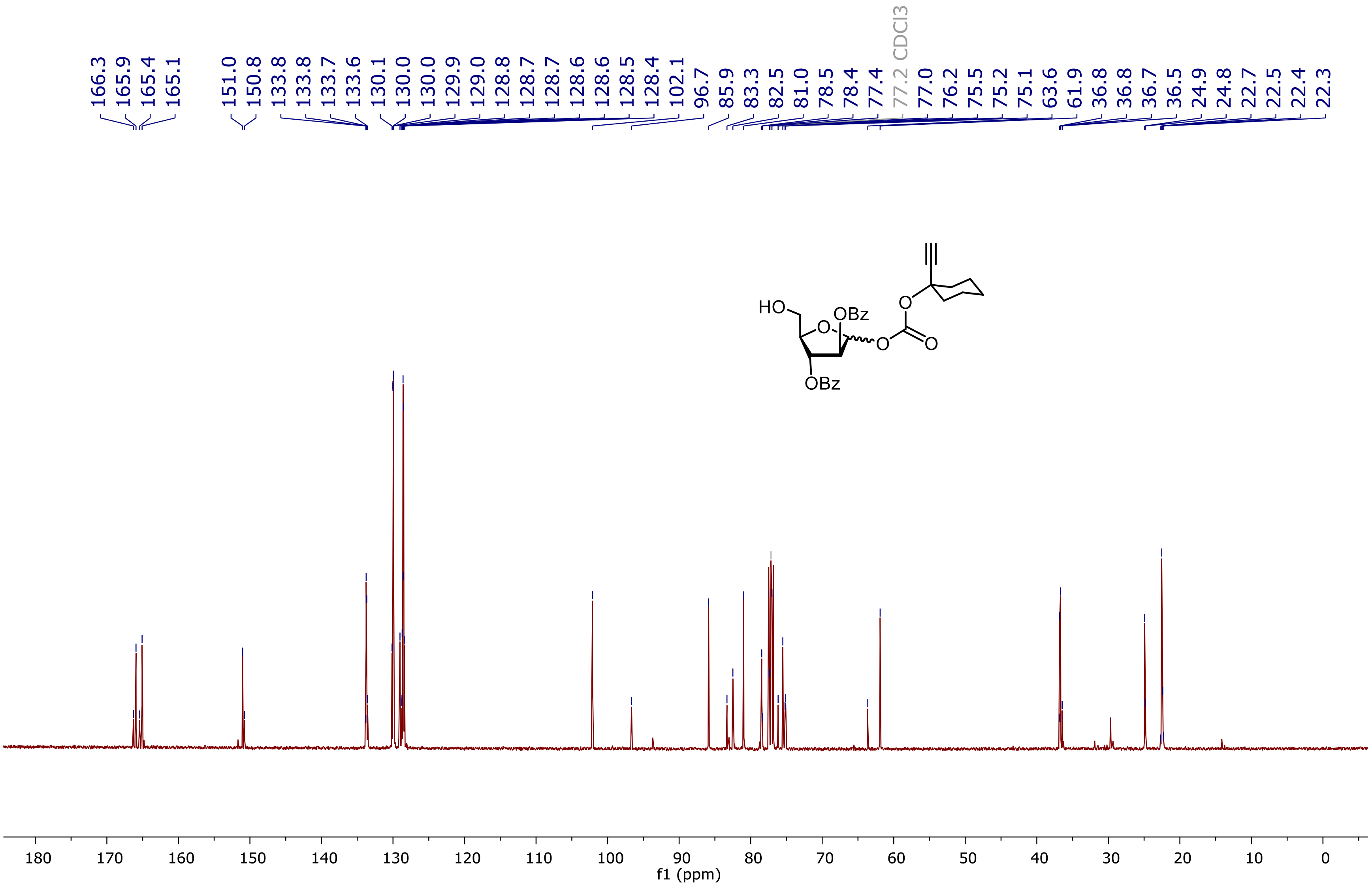

Supplementary Figure S12c. DEPT NMR Spectrum (101 MHz, $CDCl_3$) of compound **6c**

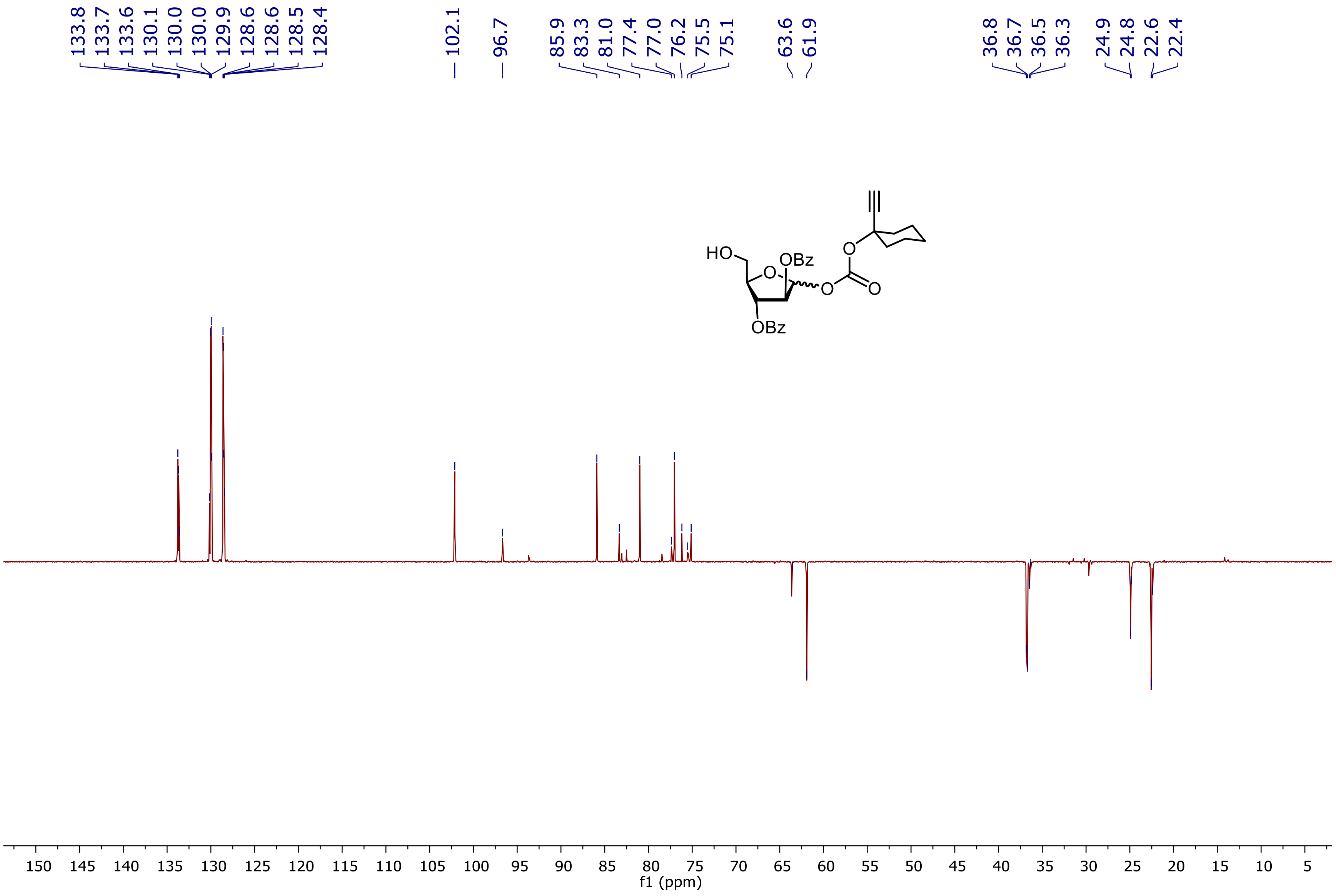

Supplementary Figure S13a. ^{1}H NMR Spectrum (400 MHz, $CDCl_3$) of compound **S13**

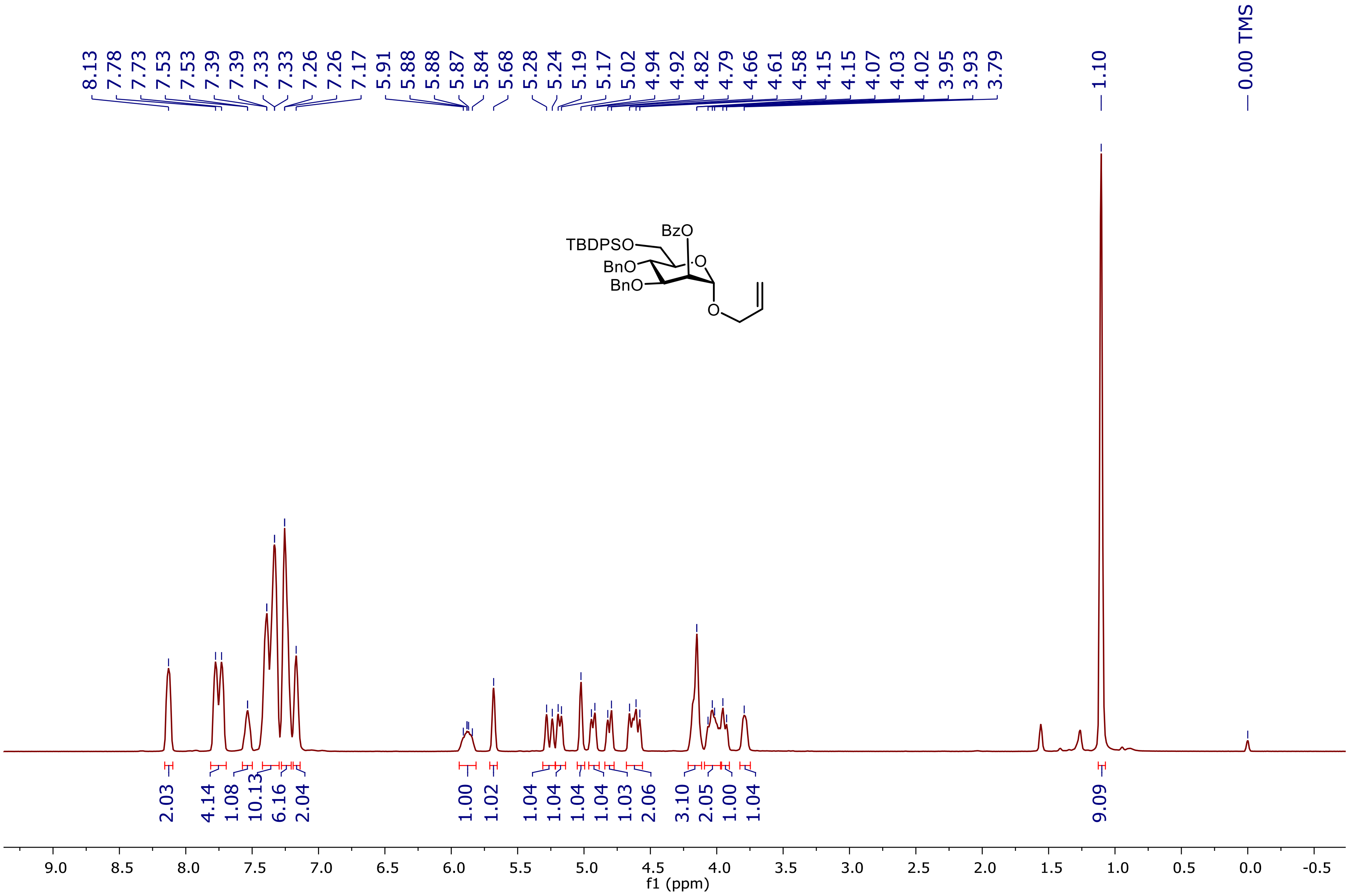

Supplementary Figure S13b. ^{13}C NMR Spectrum (101 MHz, $CDCl_3$) of compound **S13**

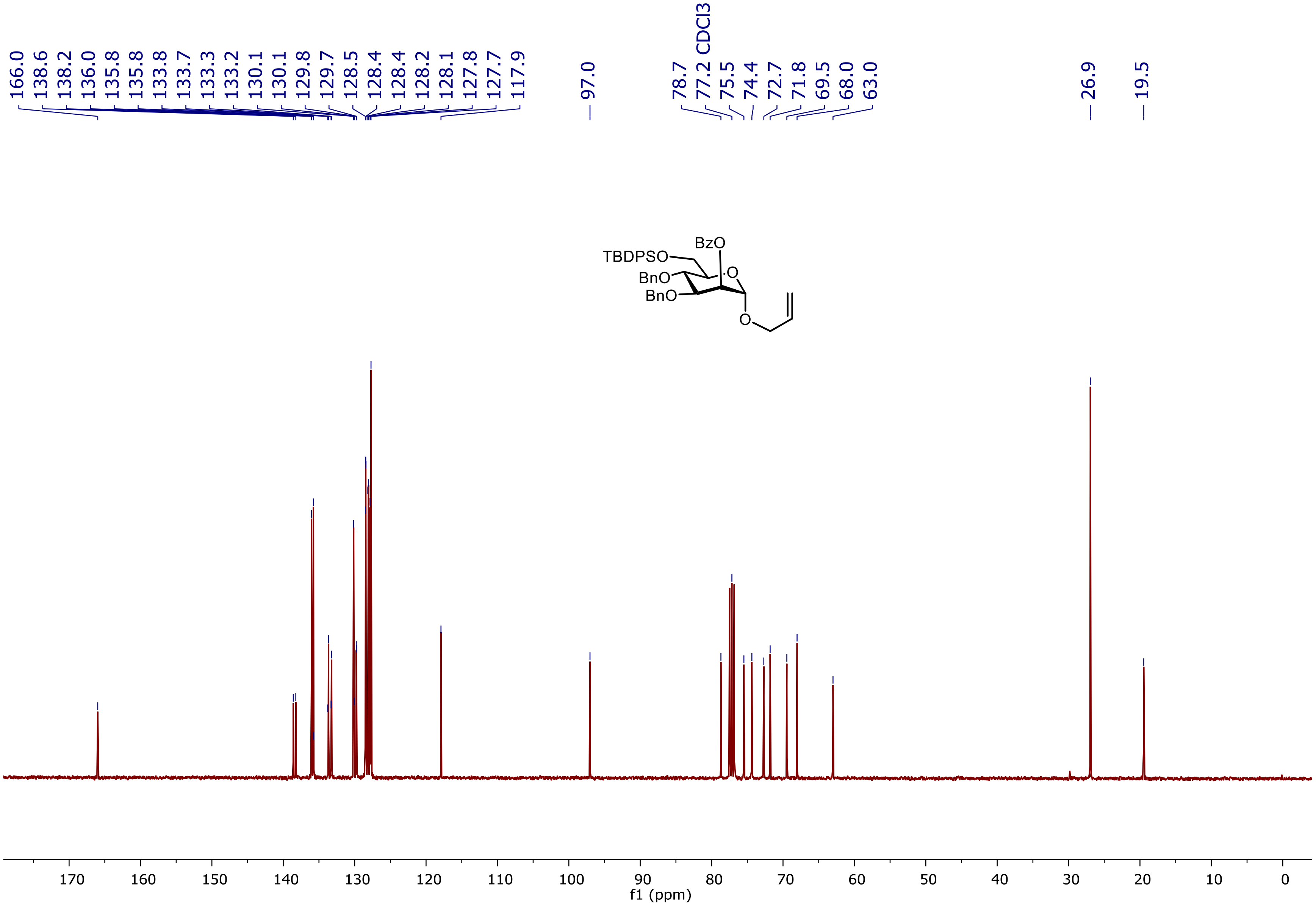

Supplementary Figure S13c. DEPT NMR Spectrum (101 MHz, $CDCl_3$) of compound **S13**

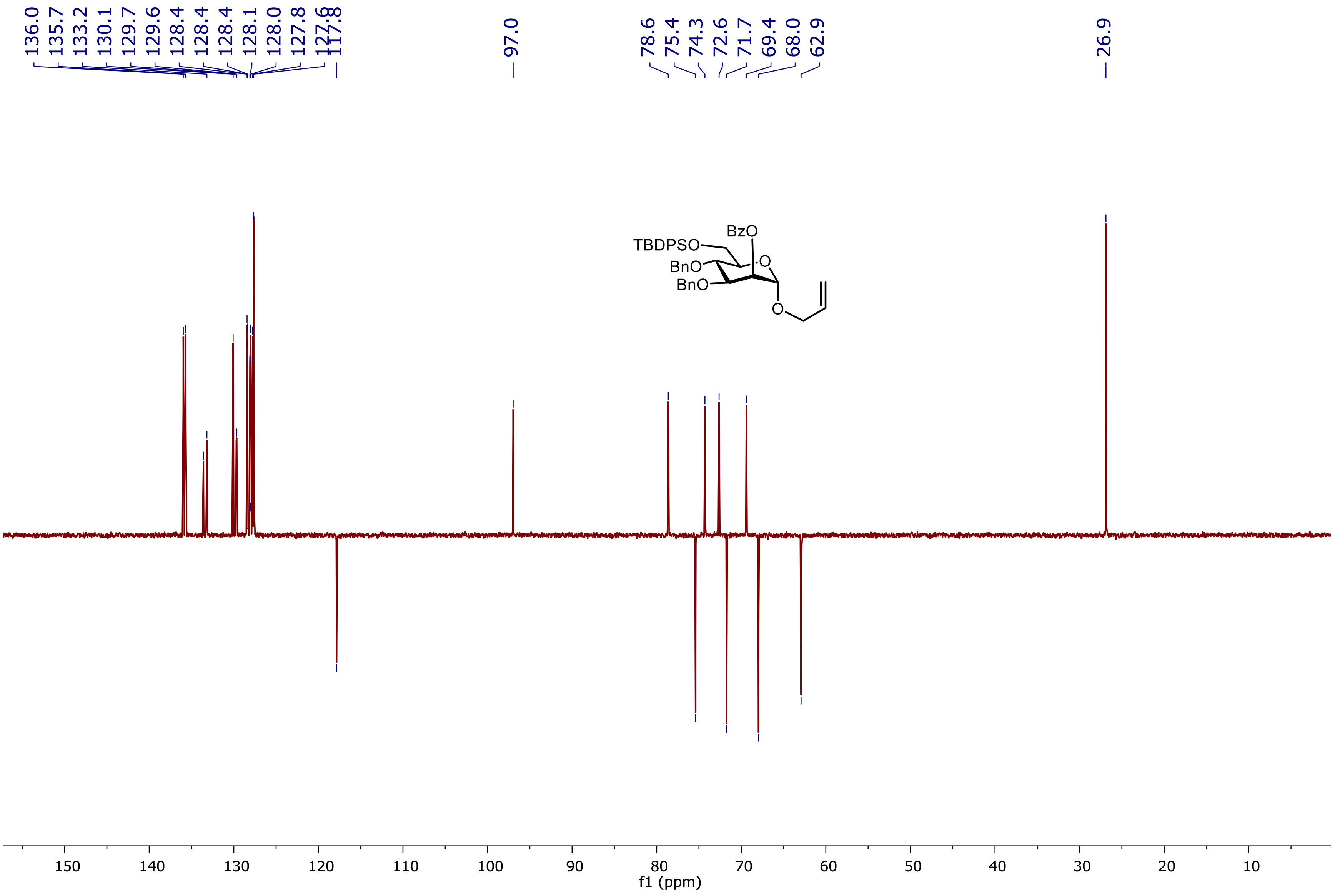

Supplementary Figure S14a. ^{1}H NMR Spectrum (400 MHz, $CDCl_3$) of compound **6a**

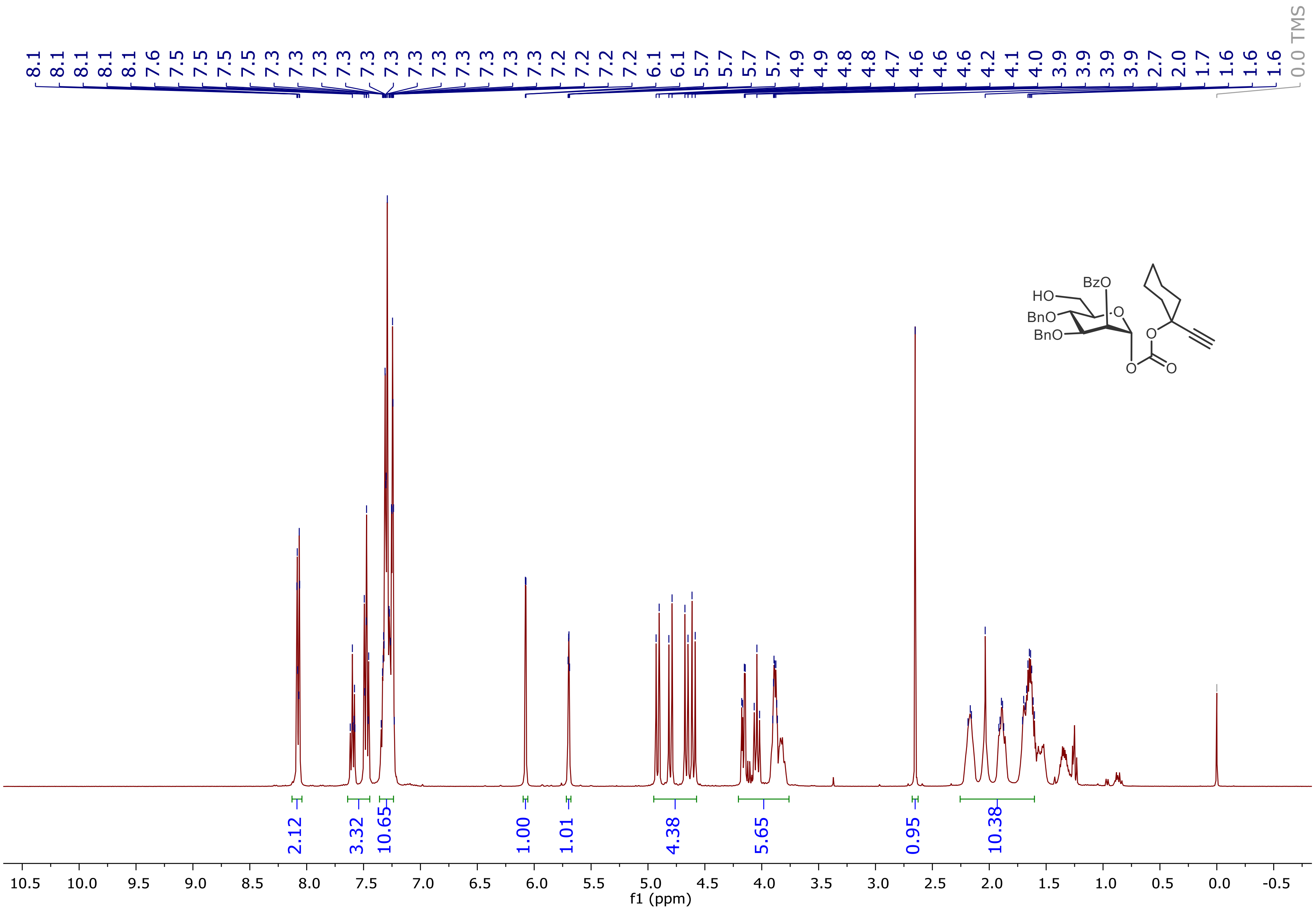

Supplementary Figure S14b. ^{13}C NMR Spectrum (101 MHz, $CDCl_3$) of compound **6a**

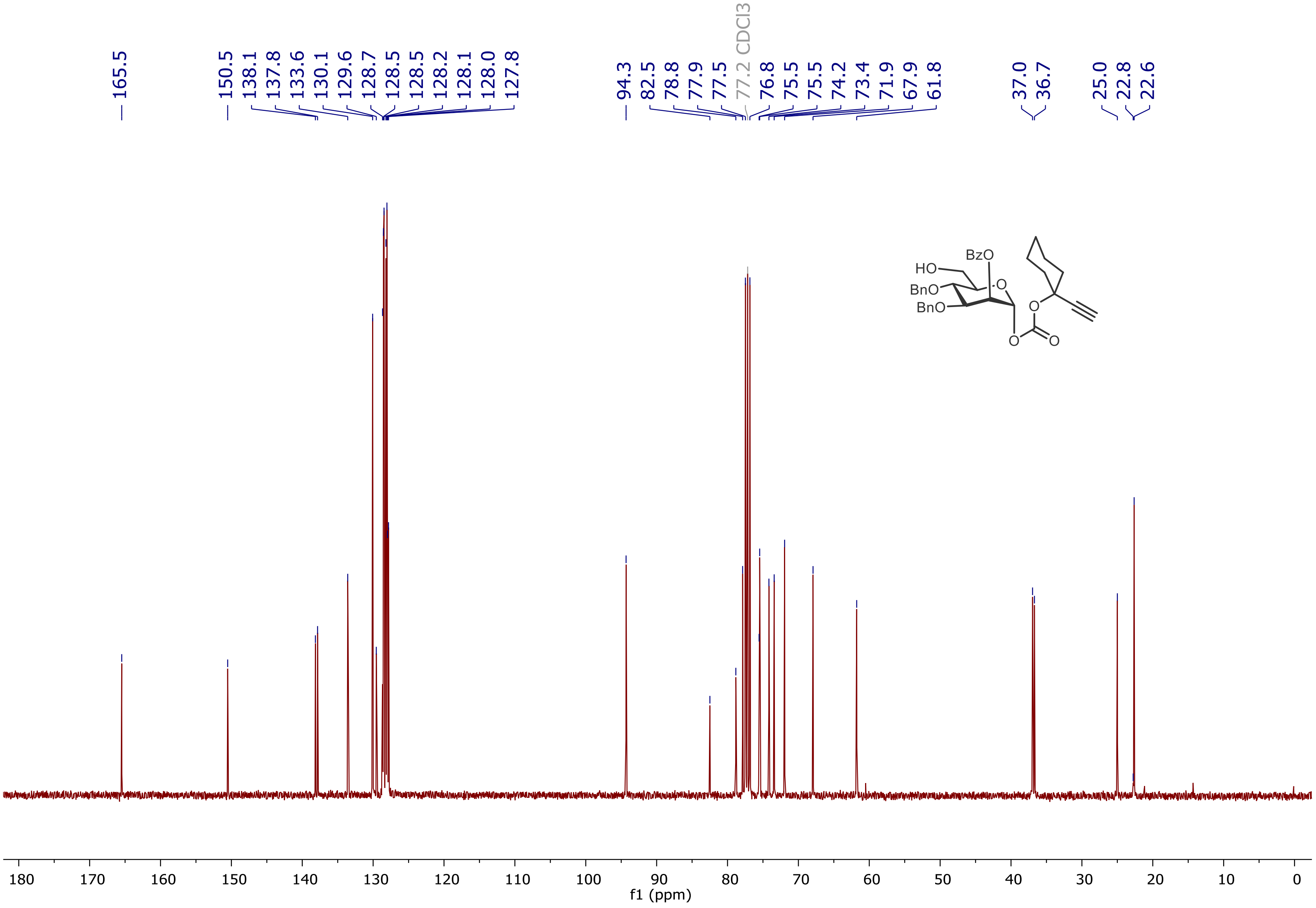

Supplementary Figure S14c. DEPT NMR Spectrum (101 MHz, $CDCl_3$) of compound **6a**

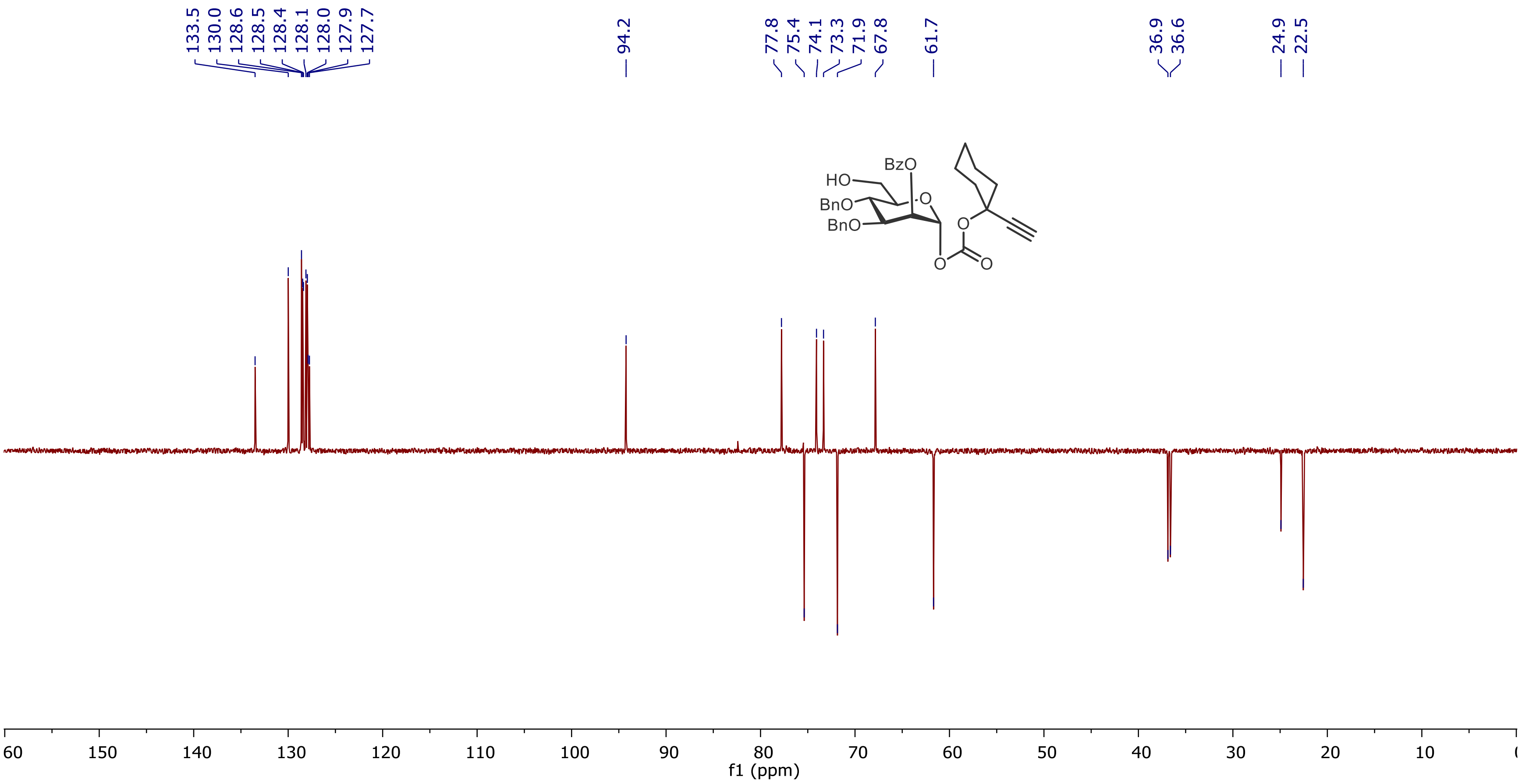

Supplementary Figure S15a. ^{1}H NMR Spectrum (400 MHz, $CDCl_3$) of compound **6b**

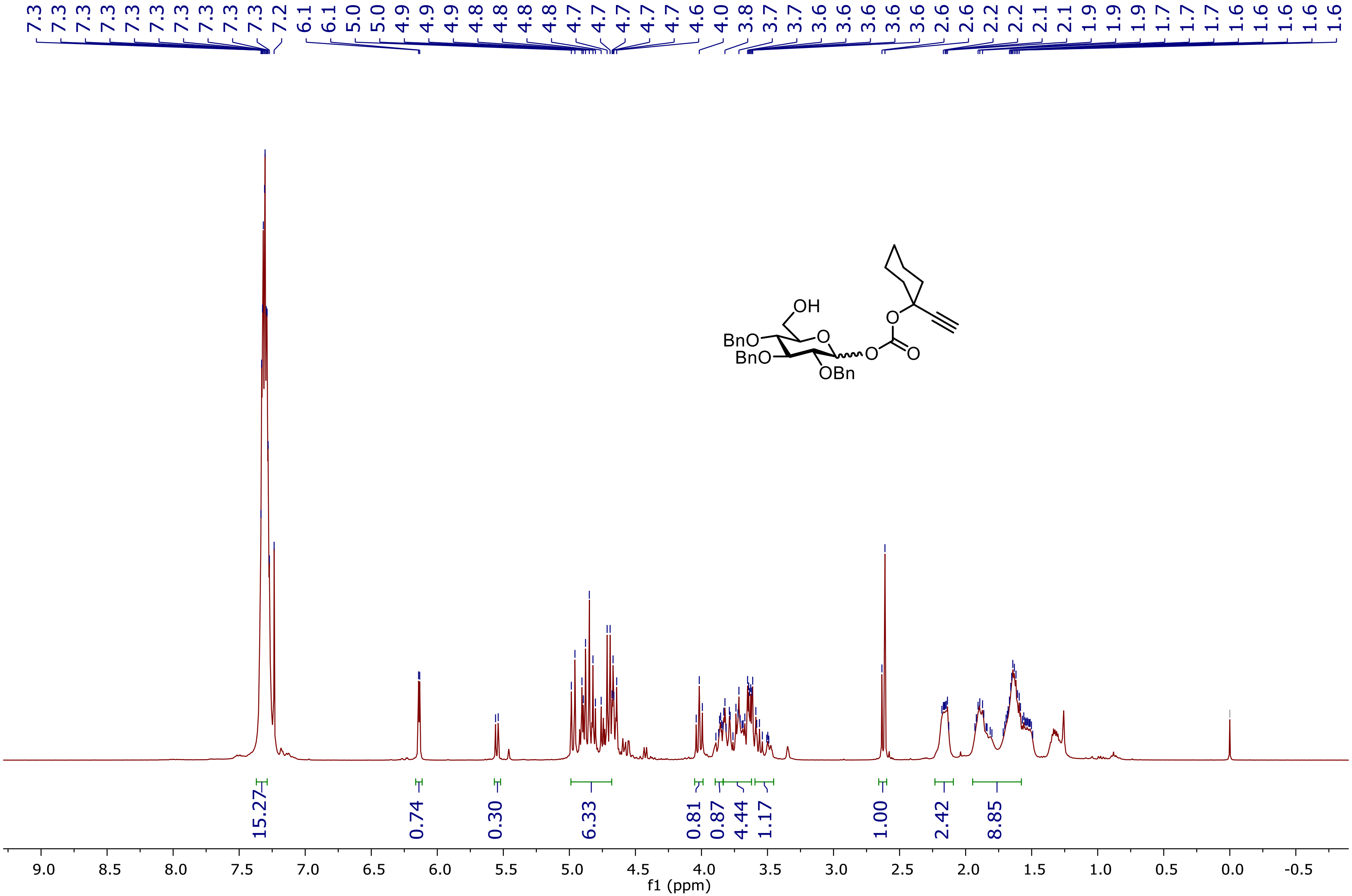

Supplementary Figure S15b. ^{13}C NMR Spectrum (101 MHz, $CDCl_3$) of compound **6b**

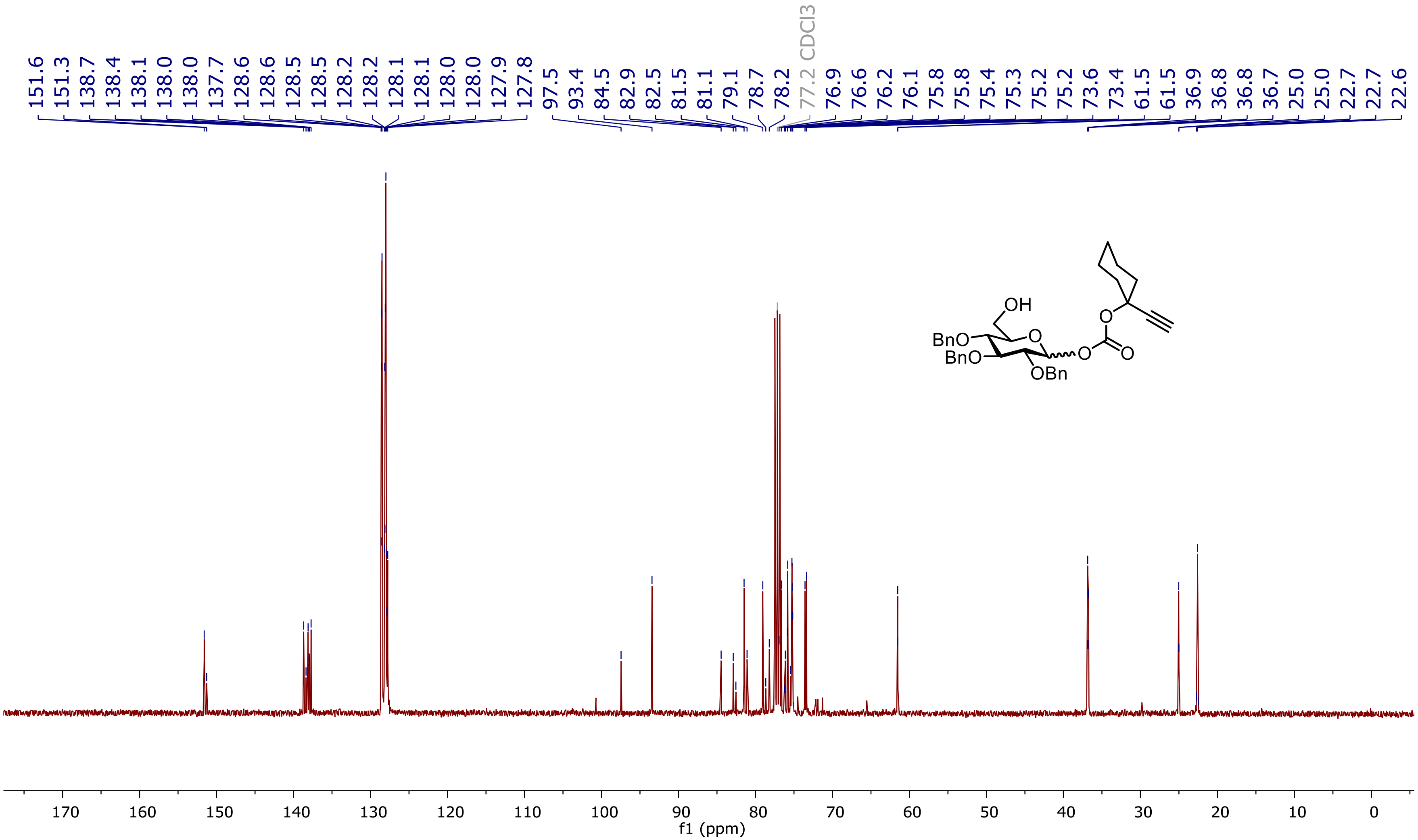

Supplementary Figure S15c. DEPT NMR Spectrum (101 MHz, $CDCl_3$) of compound **6b**

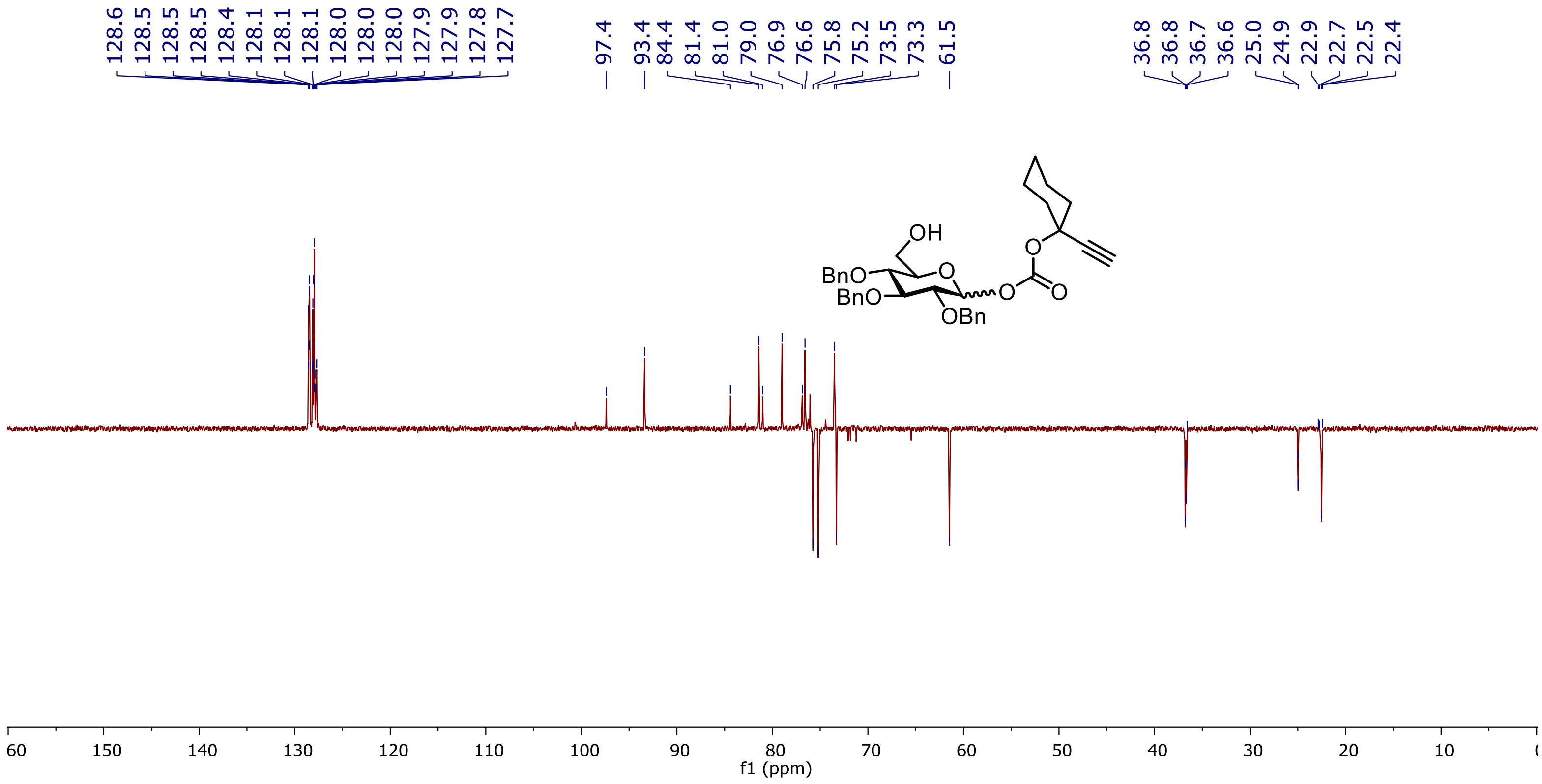

Supplementary Figure S16a. ^{1}H NMR Spectrum (400 MHz, $CDCl_3$) of compound **6d**

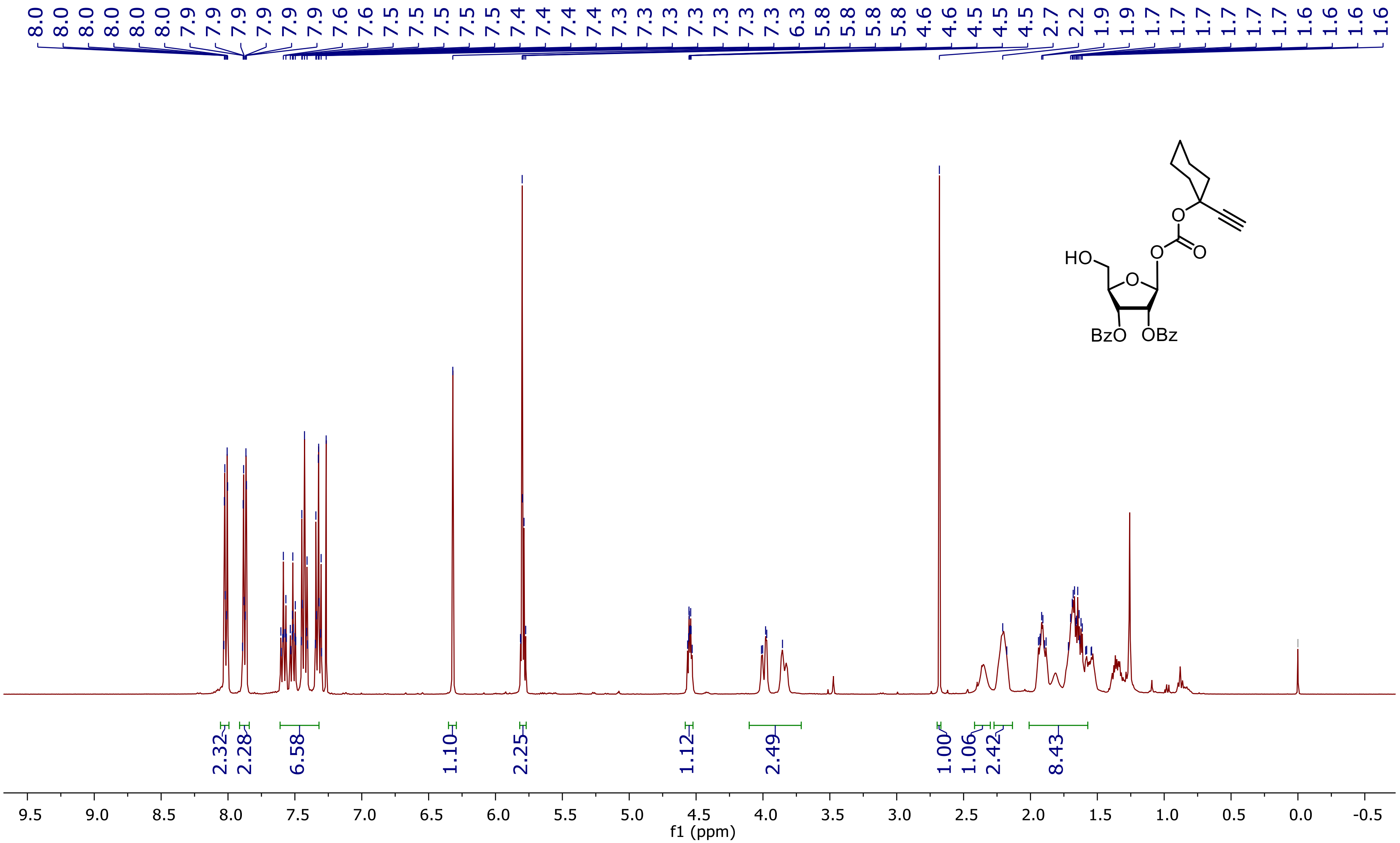

Supplementary Figure S16b. ^{13}C NMR Spectrum (101 MHz, $CDCl_3$) of compound **6d**

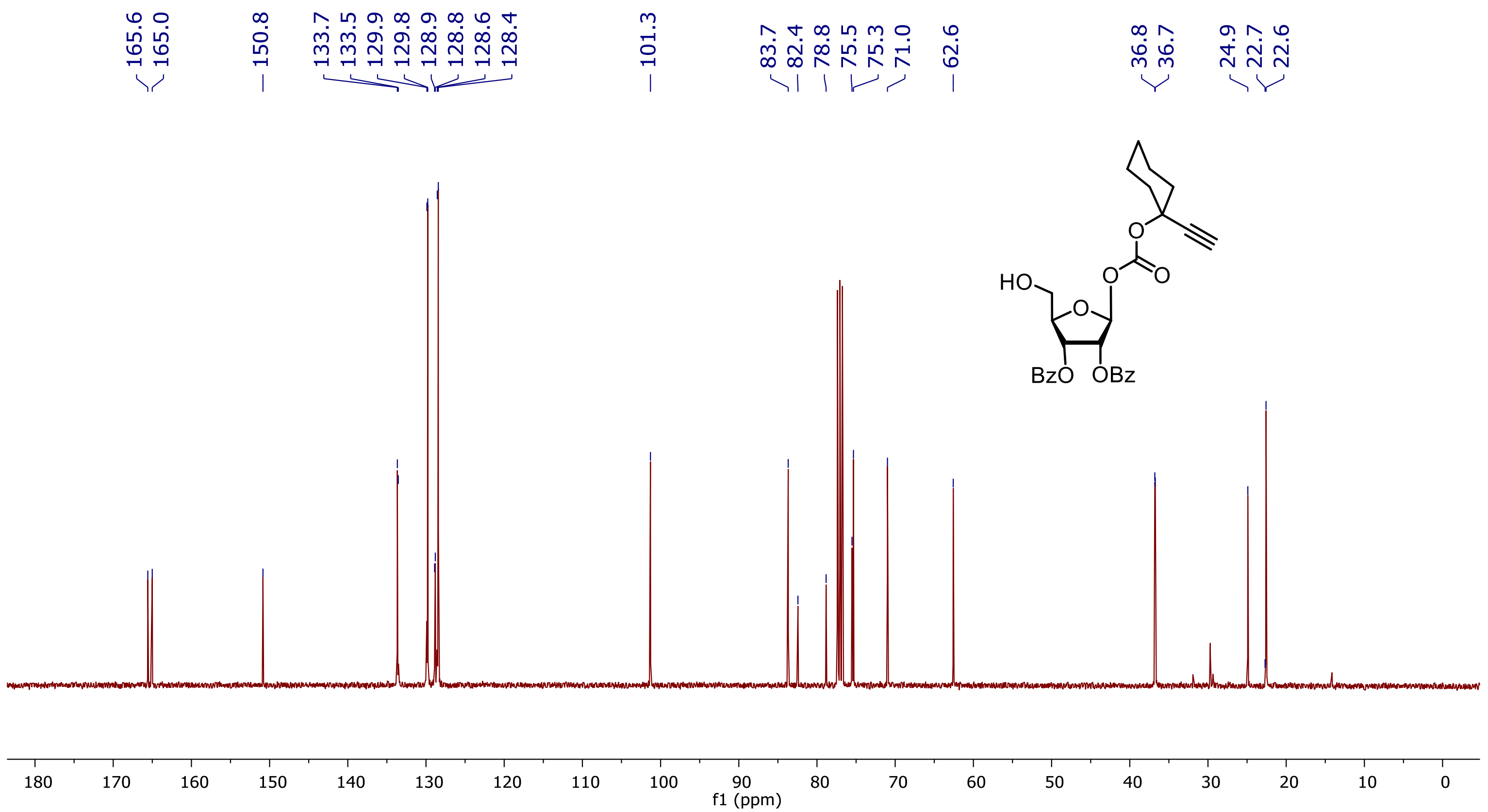

Supplementary Figure S16c. DEPT NMR Spectrum (101 MHz, $CDCl_3$) of compound **6d**

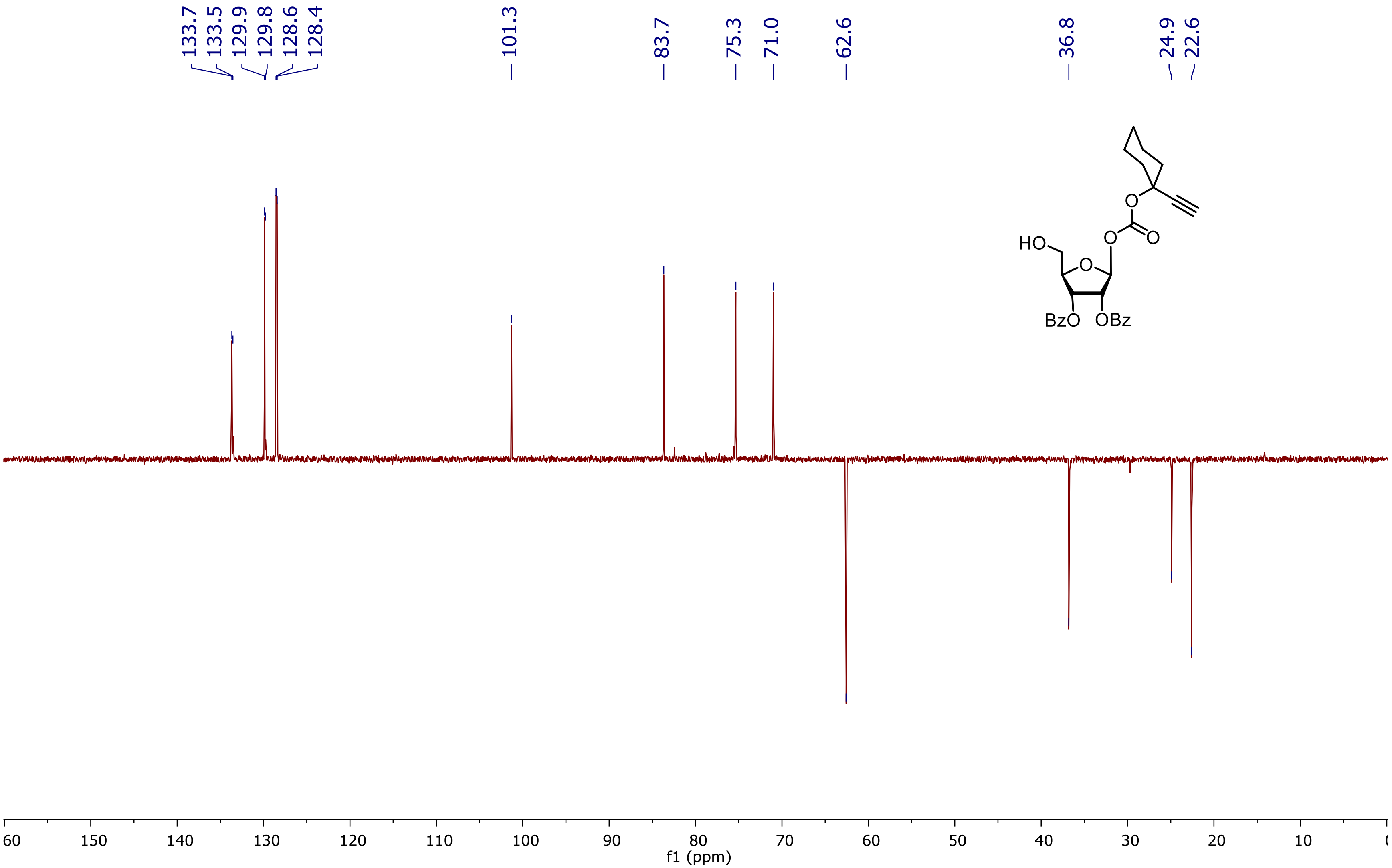

Supplementary Figure S17a. ^{1}H NMR Spectrum (400 MHz, $CDCl_3$) of compound **6e**

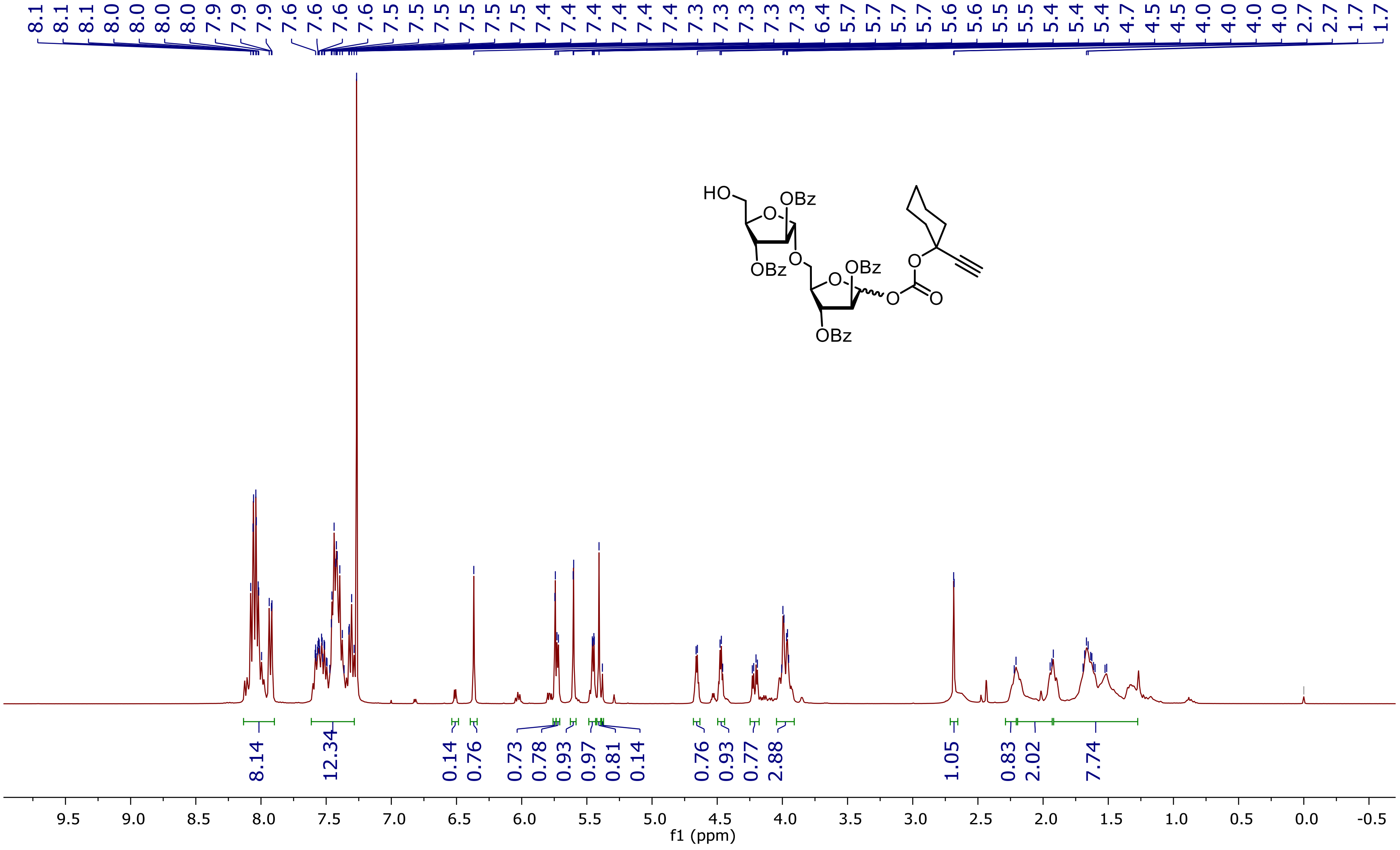

Supplementary Figure S17b. ^{13}C NMR Spectrum (101 MHz, $CDCl_3$) of compound **6e**

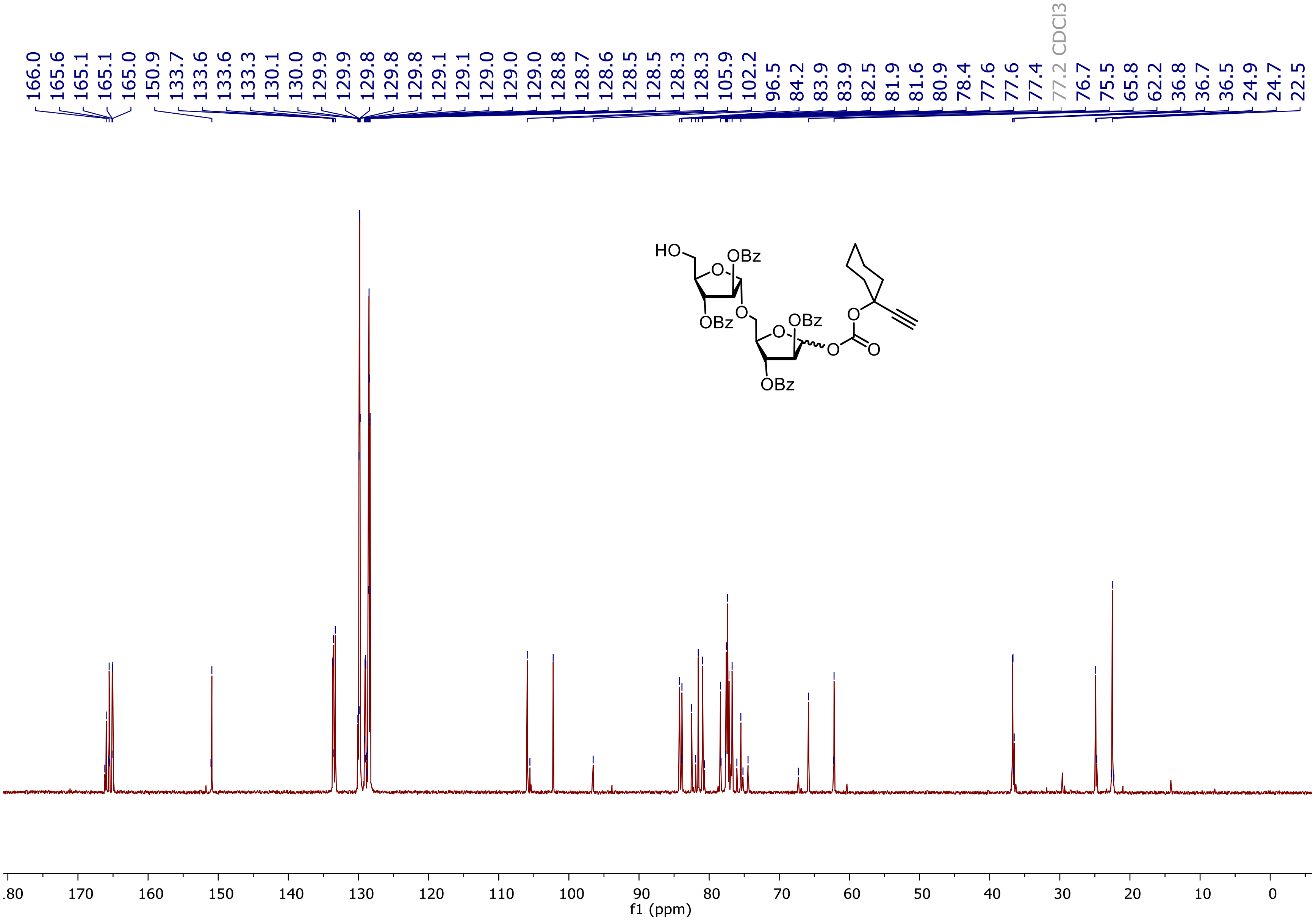

Supplementary Figure S17c. DEPT NMR Spectrum (101 MHz, $CDCl_3$) of compound **6e**

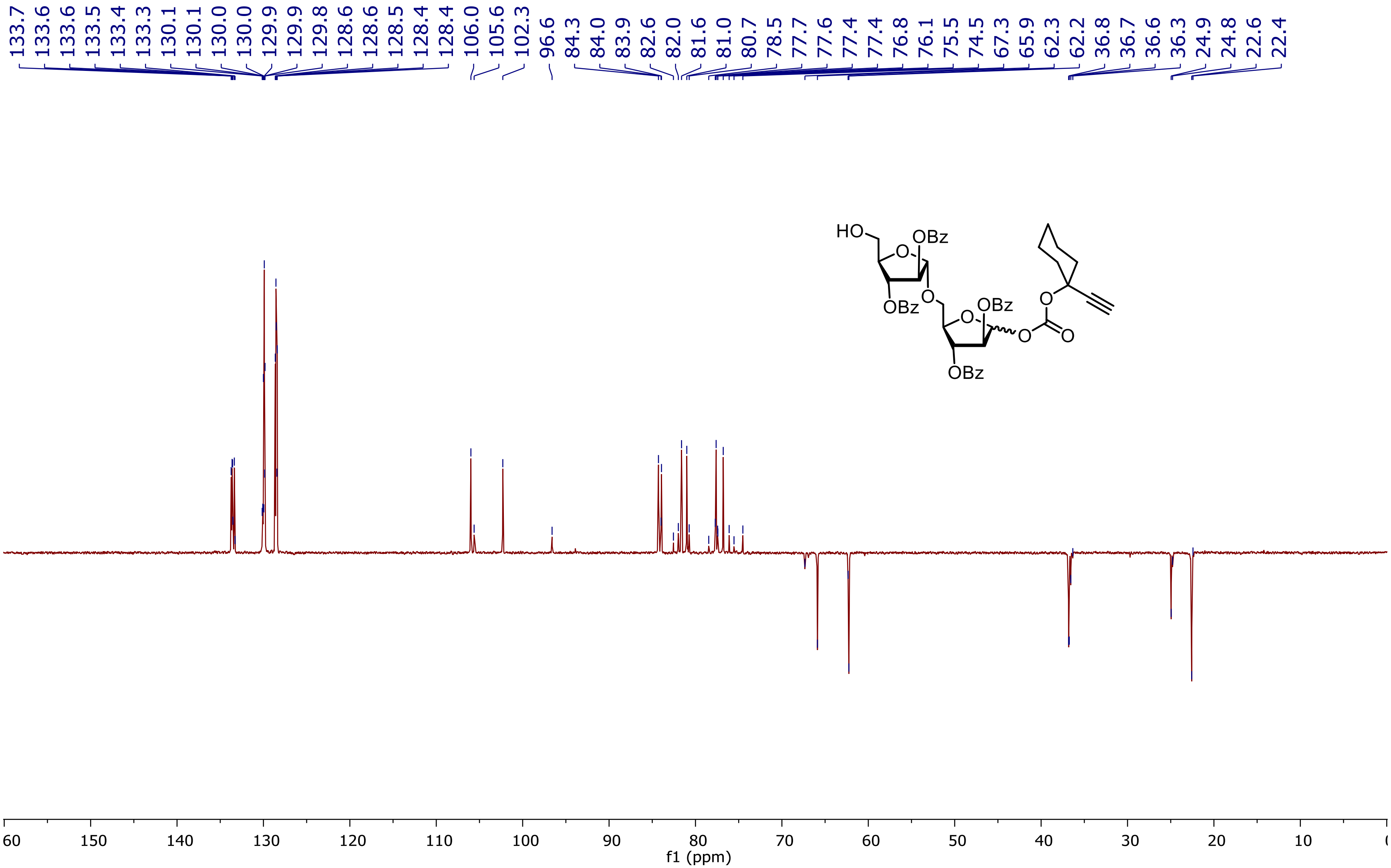

Supplementary Figure S18a. ^{1}H NMR Spectrum (400 MHz, $CDCl_3$) of compound **6f**

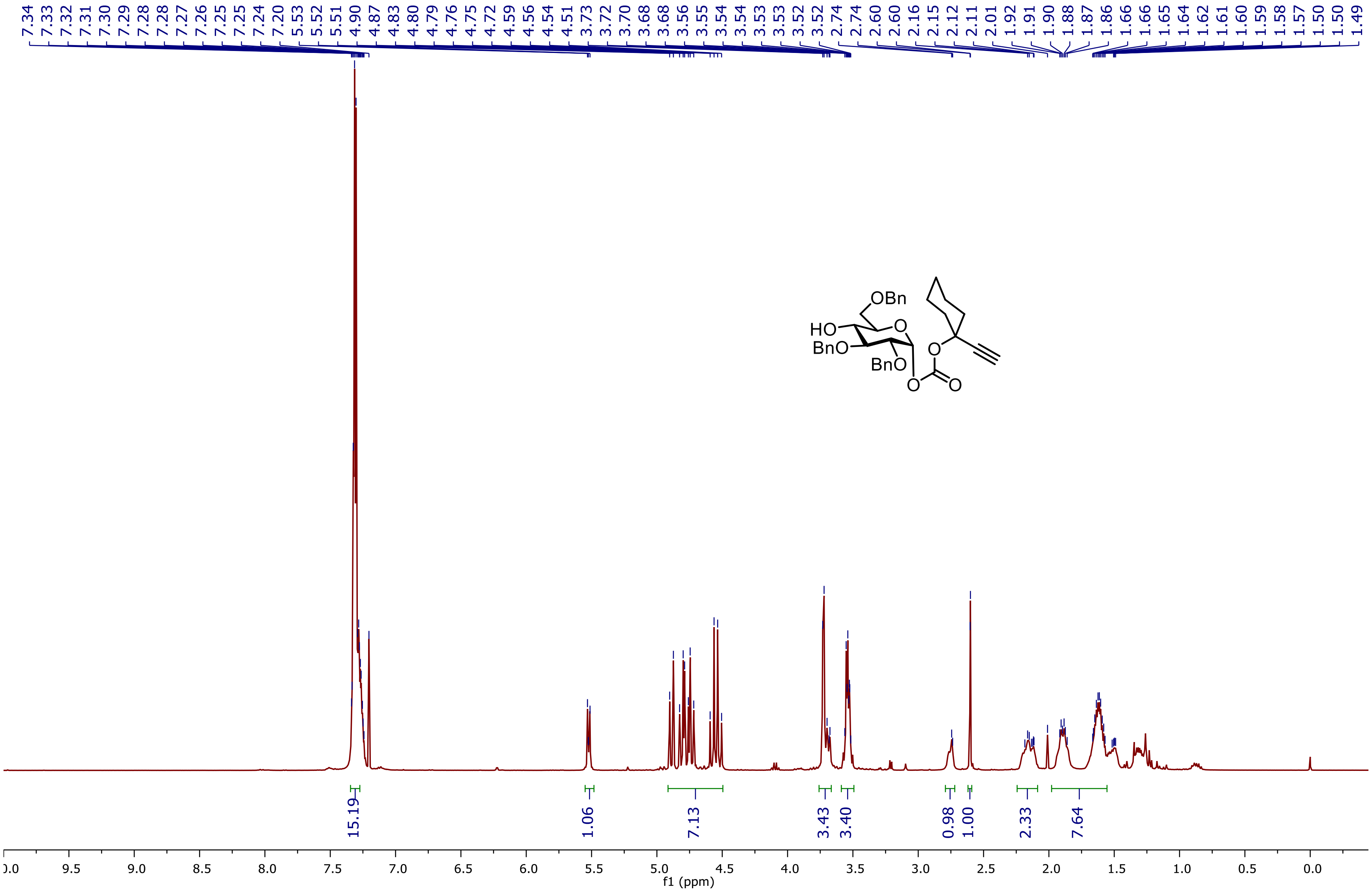

Supplementary Figure S18b. ^{13}C NMR Spectrum (101 MHz, $CDCl_3$) of compound **6f**

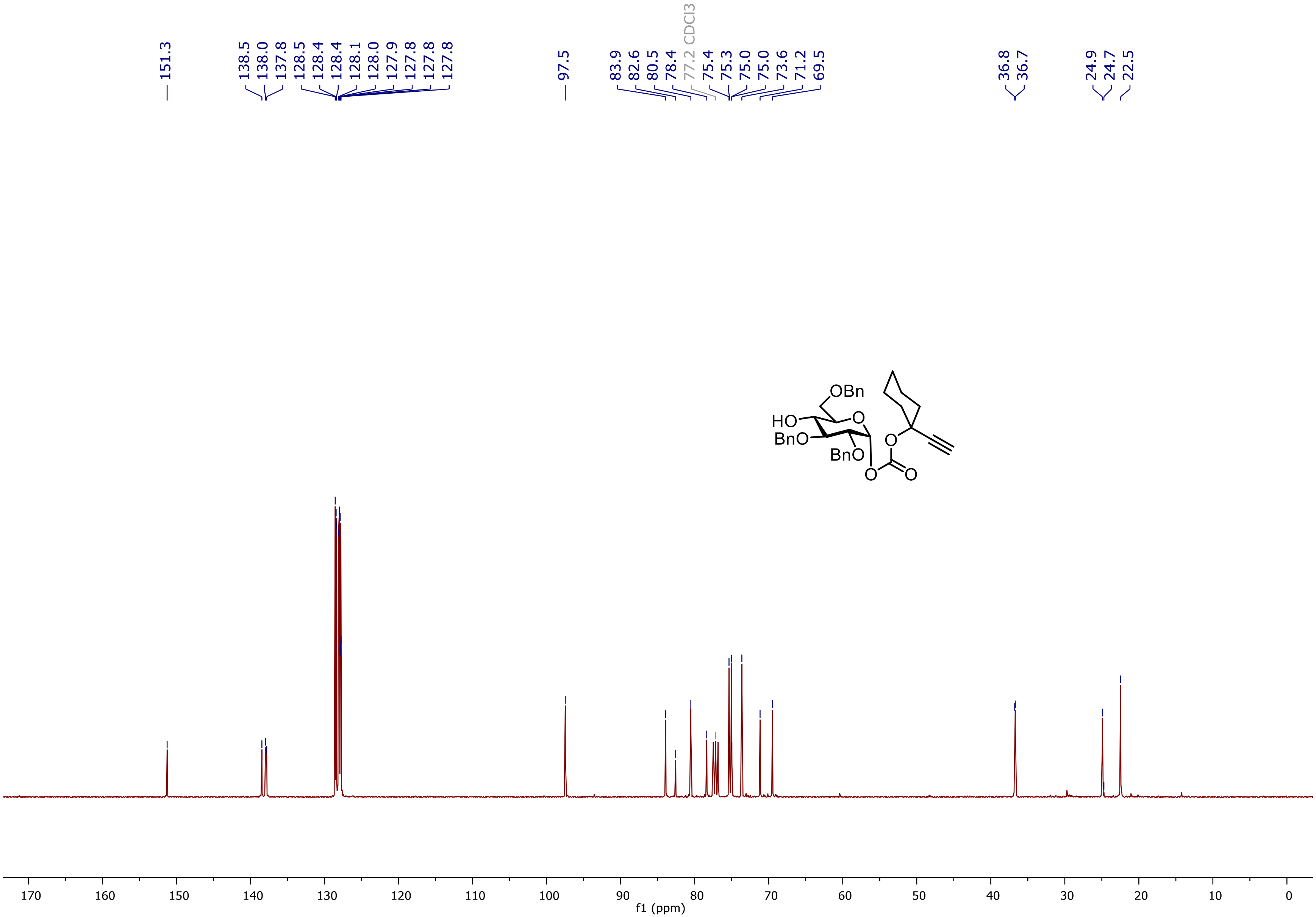

Supplementary Figure S18c. DEPT NMR Spectrum (101 MHz, $CDCl_3$) of compound **6f**

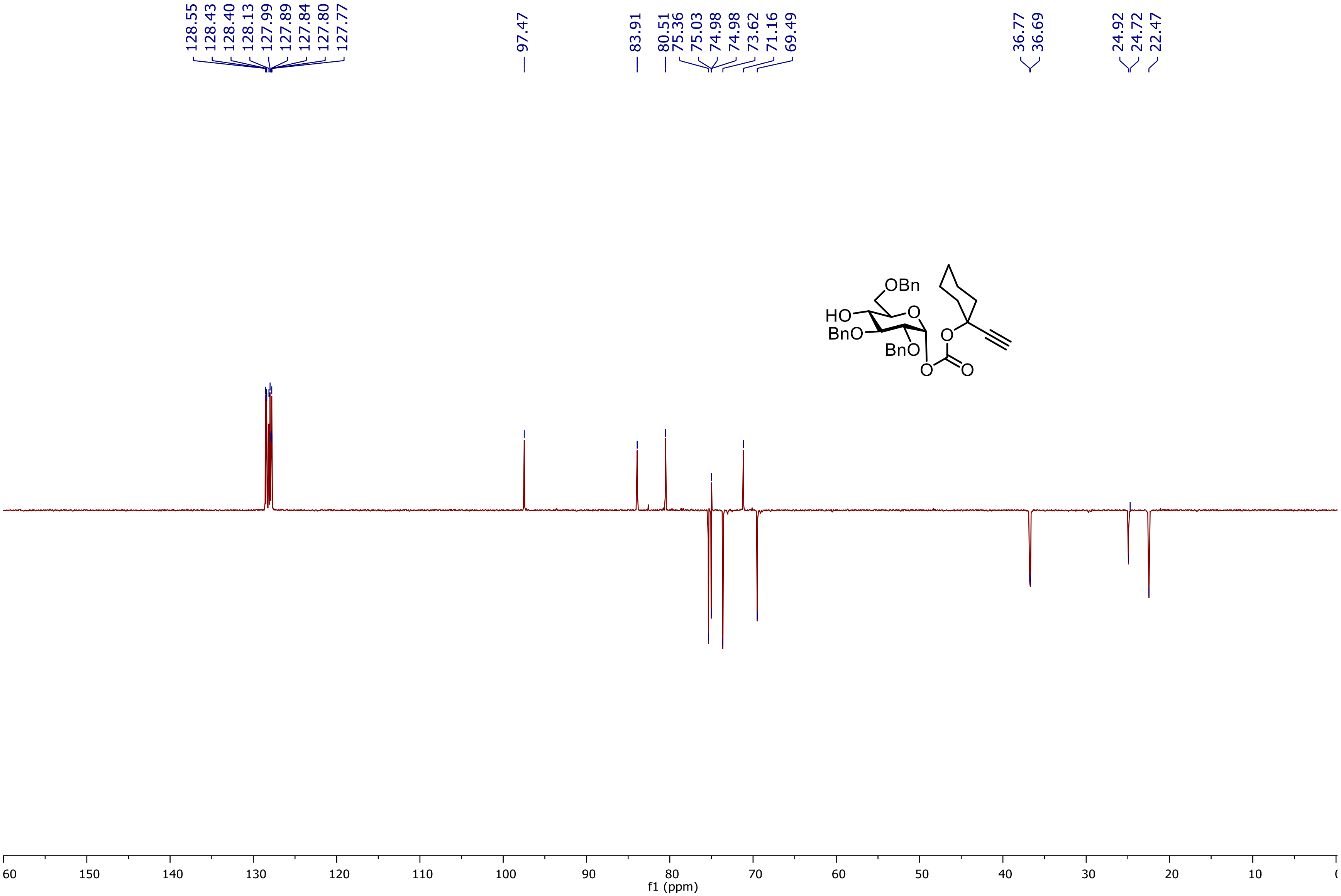

Supplementary Figure S19a. ^{1}H NMR Spectrum (400 MHz, $CDCl_3$) of compound **S25**

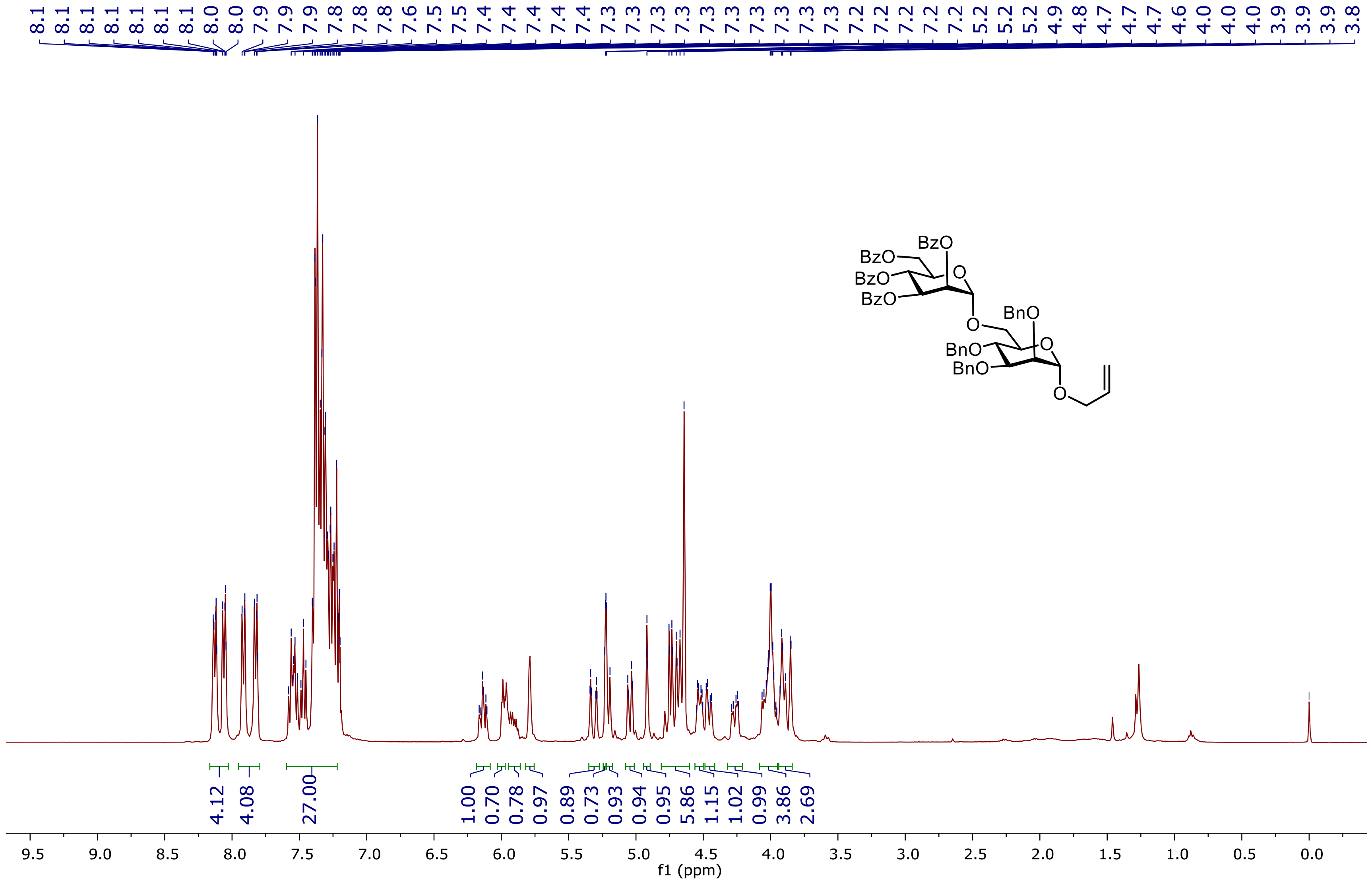

Supplementary Figure S19b. ^{13}C NMR Spectrum (101 MHz, $CDCl_3$) of compound **S25**

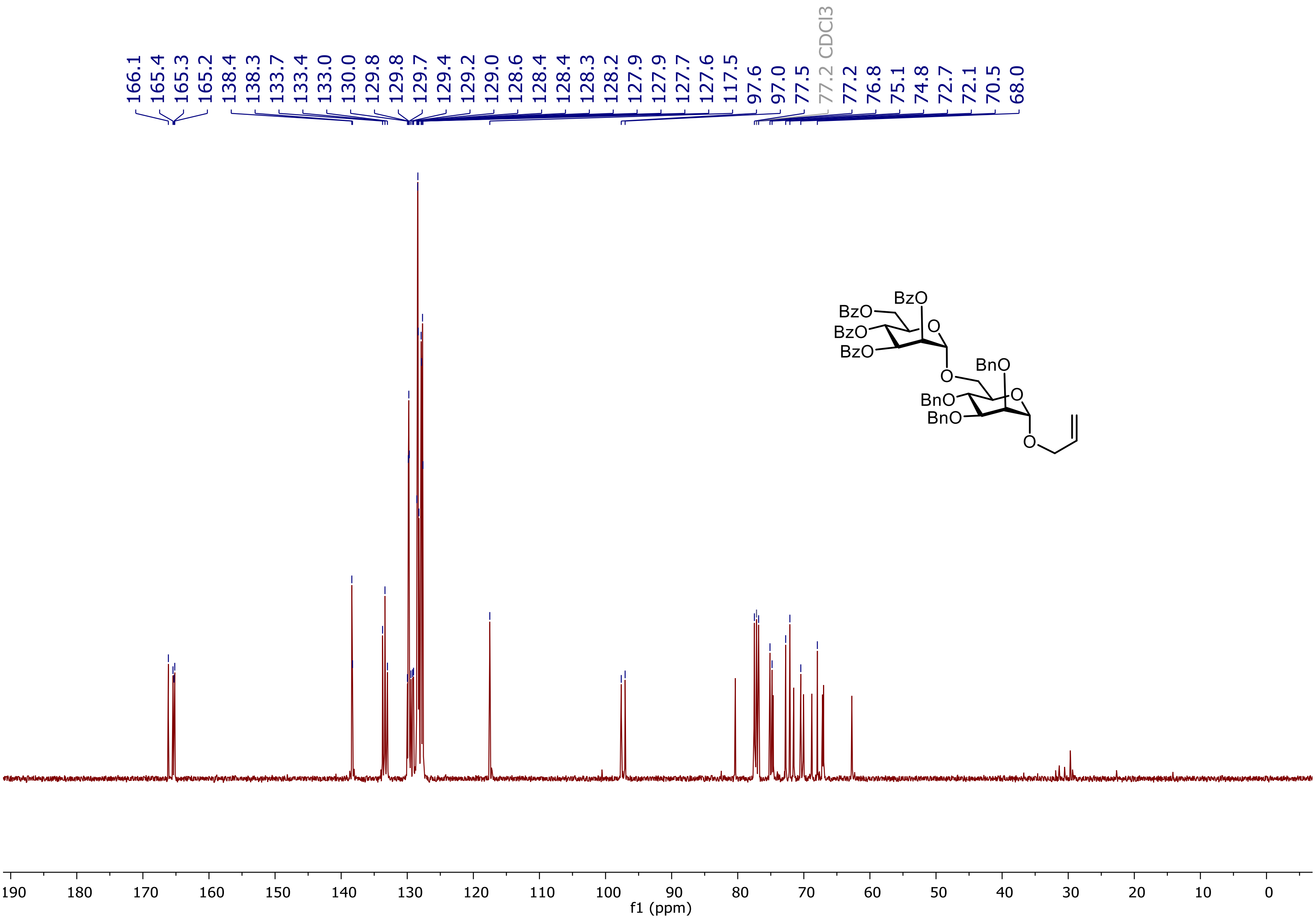

Supplementary Figure S19c. DEPT NMR Spectrum (101 MHz, $CDCl_3$) of compound **S25**

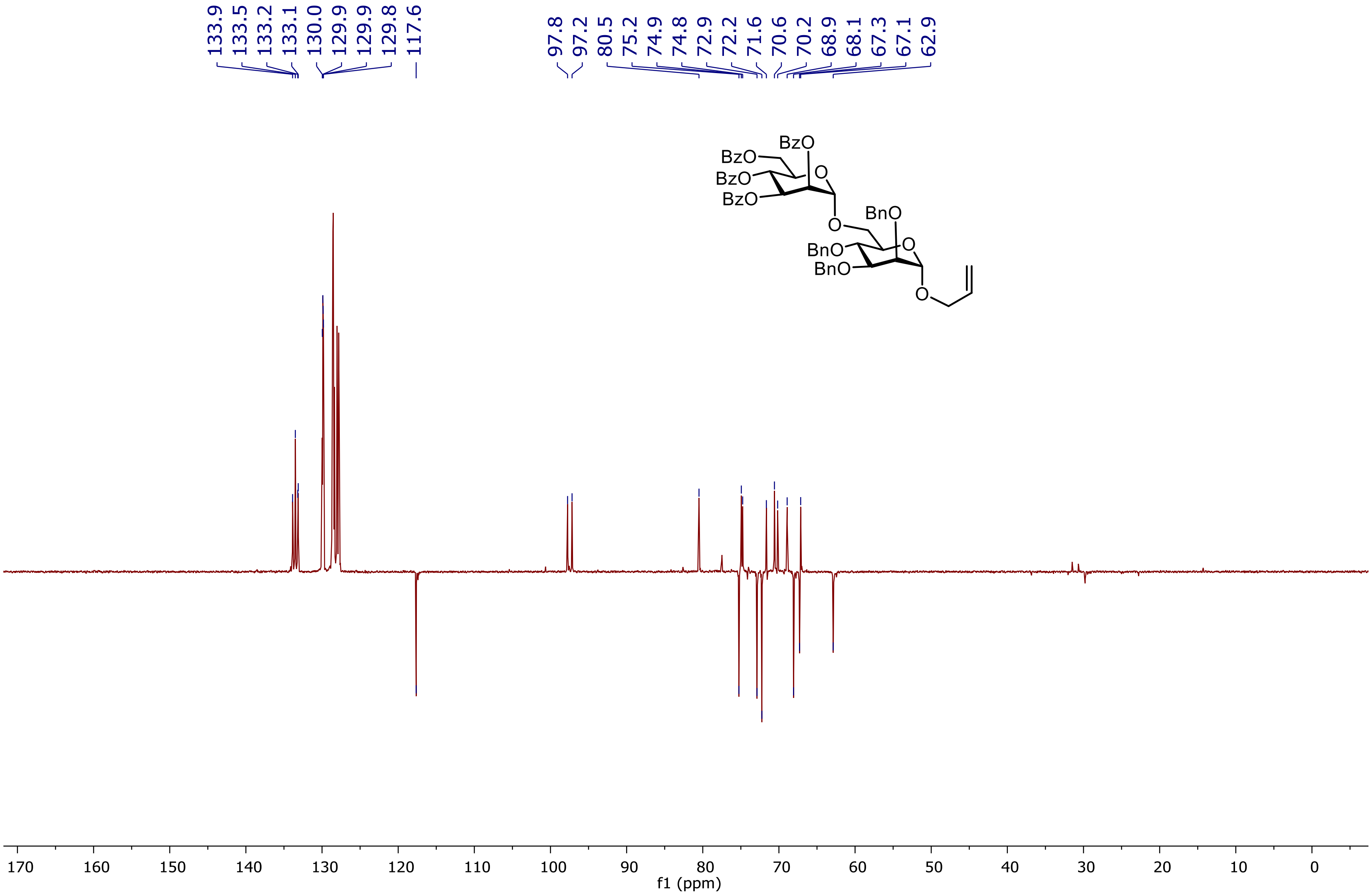

Supplementary Figure S20a. ^{1}H NMR Spectrum (400 MHz, $CDCl_3$) of compound **18a**

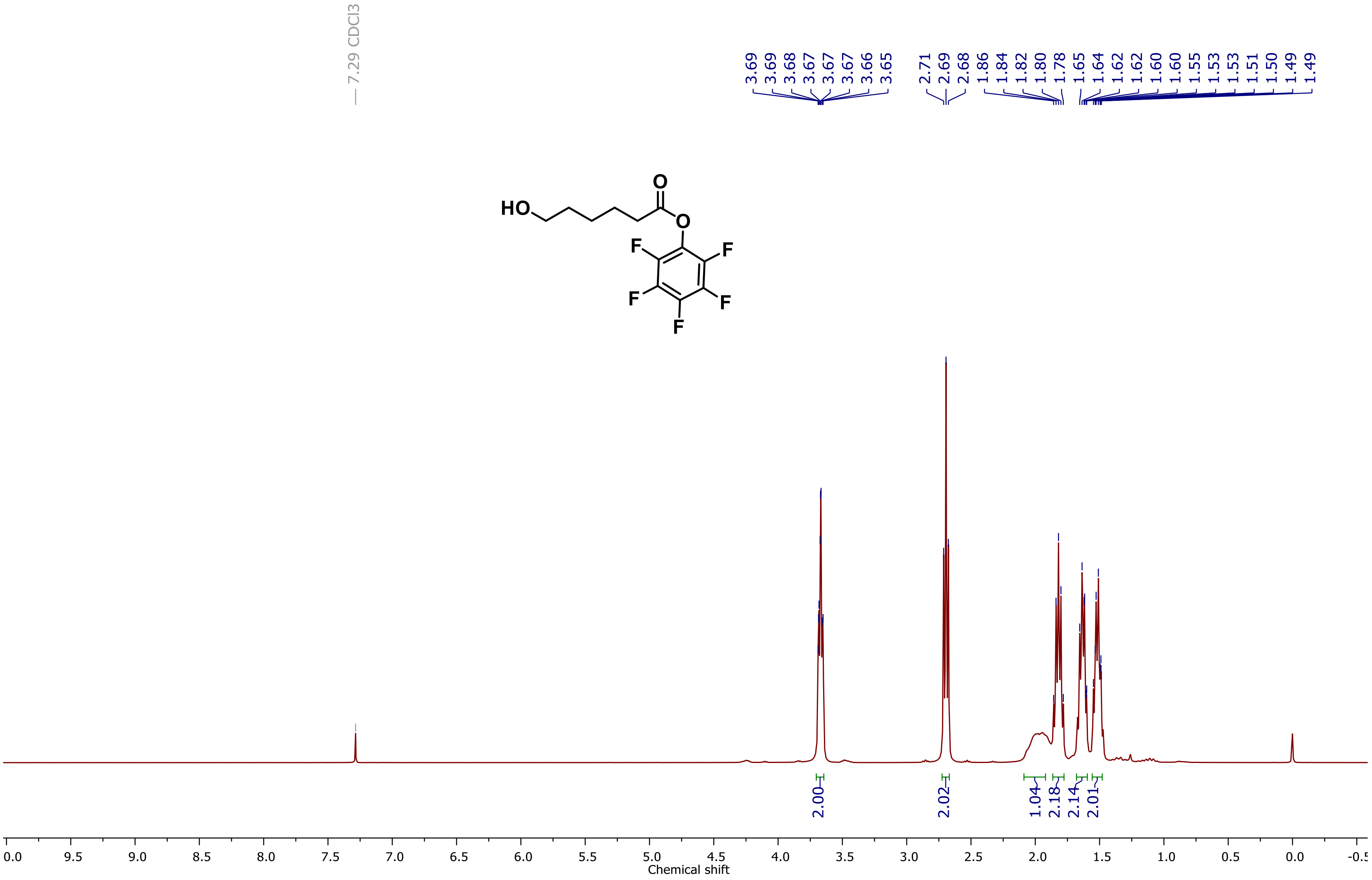

Supplementary Figure S20b. ^{19}F NMR Spectrum (400 MHz, $CDCl_3$) of compound **18a**

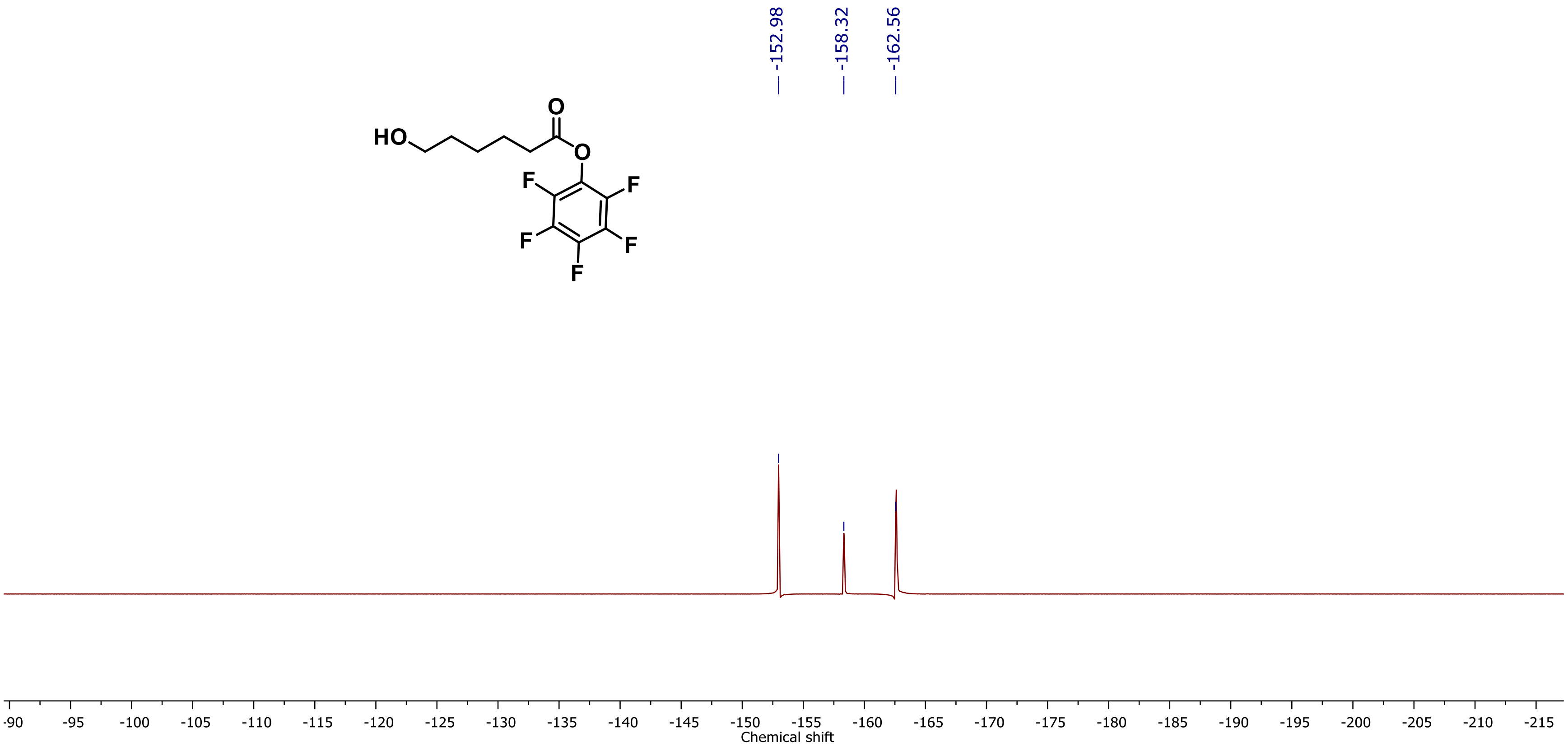

Supplementary Figure S20c. ^{13}C NMR Spectrum (101 MHz, $CDCl_3$) of compound **18a**

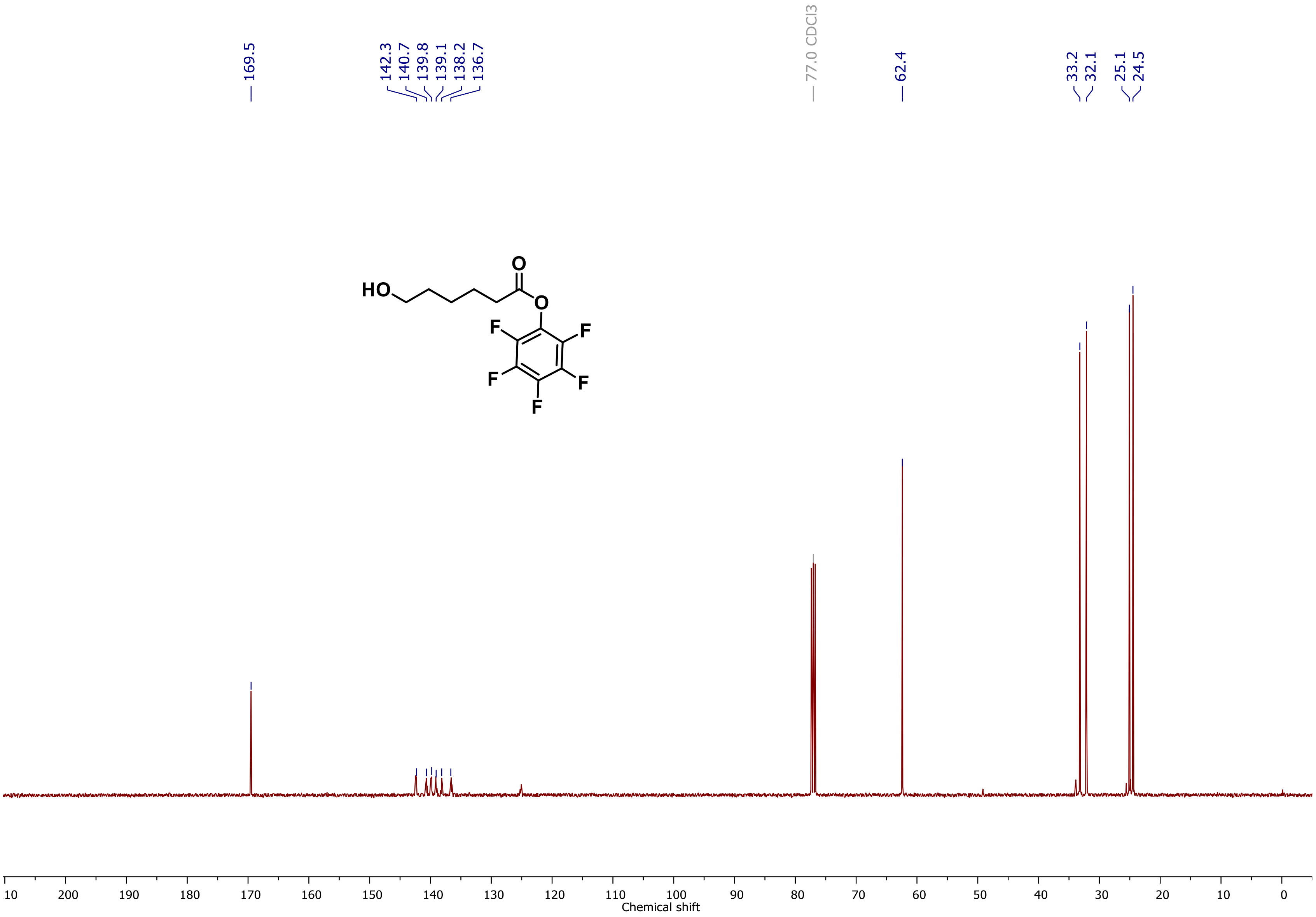

Supplementary Figure S21a. ^{1}H NMR Spectrum (400MHz, $CDCl_3$) of compound **18c**

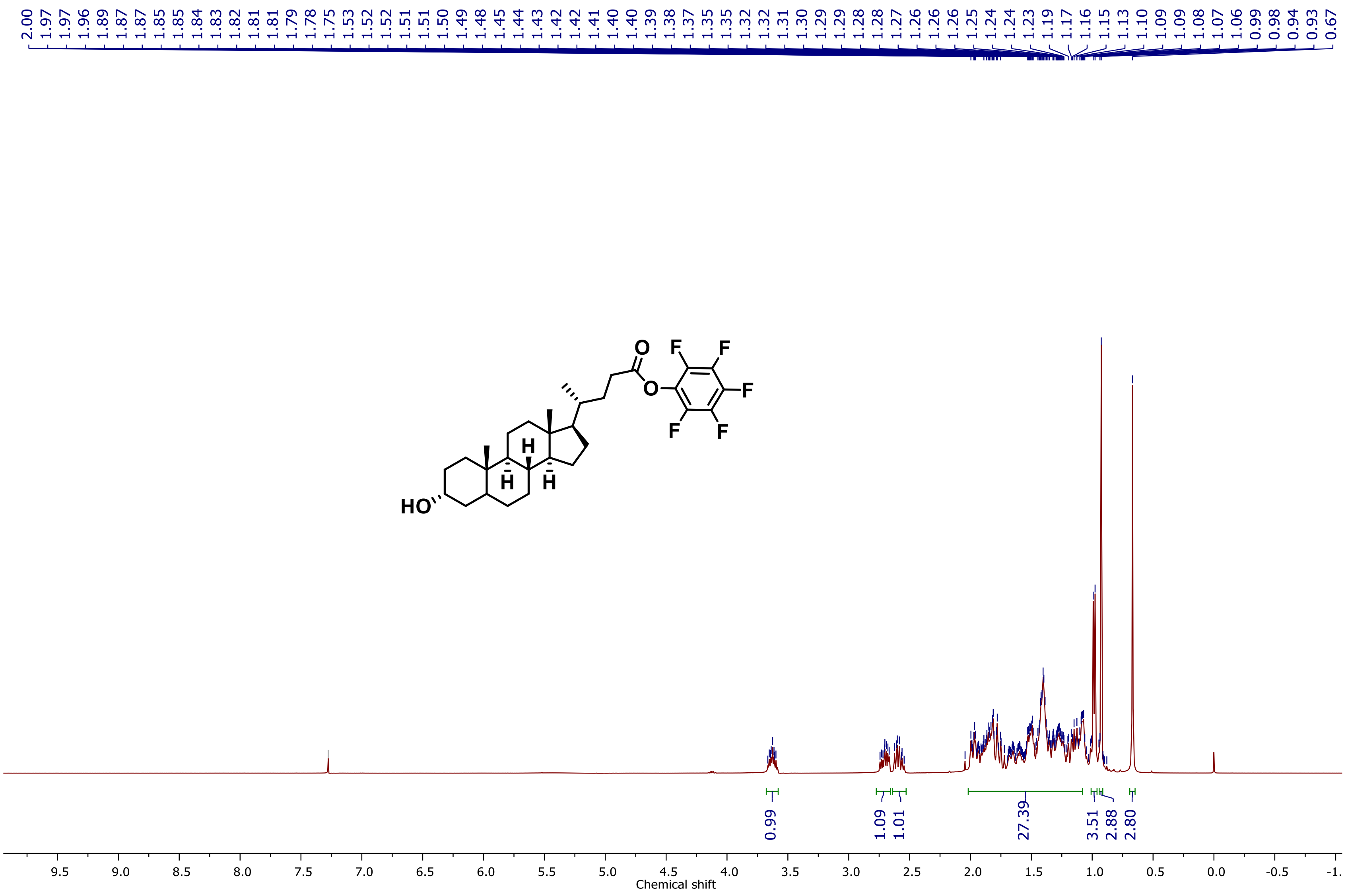

Supplementary Figure S21b. ^{13}C NMR Spectrum (400MHz, $CDCl_3$) of compound **18c**

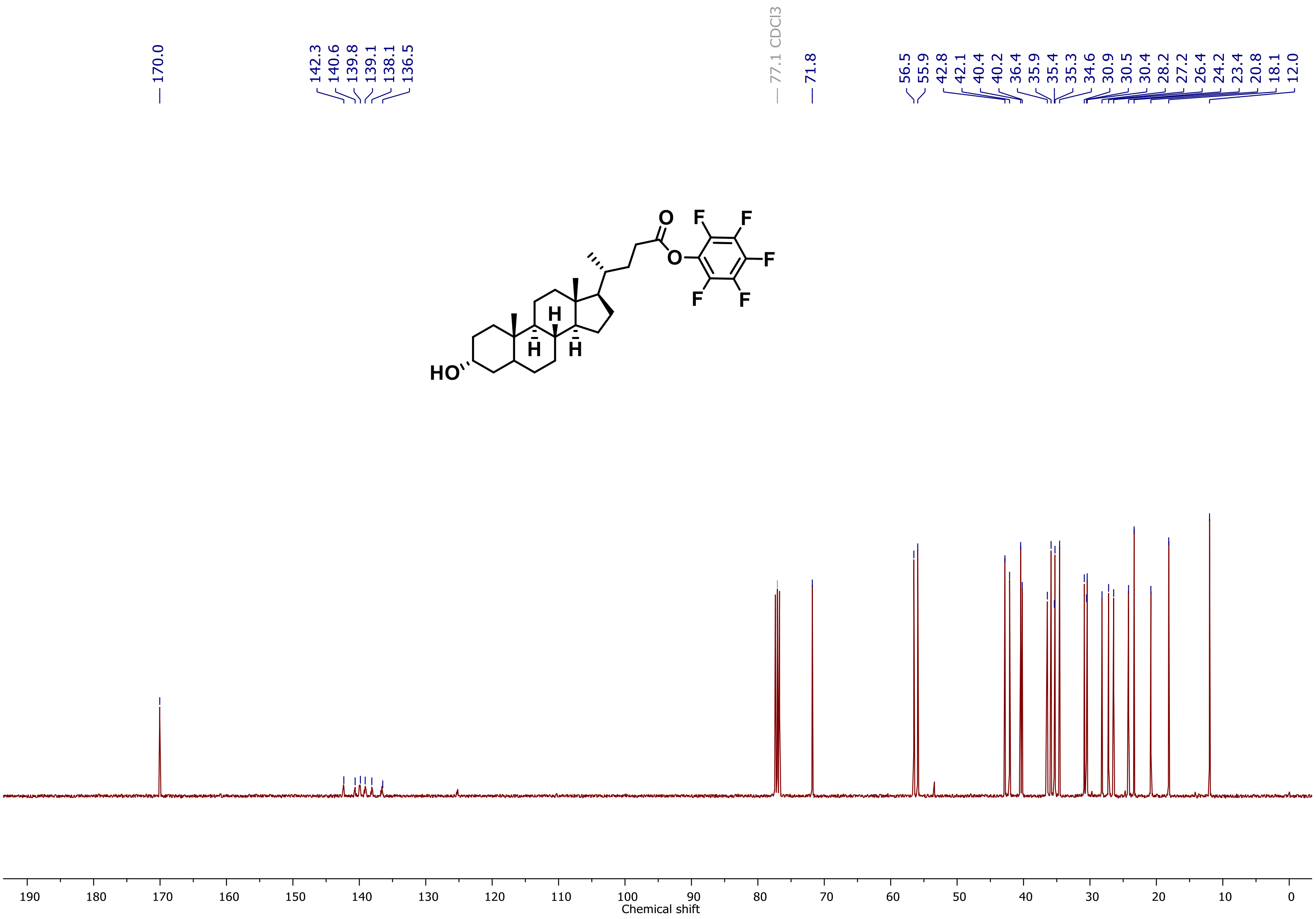

Supplementary Figure S21c. ^{19}F NMR Spectrum (400 MHz, $CDCl_3$) of compound **18c**

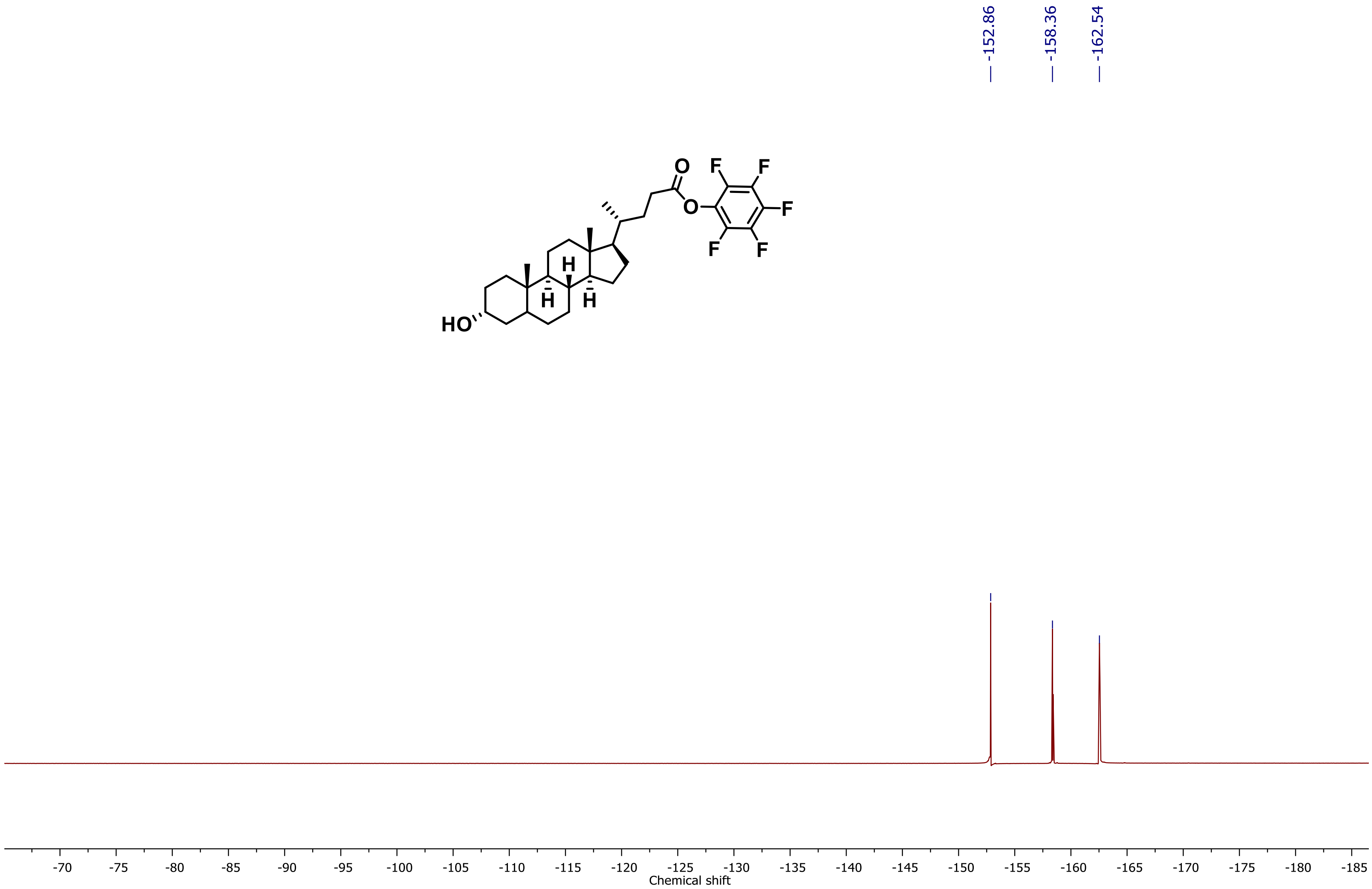

Supplementary Figure S22a. ^{1}H NMR Spectrum (400MHz, $CDCl_3$) of compound **18b**

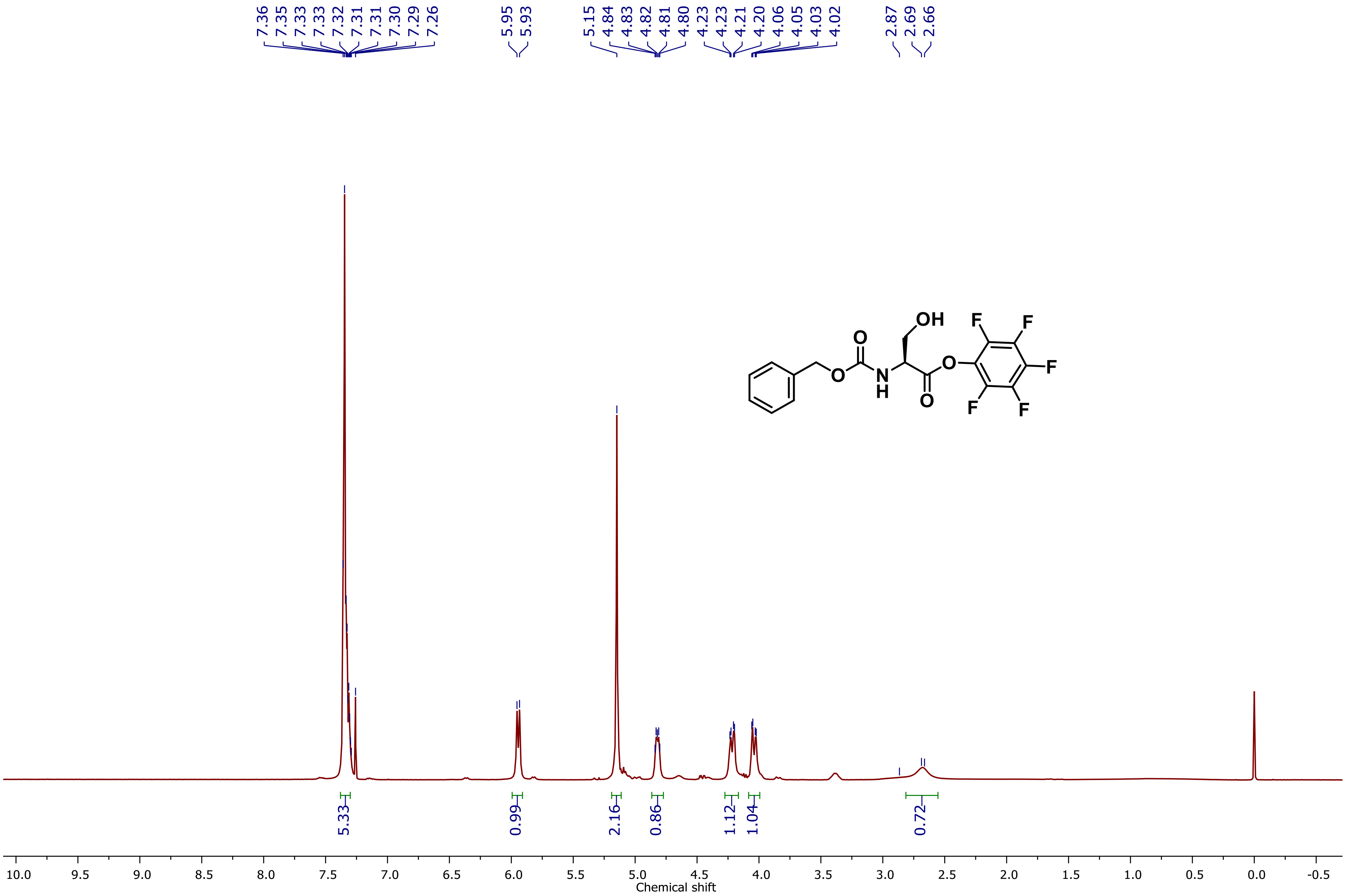

Supplementary Figure S22b. ^{19}F NMR Spectrum (400 MHz, $CDCl_3$) of compound **18b**

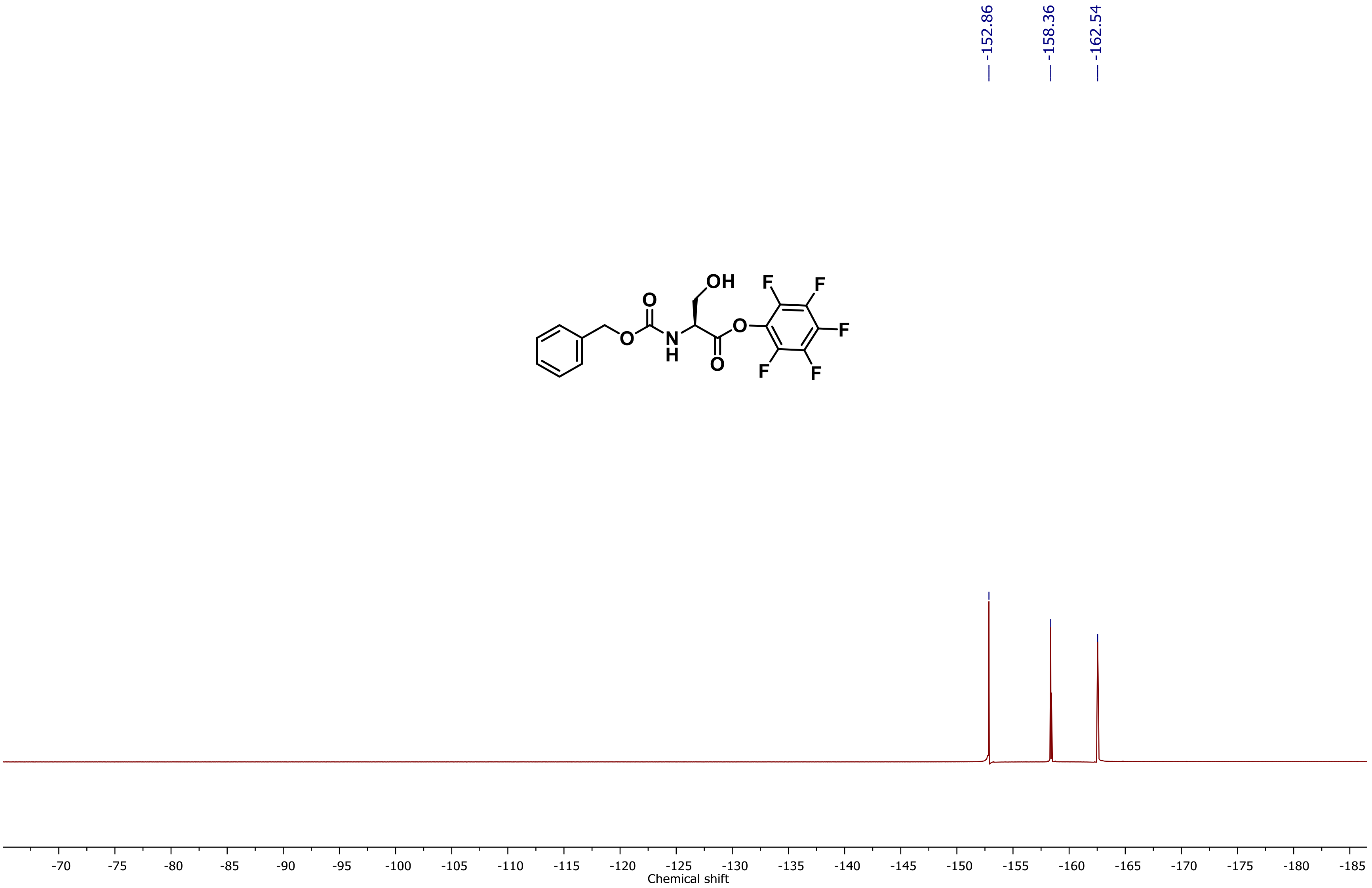

Supplementary Figure S23a. ^{1}H NMR Spectrum (400MHz, $CDCl_3$) of compound **17**

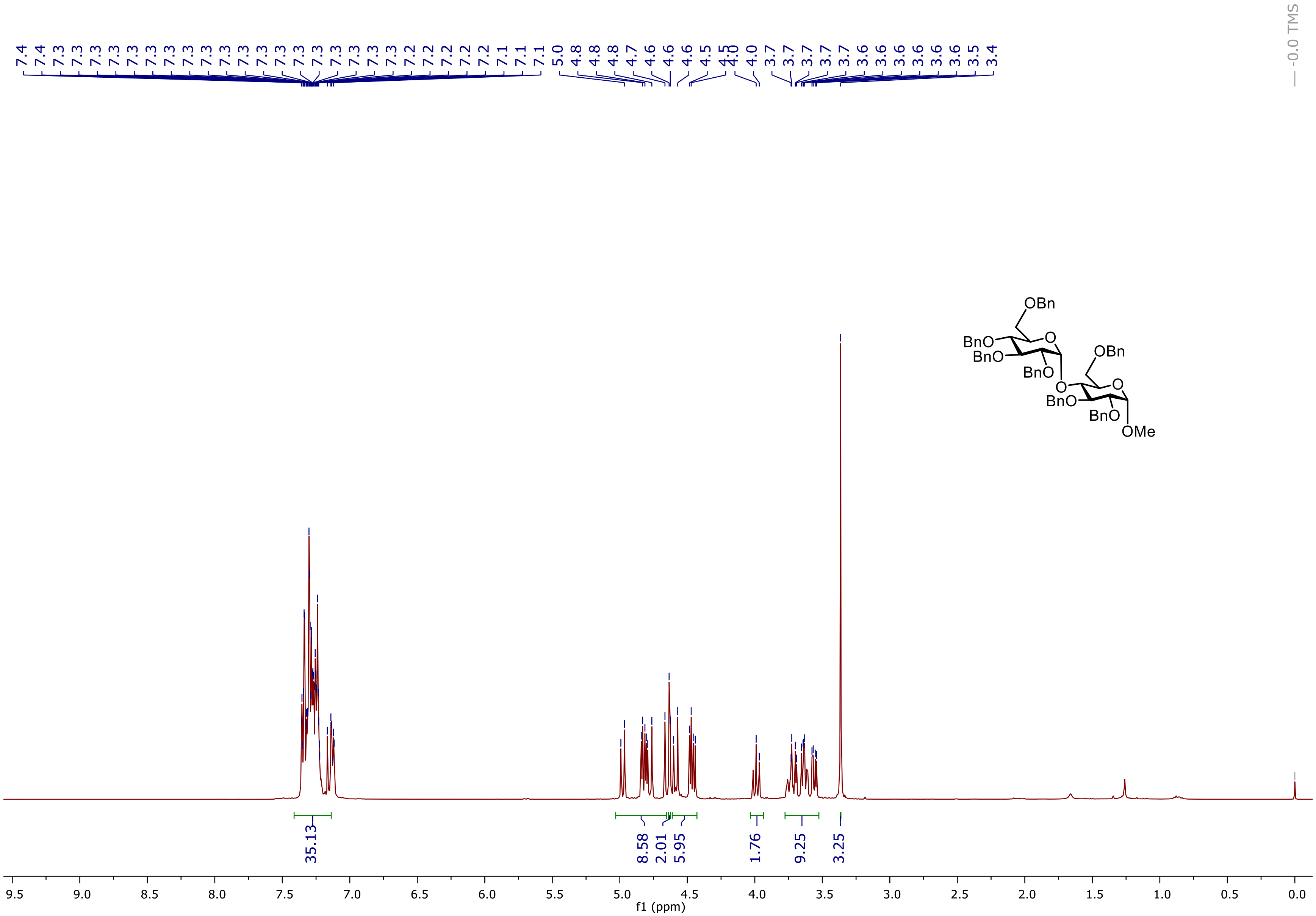

Supplementary Figure S23b. ^{13}C NMR Spectrum (101MHz, $CDCl_3$) of compound **17**

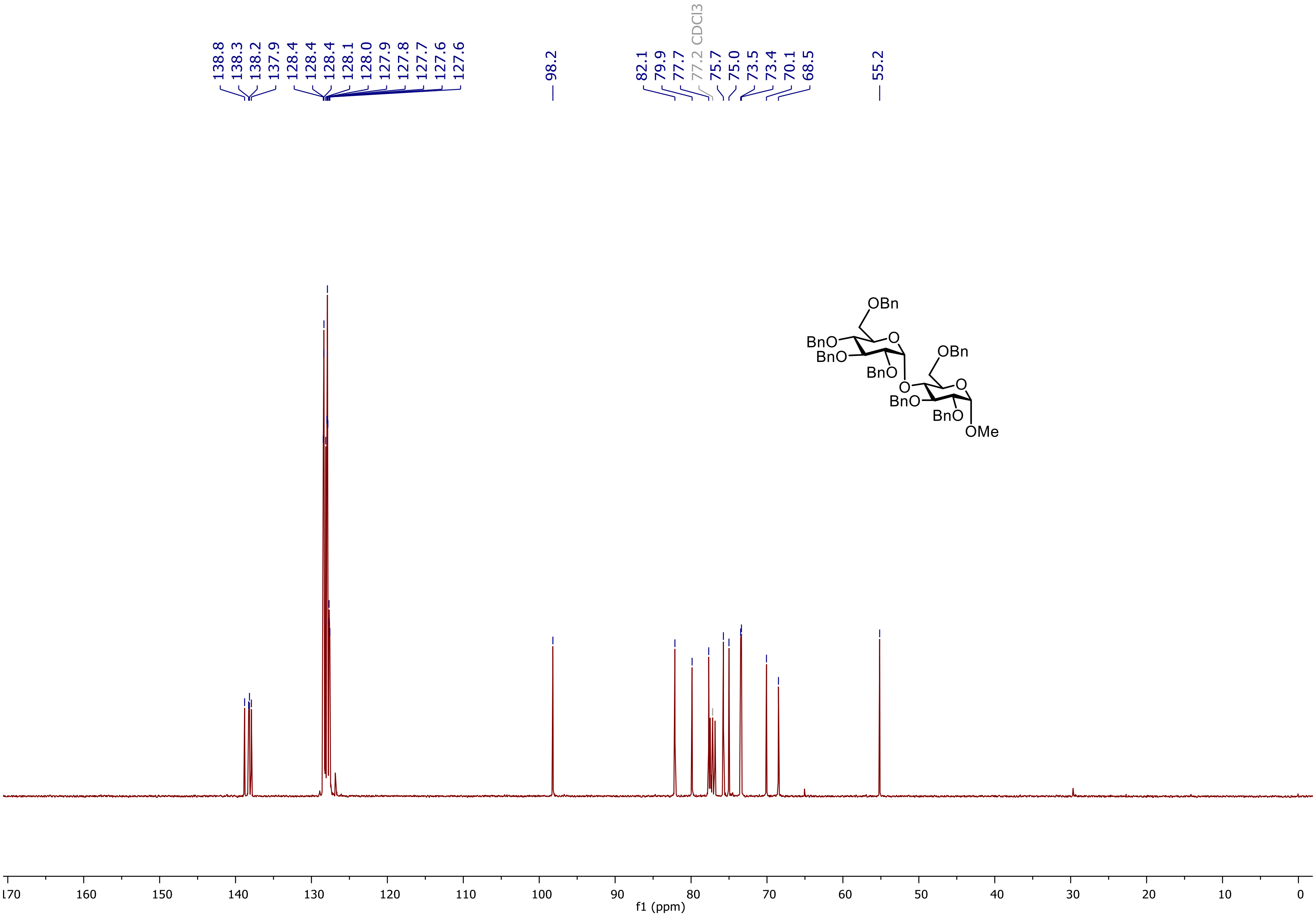

Supplementary Figure S23c. DEPT NMR Spectrum (101 MHz, $CDCl_3$) of compound **17**

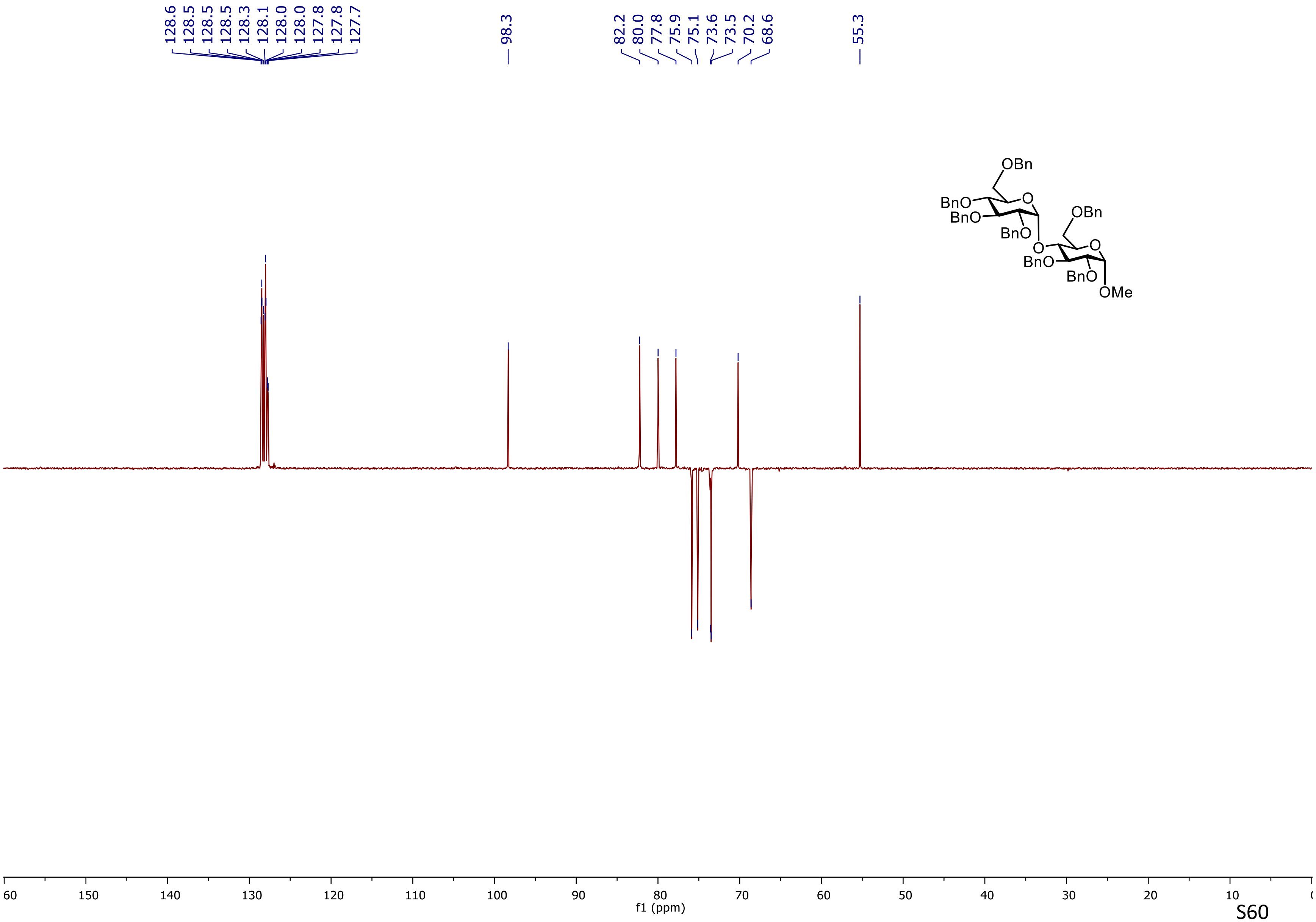

Supplementary Figure S24a. ^{1}H NMR Spectrum (400MHz, $CDCl_3$) of compound **19a**

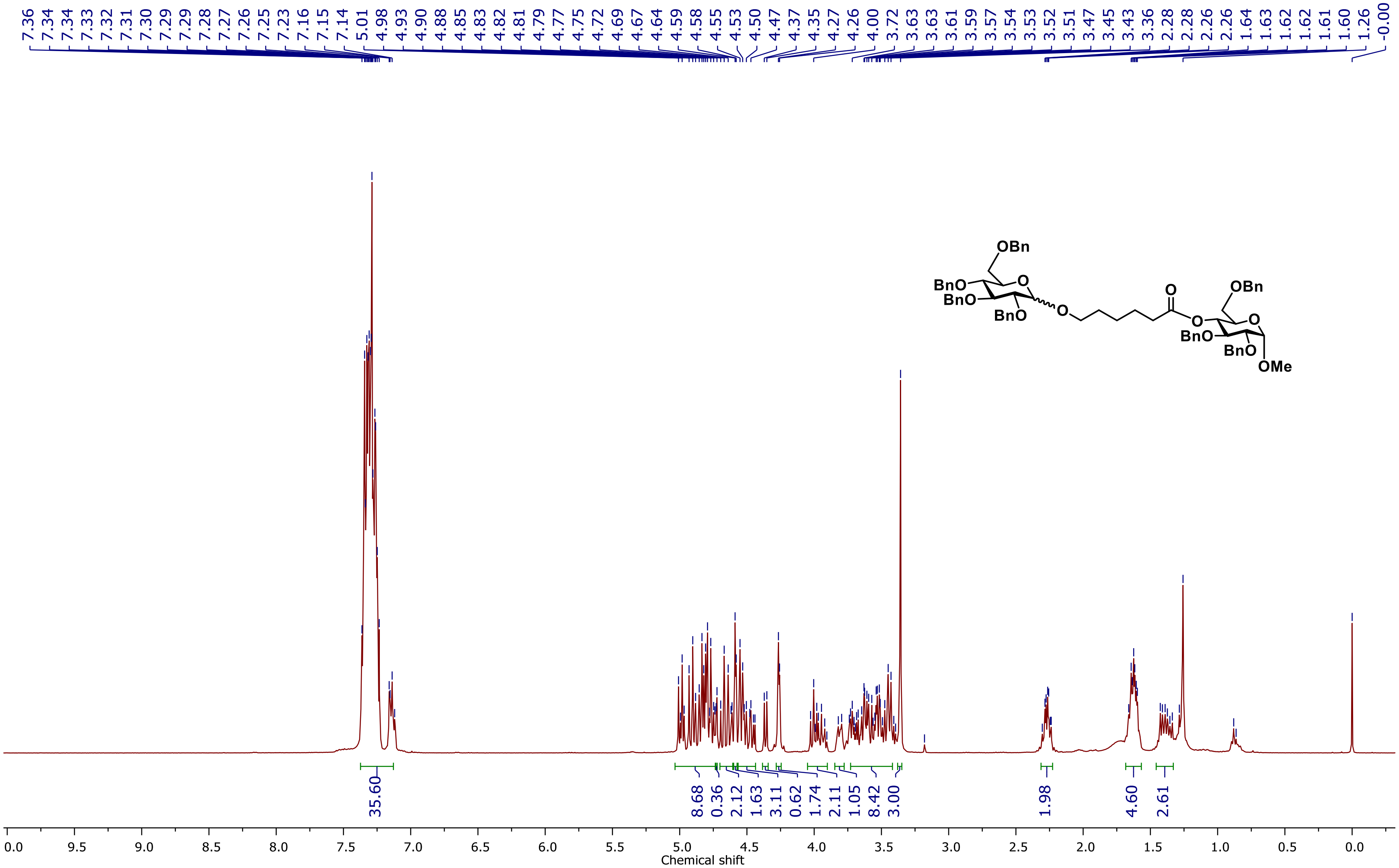

Supplementary Figure S24b. ^{13}C NMR Spectrum (101MHz, $CDCl_3$) of compound **19a**

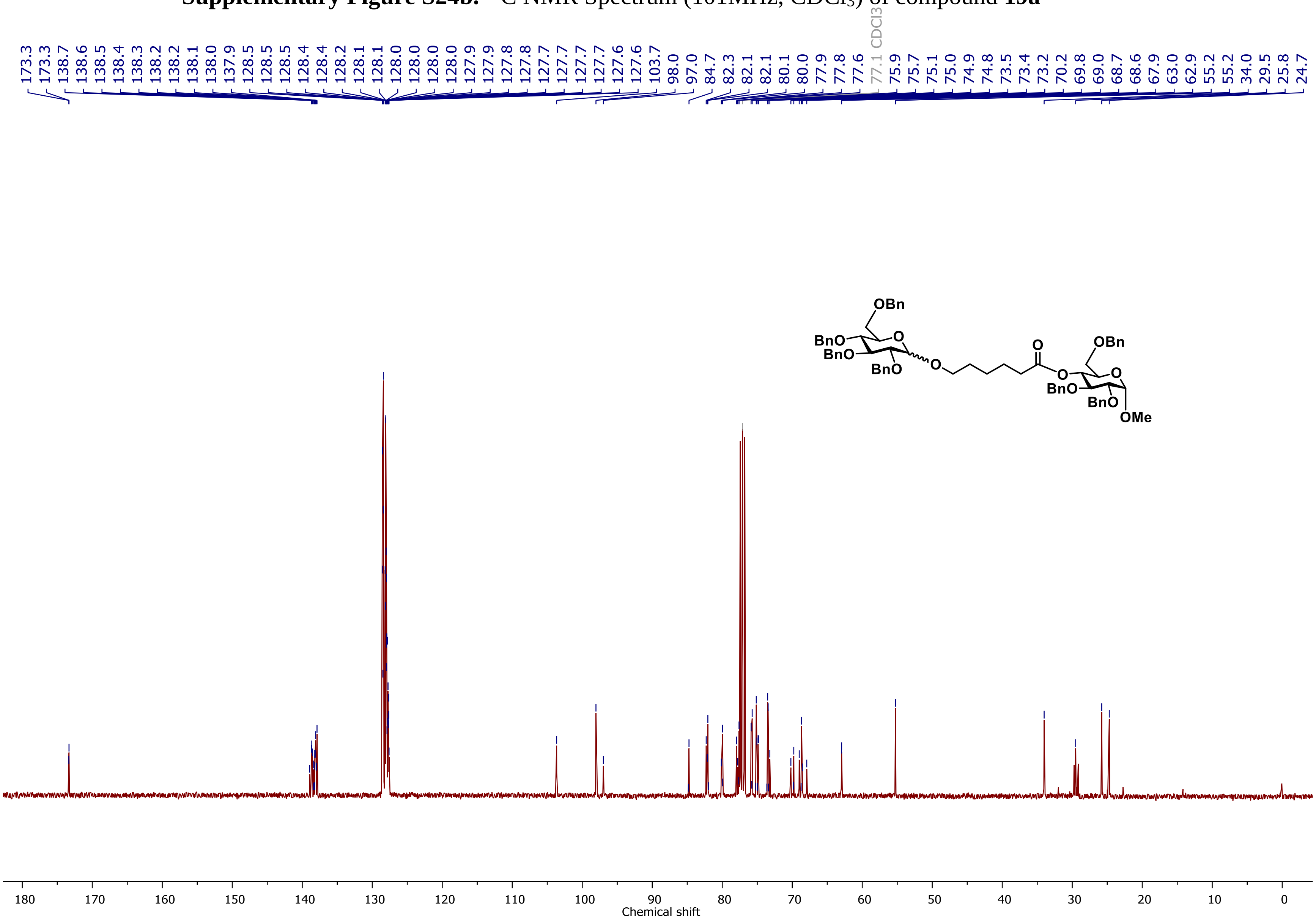

Supplementary Figure S25c. DEPT NMR Spectrum (101 MHz, $CDCl_3$) of compound **19a**

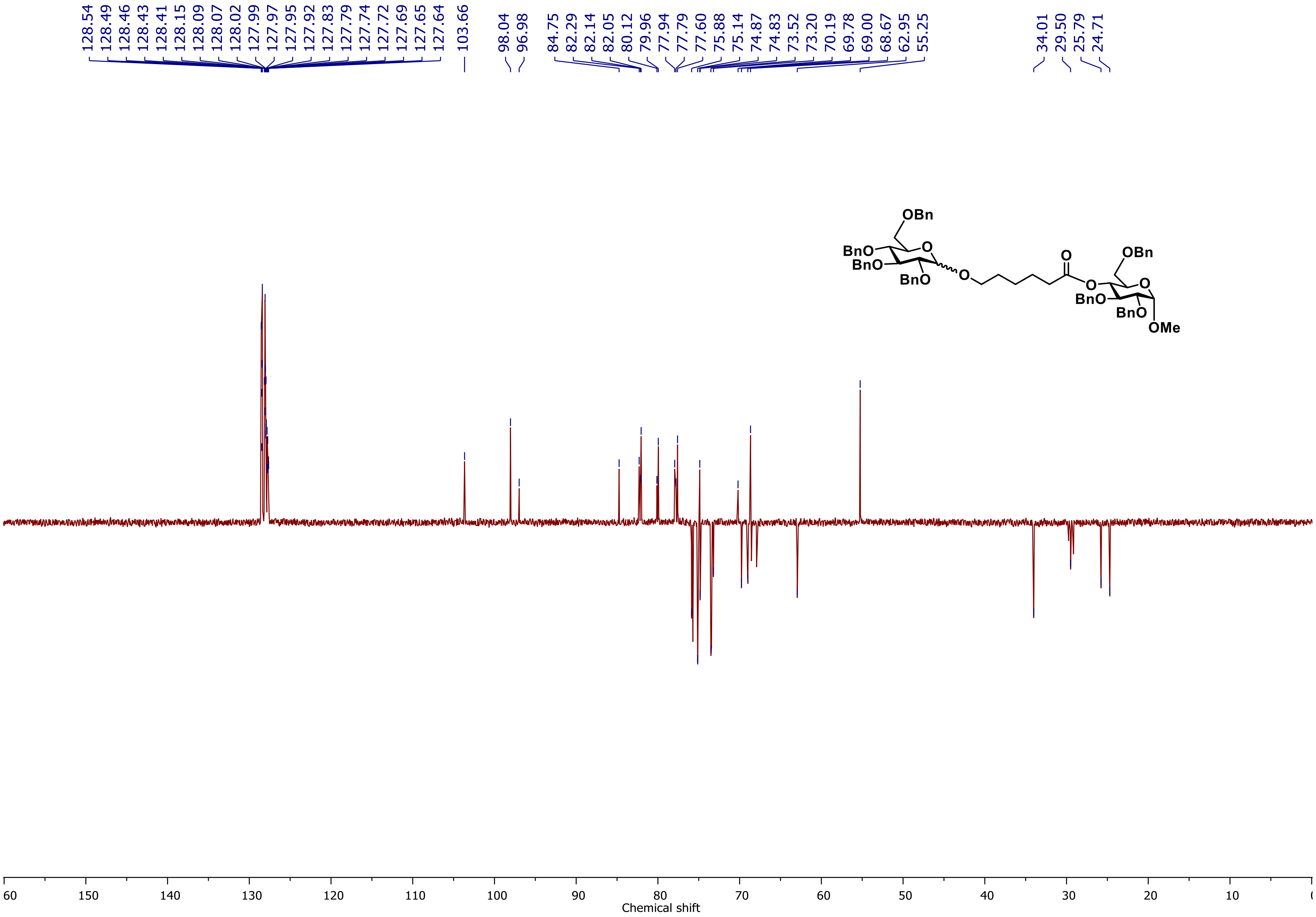

Supplementary Figure S26a. ^{1}H NMR Spectrum (400MHz, $CDCl_3$) of compound **19c**

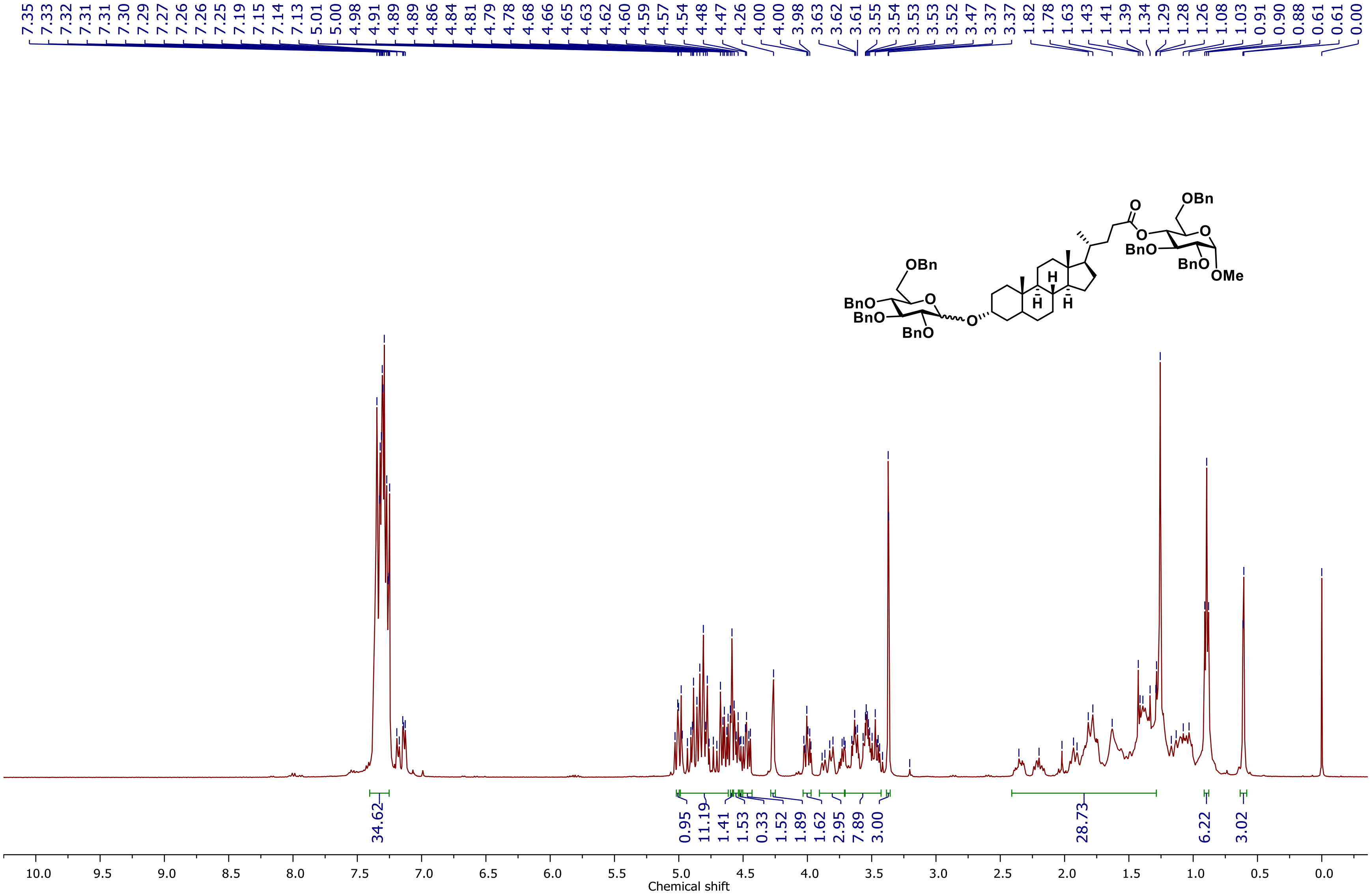

Supplementary Figure S26b. ^{13}C NMR Spectrum (101MHz, $CDCl_3$) of compound **19c**

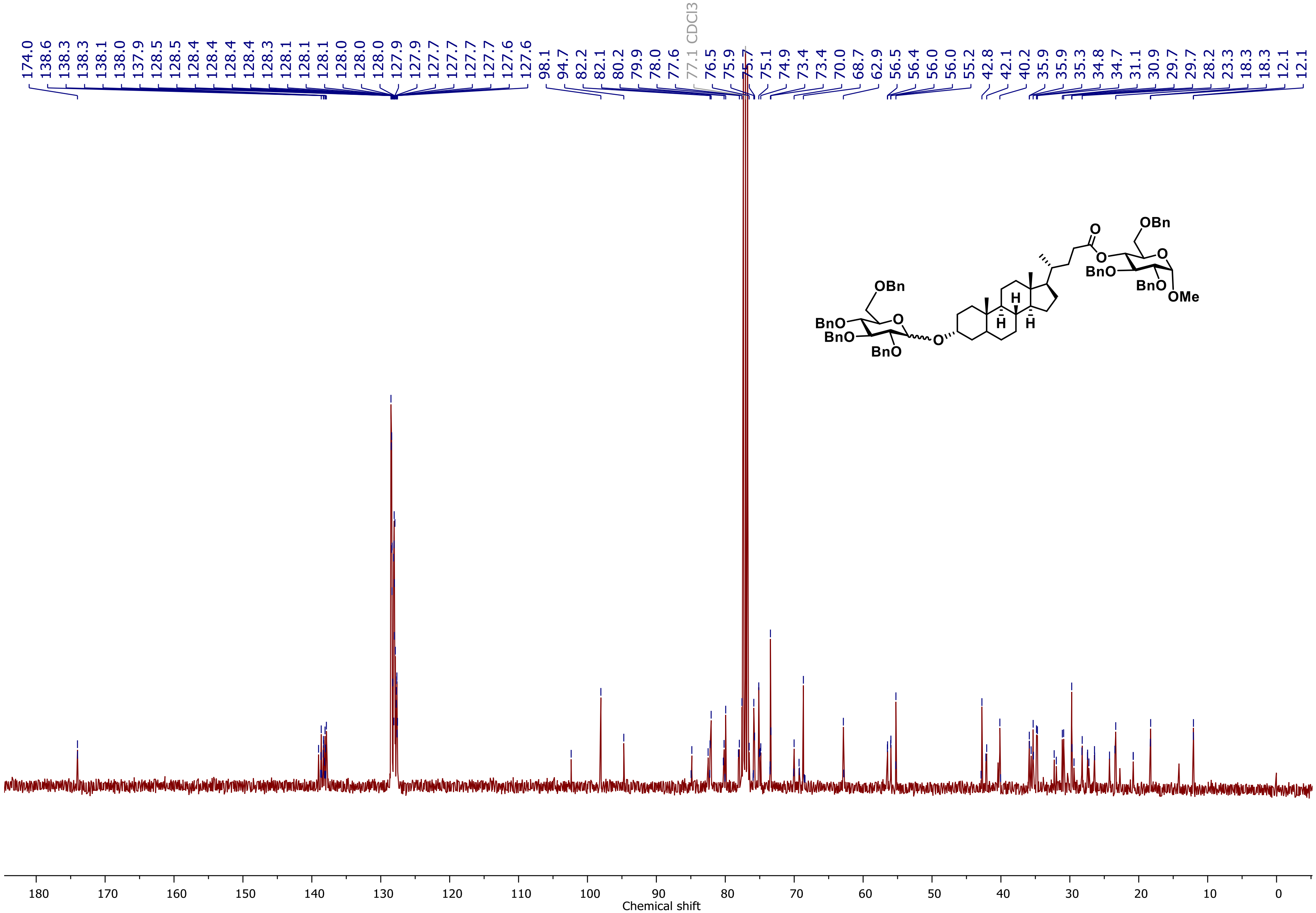

Supplementary Figure S26c. DEPT NMR Spectrum (101 MHz, $CDCl_3$) of compound **19c**

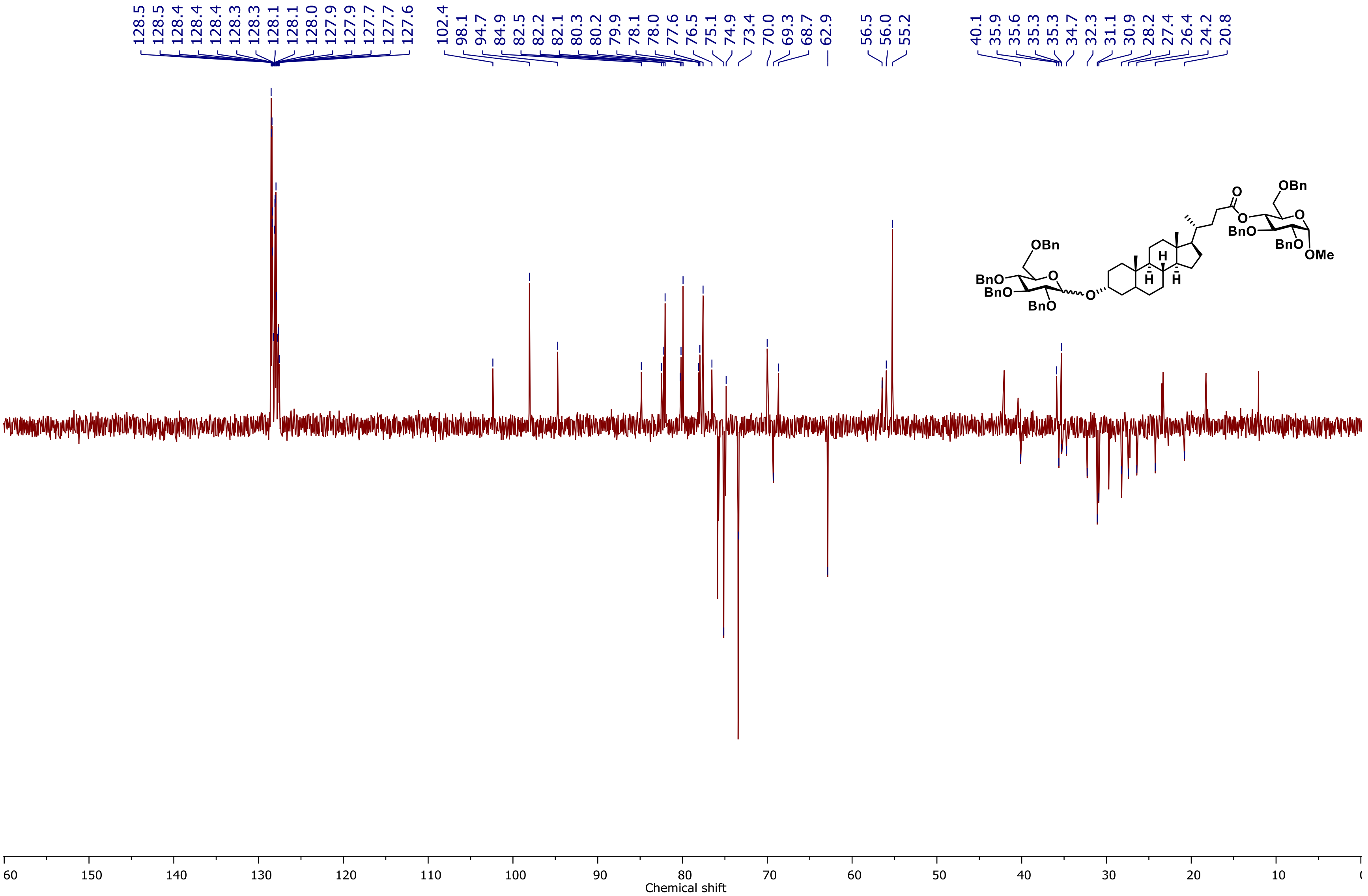

Supplementary Figure S27a. ^{1}H NMR Spectrum (400MHz, $CDCl_3$) of compound **19b**

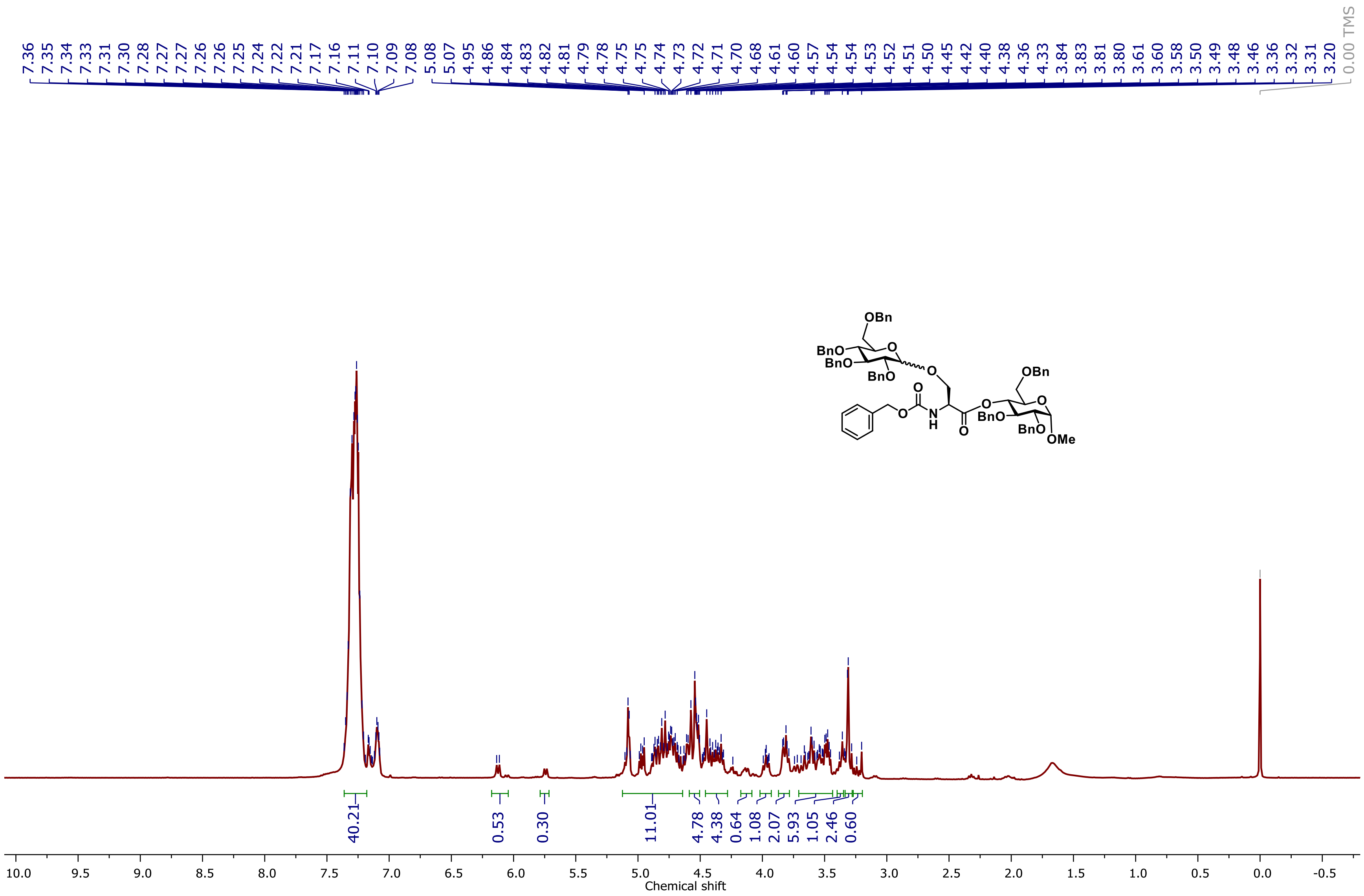

Supplementary Figure S27b. ^{13}C NMR Spectrum (101MHz, $CDCl_3$) of compound **19b**

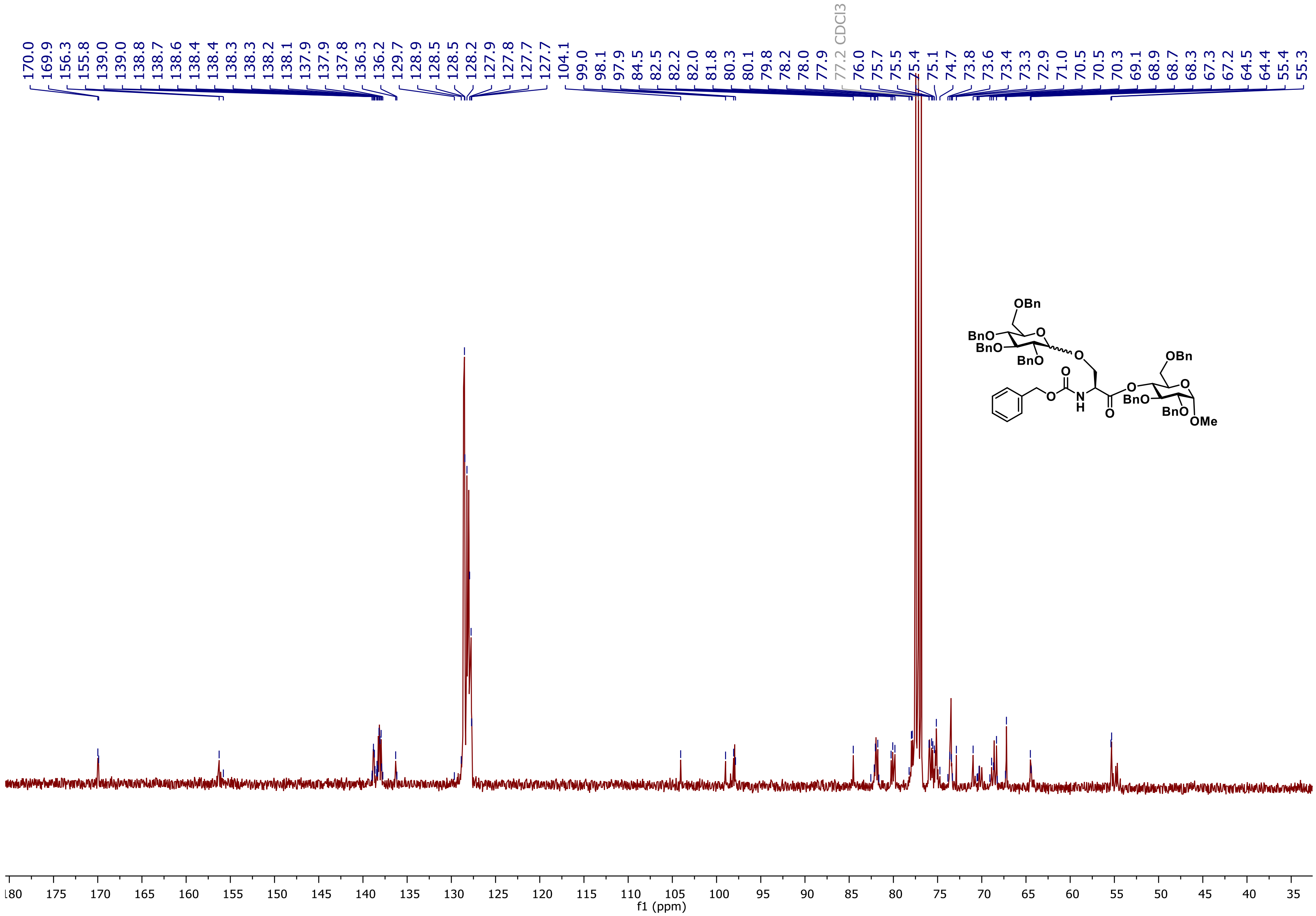

Supplementary Figure S27c. DEPT NMR Spectrum (101 MHz, $CDCl_3$) of compound **19b**

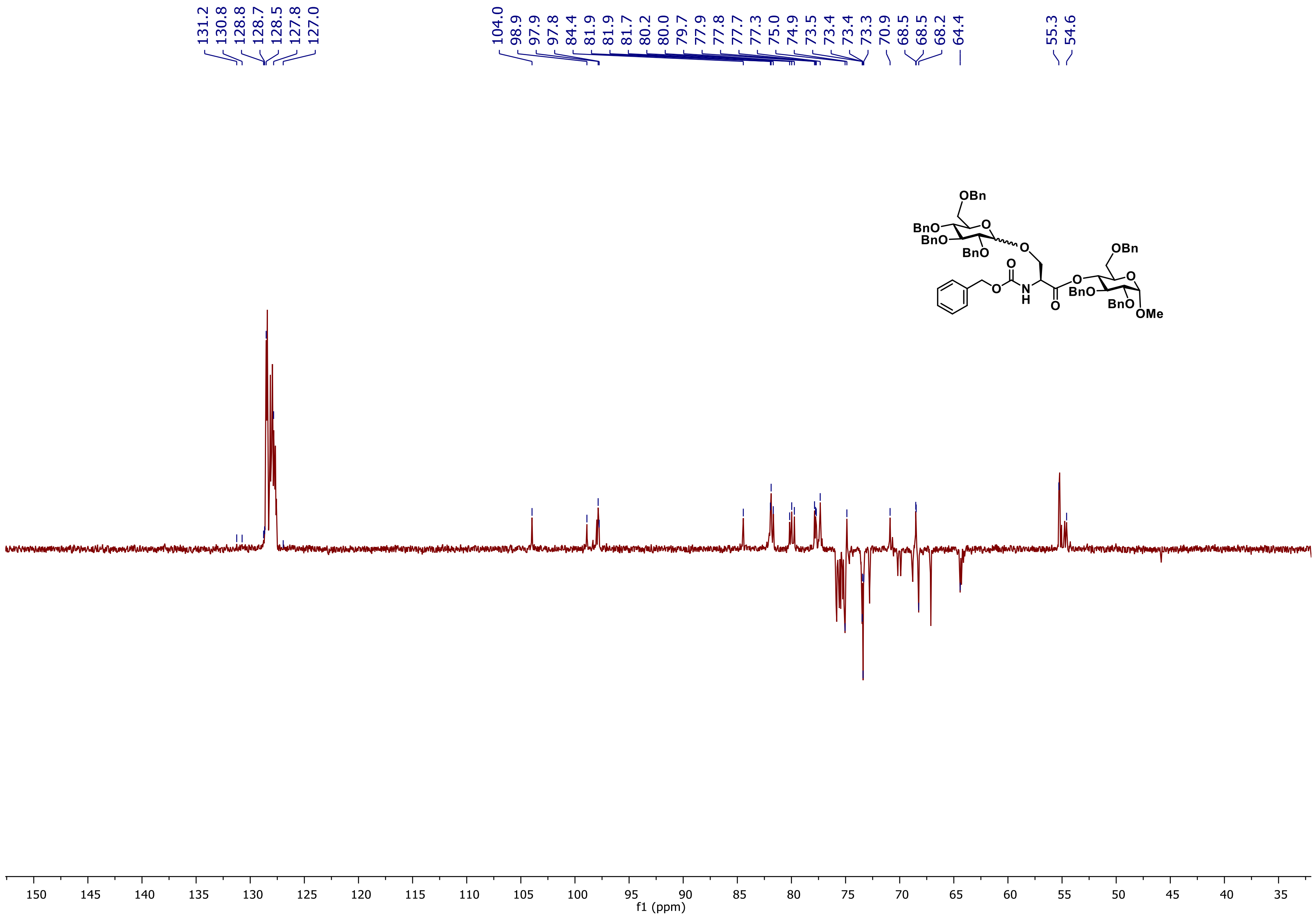

Supplementary Figure S28a. ^{1}H NMR Spectrum (400 MHz, $CDCl_3$) of compound **S26**

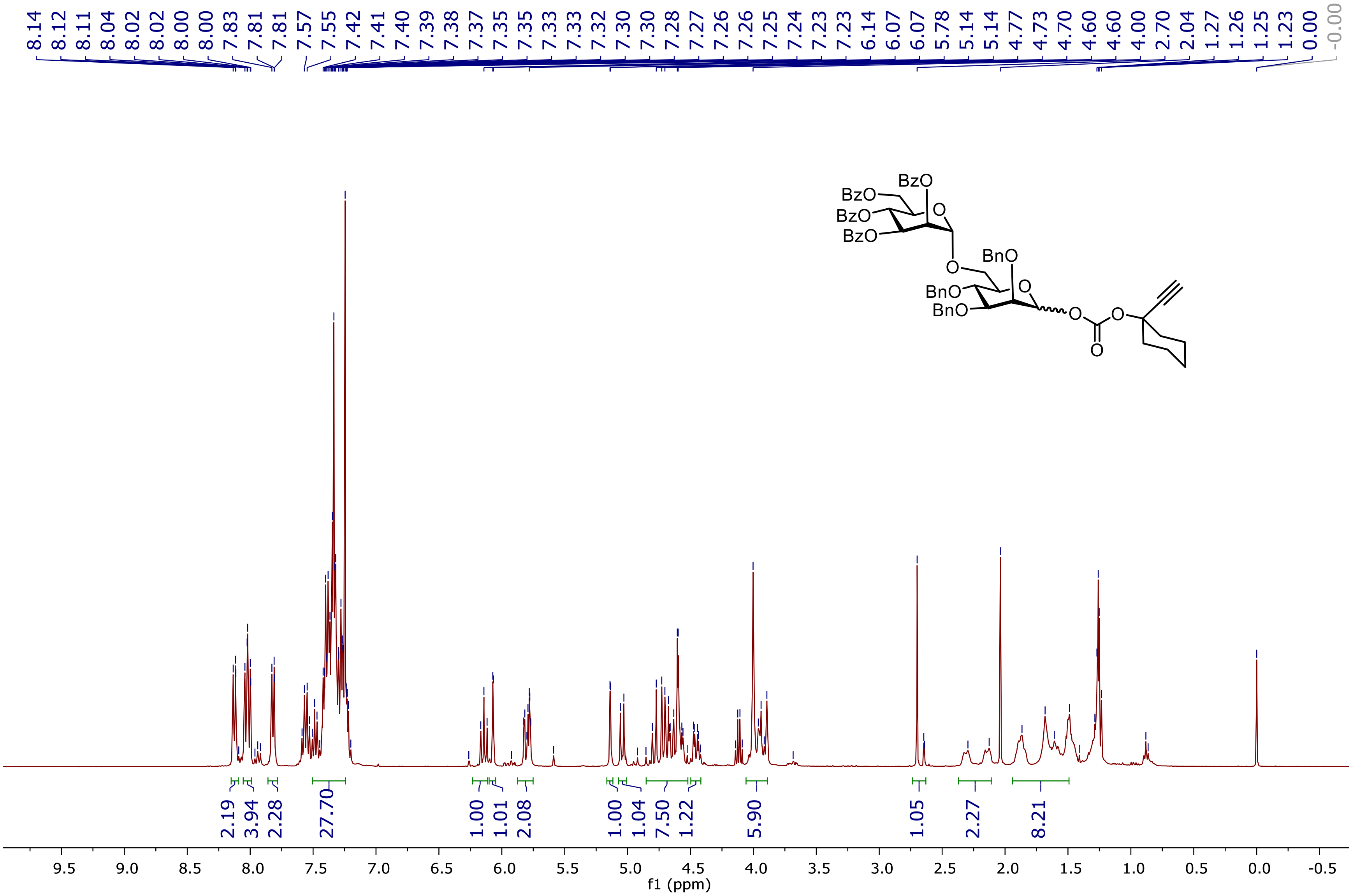

Supplementary Figure S28b. ^{13}C NMR Spectrum (101 MHz, $CDCl_3$) of compound **S26**

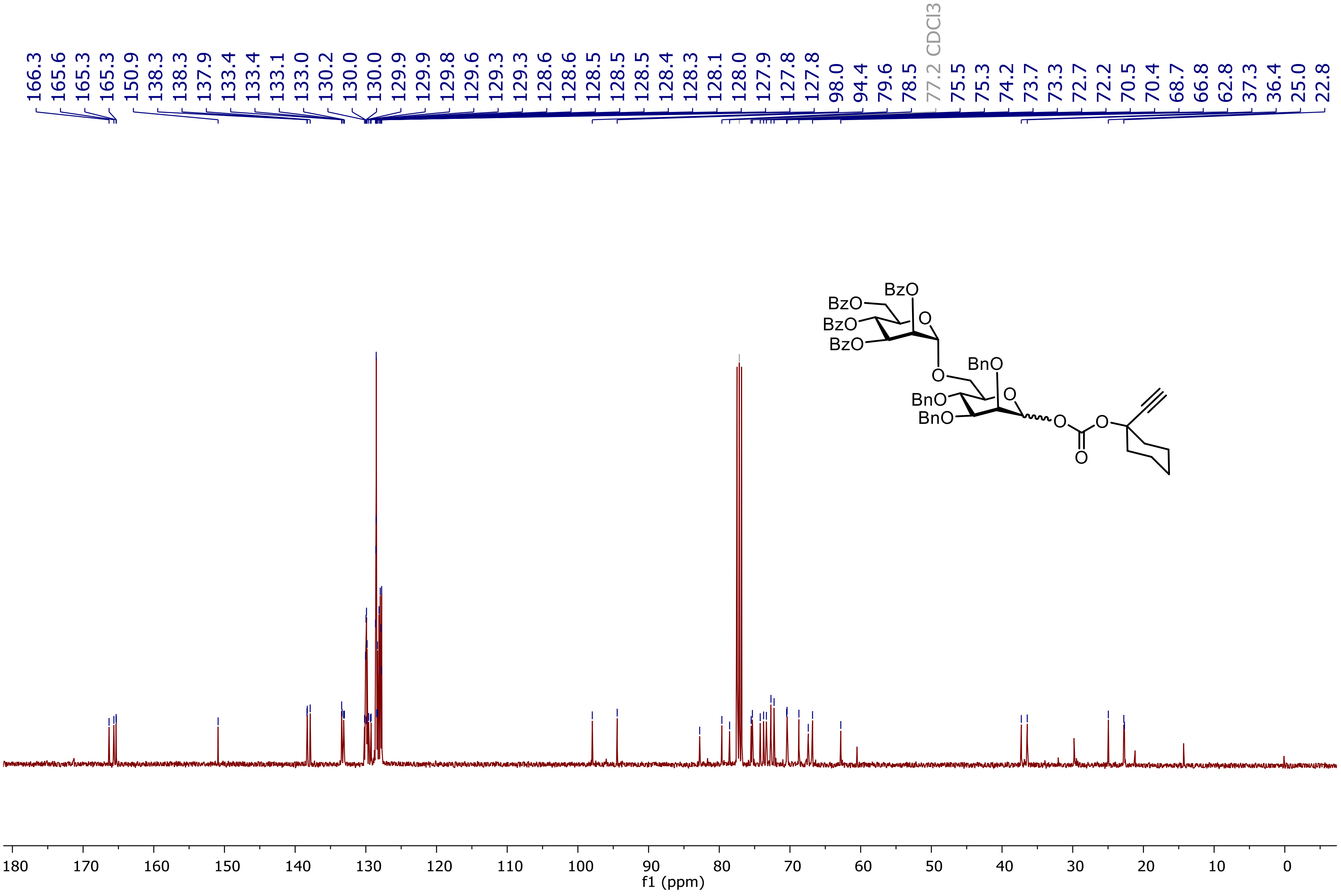

Supplementary Figure S28c. DEPT NMR Spectrum (101 MHz, $CDCl_3$) of compound **S26**

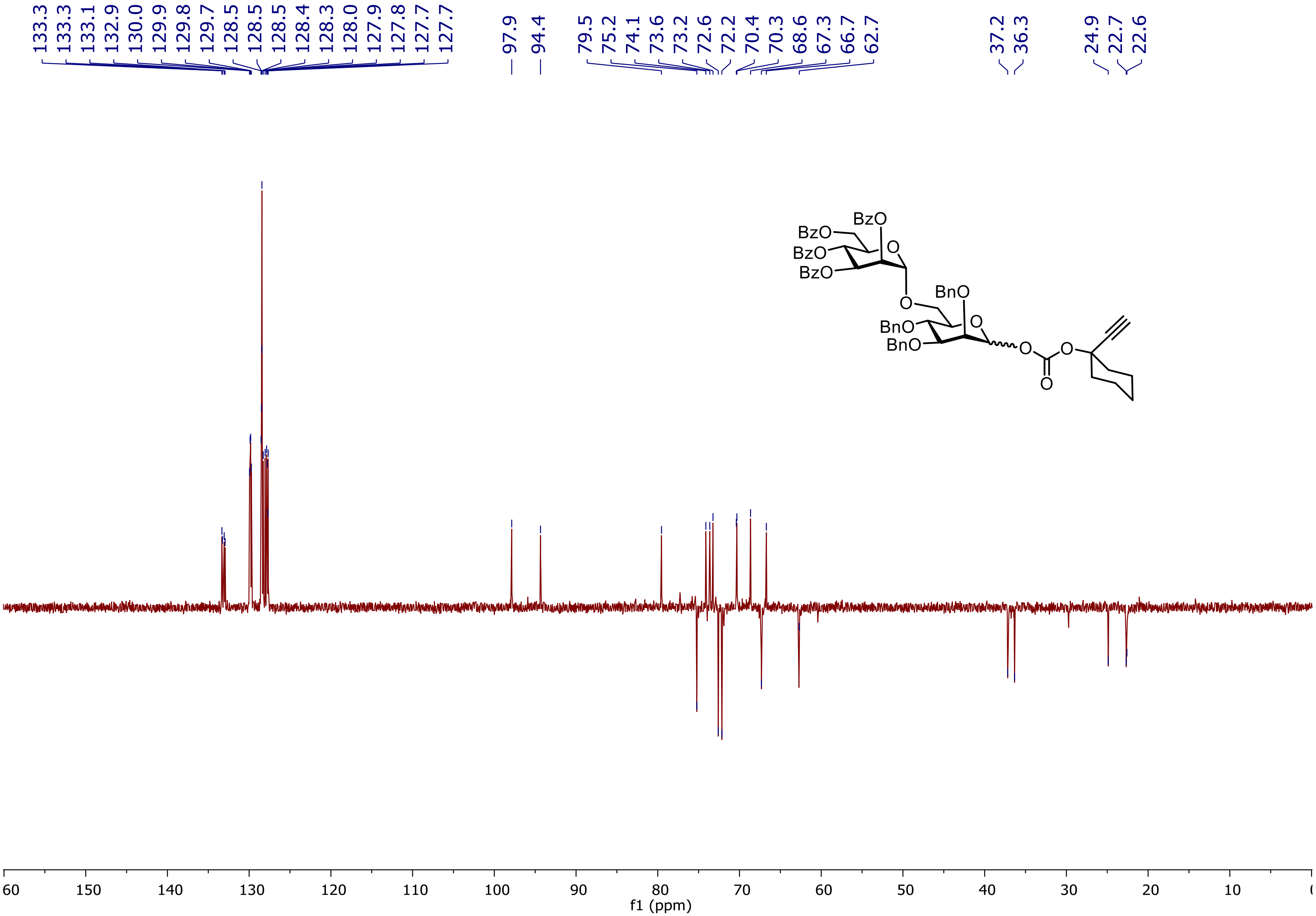

Supplementary Figure S29a. ^{1}H NMR Spectrum (400 MHz, $CDCl_3$) of compound **23**

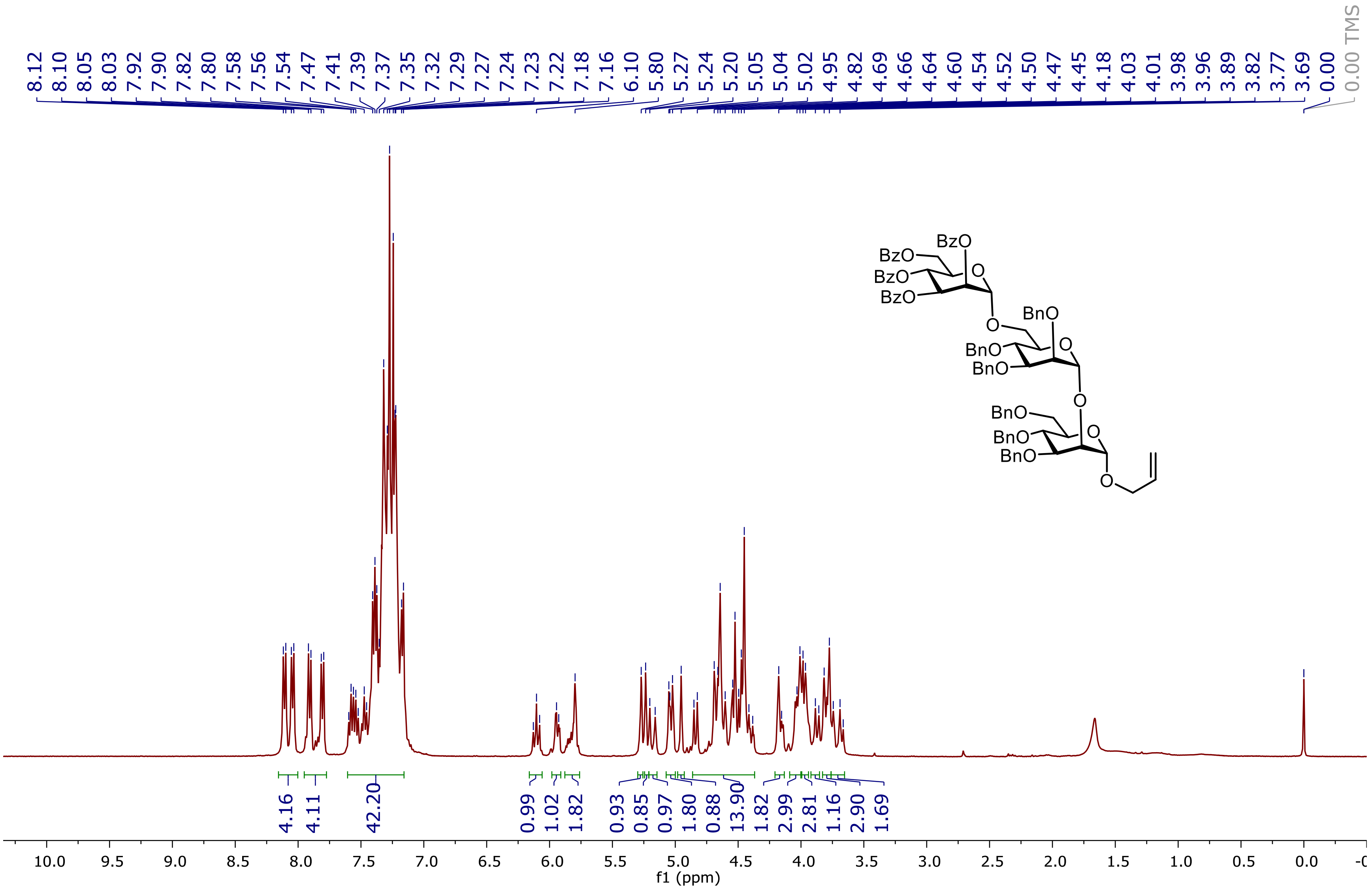

Supplementary Figure S29b. ^{13}C NMR Spectrum (101 MHz, $CDCl_3$) of compound **23**

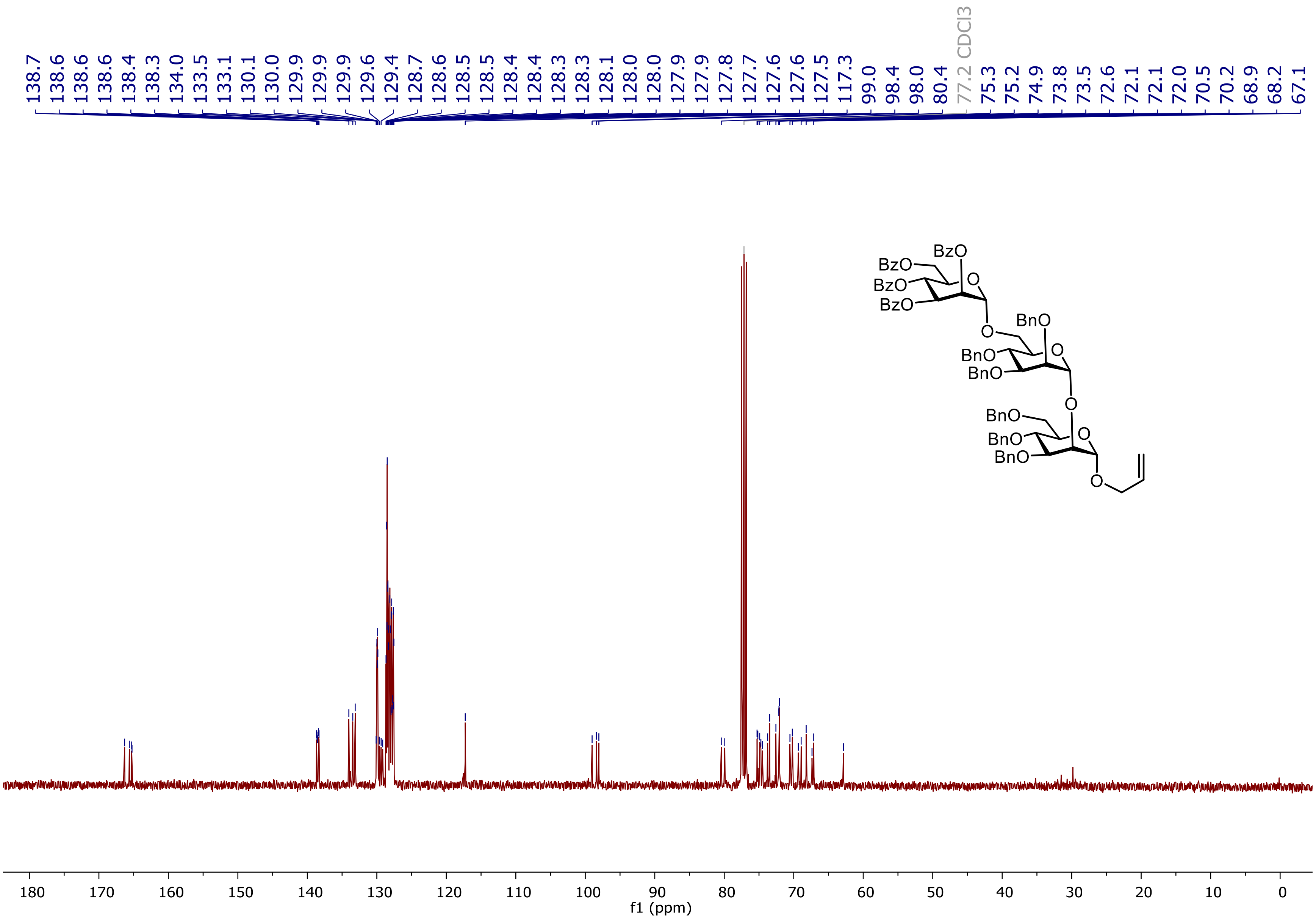

Supplementary Figure S29c. DEPT NMR Spectrum (101 MHz, $CDCl_3$) of compound **23**

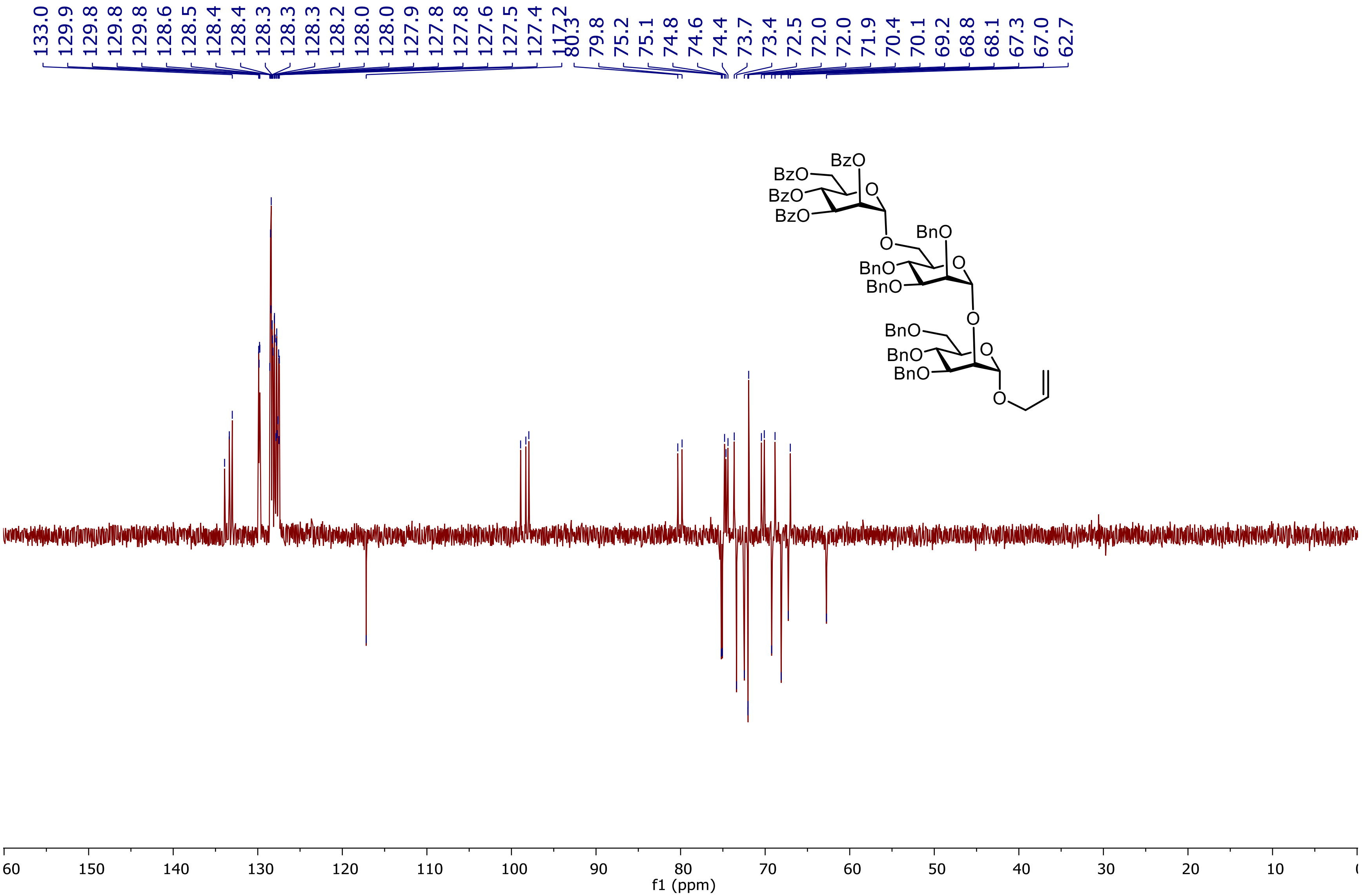

Supplementary Figure S30a. ^{1}H NMR Spectrum (400 MHz, $CDCl_3$) of compound **S30**

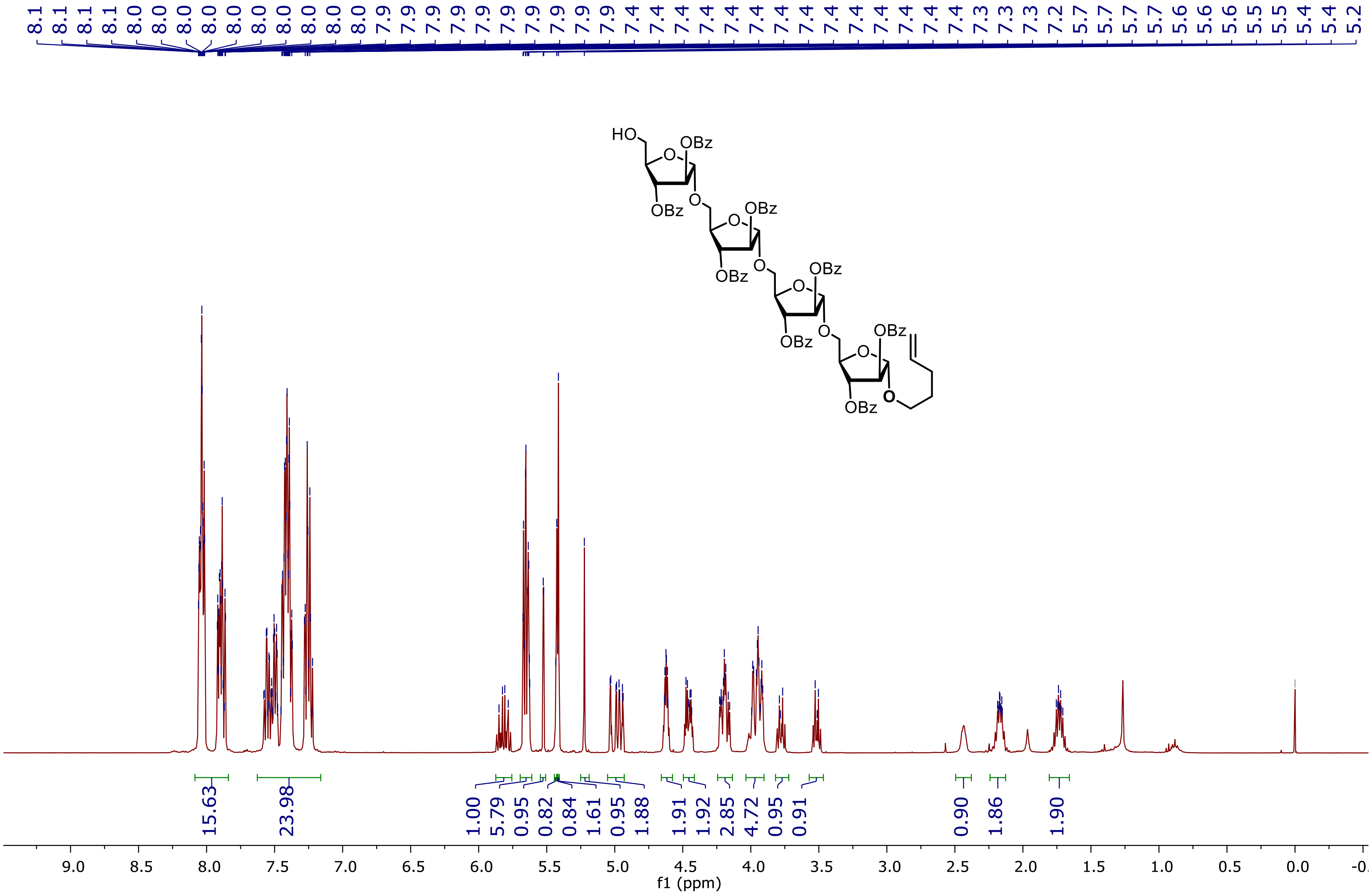

Supplementary Figure S30b. ^{13}C NMR Spectrum (101 MHz, $CDCl_3$) of compound **S30**

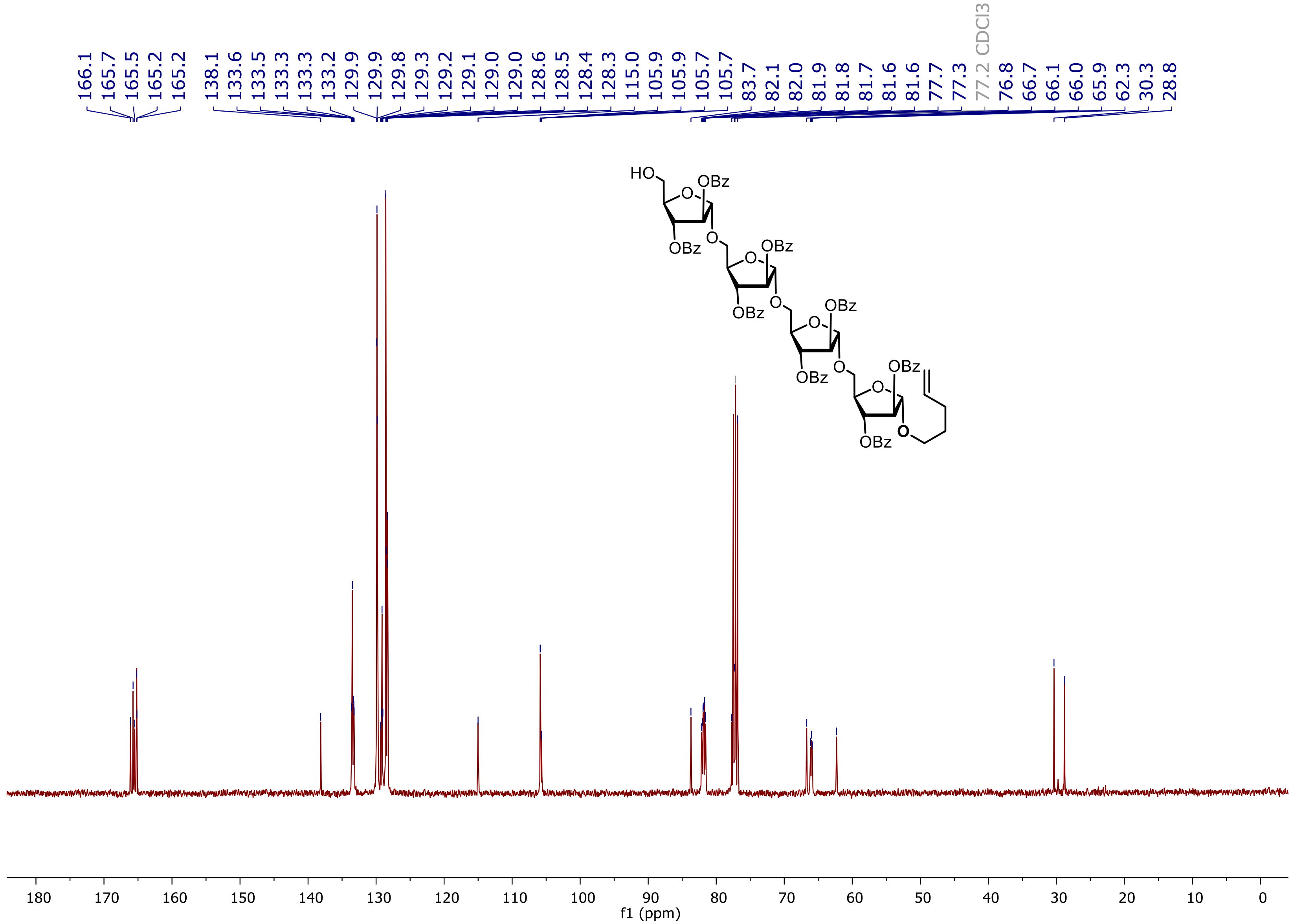

Supplementary Figure S30c. DEPT NMR Spectrum (101 MHz, $CDCl_3$) of compound **S30**

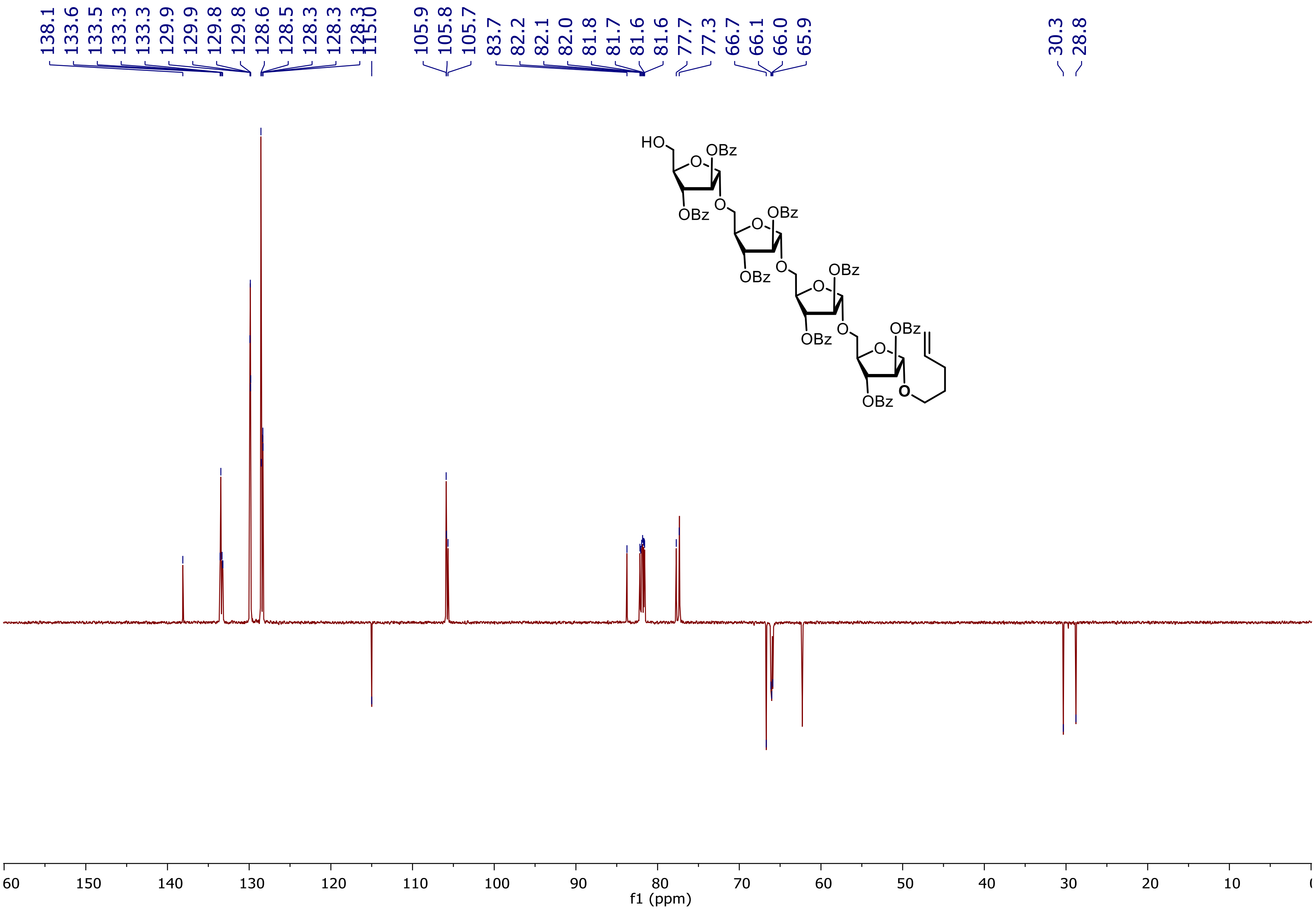

Supplementary Figure S31a. ¹H NMR Spectrum (400 MHz, $CDCl_3$) of compound **S32**

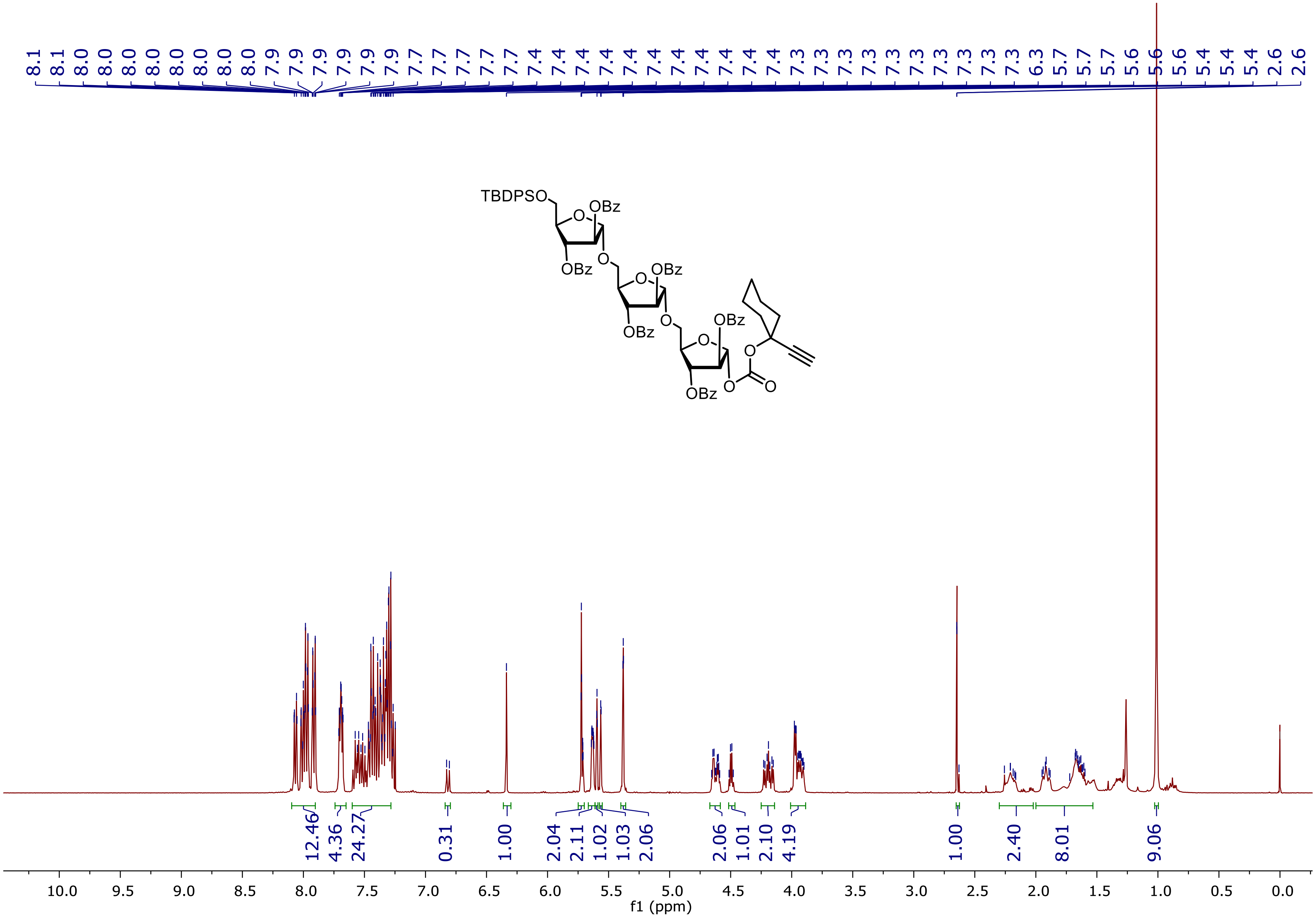

Supplementary Figure S31b. ^{13}C NMR Spectrum (101 MHz, $CDCl_3$) of compound **S32**

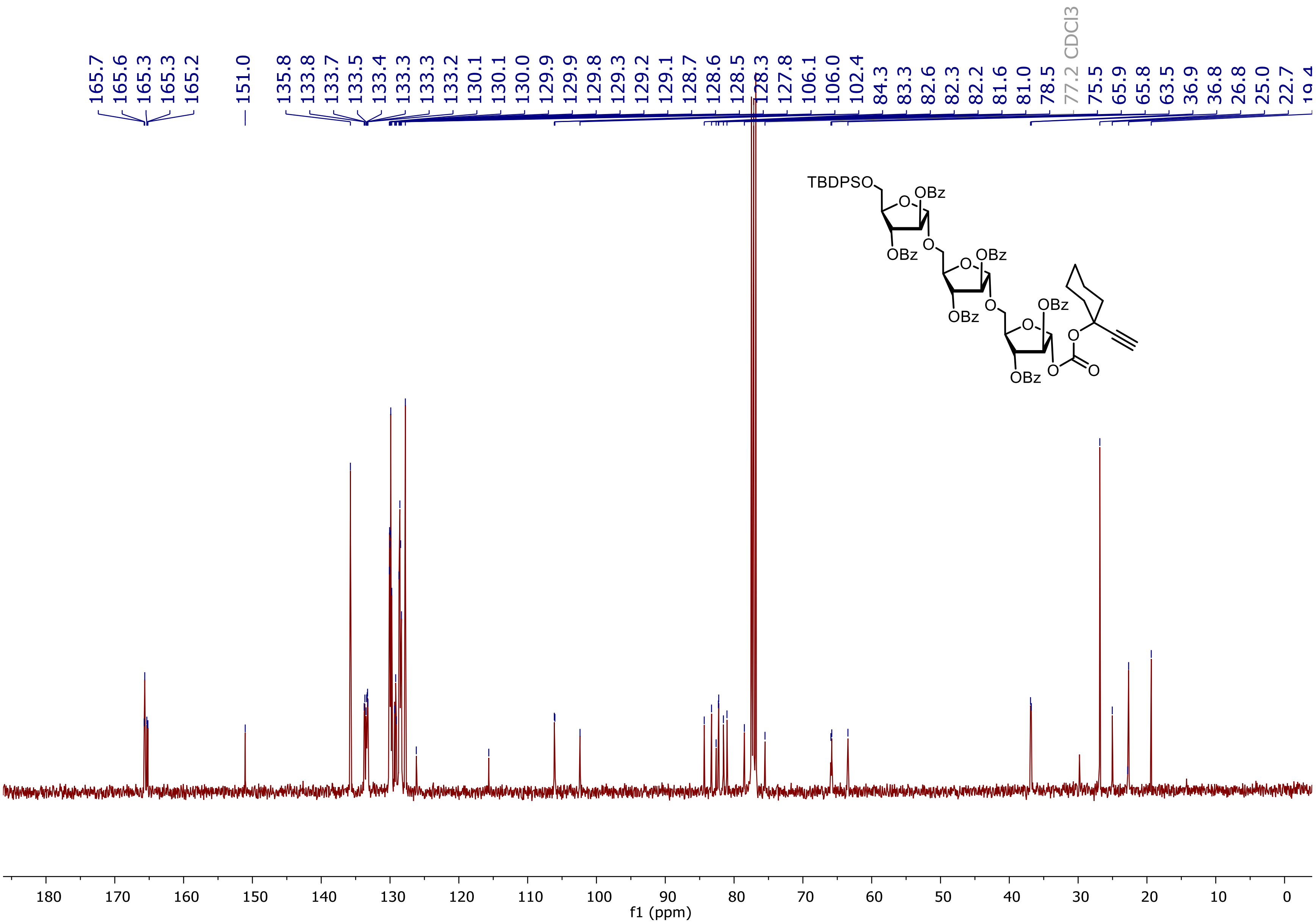

Supplementary Figure S32a. ^{1}H NMR Spectrum (400 MHz, $CDCl_3$) of compound **S34**

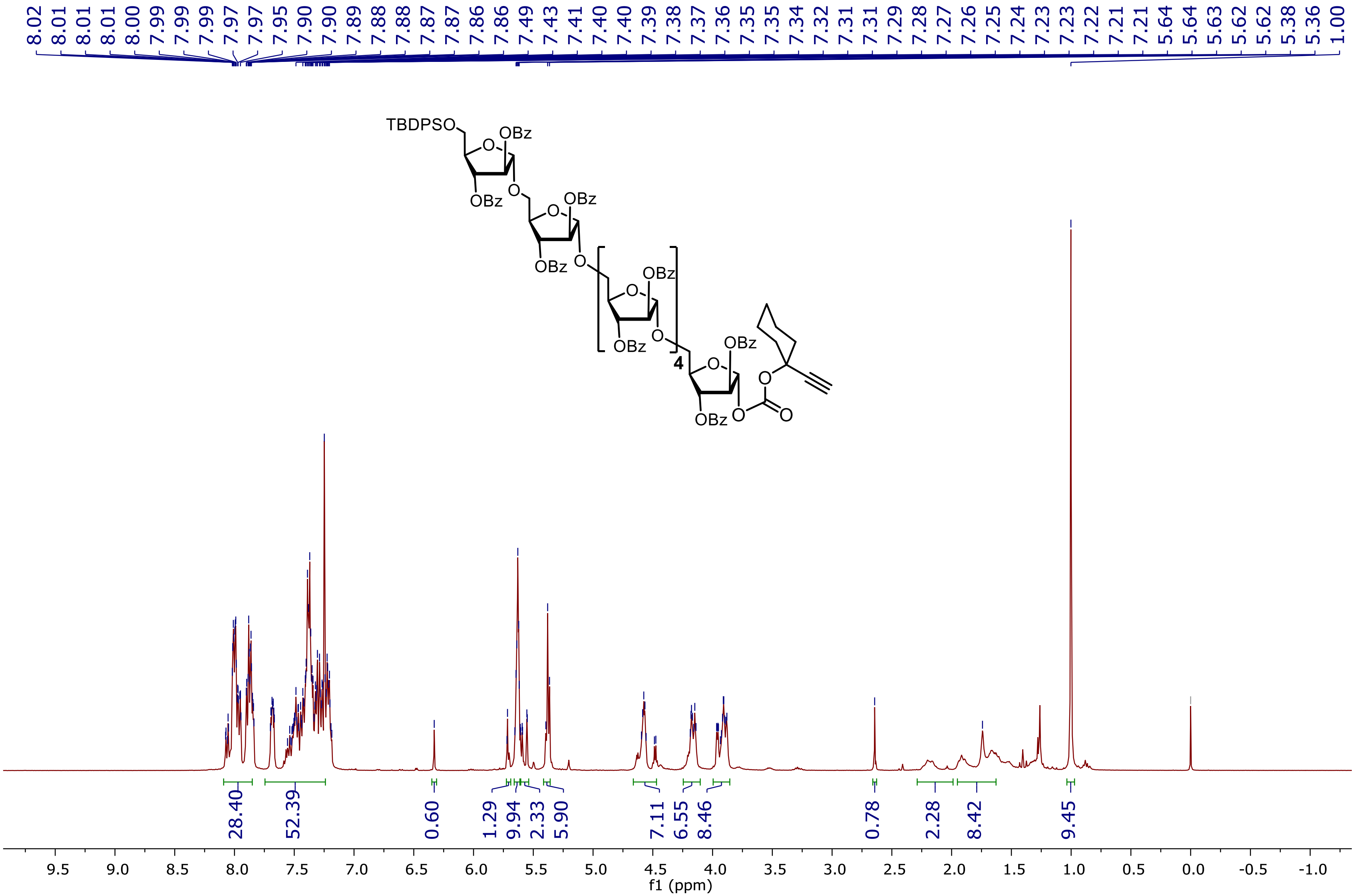

Supplementary Figure S32b. ^{13}C NMR Spectrum (101 MHz, $CDCl_3$) of compound **S34**

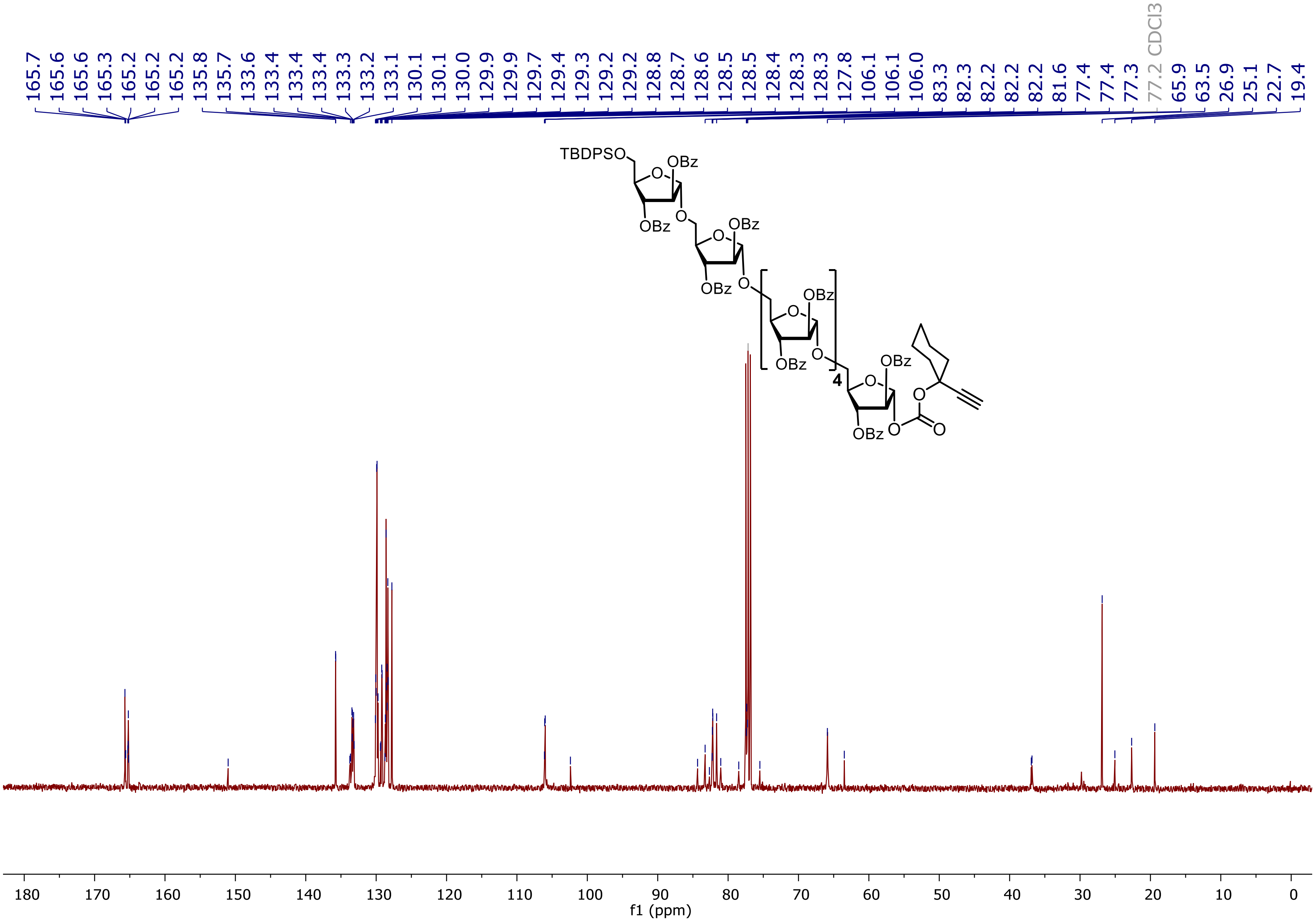

Supplementary Figure S32c. DEPT NMR Spectrum (101 MHz, $CDCl_3$) of compound **S34**

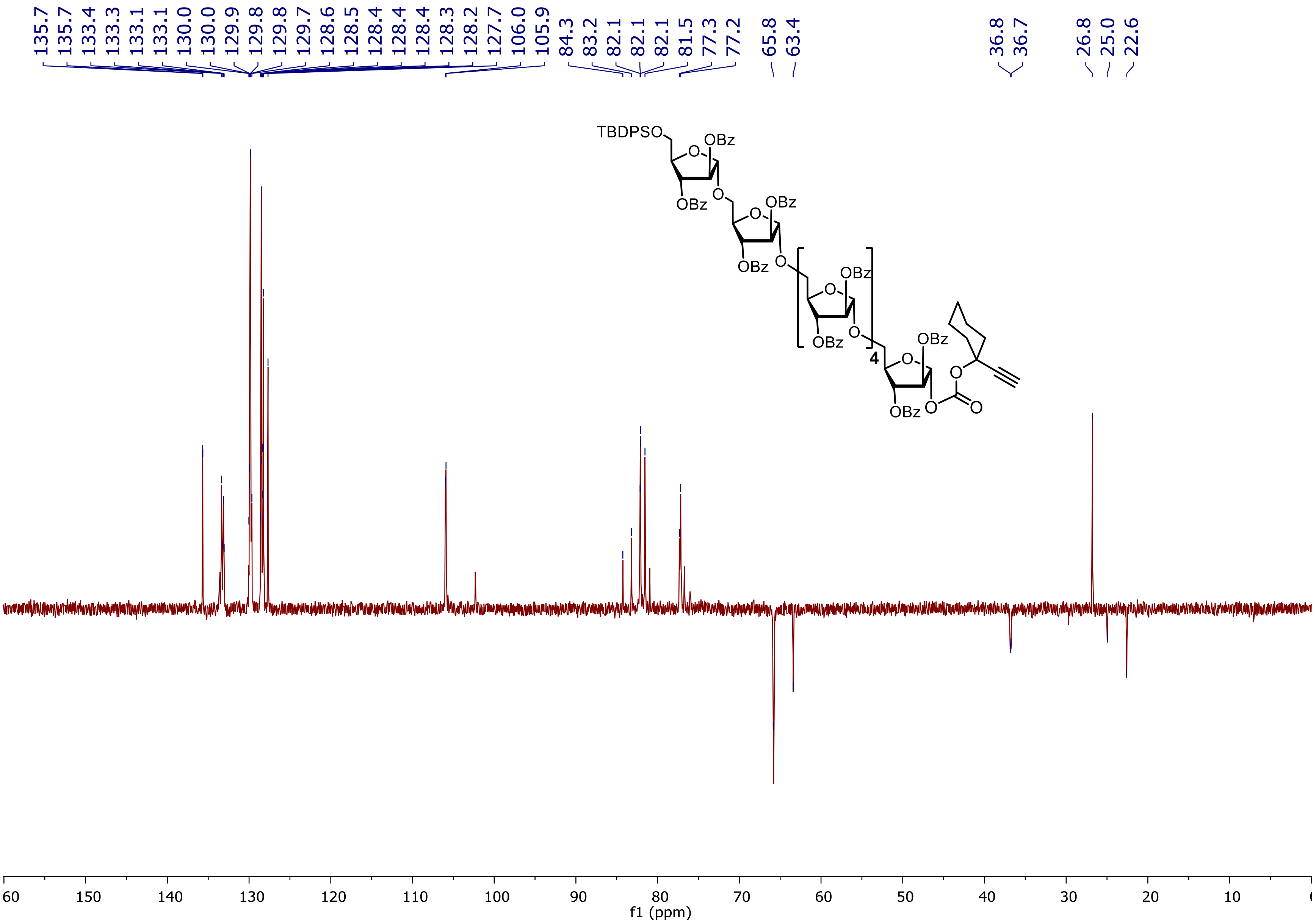

Supplementary Figure S33a. ^{1}H NMR Spectrum (600 MHz, $CDCl_3$) of compound **24**

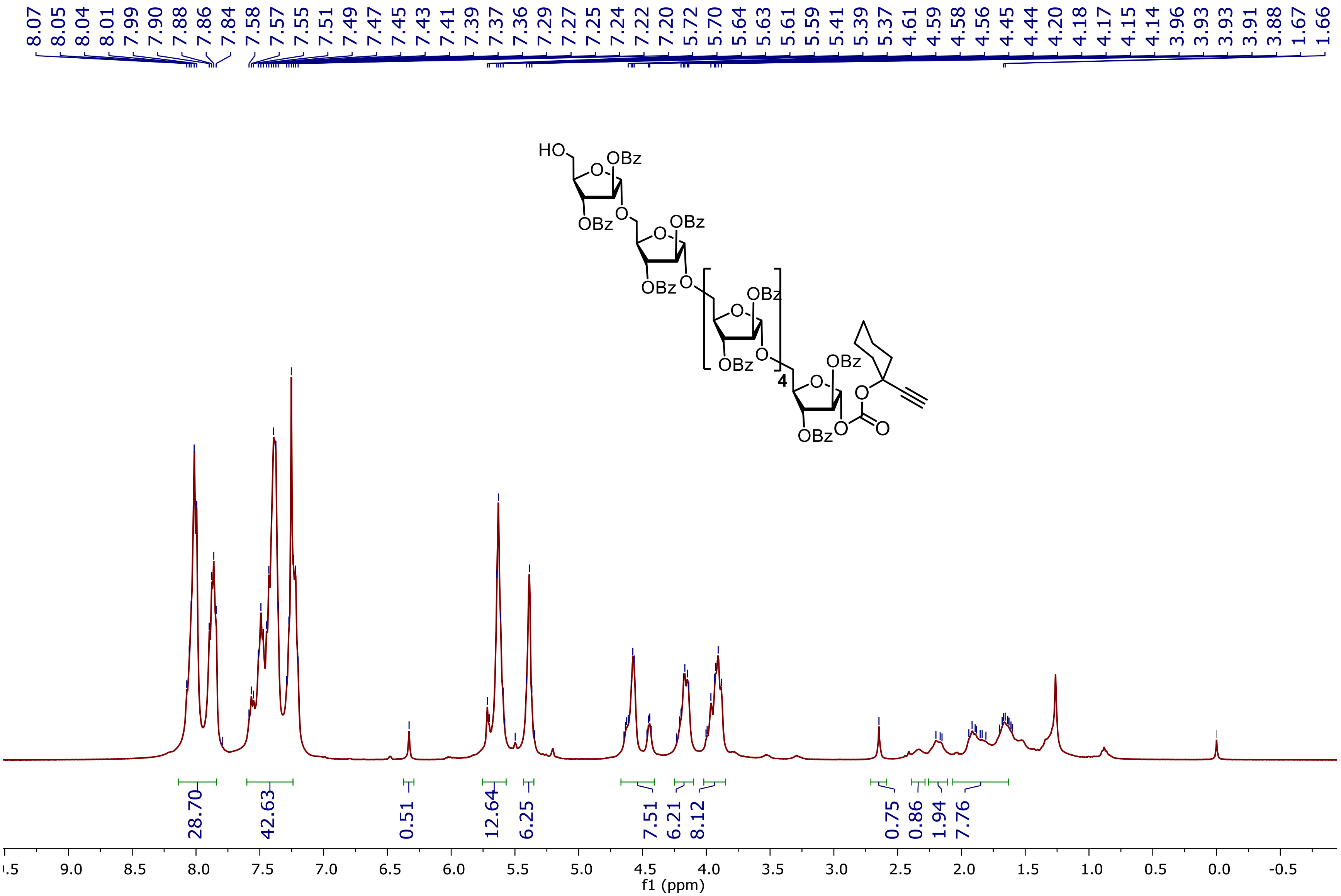

Supplementary Figure S33b. ^{13}C NMR Spectrum (151 MHz, $CDCl_3$) of compound **24**

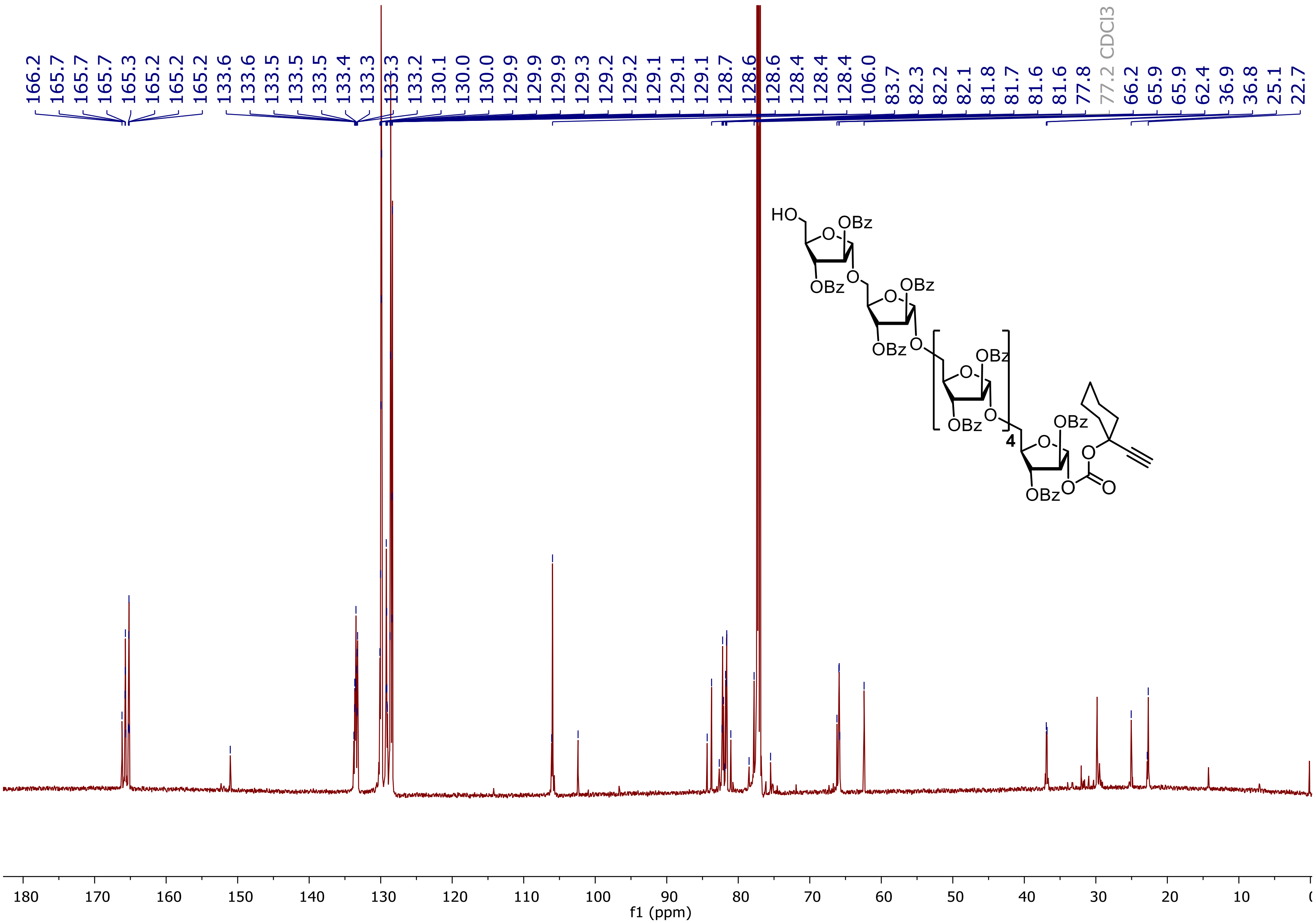

Supplementary Figure S33c. DEPT NMR Spectrum (151 MHz, $CDCl_3$) of compound **24**

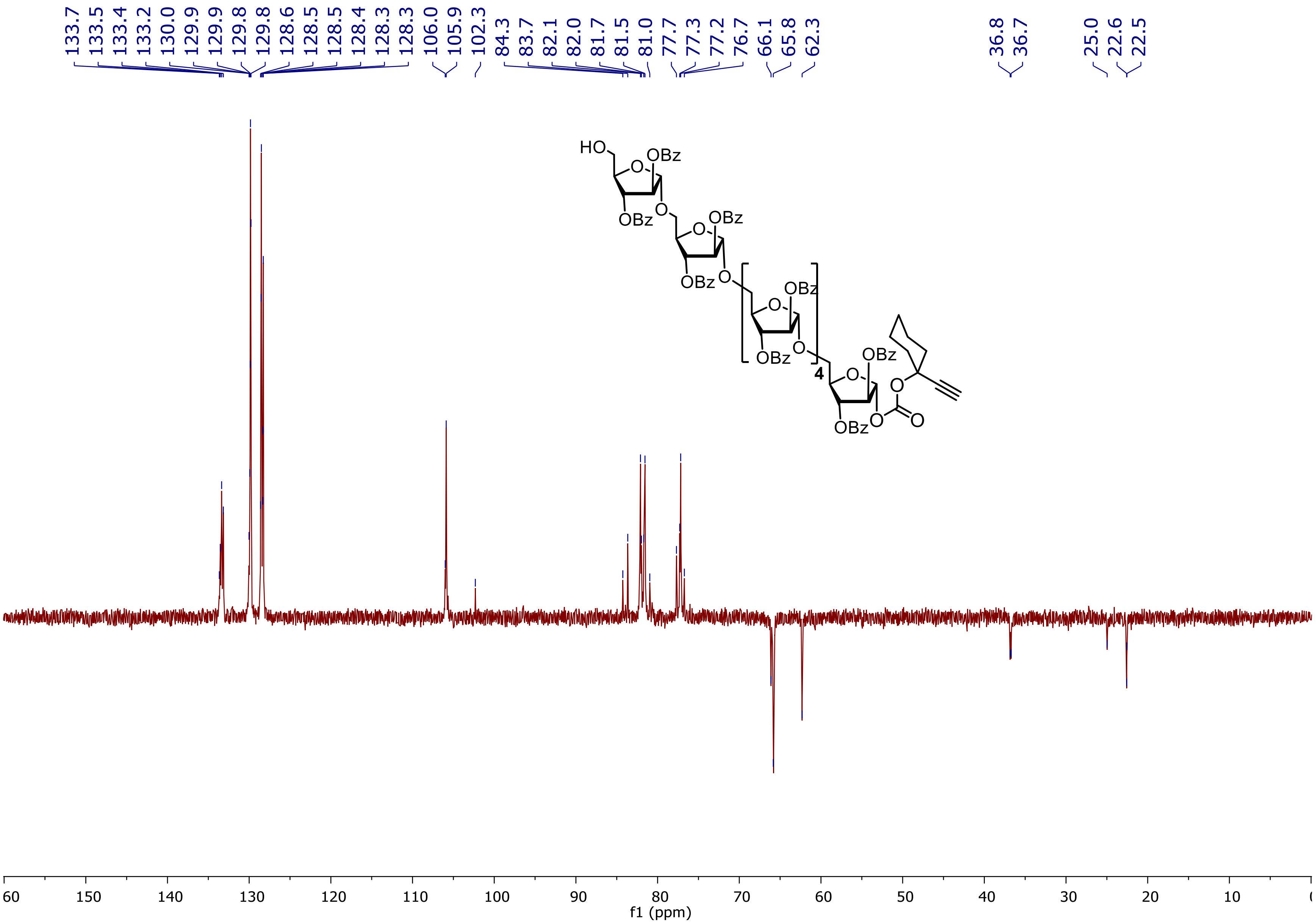

Supplementary Figure S34a. ^{1}H NMR Spectrum (600 MHz, $CDCl_3$) of compound **26**

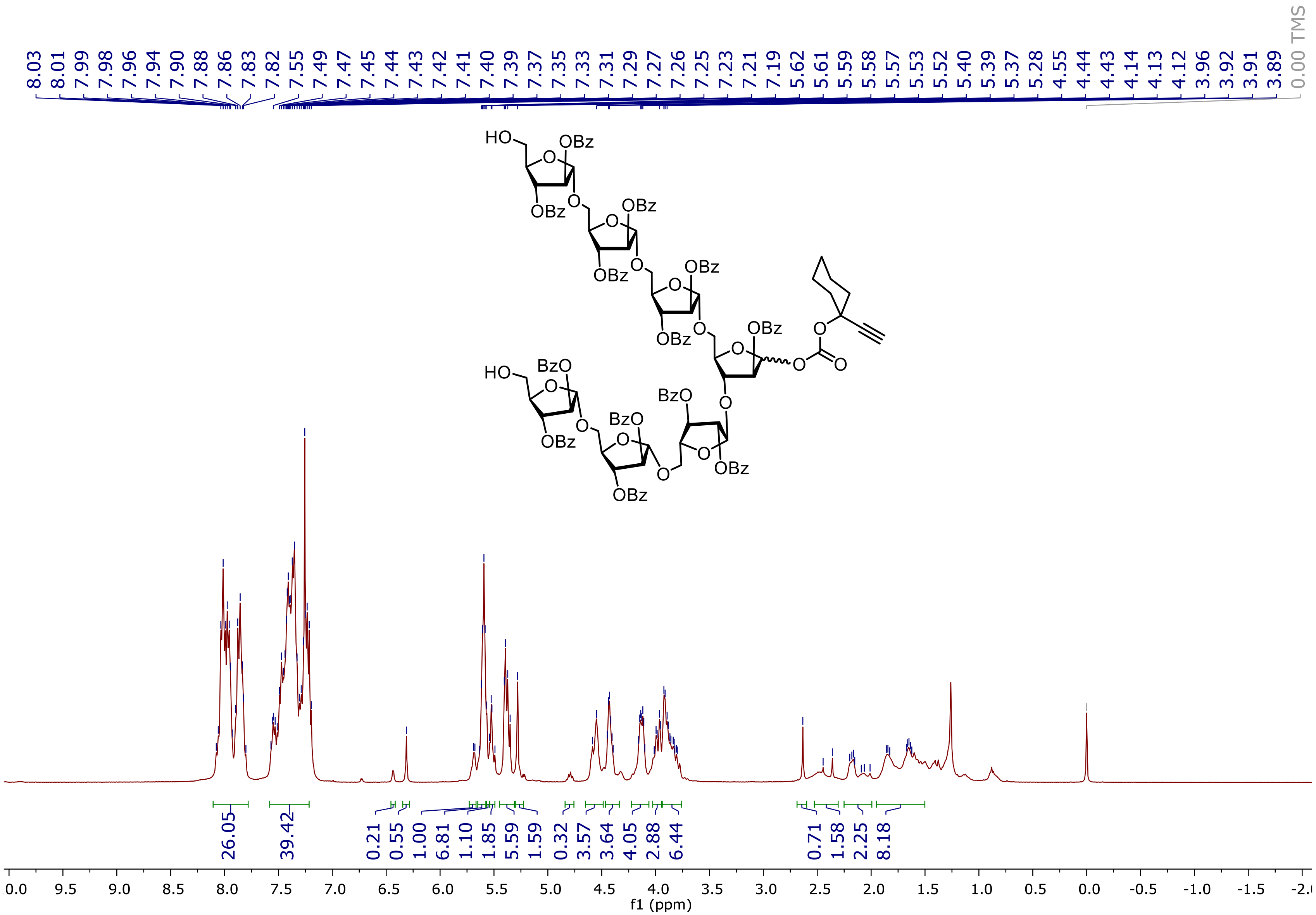

Supplementary Figure S34b. ^{13}C NMR Spectrum (151 MHz, $CDCl_3$) of compound **26**

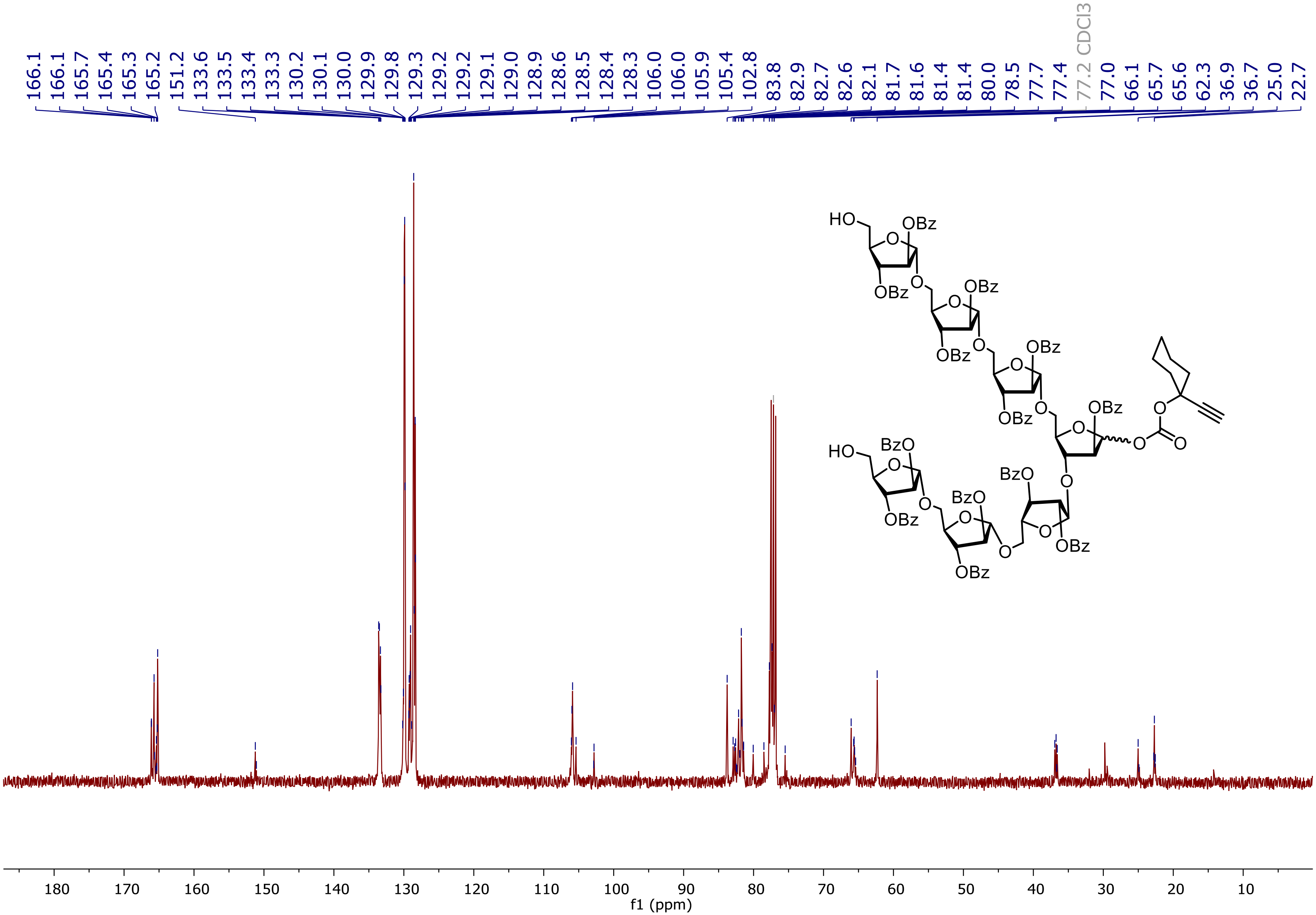

Supplementary Figure S34c. DEPT NMR Spectrum (151 MHz, $CDCl_3$) of compound **26**

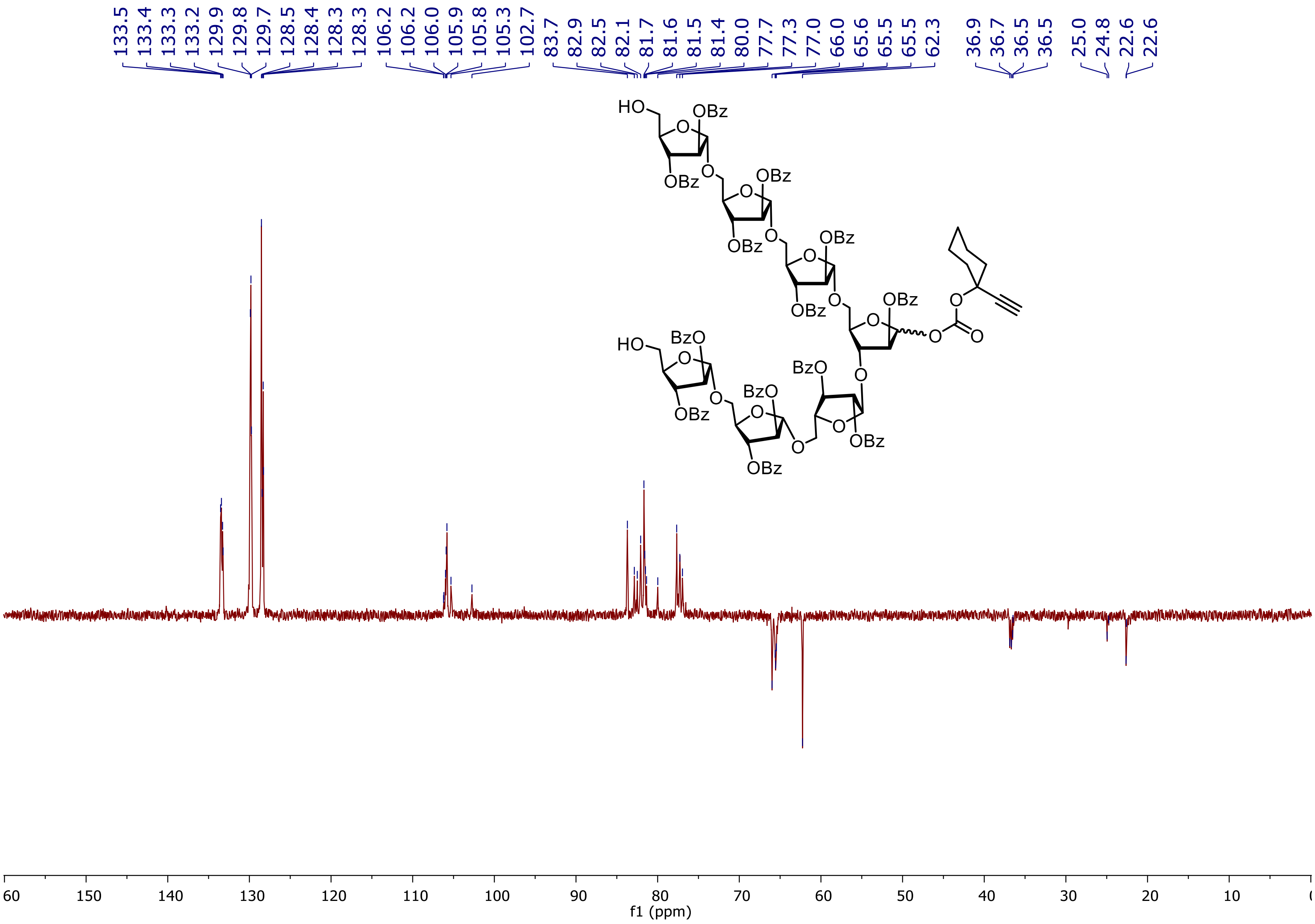

Supplementary Figure S35a. ^{1}H NMR Spectrum (400 MHz, $CDCl_3$) of compound **10**

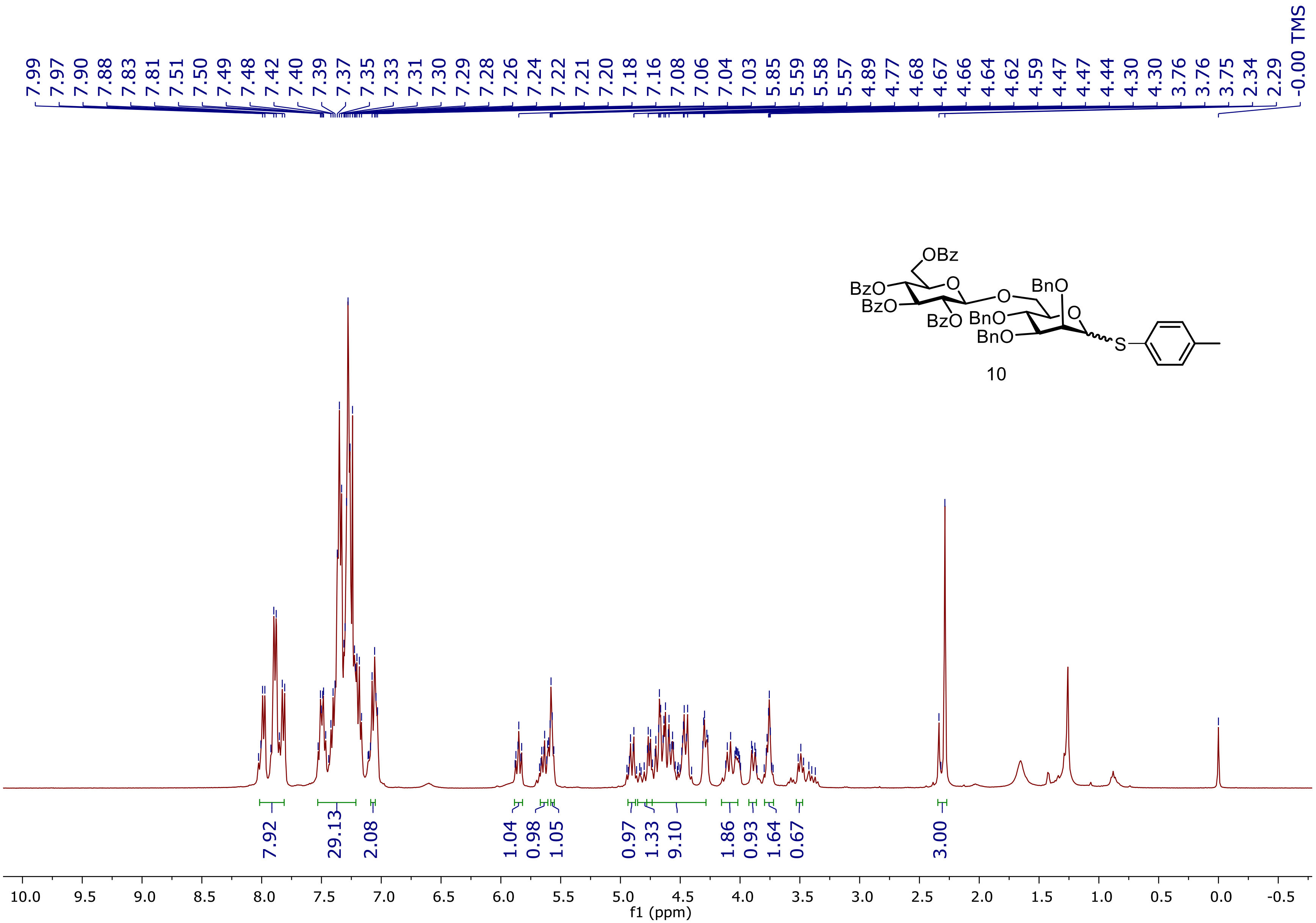

10

Supplementary Figure S35b. ^{13}C NMR Spectrum (101 MHz, $CDCl_3$) of compound **10**

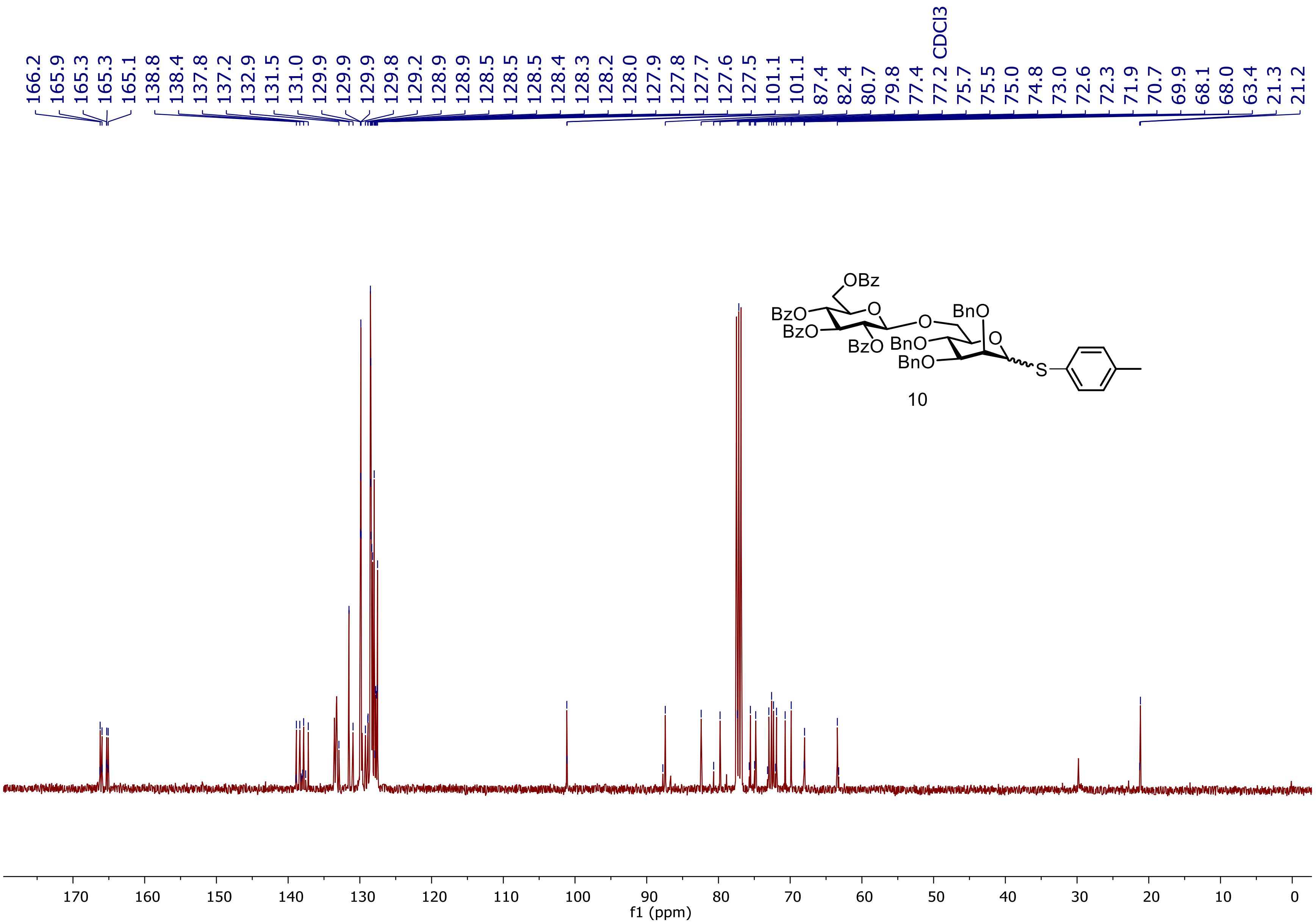

Supplementary Figure S35c. DEPT NMR Spectrum (101 MHz, $CDCl_3$) of compound **10**

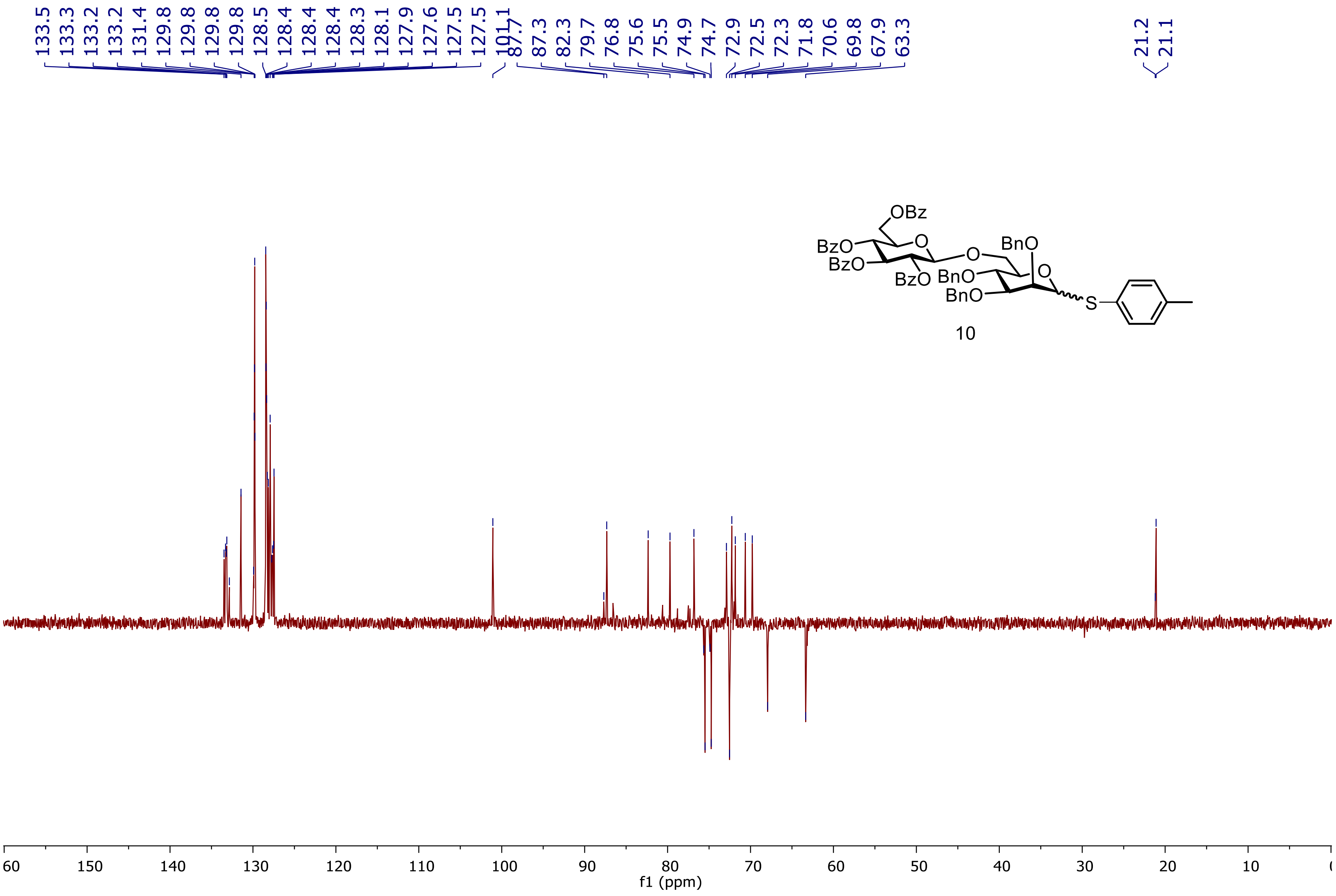

Supplementary Figure S36a. ^{1}H NMR Spectrum (400 MHz, $CDCl_3$) of compound **11c**

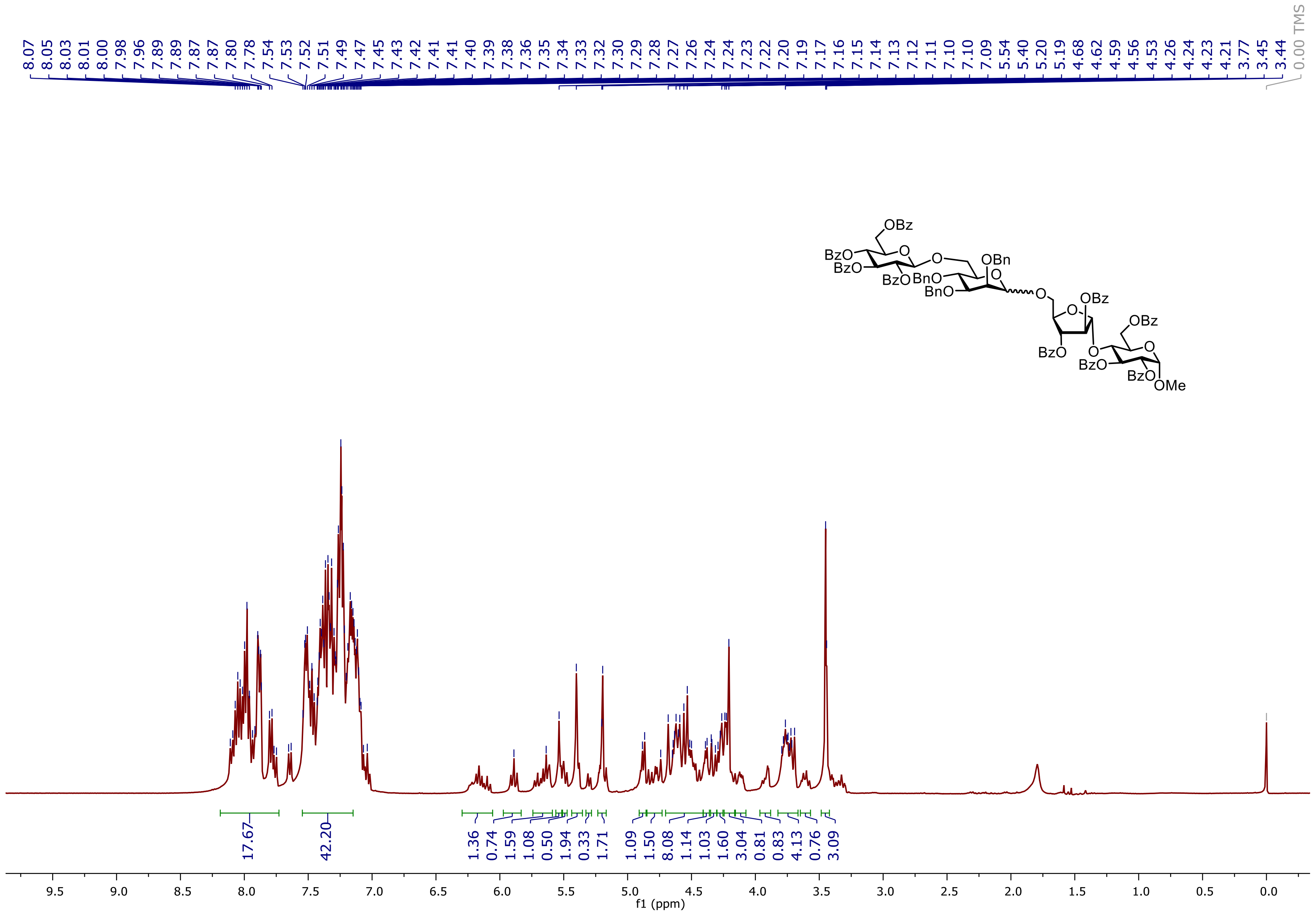

Supplementary Figure S36b. ^{13}C NMR Spectrum (101MHz, $CDCl_3$) of compound **11c**

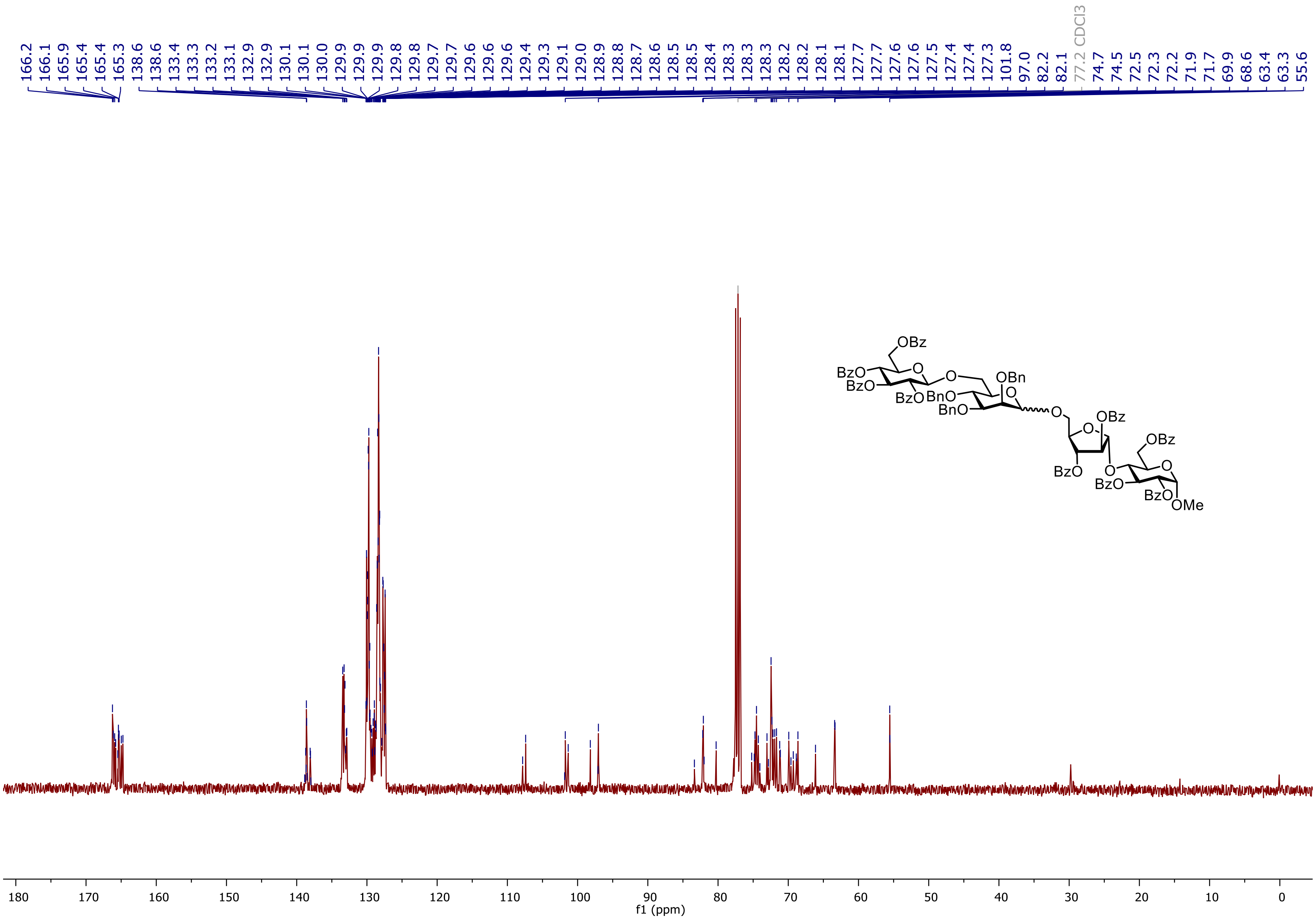

Supplementary Figure S36c. DEPT NMR Spectrum (101 MHz, $CDCl_3$) of compound **11c**

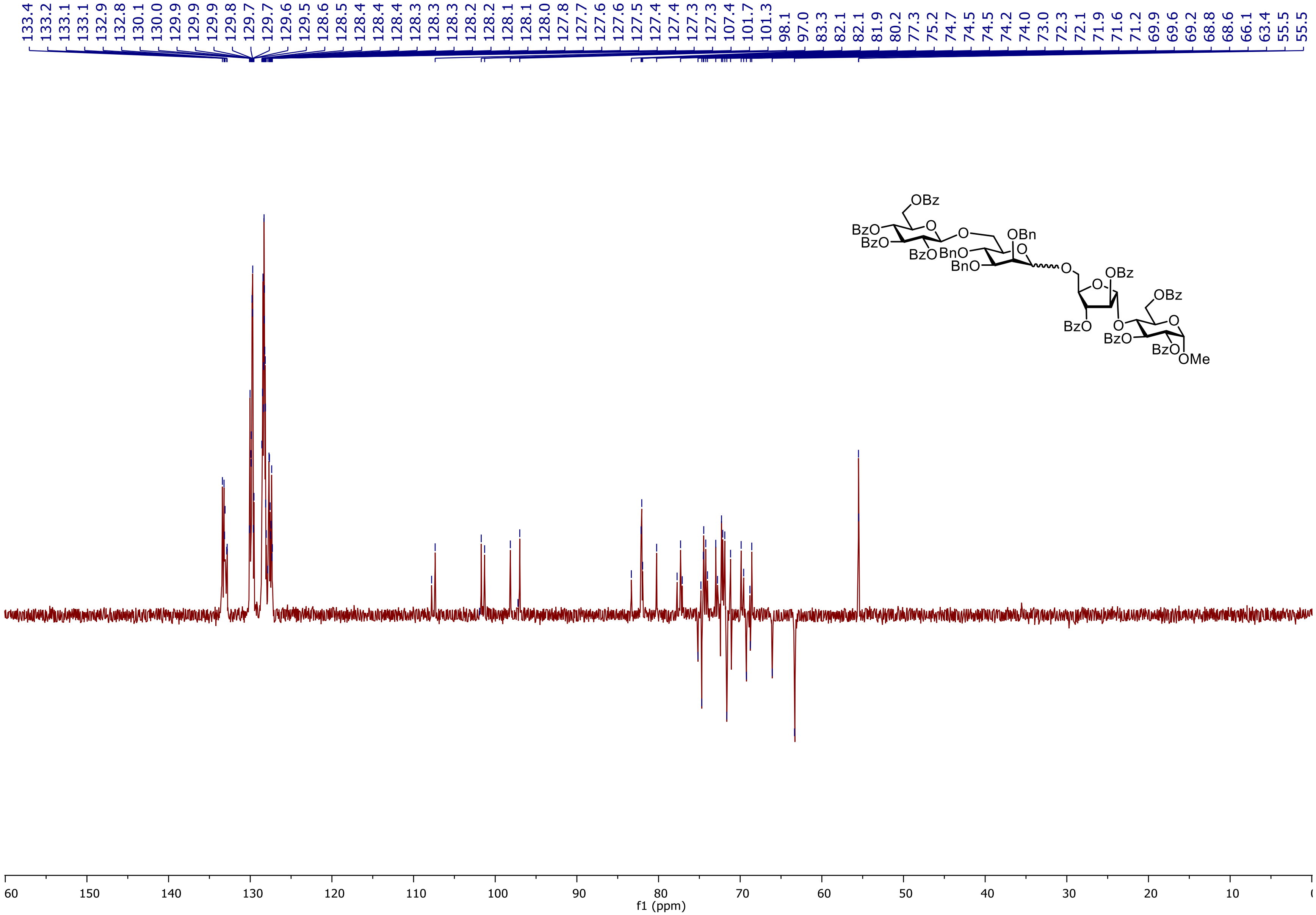

Supplementary Figure S37a. ^{1}H NMR Spectrum (400 MHz, $CDCl_3$) of compound **11b**

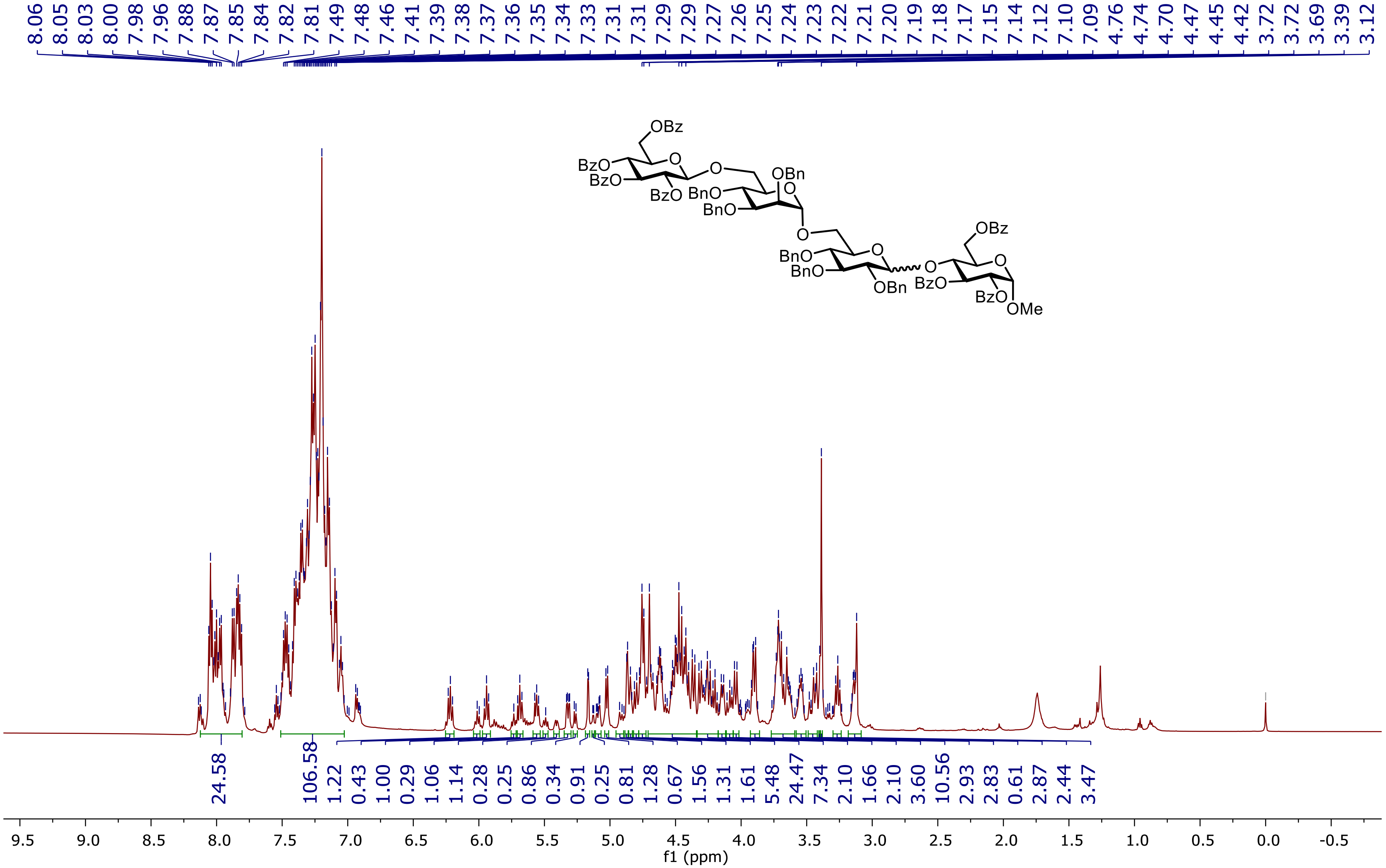

Supplementary Figure S37b. ^{13}C NMR Spectrum (101 MHz, $CDCl_3$) of compound **11b**

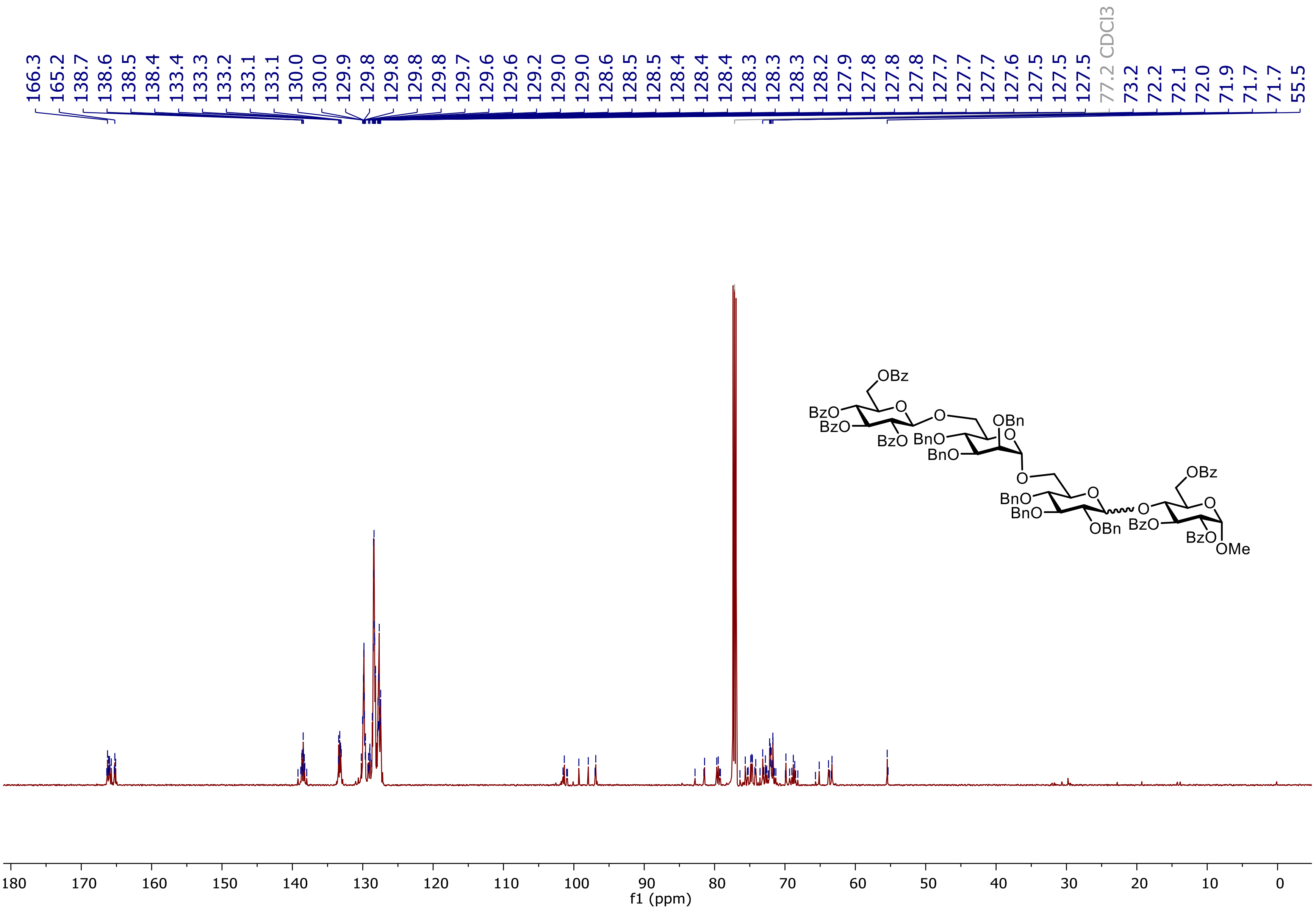

Supplementary Figure S37c. DEPT NMR Spectrum (101 MHz, $CDCl_3$) of compound **11b**

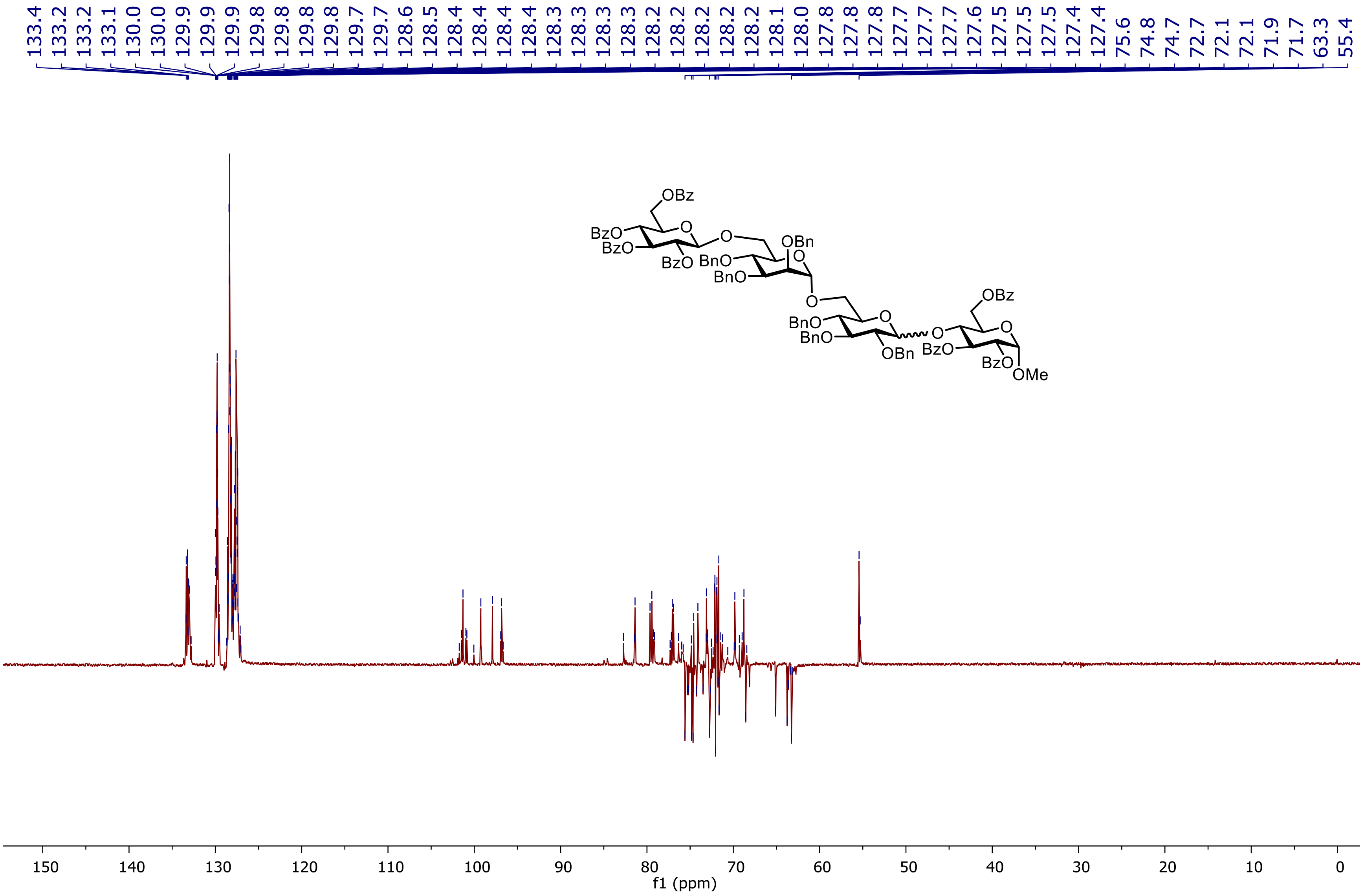

Supplementary Figure S38a. ^{1}H NMR Spectrum (400 MHz, $CDCl_3$) of compound **11a**

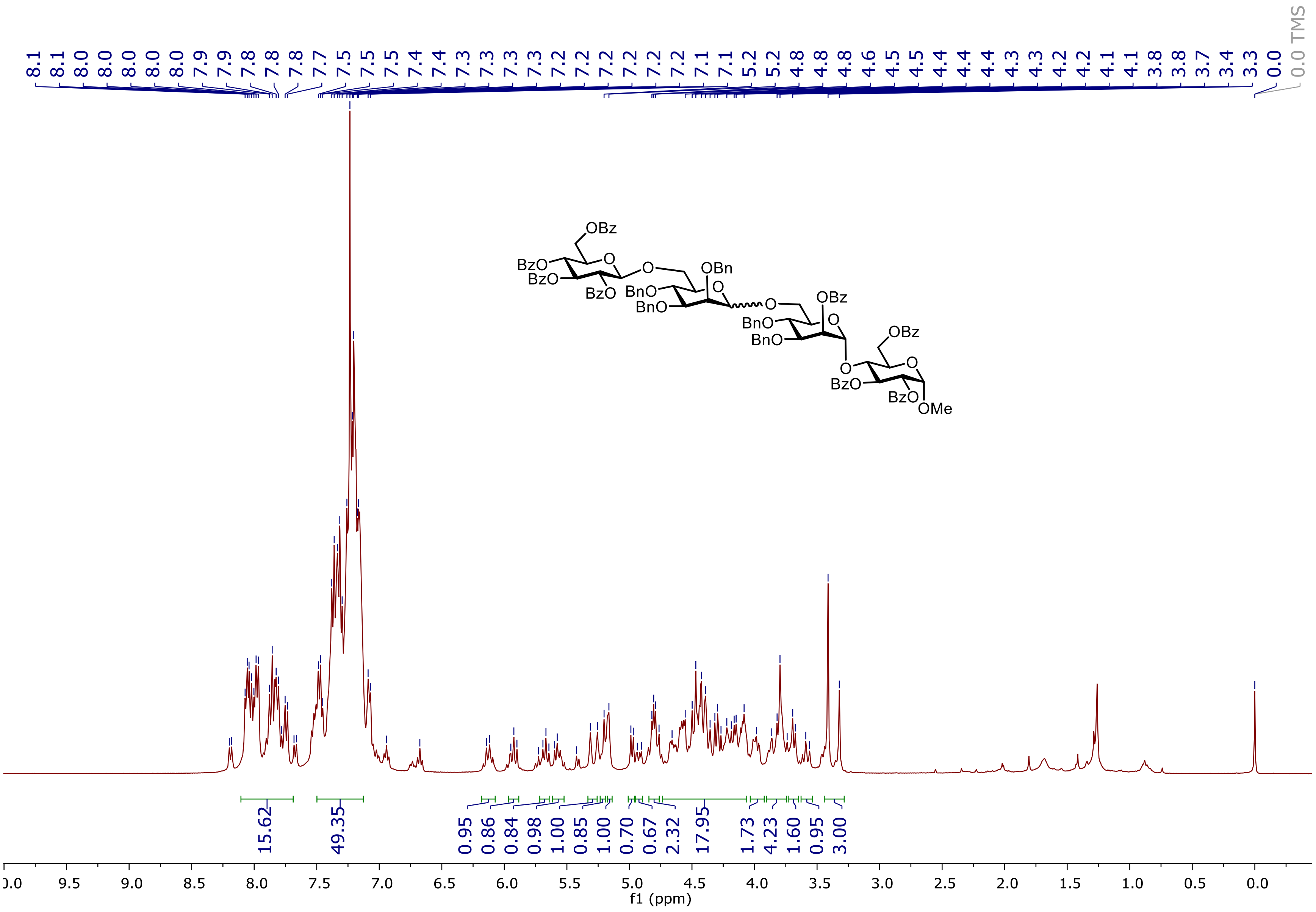

Supplementary Figure S38b. ^{13}C NMR Spectrum (101 MHz, $CDCl_3$) of compound **11a**

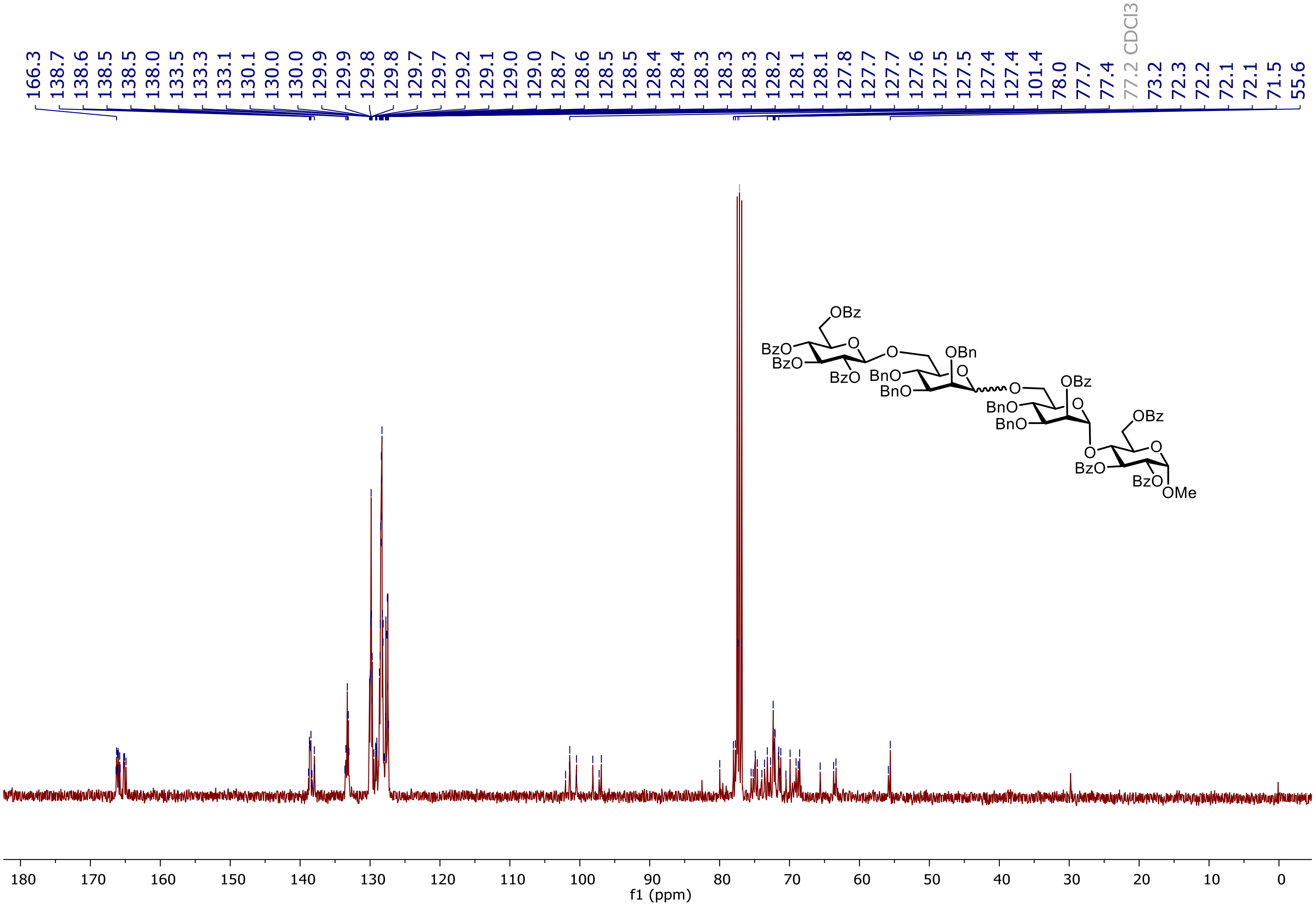

Supplementary Figure S38c. DEPT NMR Spectrum (101 MHz, $CDCl_3$) of compound **11a**

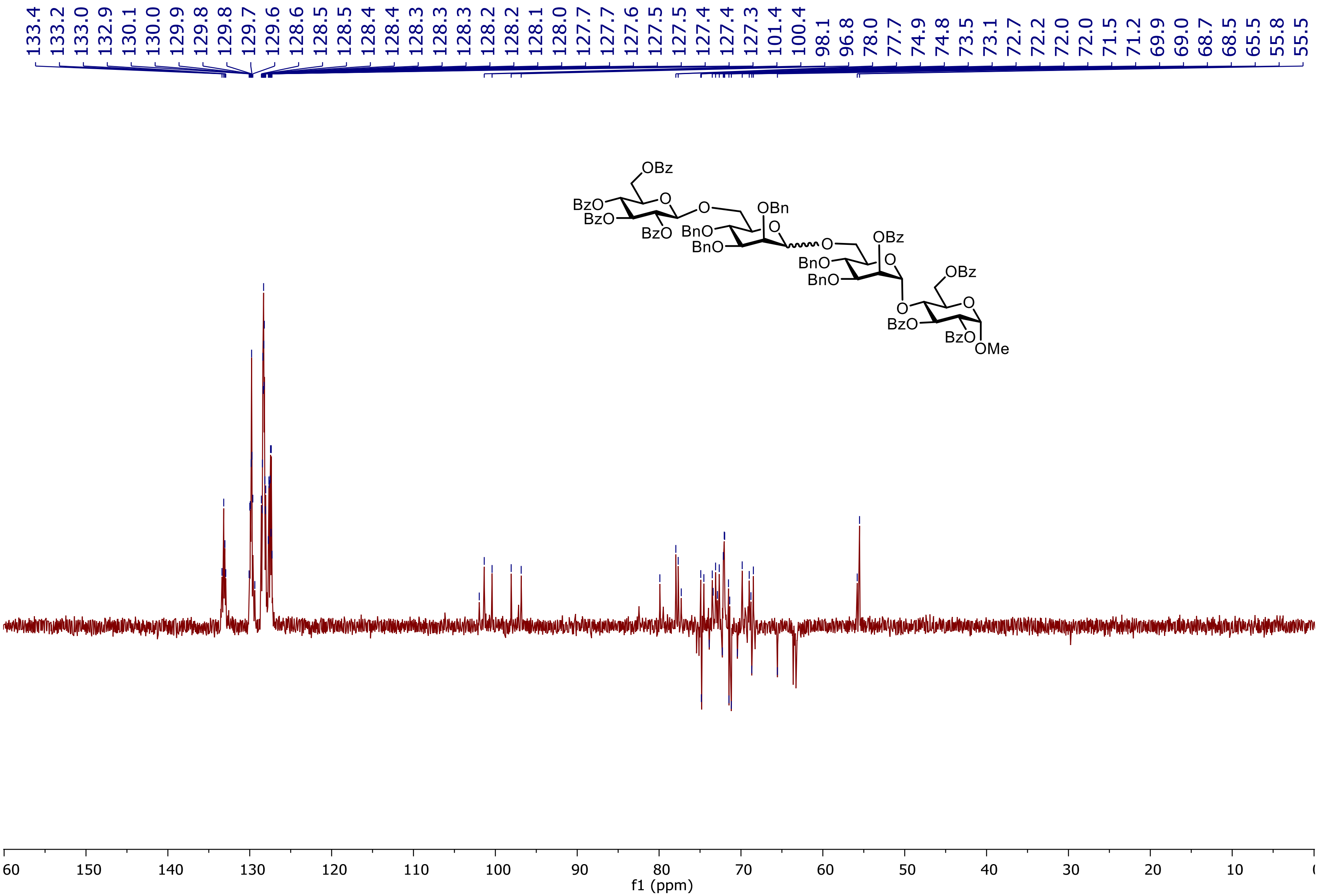

Supplementary Figure S39a. ^{1}H NMR Spectrum (600 MHz, $CDCl_3$) of compound **11d**

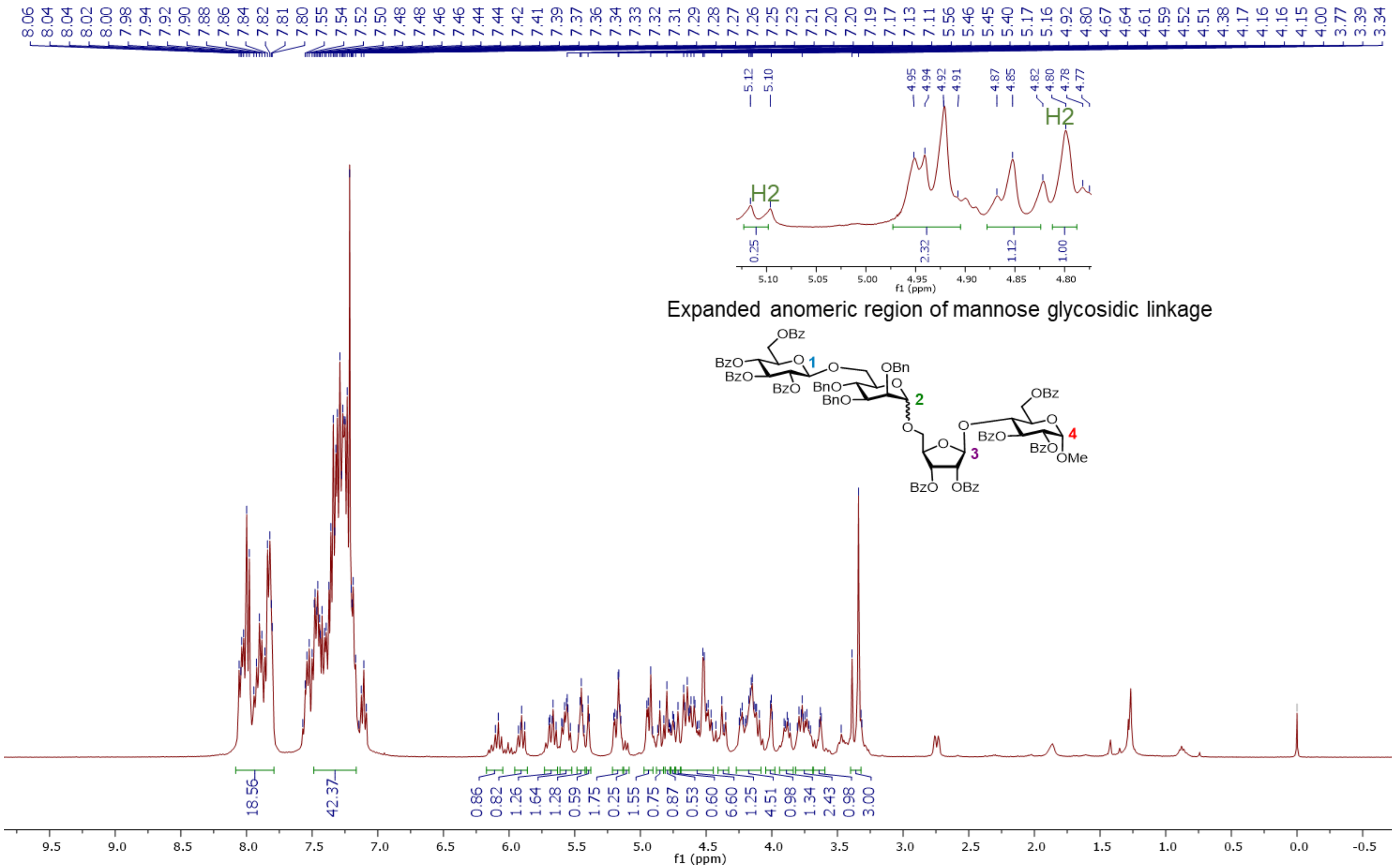

Supplementary Figure S39b. ^{13}C NMR Spectrum (151 MHz, $CDCl_3$) of compound **11d**

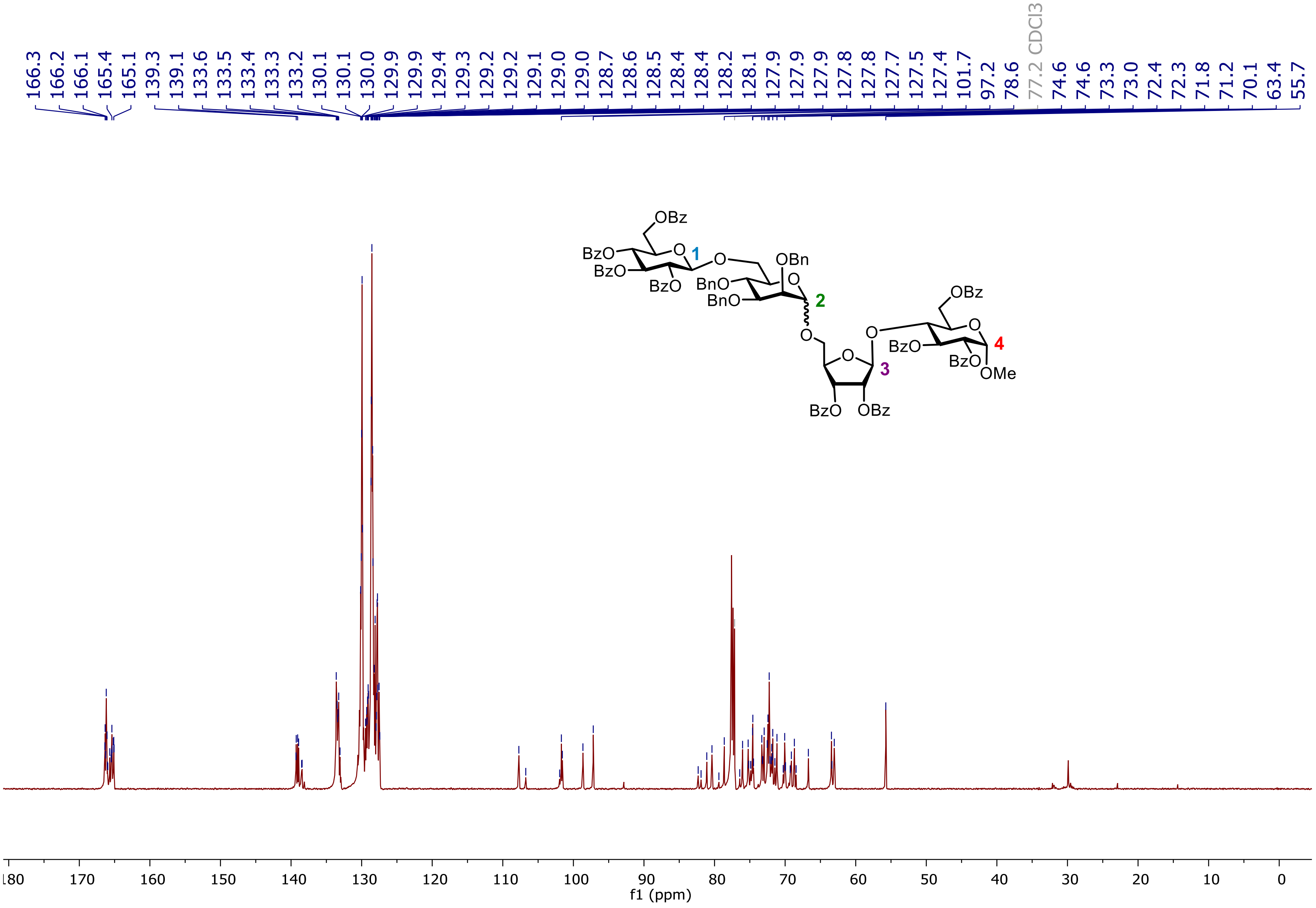

Supplementary Figure S39c. DEPT NMR Spectrum (151MHz, $CDCl_3$) of compound **11d**

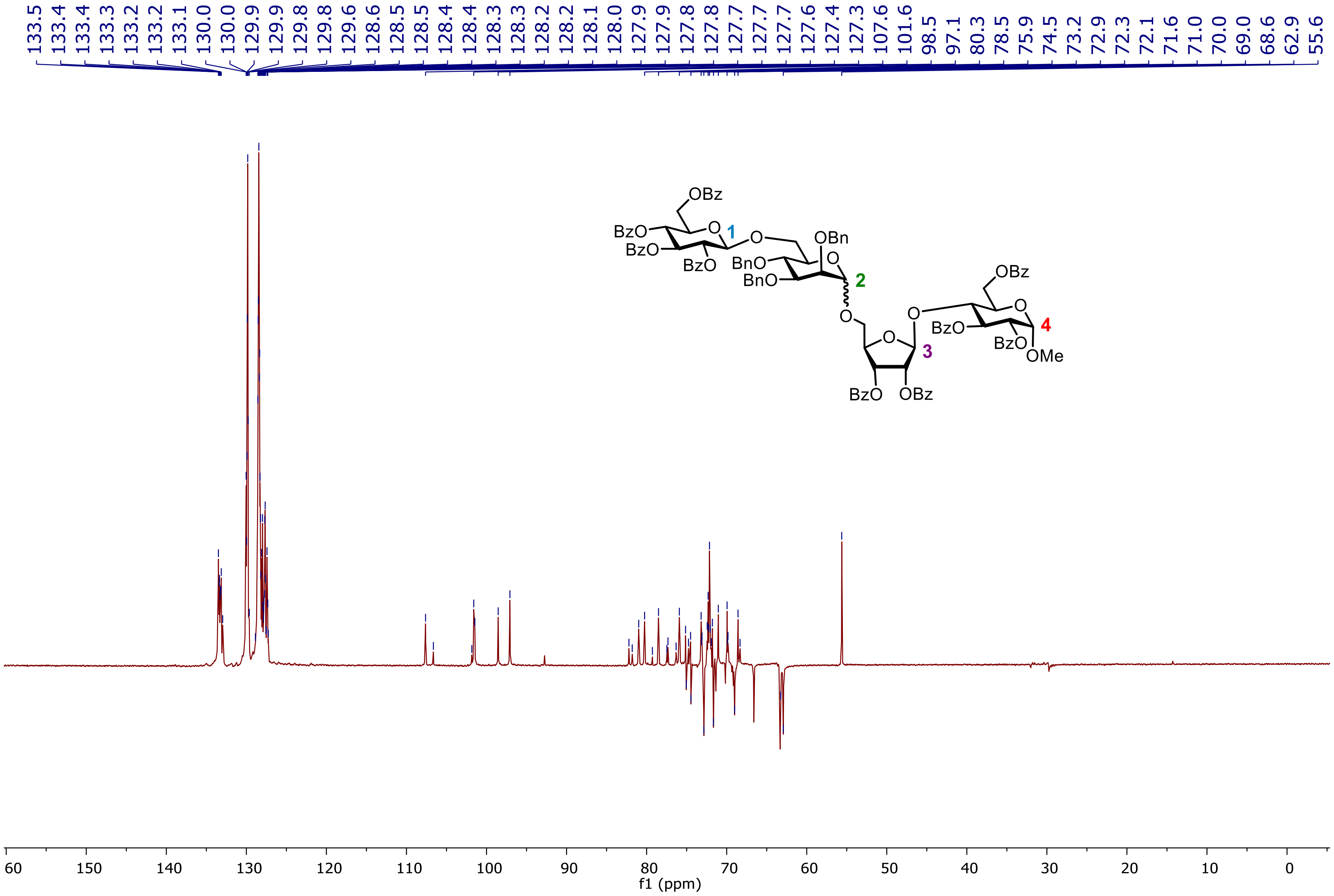

Supplementary Figure S39d. ^{1}H-^{13}C HSQC NMR Spectrum (600 MHz, 151 MHz, $CDCl_3$) of Compound **11d** (Anomeric region Expanded)

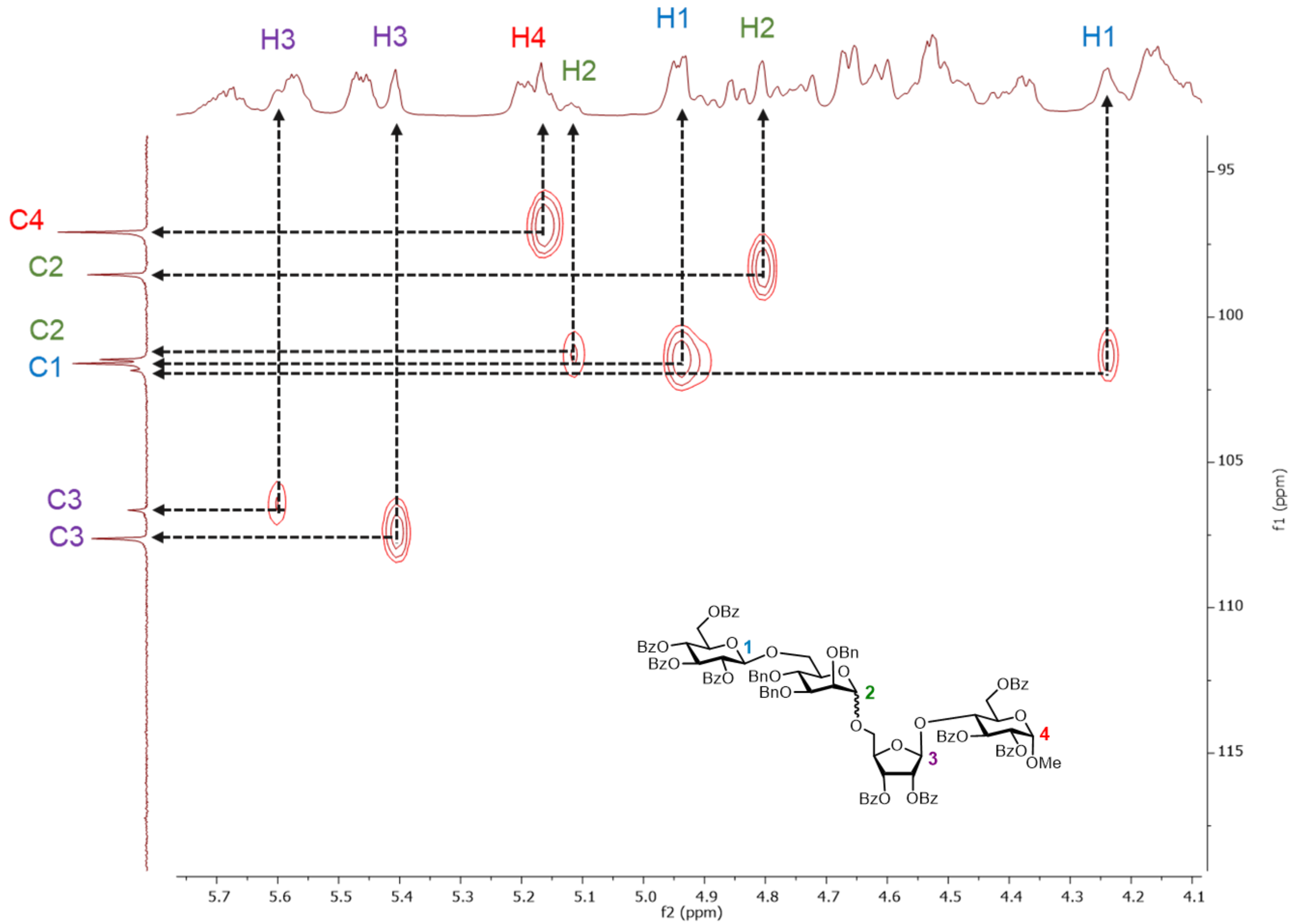

Supplementary Figure S39e. ^{1}H-^{13}C HSQC-Coupled NMR Spectrum (600 MHz, 151 MHz, $CDCl_3$) of Compound **11d** (Anomeric region Expanded)

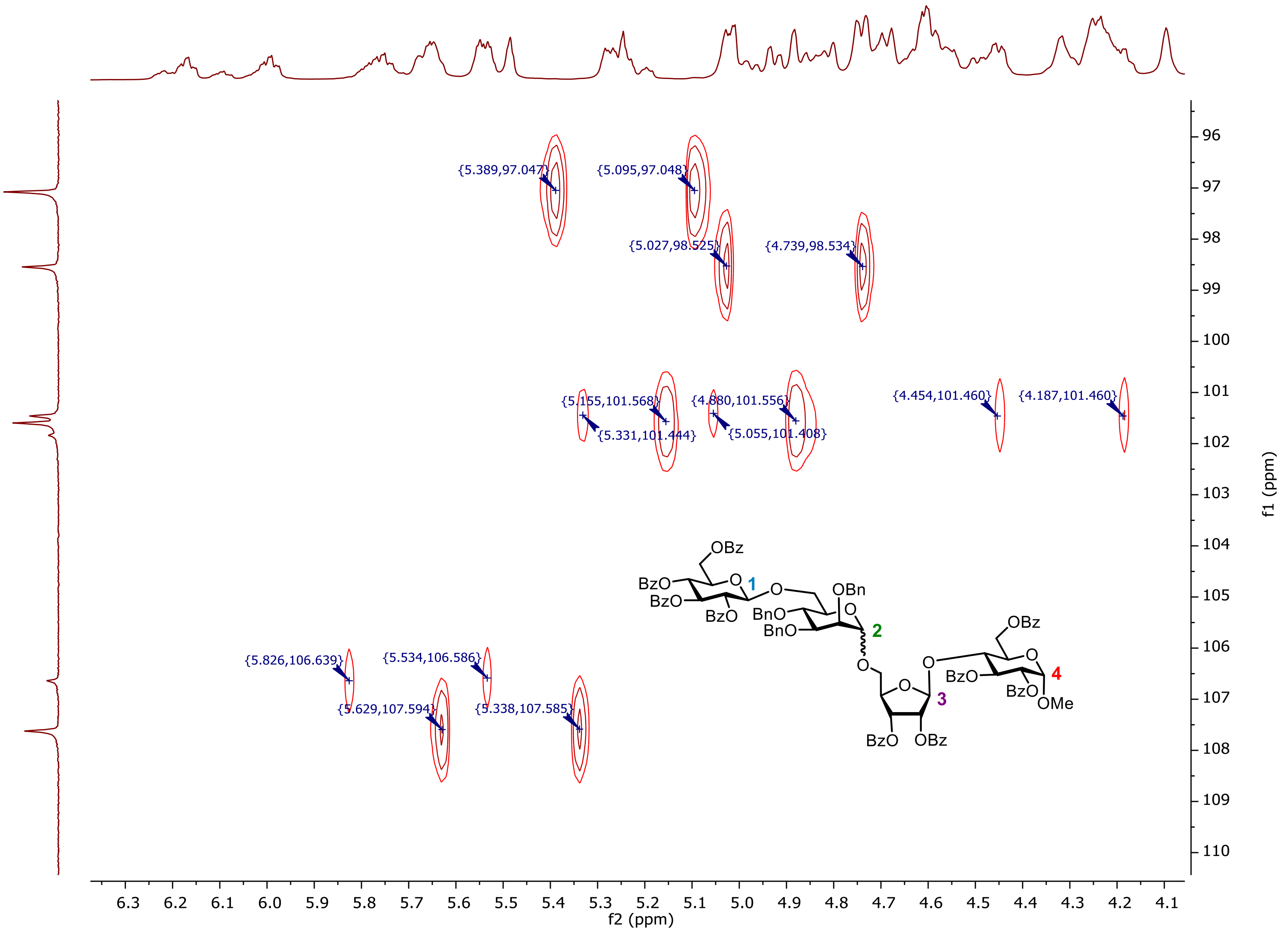

Supplementary Figure S40a. ^{1}H NMR Spectrum (600 MHz, $CDCl_3$) of compound **11e**

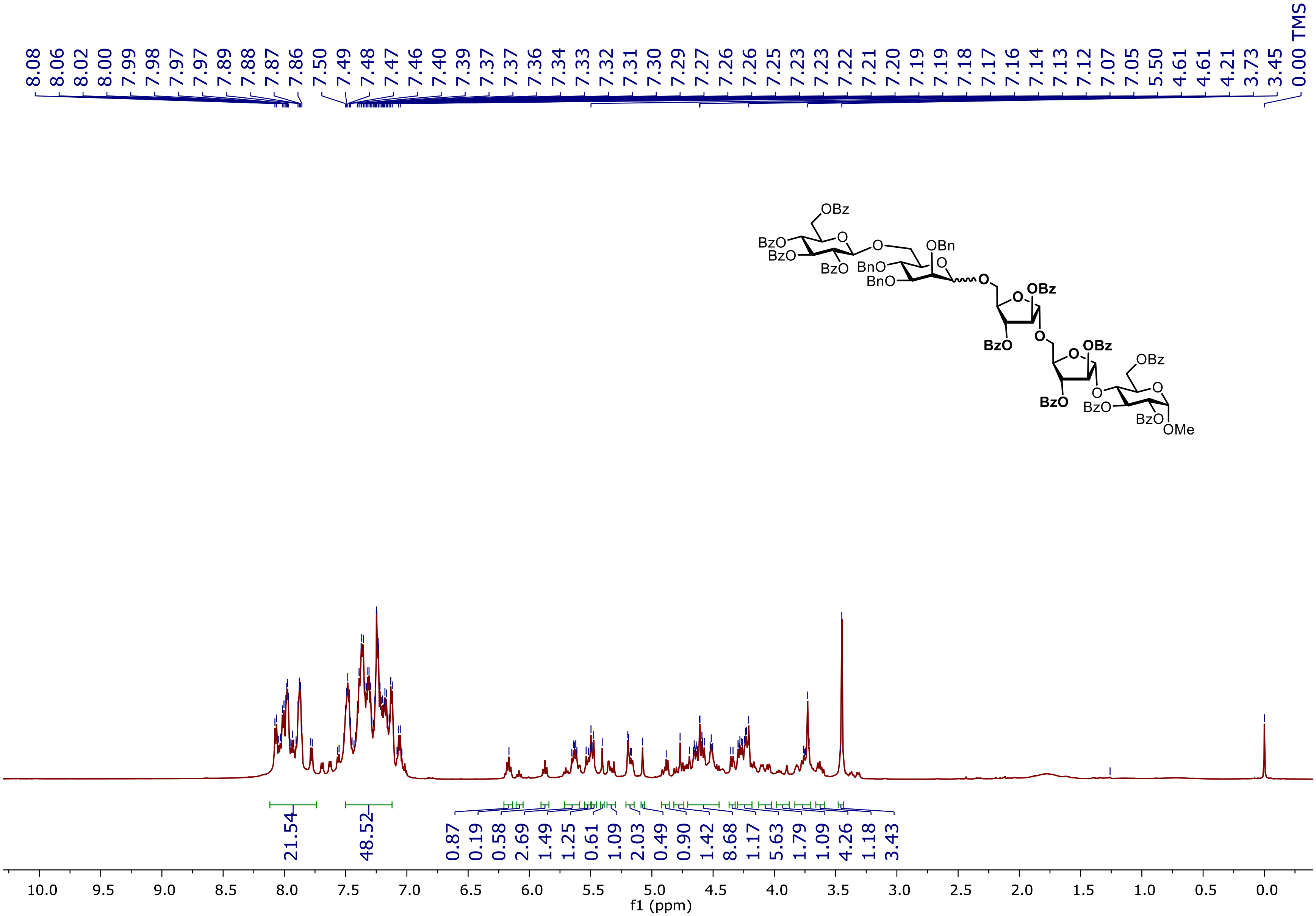

Supplementary Figure S40b. ^{13}C NMR Spectrum (151 MHz, $CDCl_3$) of compound **11e**

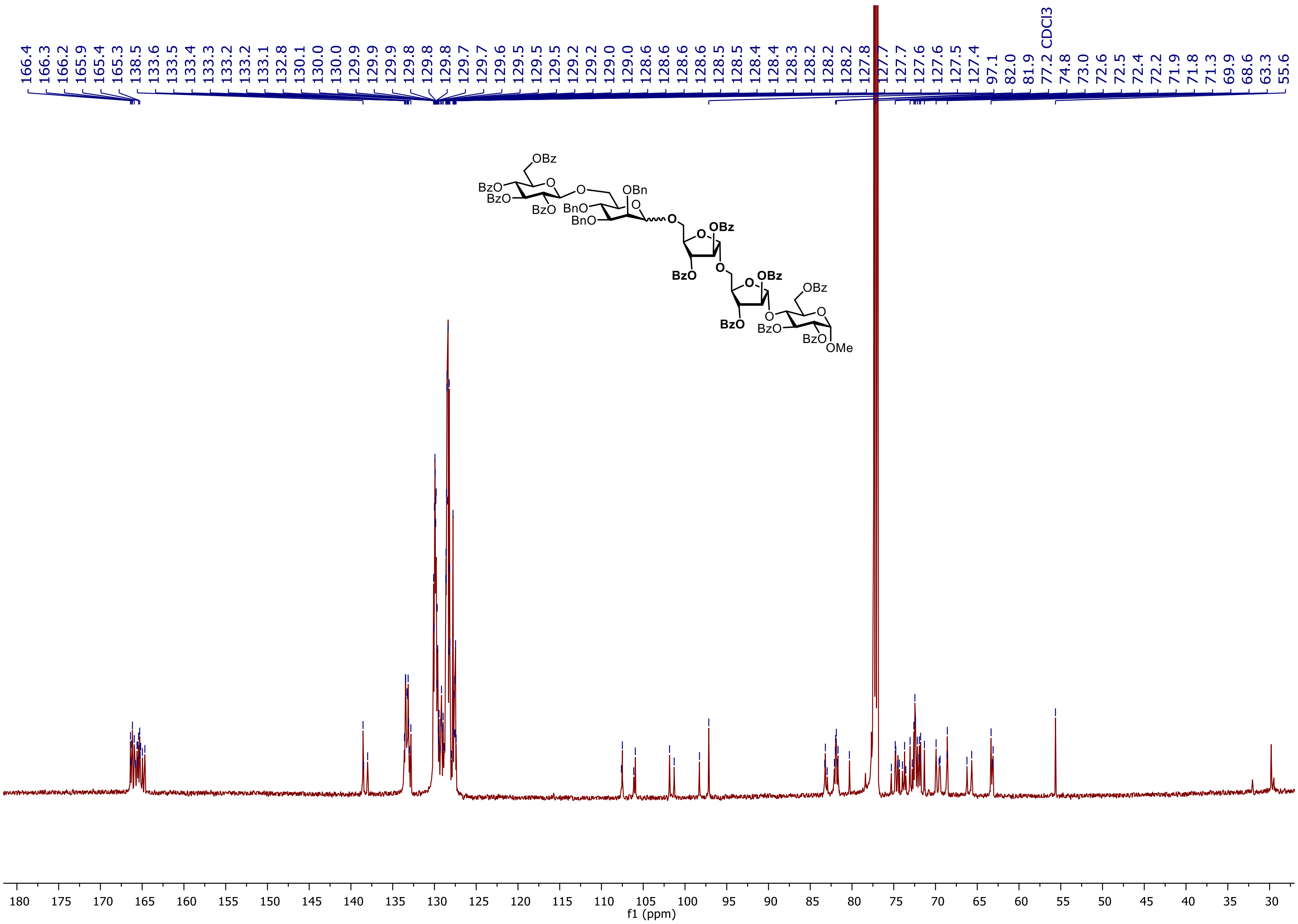

Supplementary Figure S40c. DEPT NMR Spectrum (151 MHz, $CDCl_3$) of compound **11e**

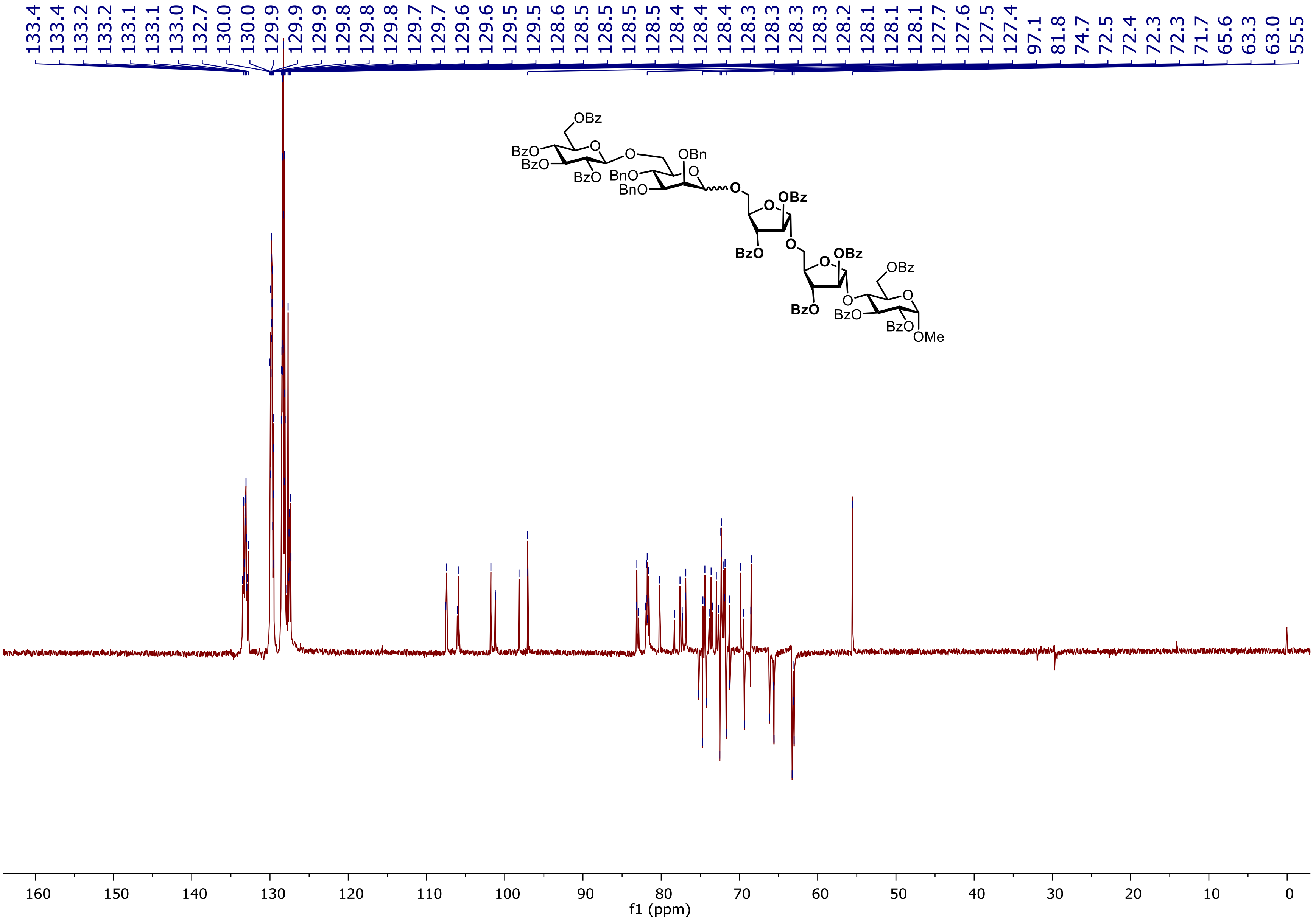

Supplementary Figure S41a. ^{1}H NMR Spectrum (600 MHz, $CDCl_3$) of compound **11f**

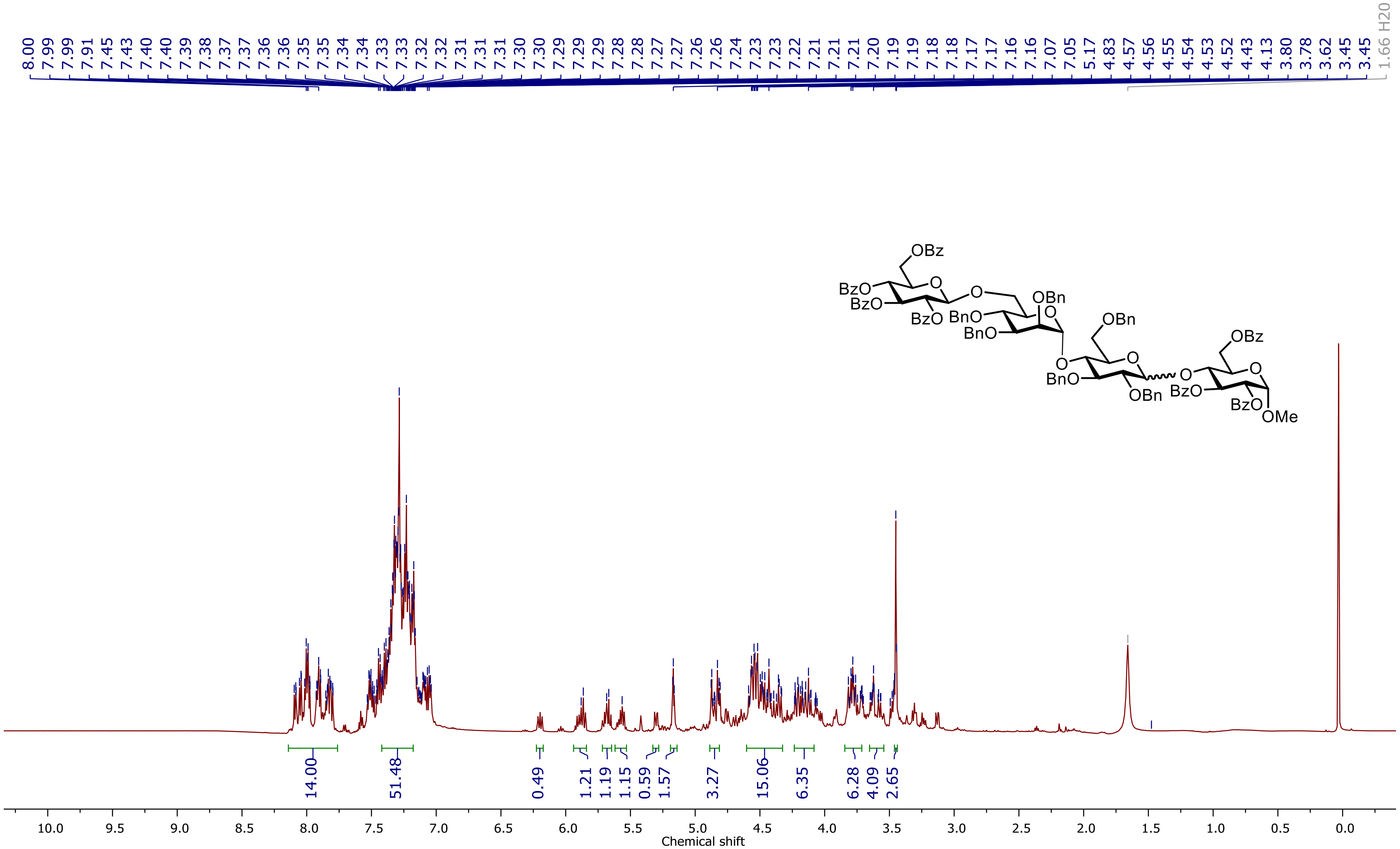

Supplementary Figure S42b. ^{13}C NMR Spectrum (600 MHz, $CDCl_3$) of compound **11f**

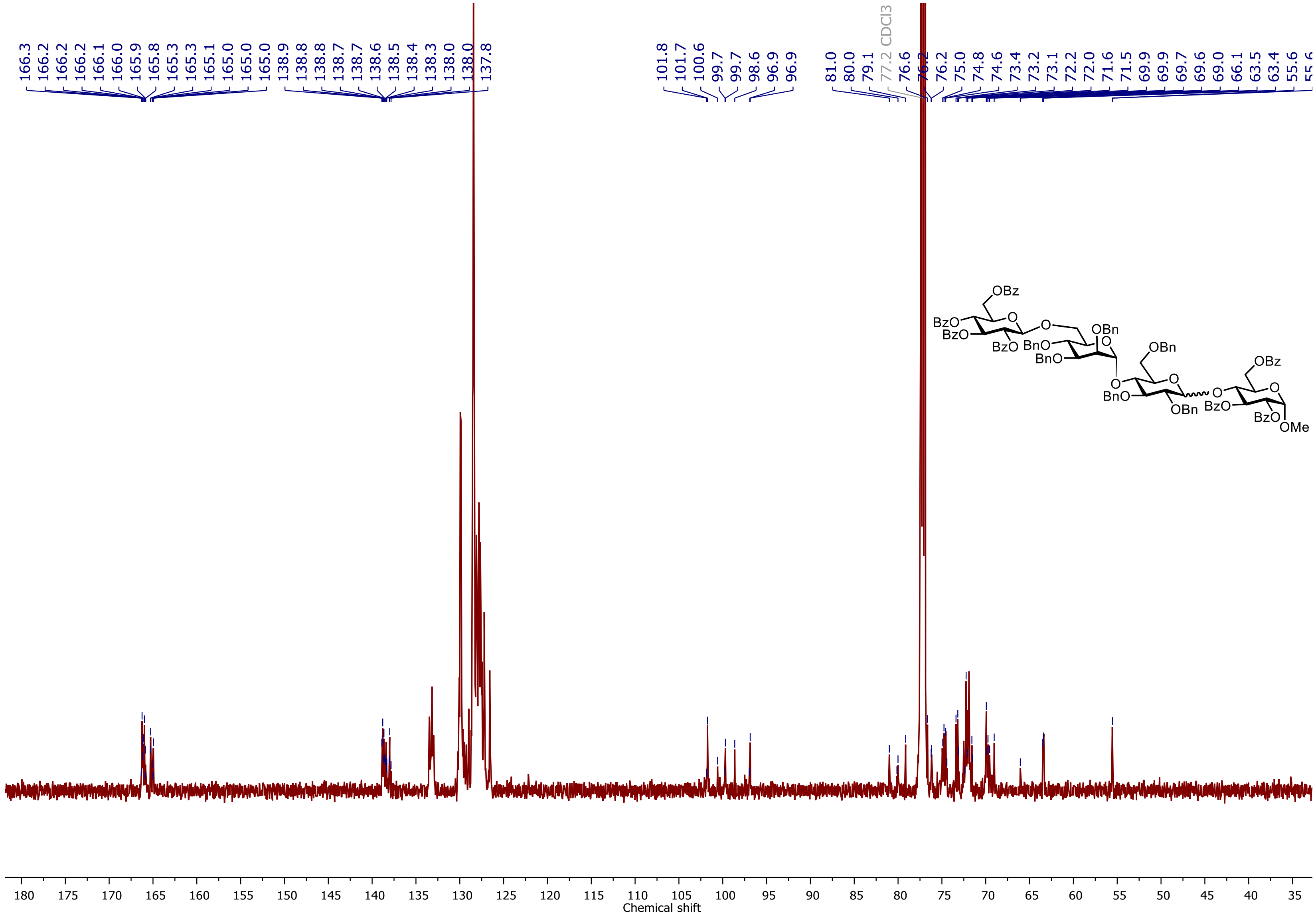

Supplementary Figure S42c. DEPT NMR Spectrum (151 MHz, $CDCl_3$) of compound **11f**

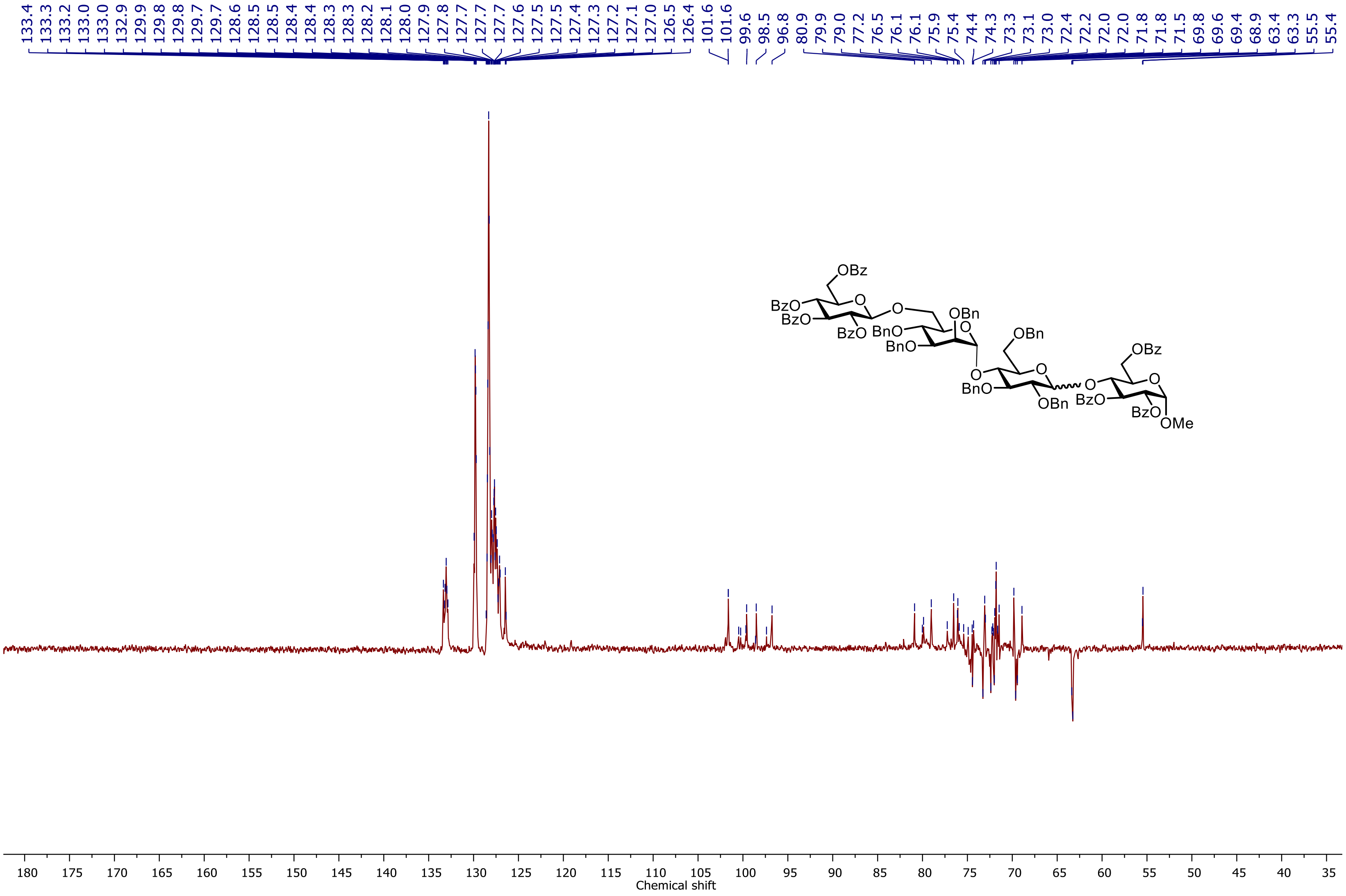

Supplementary Figure S43a. ^{1}H NMR Spectrum (101 MHz, $CDCl_3$) of compound **S40**

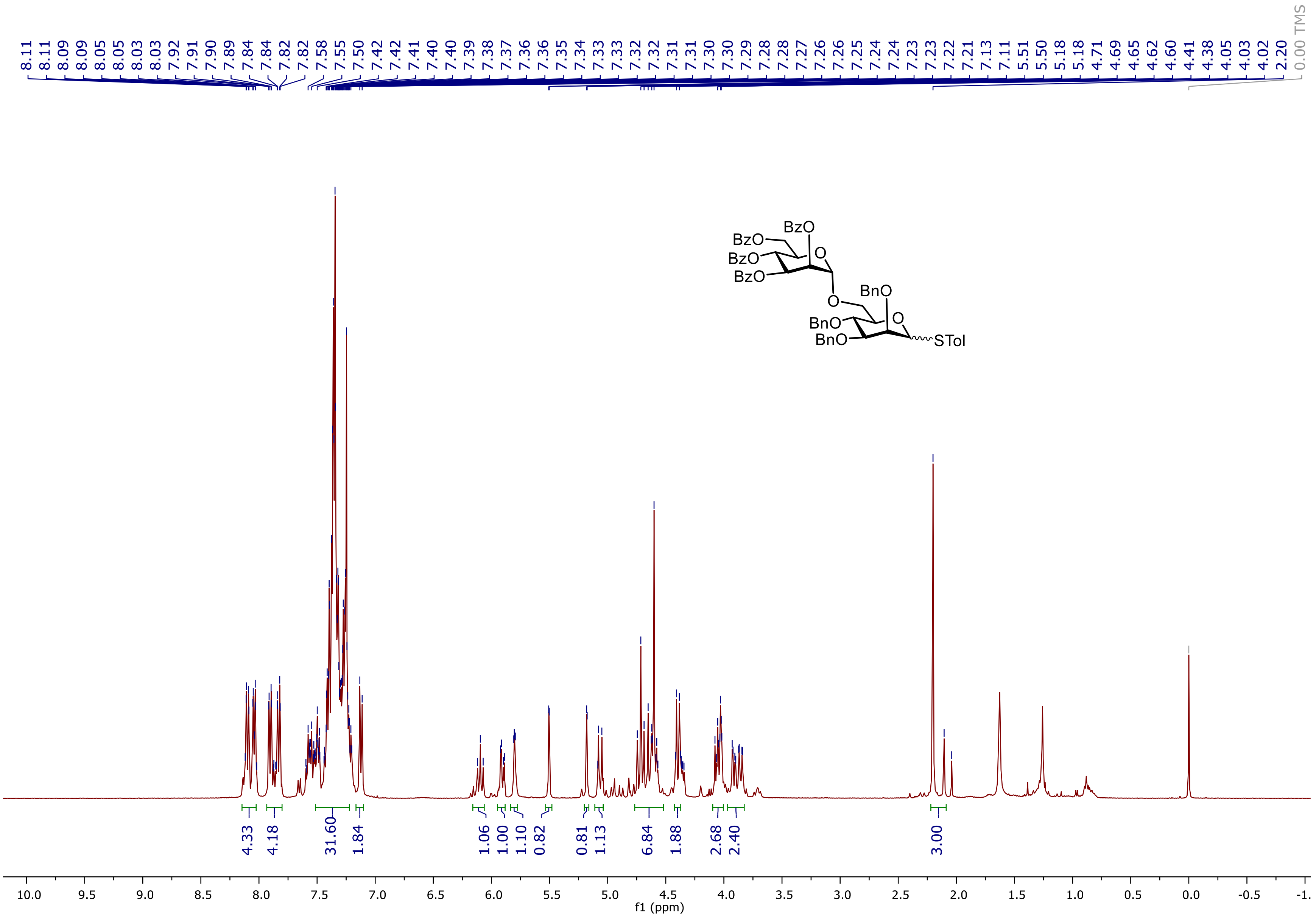

Supplementary Figure S42b. ^{13}C NMR Spectrum (101 MHz, $CDCl_3$) of compound **S40**

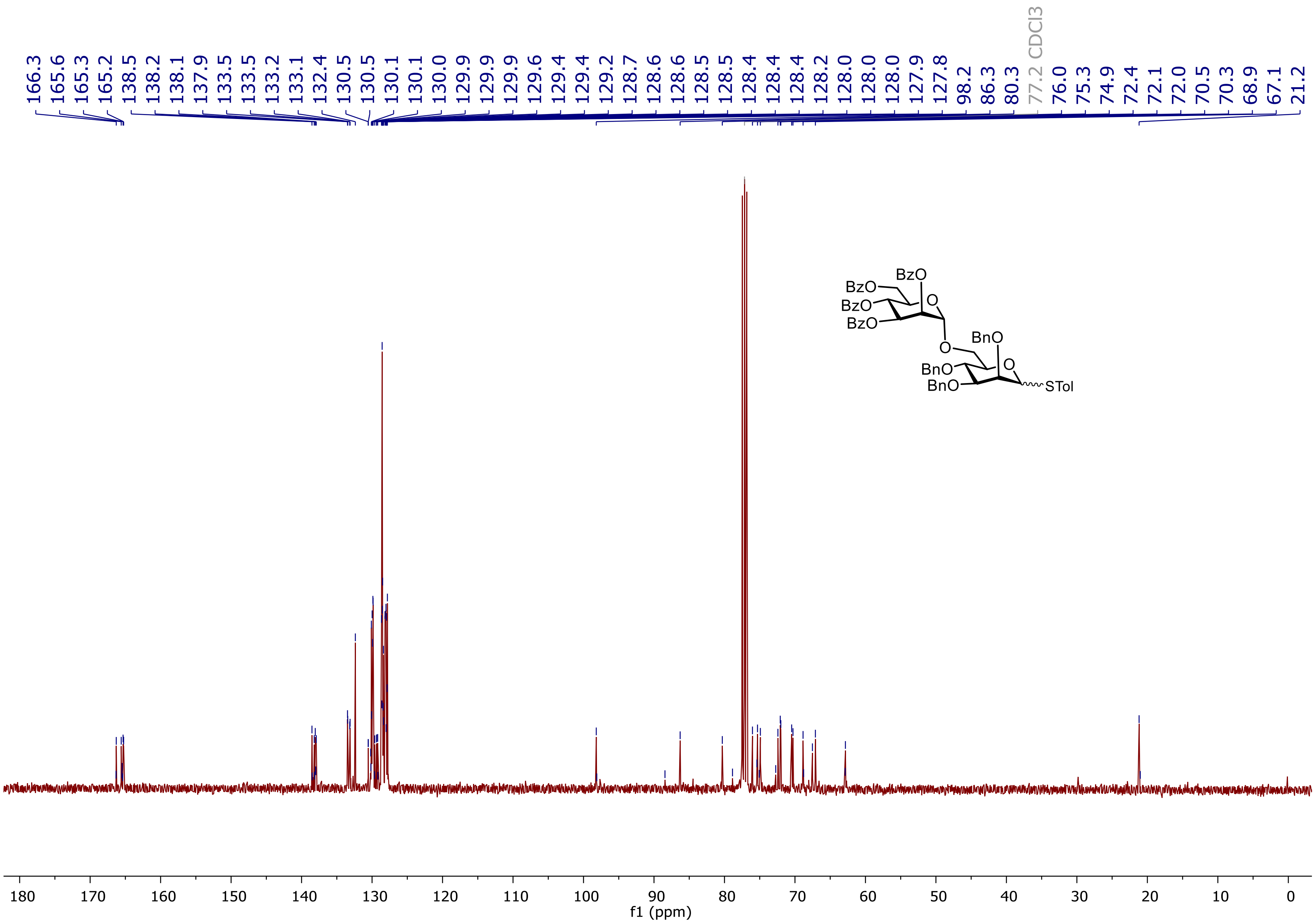

Supplementary Figure S42c. DEPT NMR Spectrum (101 MHz, $CDCl_3$) of compound **S40**

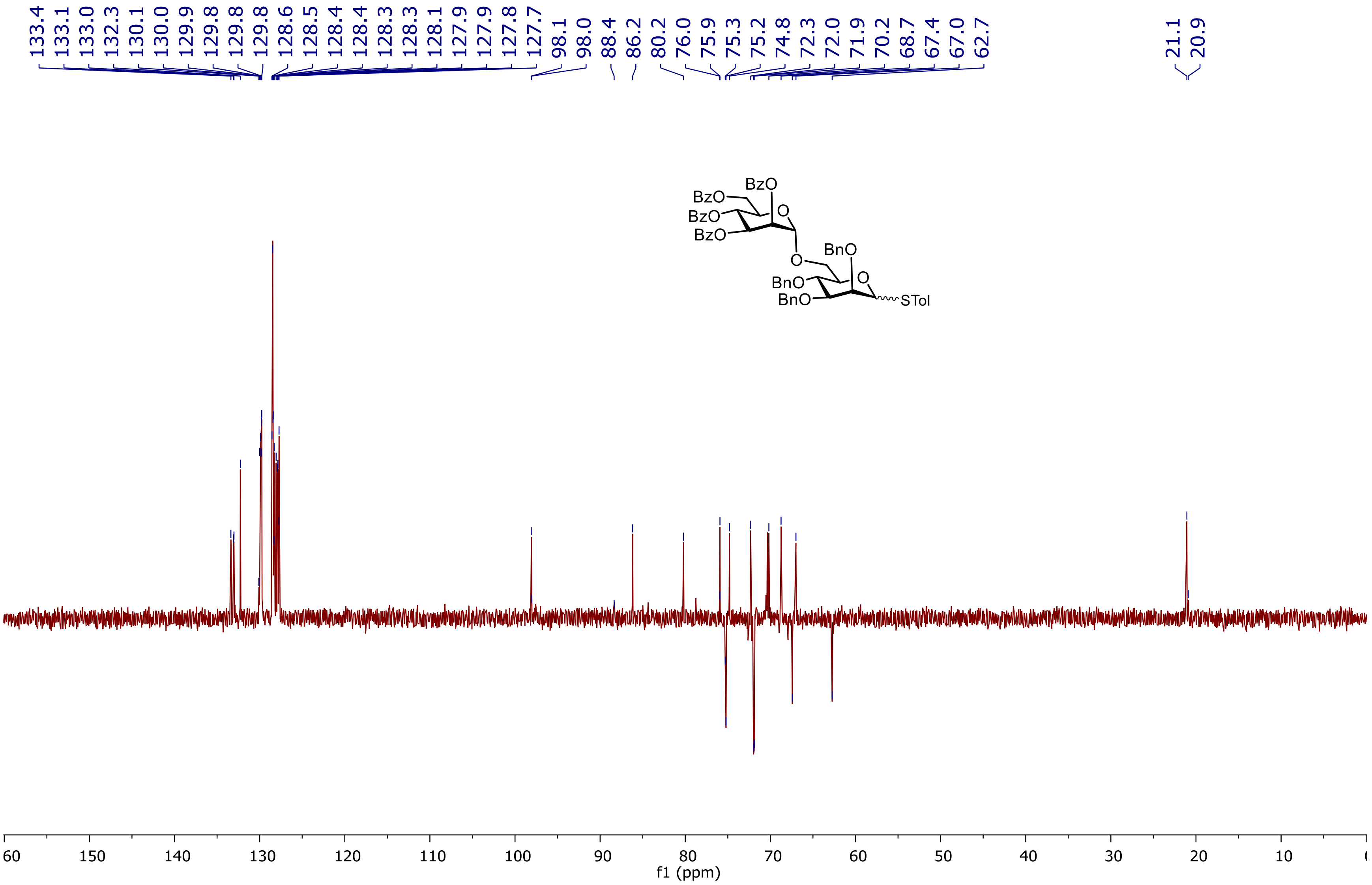

Supplementary Figure S43a. ^{1}H NMR Spectrum (600MHz, $CDCl_3$) of compound **25**

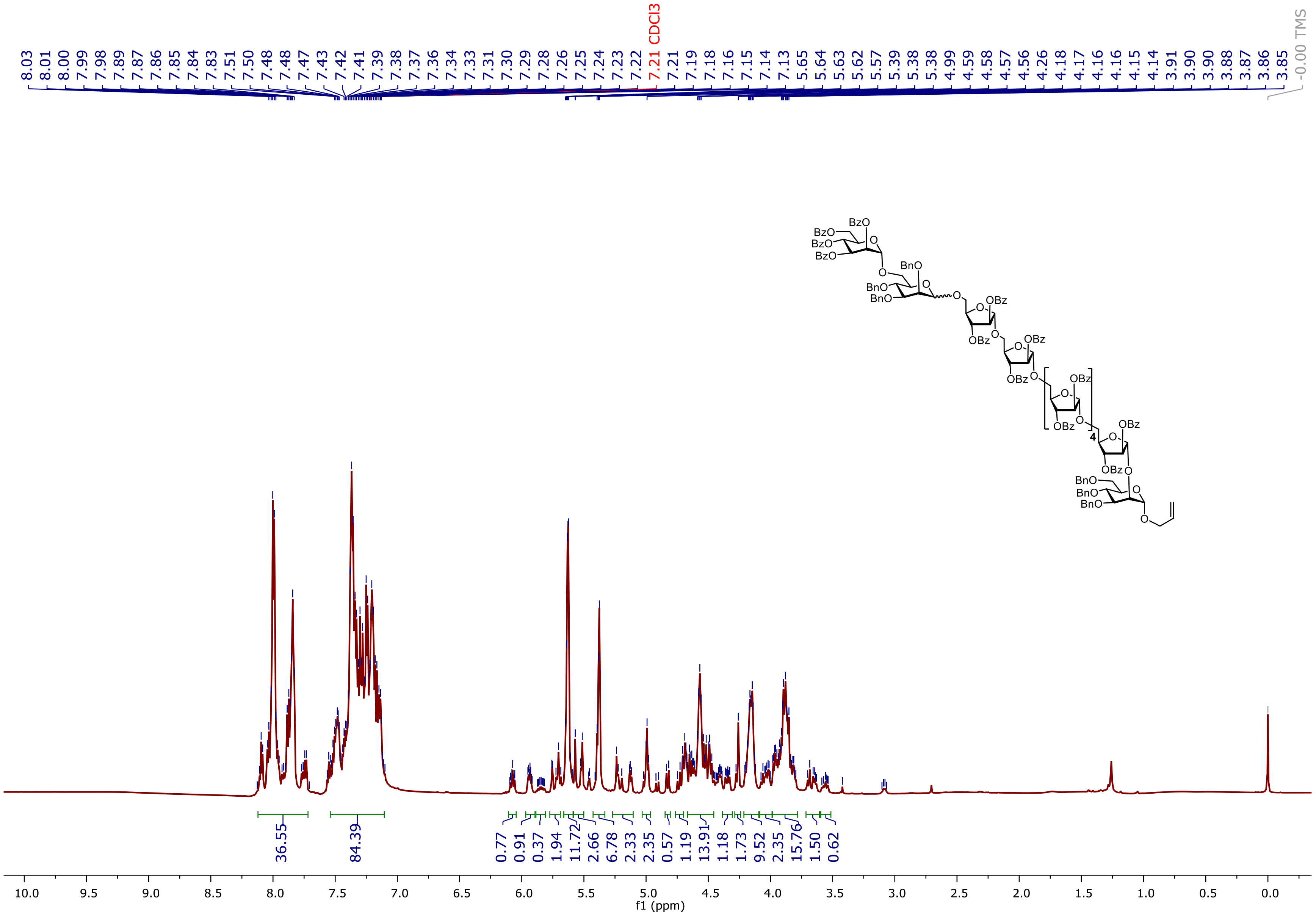

Supplementary Figure S43b. ^{13}C NMR Spectrum (151MHz, $CDCl_3$) of compound **25**

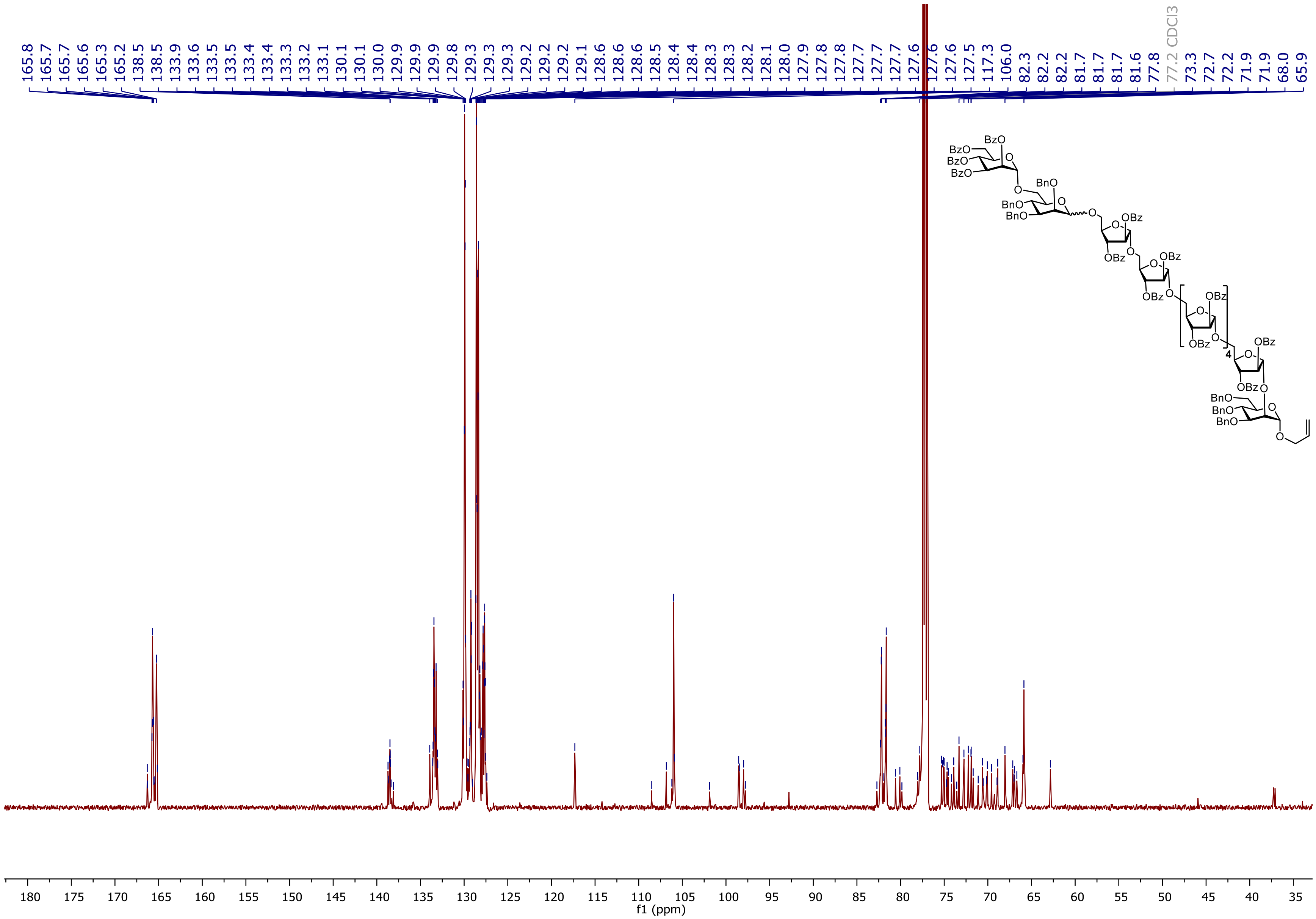

Supplementary Figure S43c. DEPT NMR Spectrum (151MHz, $CDCl_3$) of compound **25**

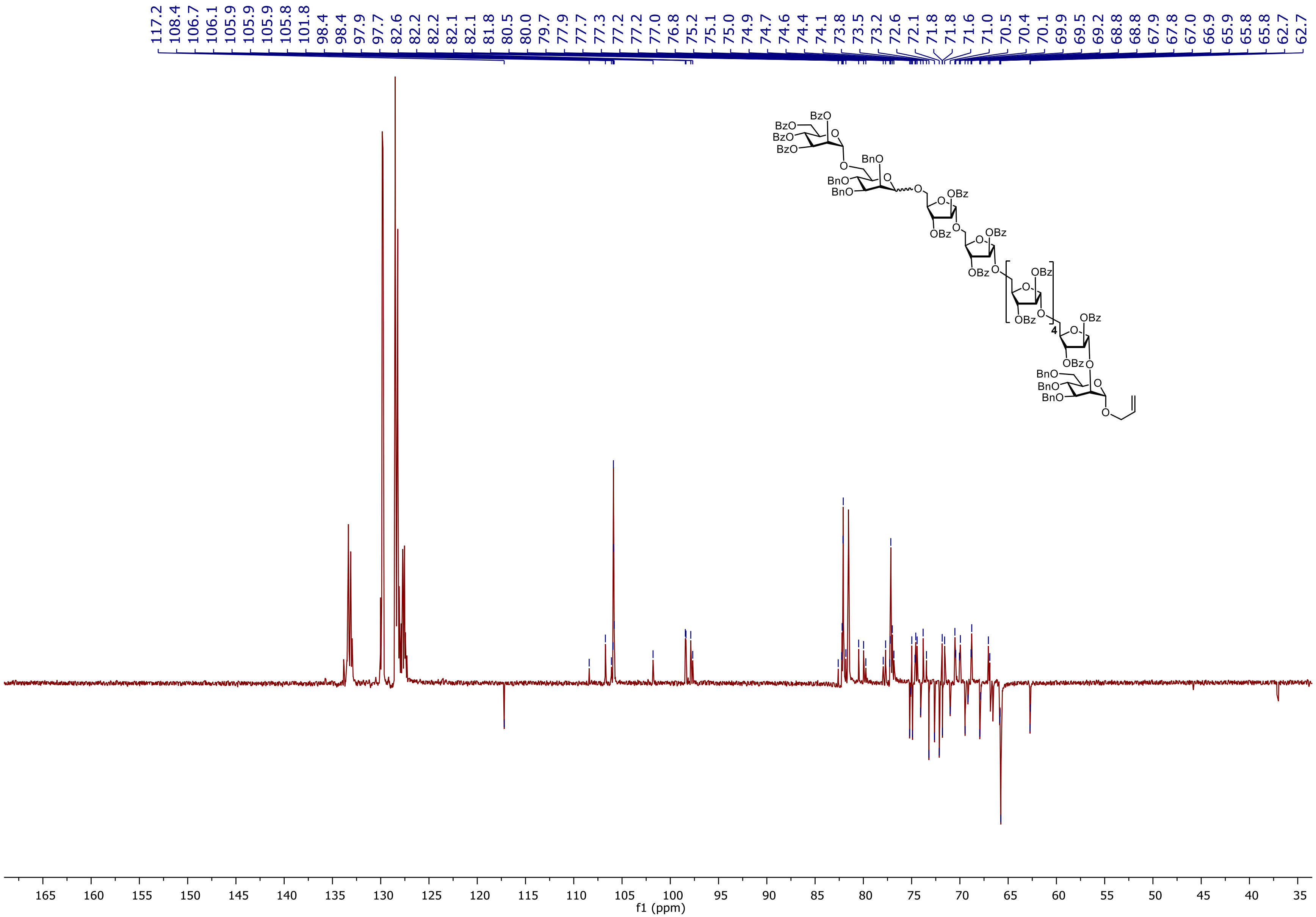

Supplementary Figure S44a. ^{1}H NMR Spectrum (600MHz, $CDCl_3$) of compound **27**

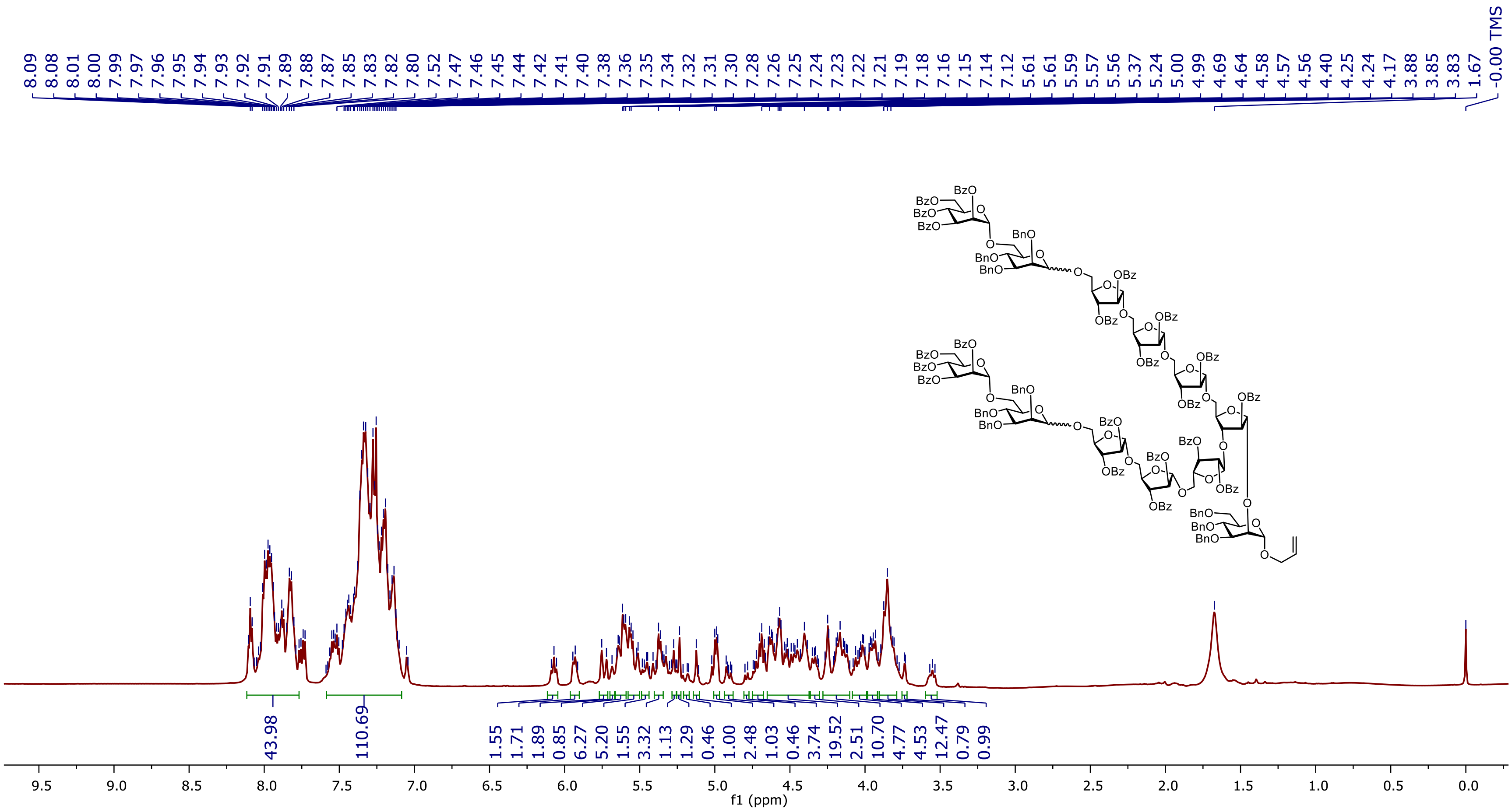

Supplementary Figure S44b. ^{13}C NMR Spectrum (151MHz, $CDCl_3$) of compound **27**

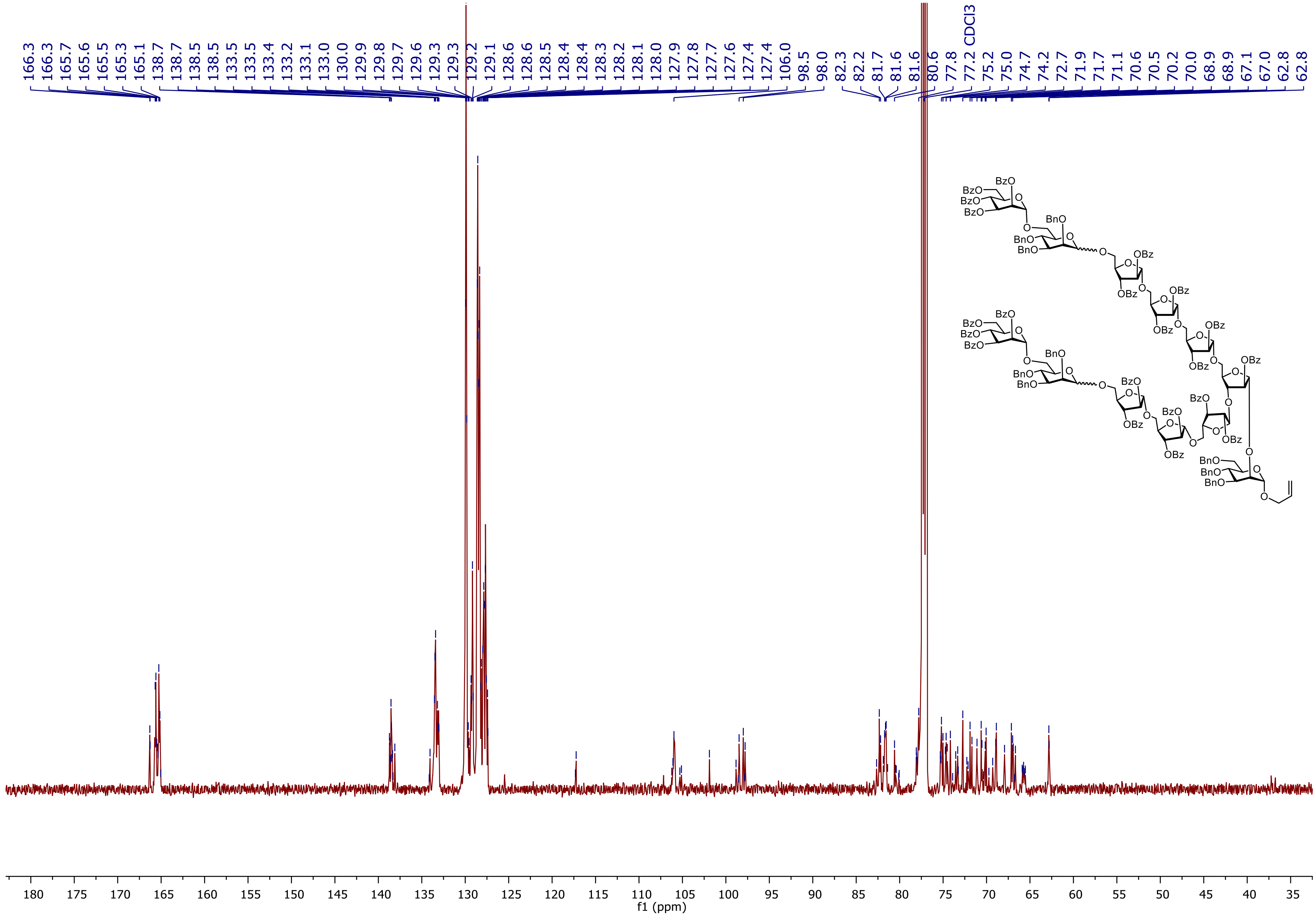

Supplementary Figure S44c. DEPT NMR Spectrum (151 MHz, $CDCl_3$) of compound **27**

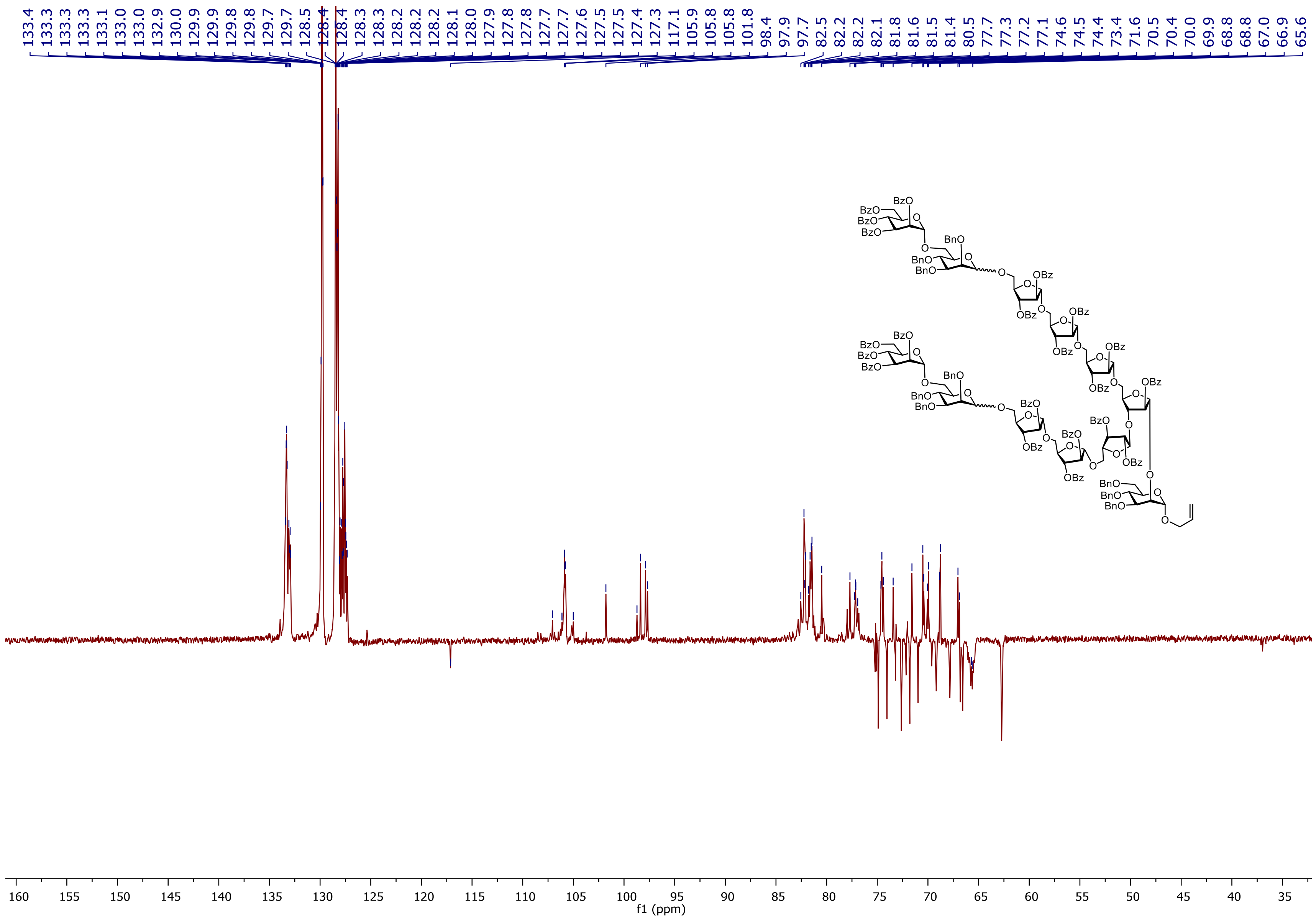

Supplementary Figure S45a. ^{1}H NMR Spectrum (400MHz, $CDCl_3$) of compound **21**

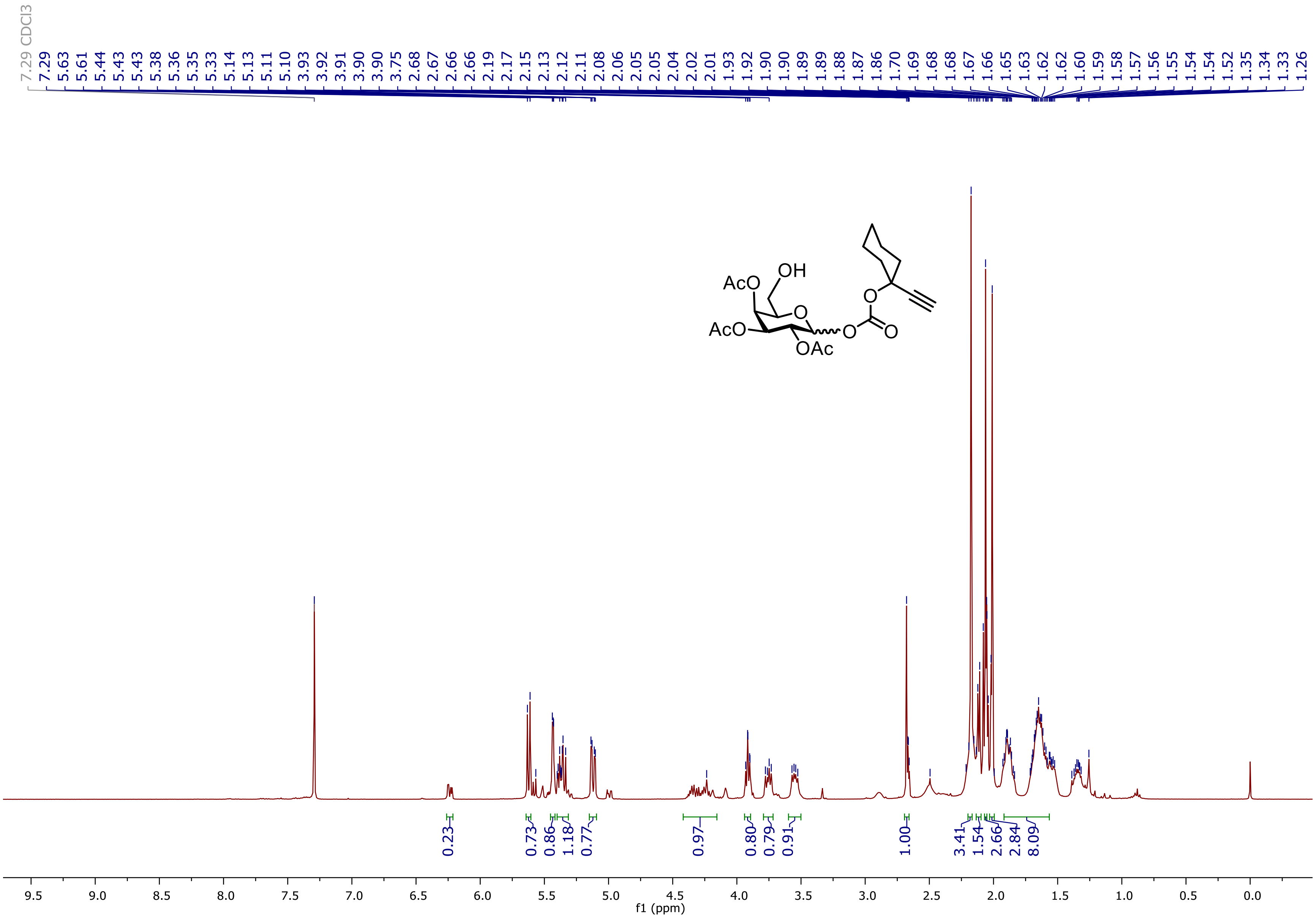

Supplementary Figure S45b. ^{13}C NMR Spectrum (101MHz, $CDCl_3$) of compound **21**

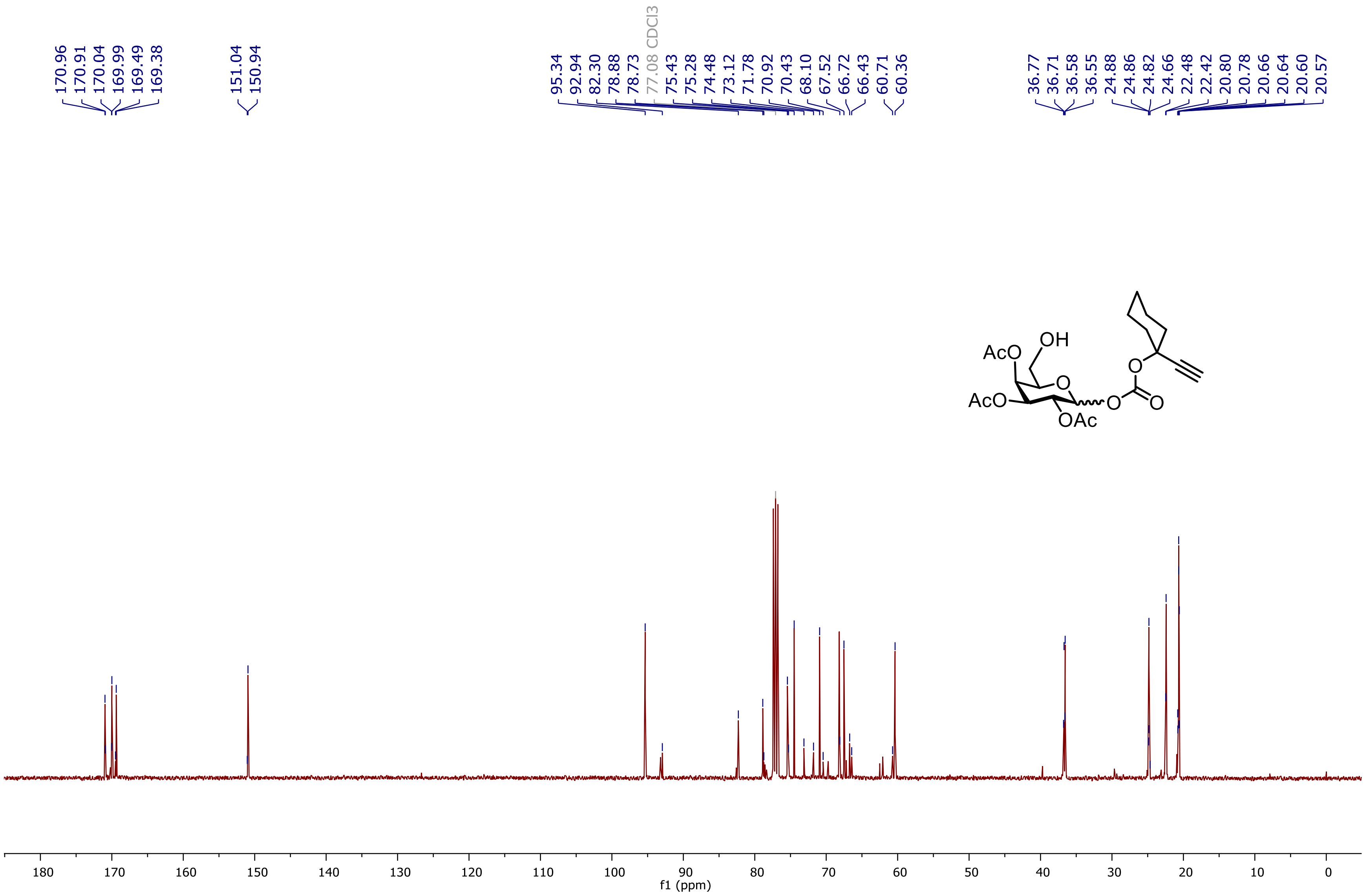

Supplementary Figure S45c. DEPT NMR Spectrum (101 MHz, $CDCl_3$) of compound **21**

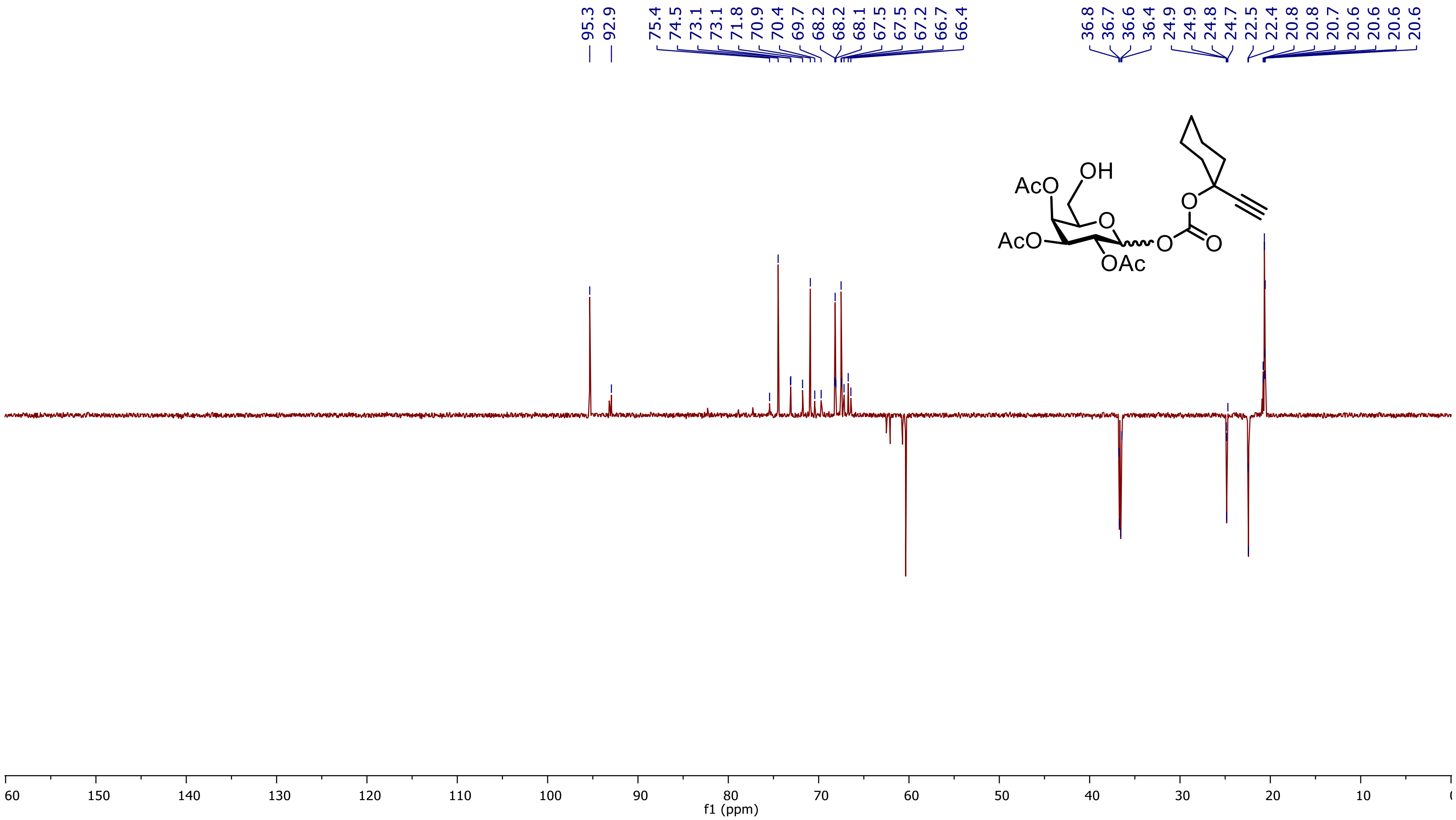

Supplementary Figure S46a. ^{1}H NMR Spectrum (600MHz, D_2O) of compound **22**

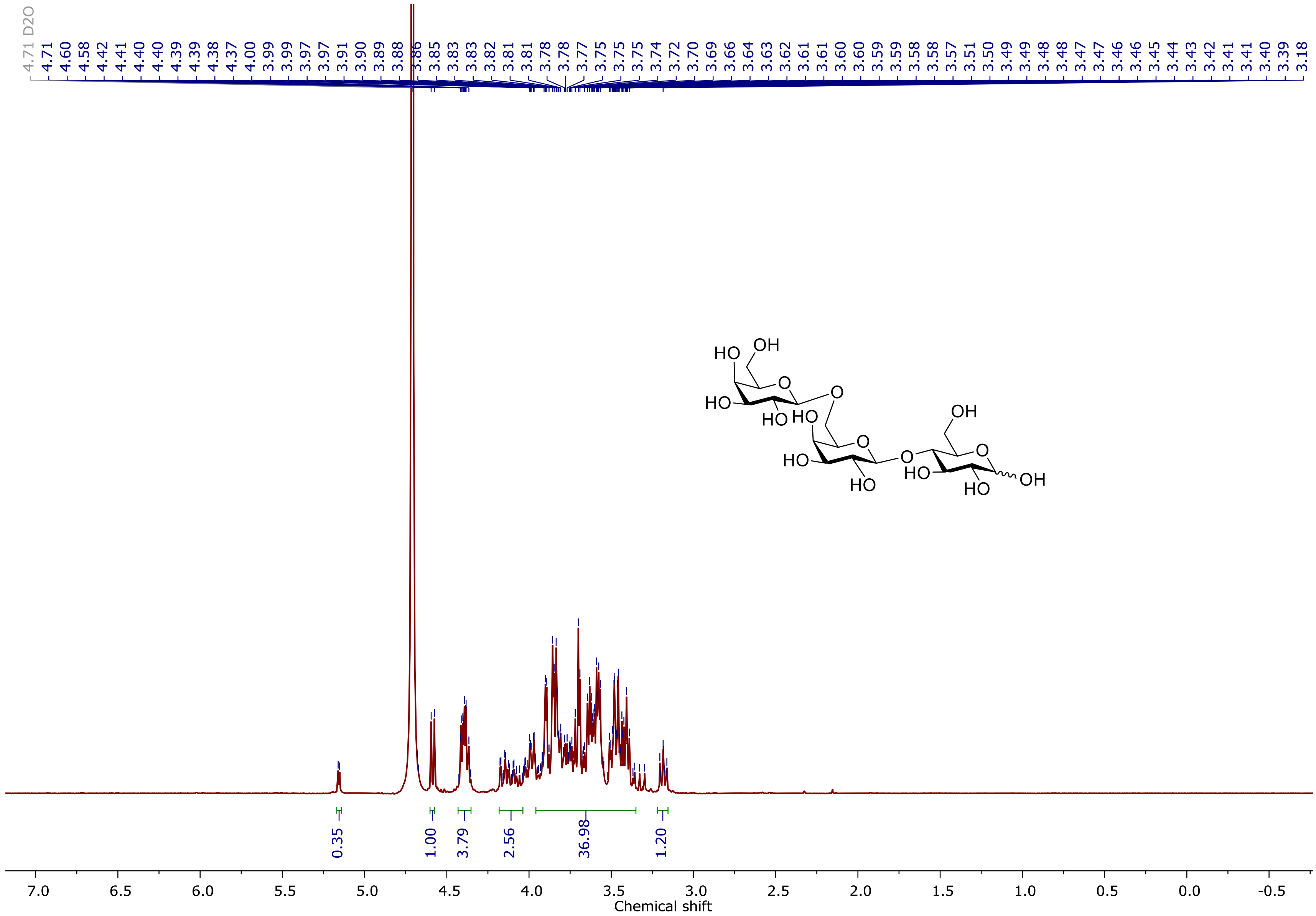

Supplementary Figure S46b. ^{13}C NMR Spectrum (151MHz, D_2O) of compound **22**

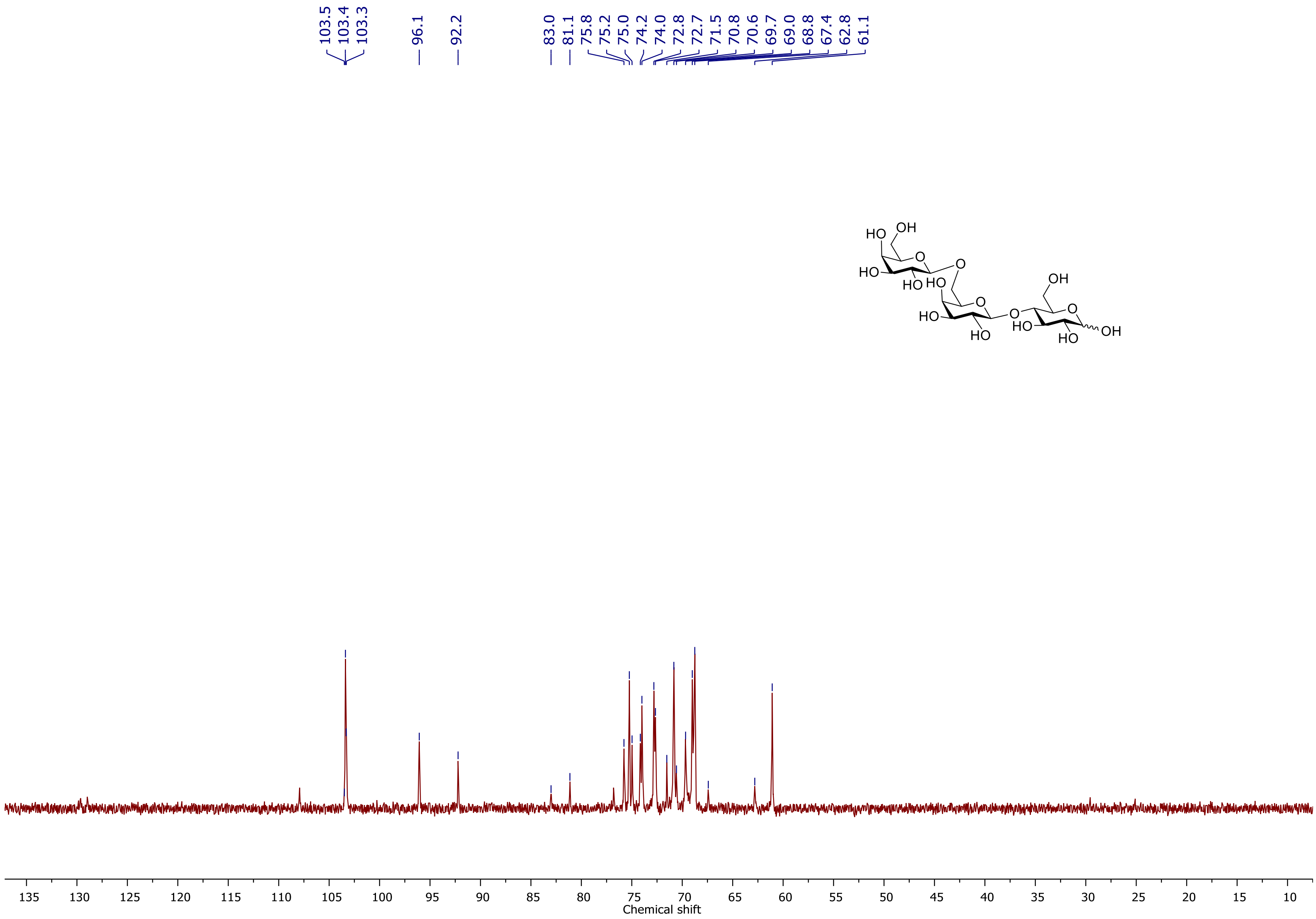

Supplementary Figure S46c. ^{1}H COSY NMR Spectrum (600 MHz, D_2O) of Compound **22**

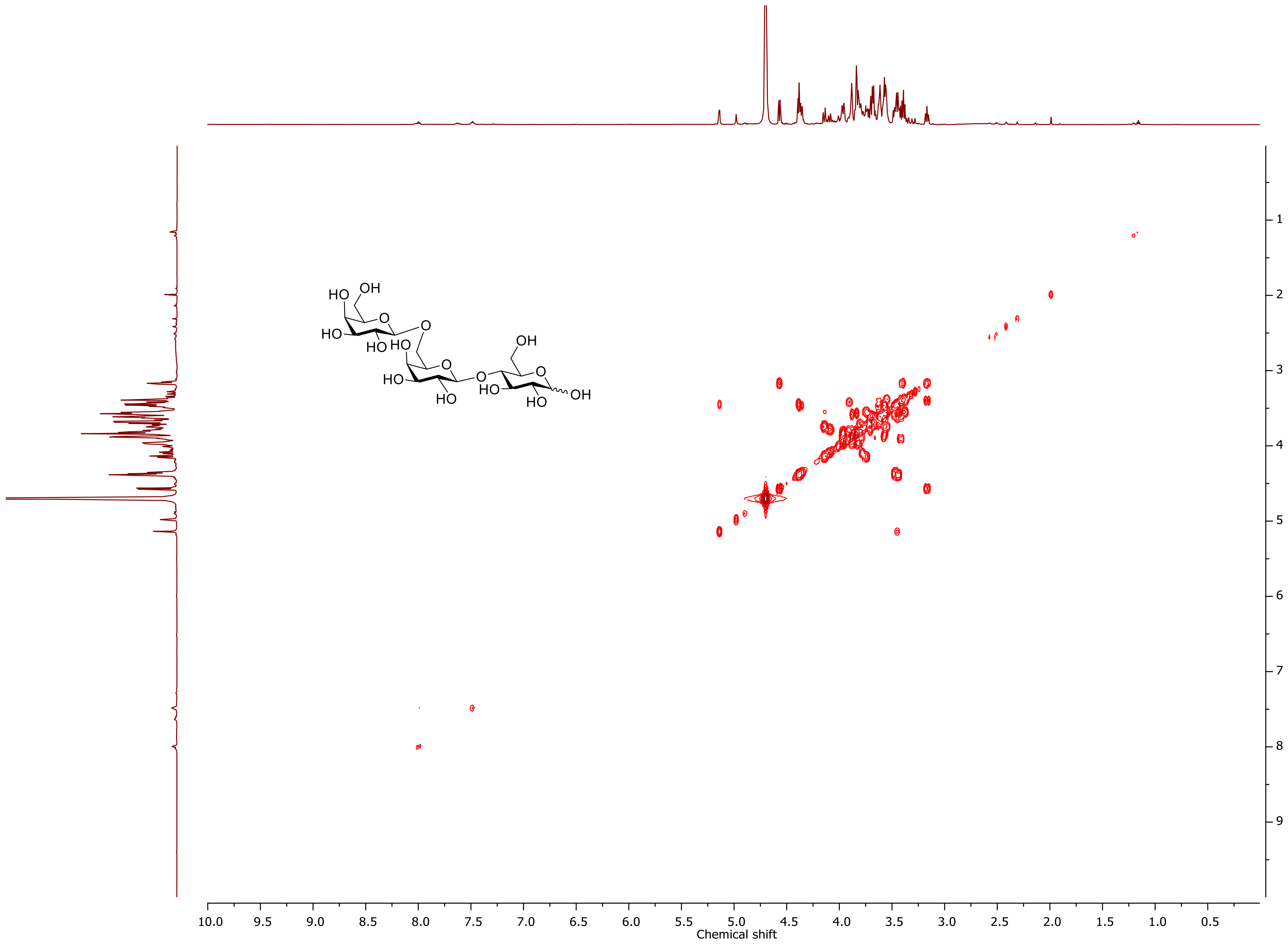

Supplementary Figure S46d. ^{1}H-^{13}C HSQC NMR Spectrum (600 MHz, 151 MHz D_2O) of Compound **22** (Anomeric Expansion)

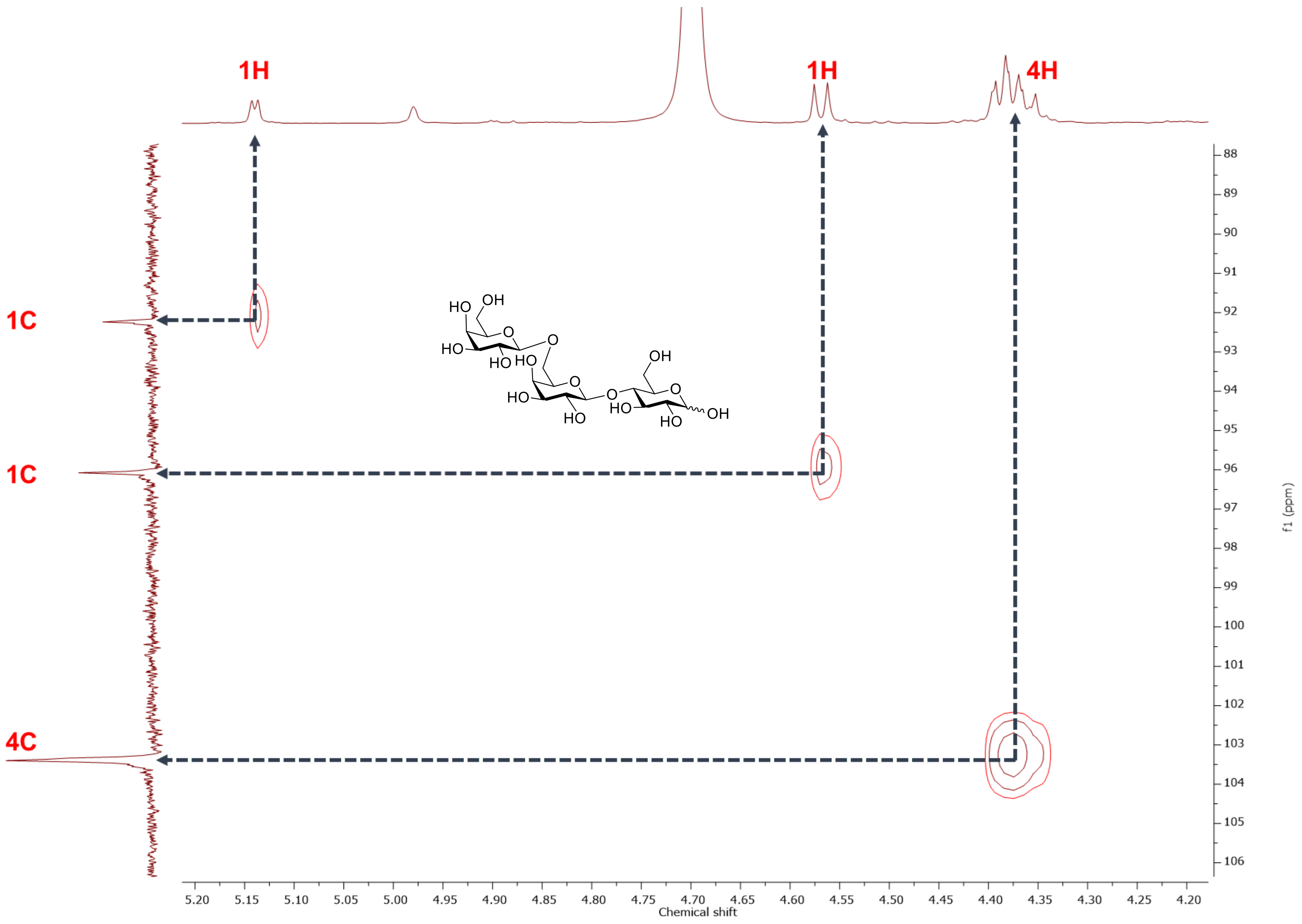

Supplementary Figure S46e. ^{1}H-^{13}C HSQC-Coupled NMR Spectrum (600 MHz, D_2O) of Compound **22** (Anomeric Region Expanded)

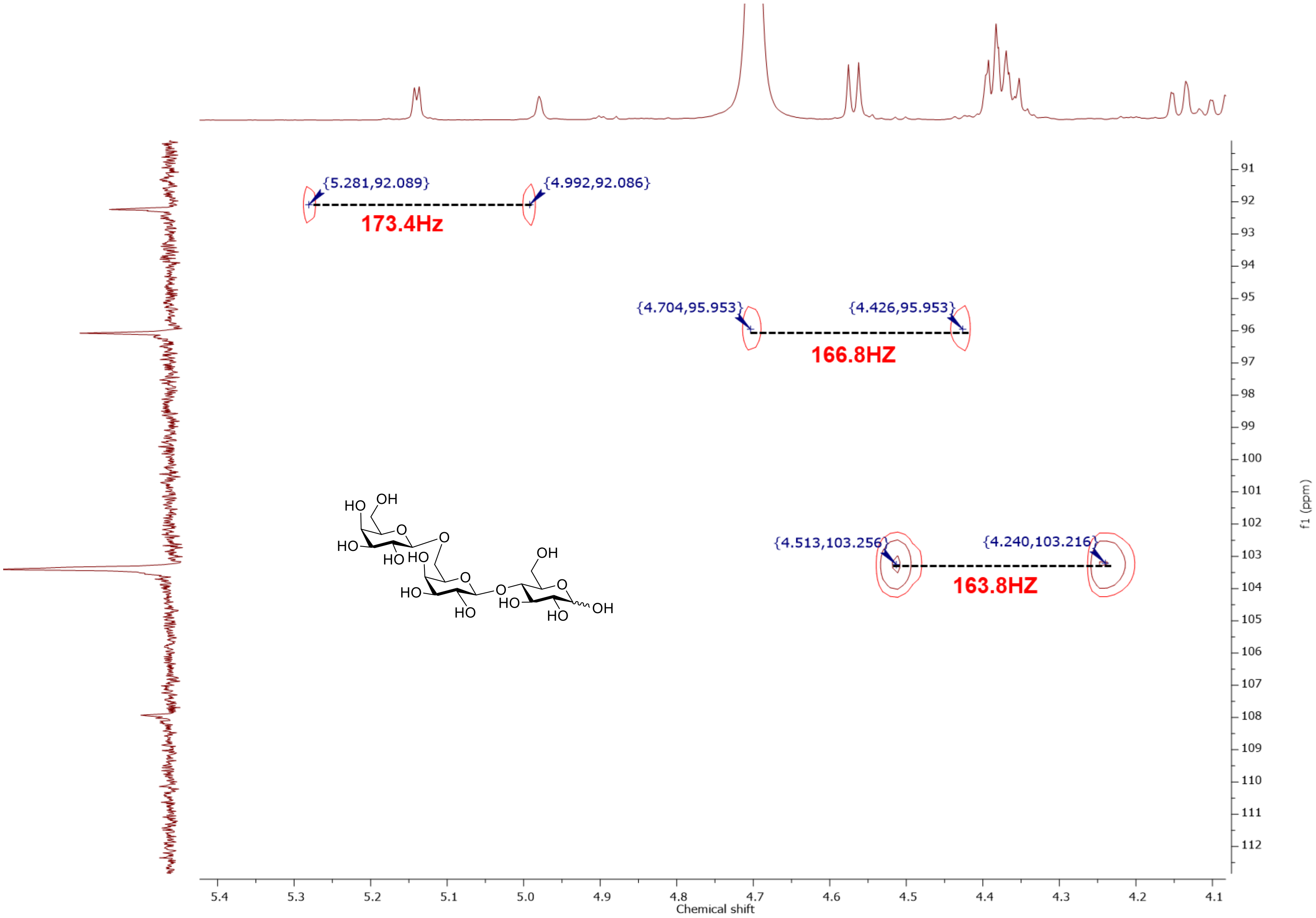

Supplement: Supplementary file 3 — Supplementary Data 1 [file 42004_2024_1152_MOESM3_ESM.pdf]
