# Supplementary material for: Cut-insert-stitch editing reaction (CIStER) sequence for surgical chemical glycan editing
Source: Commun Chem. 2024 Apr 2;7:73. doi: 10.1038/s42004-024-01152-z (PMC10987650; doi:10.1038/s42004-024-01152-z)

Supplementary Materials for

Cut-Insert-Stitch Editing Reaction (CIStER) Sequence for Surgical Chemical Glycan Editing

Sumit Sen, Suman Kundu, Sandip Pasari and Srinivas Hotha*

Department of Chemistry, Indian Institute of Science Education and Research Pune

Pune – 411 008, India

s.hotha@iiserpune.ac.in

MALDI-ToF Spectra of Representative Compounds

Supplementary Figure S47. MALDI-ToF Spectrum of compound **11c**

Calculated mass: 1880.5969 ($C_{108}H_{96}O_{29}Na$)

Observed mass:1882.6400

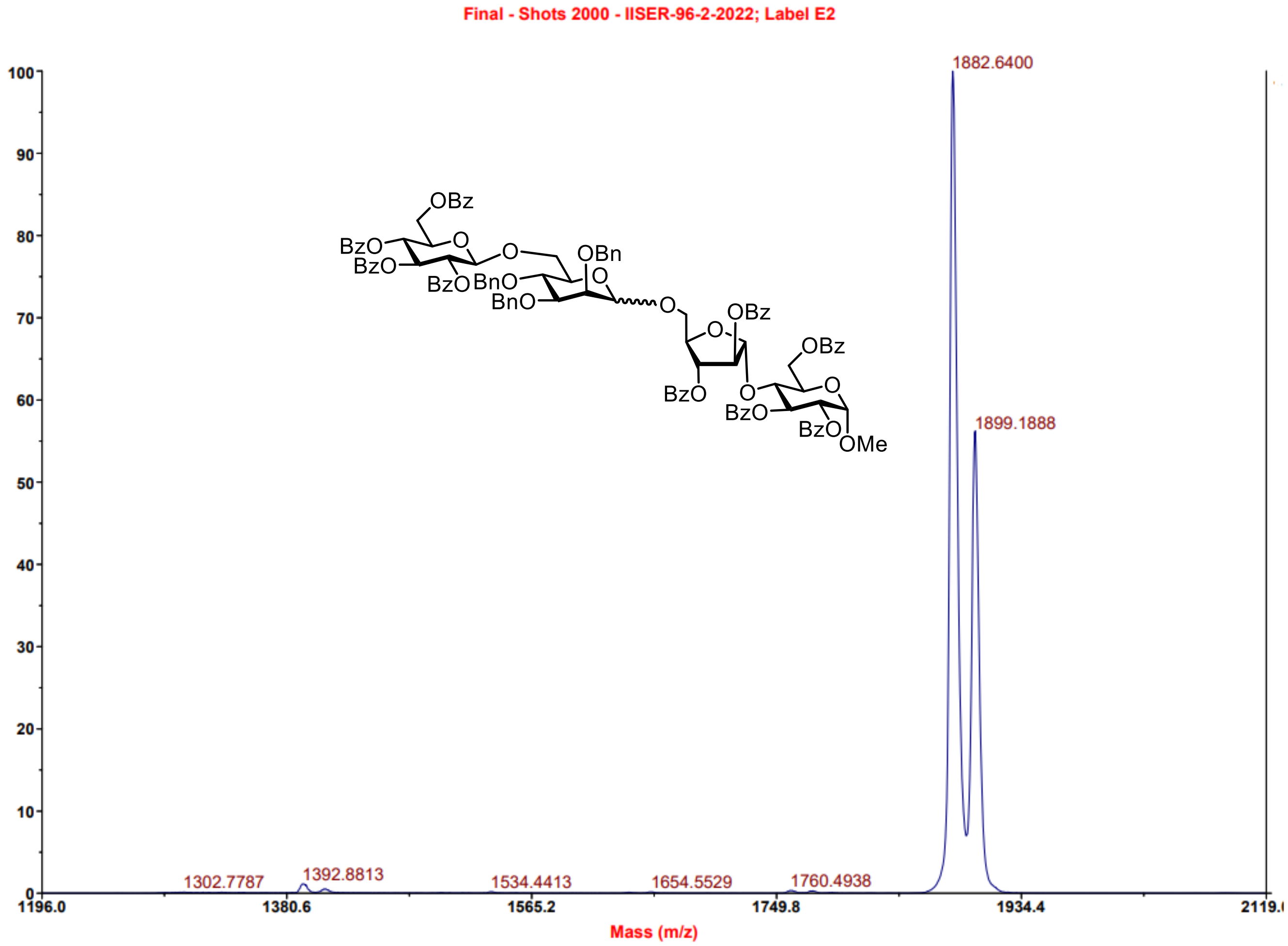

Supplementary Figure S48. MALDI-ToF Spectrum of compound **11b**

Calculated mass : 1972.696 ($C_{116}H_{108}O_{28}Na$)

Observed mass : 1972.028

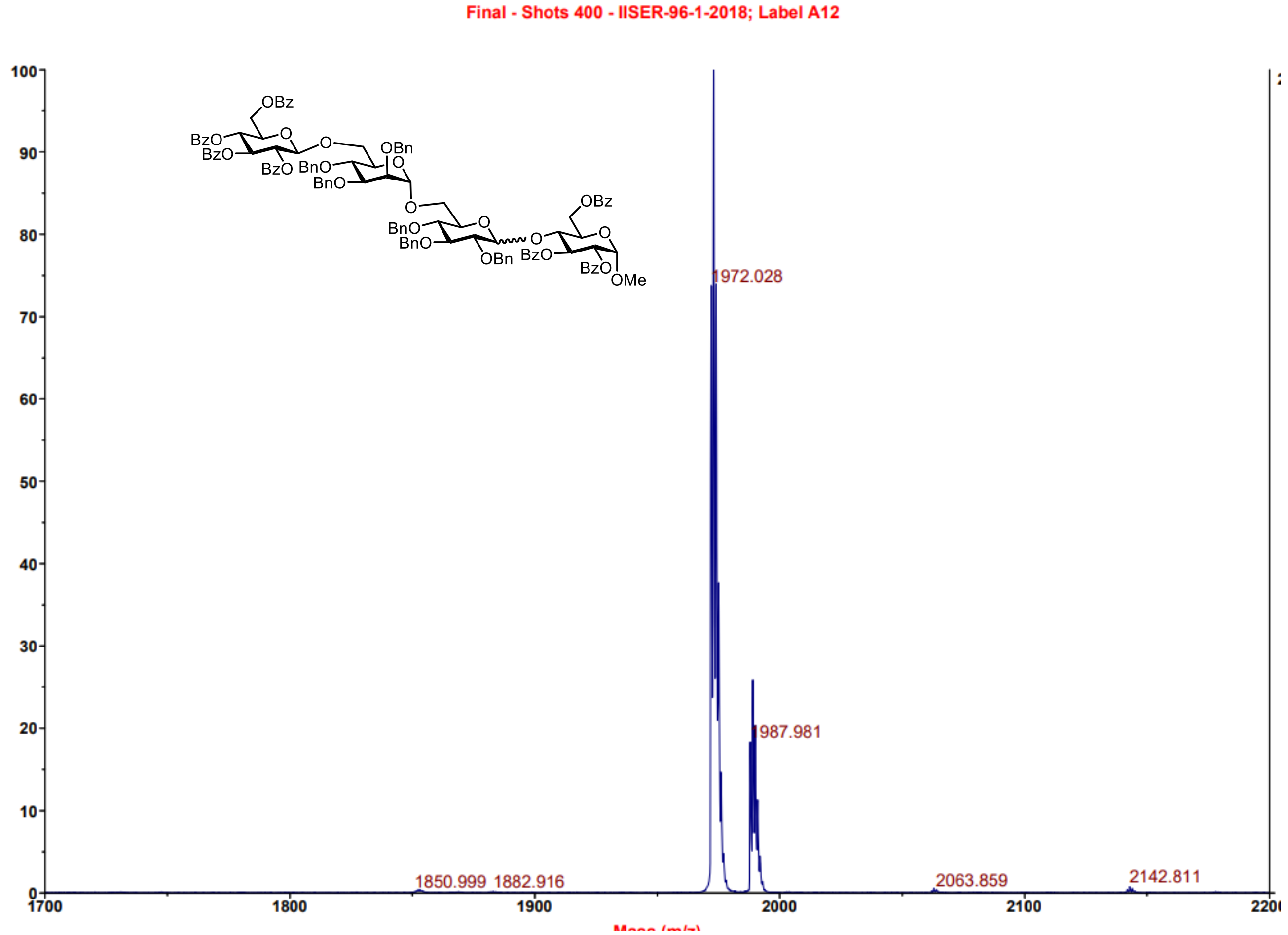

Supplementary Figure S49. MALDI-ToF Spectrum of compound **11a**

Calculated mass: 1986.675 ($C_{116}H_{106}O_{29}Na$)

Observed mass: 1986.635

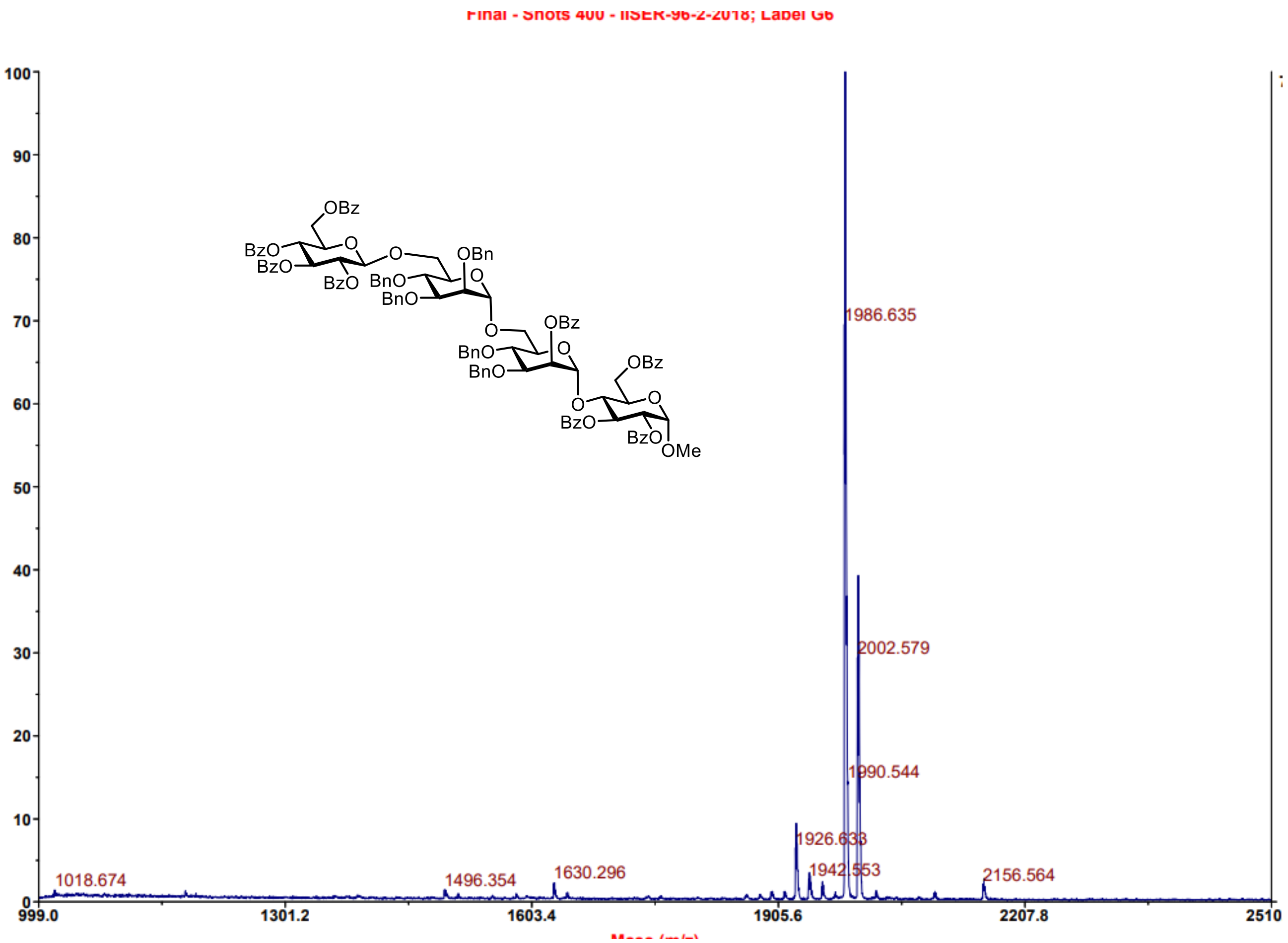

Supplementary Figure S50. MALDI-ToF Spectrum of compound **11d**

Calculated mass:1880.5969 ($C_{108}H_{96}O_{29}Na$)

Observed mass:1879.900

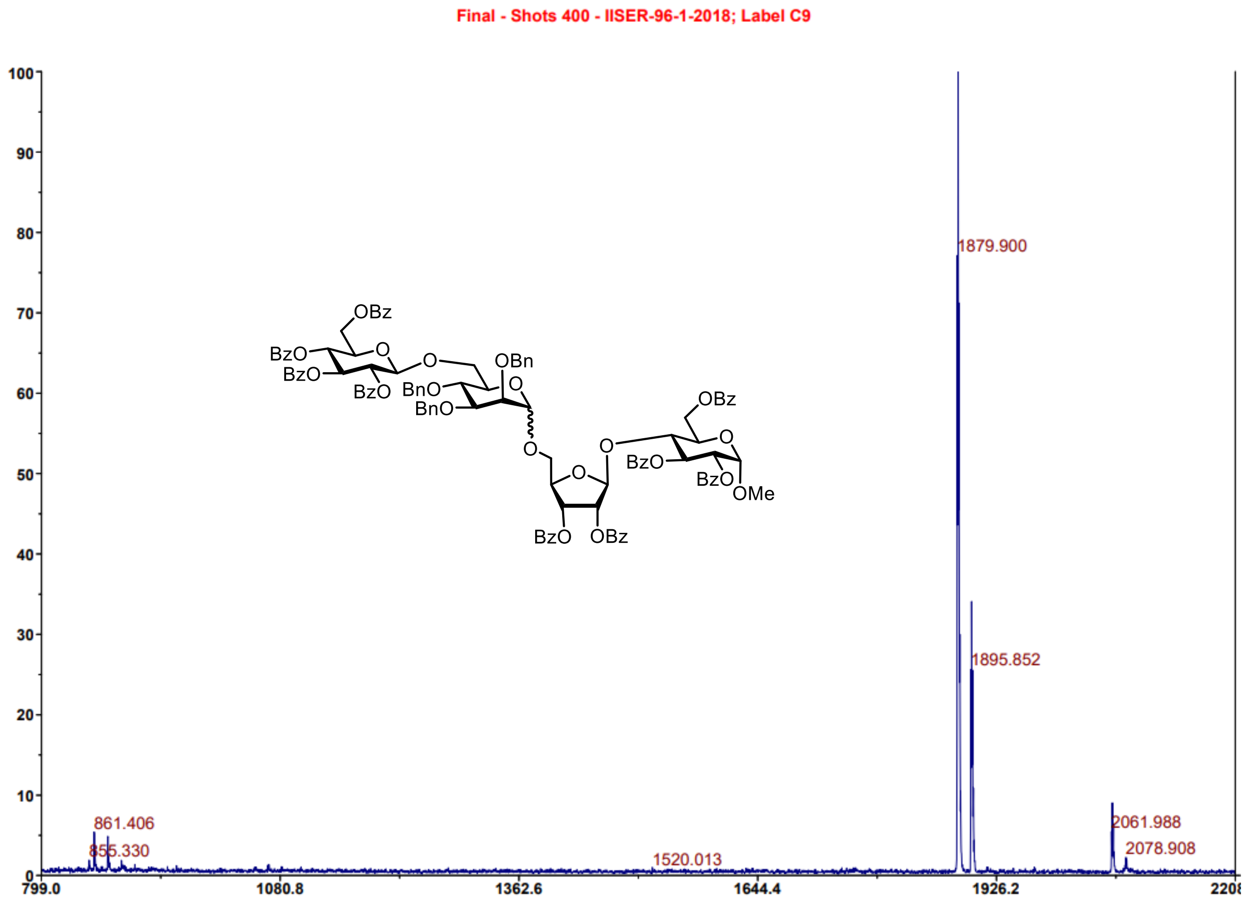

Supplementary Figure S51. MALDI-ToF Spectrum of compound **11e**

Calculated mass: 2219.688 ($C_{127}H_{112}O_{35}Na$)

Observed mass: 2219.529

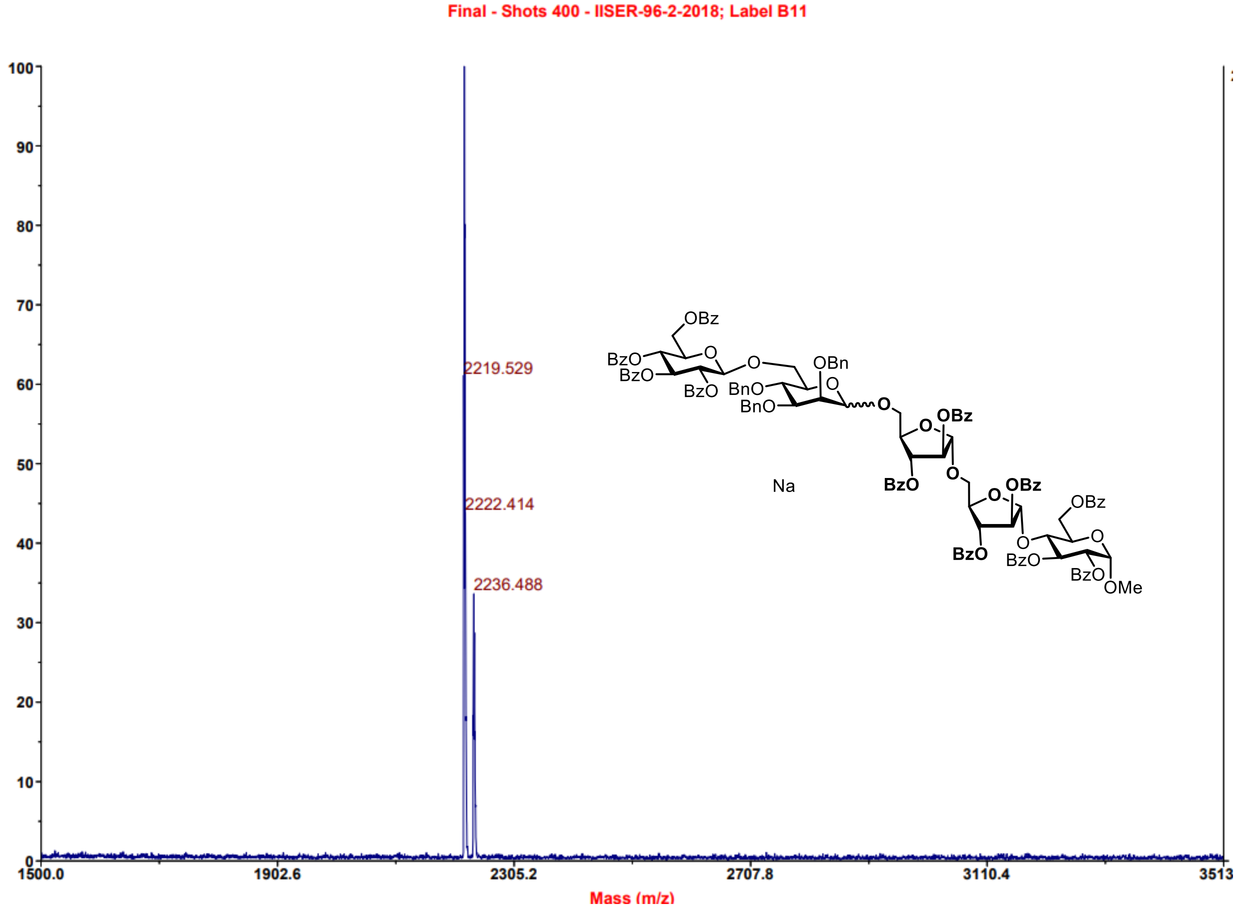

Supplementary Figure S52. MALDI-ToF Spectrum of compound **11f**

Calculated mass: 1973.101 ($C_{116}H_{108}O_{28}Na$)

Observed mass: 1973.321

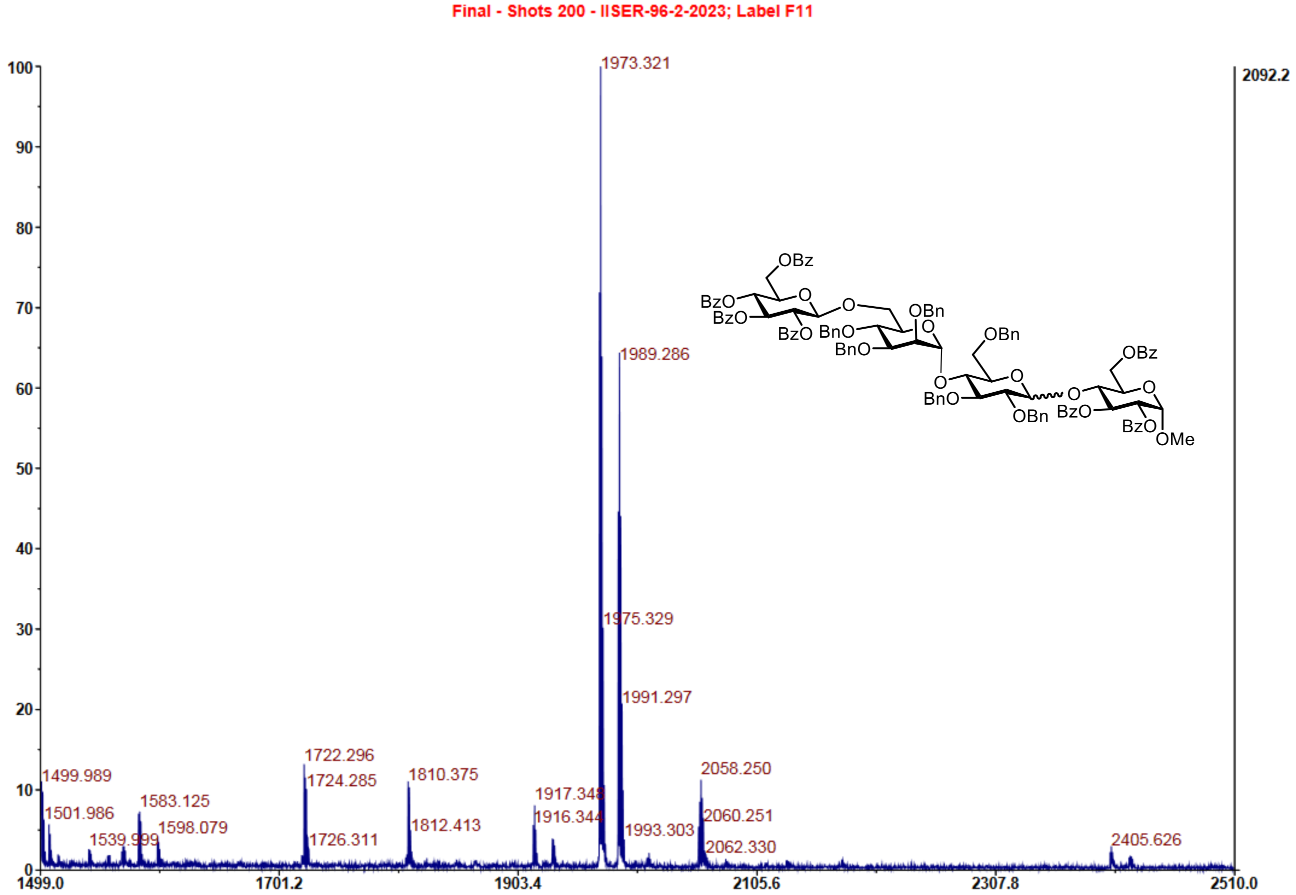

Supplementary Figure S53. MALDI-ToF Spectrum of compound **19c**

Calculated mass: 1368.740 ($C_{86}H_{104}O_{13}Na$)

Observed mass: 1368.028

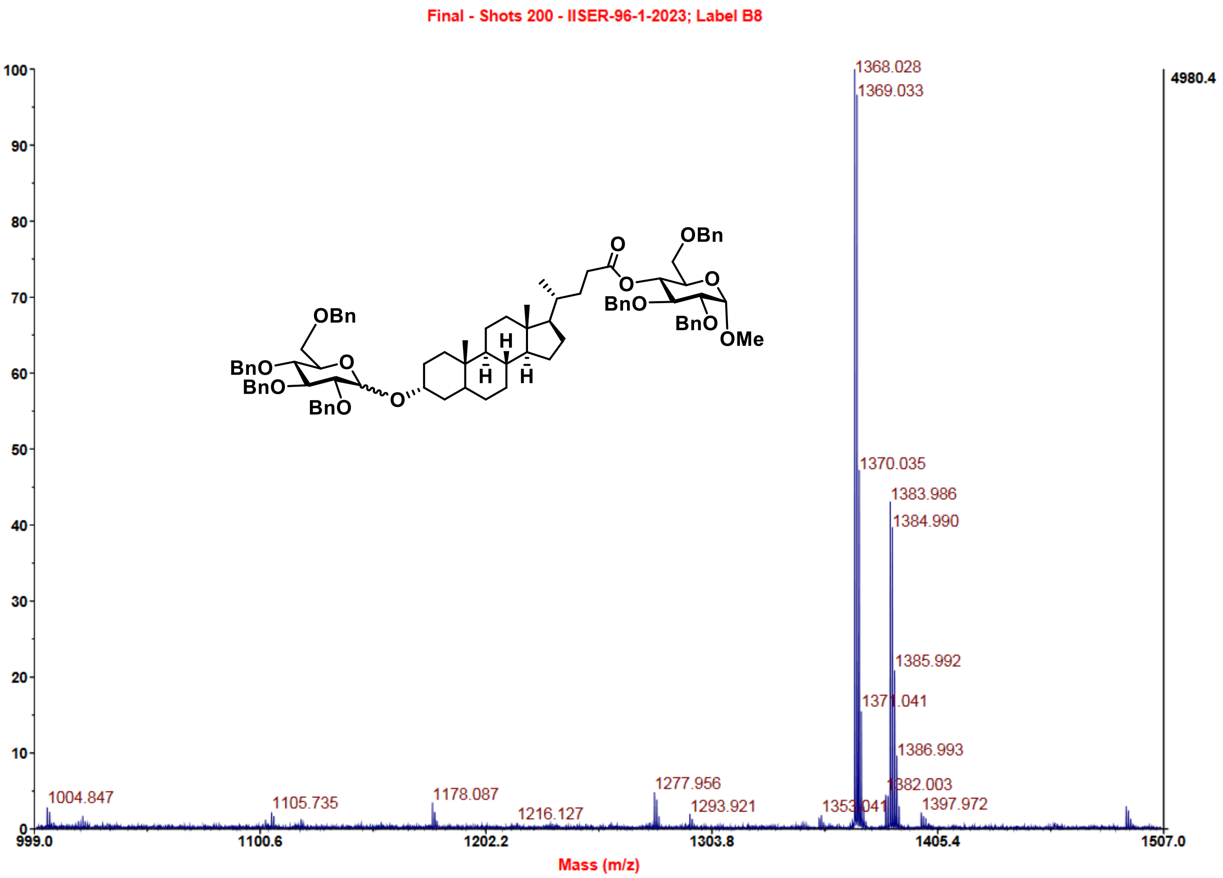

Supplementary Figure S54. MALDI-ToF Spectrum of compound **19a**

Calculated mass: 1123.518 ($C_{68}H_{76}O_{13}Na$)

Observed mass: 1123.824

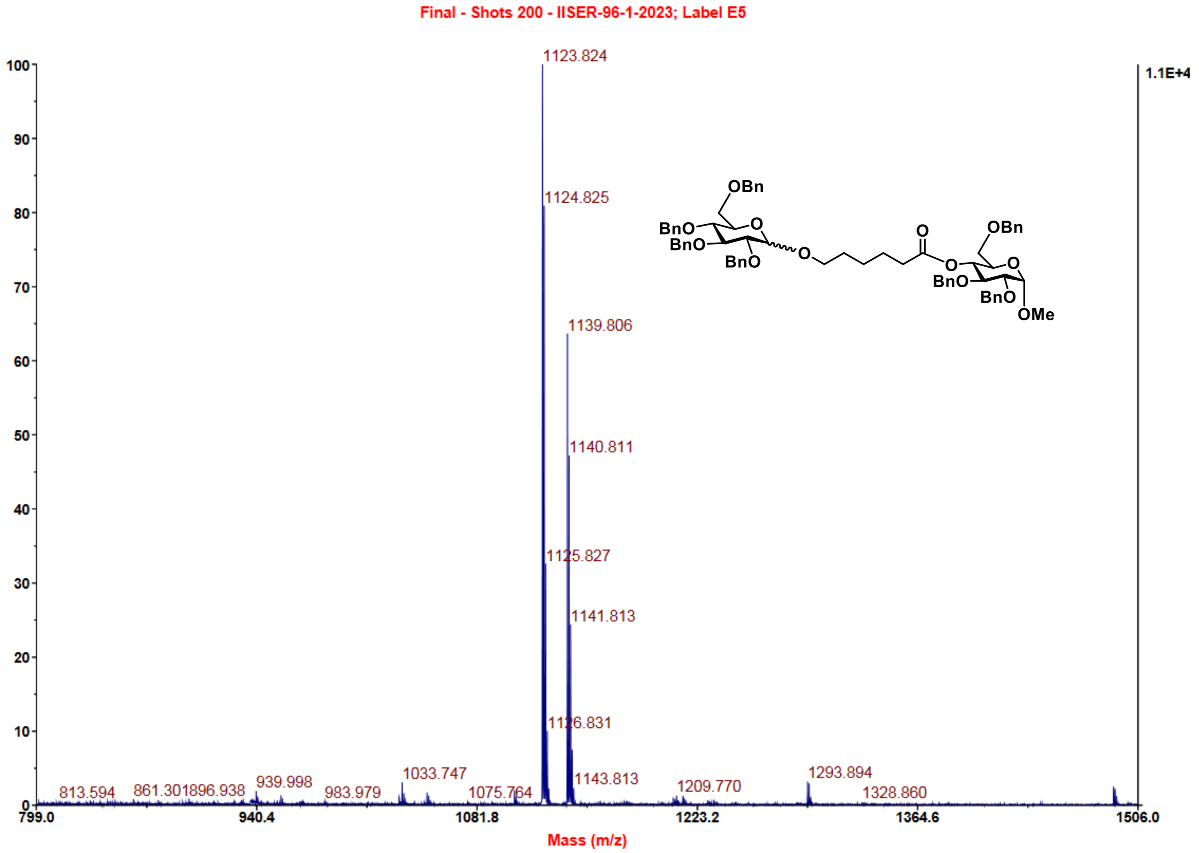

Supplementary Figure S55. MALDI-ToF Spectrum of compound **19b**

Calculated mass: 1230.519 ($C_{73}H_{77}O_{15}NNa$)

Observed mass: 1230.910

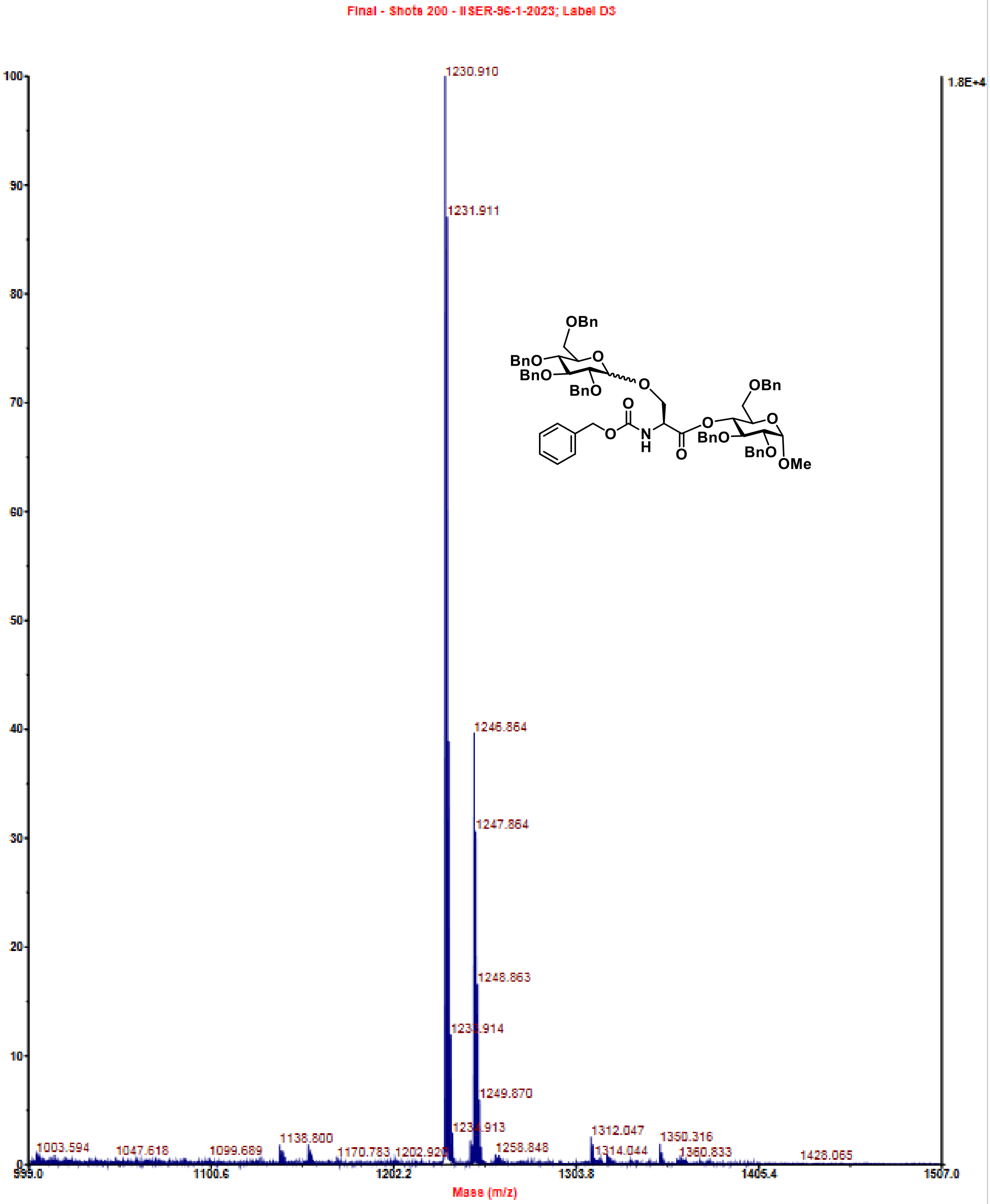

Supplementary Figure S56. MALDI-ToF Spectrum of compound **25**

Calculated mass**:** 3905.243 **(**$C_{224}H_{200}O_{62}Na$**)**

Observed mass: 3905.878

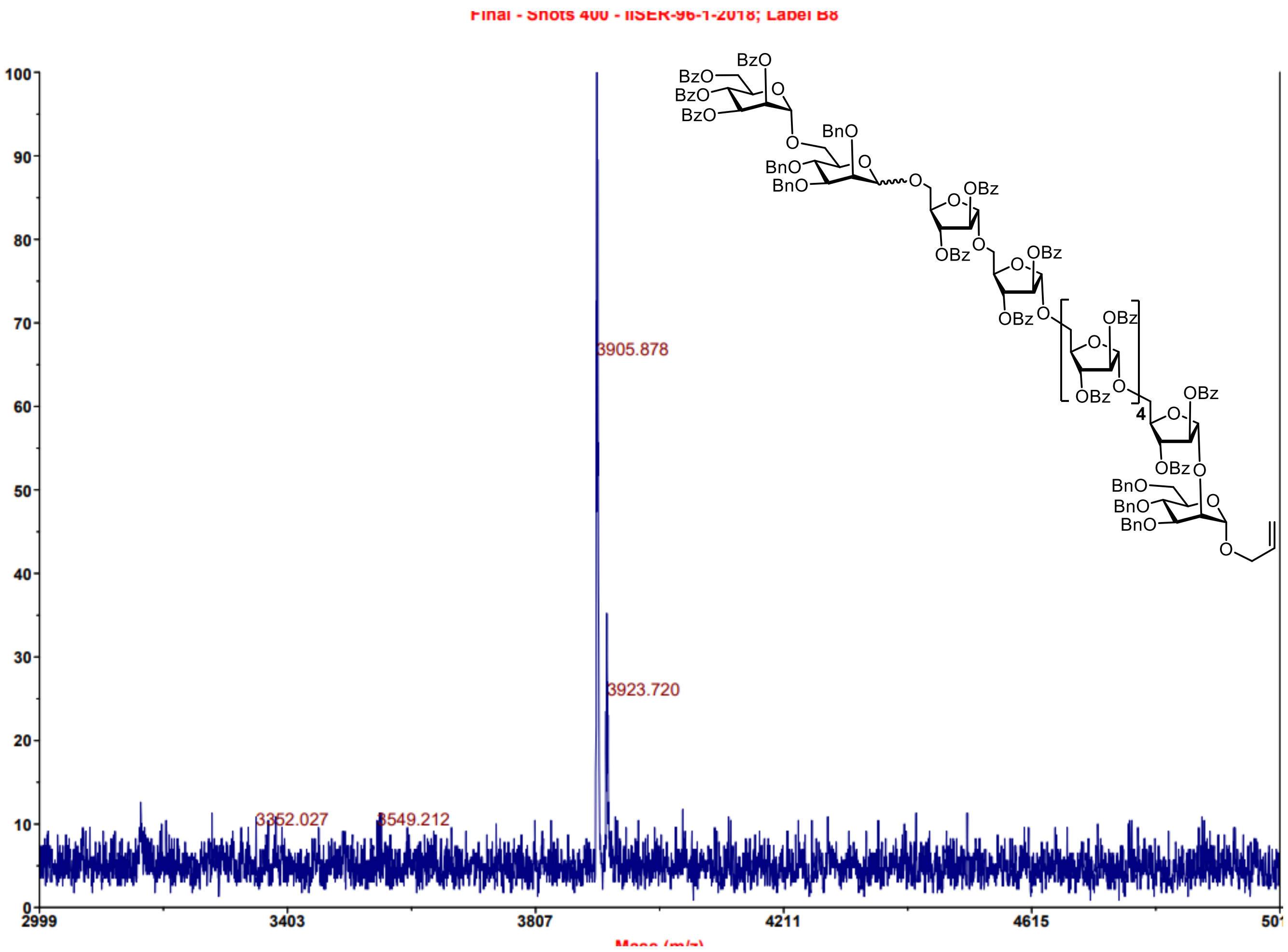

Supplementary Figure S57. MALDI-ToF Spectrum of compound **27**

Calculated mass: 4813.241($C_{278}H_{250}O_{75}Na$)

Observed mass:4813.145

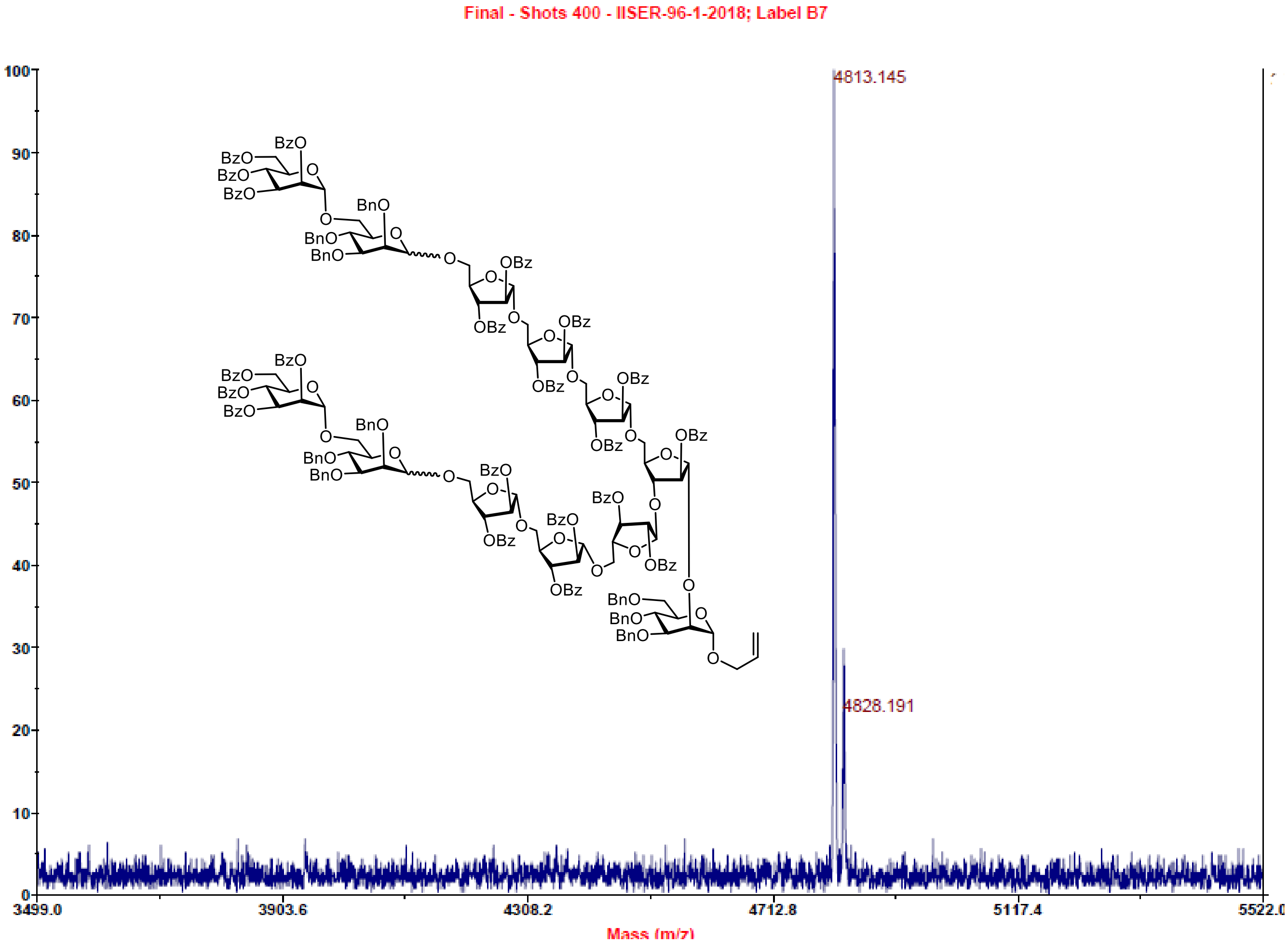

Supplementary Figure S58. MALDI-ToF Spectrum of compound **22**

Calculated mass: 527.1587 ($C_{73}H_{77}O_{15}NNa$)

Observed mass: 527.1583

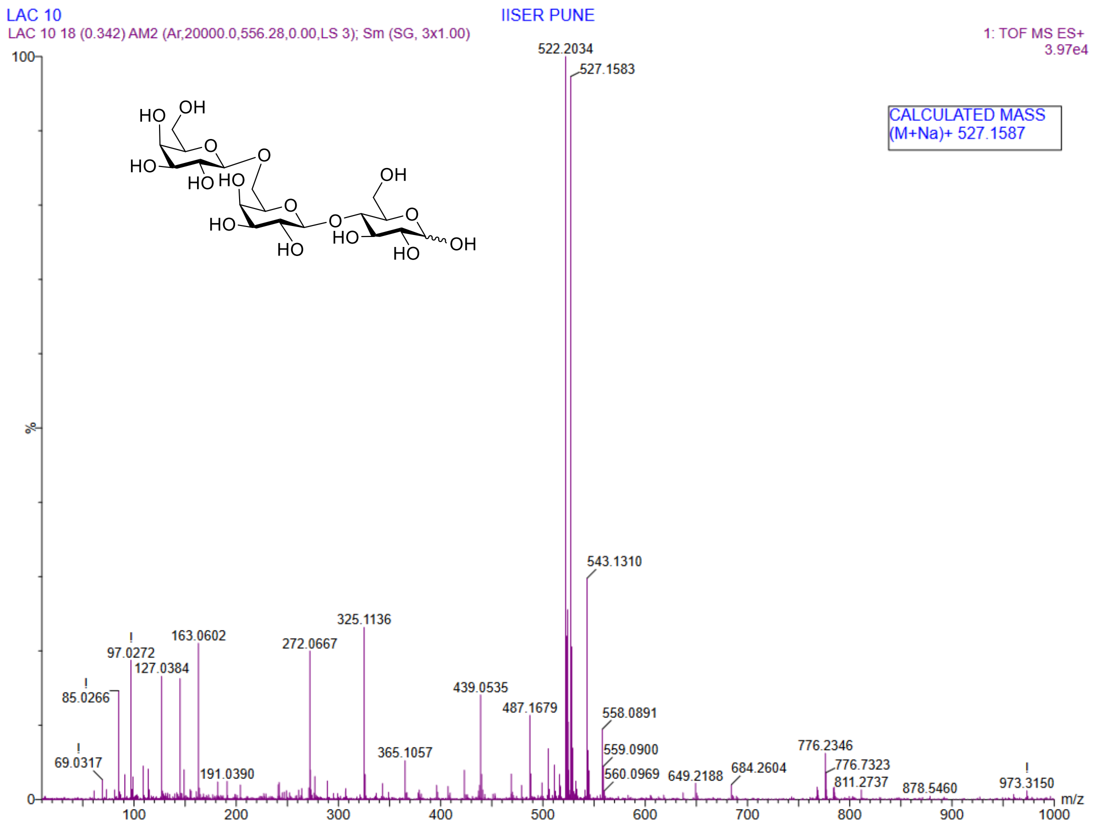

Supplement: Supplementary file 4 — Supplementary Data 2 [file 42004_2024_1152_MOESM4_ESM.pdf]
